# The High Prevalence of Low HDL-Cholesterol Levels and Dyslipidemia in Rural Populations in Northwestern China

**DOI:** 10.1371/journal.pone.0144104

**Published:** 2015-12-07

**Authors:** Pengfei Ge, Caixia Dong, Xiaolan Ren, Elisabete Weiderpass, Chouji Zhang, Haoqiang Fan, Jing Zhang, Yongrui Zhang, Jinen Xi

**Affiliations:** 1 Institute of Non-communicable Chronic Diseases, Gansu Center for Disease Control and Prevention, 230 Donggang Western Road, Chengguan District, 730000, Lanzhou, Gansu Province, China; 2 Department of Community Medicine, Faculty of Health Sciences, University of Tromsø, Tromsø, Norway; 3 Department of Research, Cancer Registry of Norway, Oslo, Norway; 4 Department of Medical Epidemiology and Biostatistics, Karolinska Institutet, Stockholm, Sweden; 5 Samfundet Folkhälsan, Helsinki, Finland; Weill Cornell Medical College in Qatar, QATAR

## Abstract

**Background:**

Dyslipidemia is a major health problem in China and an important modifiable cardiovascular disease (CVD) risk factor. This study aimed to describe the prevalence of dyslipidemia and low high density lipoprotein cholesterol (HDL-cholesterol) and associated risk factors among adults in rural northwest China.

**Methods:**

In a cross-sectional analyses involving 2,980 adults aged >18 years, information on the demographics, cigarette smoking, alcohol consumption, education, and medical history was collected via face-to-face interviews. Blood samples were collected to determine total cholesterol (TC), low-density lipoprotein cholesterol (LDL-cholesterol), and HDL-cholesterol, and triglycerides (TG) levels.

**Results:**

The prevalence of high TC, high LDL-cholesterol, low HDL-cholesterol, and high TG were 1.0%, 0.6%, 60.9%, and 13.7%, respectively. TC, LDL-cholesterol, and TG increased with age in females. Elevated TC was more common in females than in males. The prevalence of low HDL-cholesterol was 67.6% in males and 55.4% in females. Current smokers, those with less education, those who were overweight or obese, and those with large waist circumference were more likely to have low HDL-cholesterol (p<0.05). Multivariable regression showed that male gender showed an association with low HDL-cholesterol (OR 2.10, 95%CI 1.68–2.61), age ≥60 years (OR 0.80, 95% CI 0.64–0.99), BMI (BMI = 24–27.9, OR 1.27, 95%CI 1.04–1.54, p = 0.02 and BMI≥28, OR 1.56, 95%CI 1.10–2.20, p = 0.01) and enlarged waist circumference (OR 2.10, 95%CI 1.51–2.92). Non-alcohol drinker was associated with low HDL-cholesterol levels (OR 0.72, 95%CI 0.53–0.99, p = 0.04).

**Conclusions:**

This study found that the prevalence of low HDL-cholesterol was 67.6% and 55.4% for males and females. Male gender, non-alcohol drinker, BMI and central obesity were important risk factors for low HDL-cholesterol in Chinese adults.

## Introduction

Cardiovascular disease (CVD) is the leading cause of mortality worldwide. Over the next 20 years, CVD morbidity and mortality in China is projected to increase in absolute number [1, 2]. It has been estimated that the prevalence of CVD will increase by more than 50% over the next 20 years in China [2]. Dyslipidemia is a common health problem in developing countries [3, 4], including China [5] and has been shown to be associated with an increased risk of developing CVD [6]. However, the prevalence of dyslipidemias has been found to differ among populations [5, 7]. Plasma lipid concentrations are considered to be important risk factors for atherosclerosis and related vascular diseases [8, 9]. The critical role of dyslipidemia in the pathogenesis of atherosclerosis has been confirmed in Western and Asian populations [10, 11]. According to the Chinese National Nutrition and Health Survey of 2002, the prevalence of dyslipidemia in Chinese adults at that time was 18.6% [7].

Low high-density lipoprotein cholesterol (HDL-cholesterol) concentration has been shown to be associated with an increased risk of developing CVD, especially coronary heart disease (CHD) [12–15]. Large cohort studies have shown a strong inverse correlation between HDL-cholesterol levels and CVD risk and identified HDL-cholesterol an independent predictor of CHD risk [12, 16, 17]. Few studies have assessed which risk factors are associated with low HDL-cholesterol concentrations in China. The aim of this study was to describe the prevalence of dyslipidemia and low HDL-cholesterol and the risk factors associated with low HDL-cholesterol among adults living in northwest China.

## Methods

### Study design and participants

The Chinese Non-Communicable Chronic Disease and Risk Factor Surveillance project is a population-based cross-sectional survey of national disease surveillance points (DSP) from 30 provinces. The aim of the project is to determine the prevalence of chronic diseases, risk factors, and to explore their potential relationships. Detailed information about the study design and data collection procedures have been reported previously [18].

We used surveillance data from August to November 2010 from five DSPs in Gansu provinces such as Jingtai, Lintan, Maiji, Ganzhou, and Dunhuang. Six hundred residents aged ≥18 years were sampled from each surveillance point. At each surveillance point, a four-stage cluster-sampling plan was used to recruit participants; (1). Using probability proportional to size (PPS) sampling, four townships or streets were sampled from each surveillance point; (2). Also using PPS, three villages or communities were sampled from each township or street; (3). One resident group of 50 households was chosen from each village or community by simple randomized sampling; (4). Kish table chose one individual from each designated household. For the subjects sampled who refused to join the survey, replaced samples should be supplemented by selecting the neighbor family who had the same family structure. Finally, 3,000 adults aged were included in this study. Written informed consent was obtained from all participants and the study obtained official ethical approval by the Ethics Committee of the Chinese Center for Disease Control and Prevention.

### Data Collection

#### Questionnaire interview

Data were collected at the physical examination stations in local health centers or in temporary assessment clinics set up within the local residential center (village or street committee). During the survey visit, trained research staff members administered a standardized questionnaire and via face-to-face interviews collected information on the demographics, cigarette smoking, alcohol consumption, education level, and medical history in face-to-face interviews.

#### Anthropometric and blood pressure measurement

The participants’ weight, height, and waist circumference were measured using standard anthropometric techniques [18]. Height and waist circumference were measured to the nearest 0.1 cm, and body weight to the nearest 0.1 kg. Weight and height scales were checked with calibrated before the measurement. Body mass index (BMI) was calculated as body weight divided by height squared (kg/m^2^). Waist circumference was measured halfway between the lowest rib margin and the iliac crest. Blood pressure was measured after the participants had rested for 5 minutes. Three reading were taken for systolic and diastolic blood pressure (BP) according to the 1999 World Health Organization/International Society of Hypertension guidelines for the management of hypertension [19]. The participants left arm was placed at heart level in a sitting position and blood pressure was measured with a digital sphygmomanometer (Omron 770A, Omron Corporation, Kyoto, Japan). The averages of the last two readings of systolic and diastolic pressure were recorded and the first reading was excluded.

#### Laboratory assay

After an overnight fast, 5ml blood was collected from the participant’s antecubital vein by local hospital technicians in the morning. The blood samples were collected in serum separation tubes and kept 45 minutes and then centrifuged at 3000 rpm for 10 min at room temperature. The serum fraction was collected and kept between -20 and -30°C at the field site. After the fieldwork was completed, all frozen serum samples were transferred by air to the designed national qualified laboratory in Shanghai within 12 hours where they were stored at -80°C until analysis. Total cholesterol (TC), triglycerides (TG), HDL-cholesterol, and low-density lipoprotein cholesterol (LDL-cholesterol) were measured using the enzymatic method with an automated biochemistry analyzer (Architect ci16200, Abbott Laboratories, Abbott Park, IL) in accordance with the international quality standard. Reagents from the same batch were used to minimize laboratory variability [20].

### Definitions

Education was recorded as completed years of schooling according to the following three categories: ≤6 years (elementary school), 7–12 years (secondary school/junior high school) and >12 years (high school and above). Overweight and general obesity was classified using World Health Organization-recommended BMI cut-off points. Overweight was defined as BMI 25.0–29.9kg/m^2^ and obesity was defined as BMI ≥30kg/m^2^. Central obesity was defined as having a waist circumference above >95cm for males and >90cm for females. Hypertension was defined as having a systolic BP ≥140 mmHg and/or diastolic BP ≥90 mmHg, or receiving treatment for hypertension [19]. Current smoking was defined as daily smoking. Current drinking was defined as more than three times or three drinks per month.

Blood lipid levels were categorized as ideal, marginally high, or high in accordance with the Chinese criteria [21]. TC <5.18 mmol/L (<200 mg/dl) was considered ideal, 5.18–6.21 mmol/L (200–239 mg/dl) as marginally high, and ≥6.22 mmol/L (≥240 mg/dl) as high. LDL-cholesterol <3.37 mmol/L (<130 mg/dl) was considered ideal, 3.37–4.13 mmol/L (130–159 mg/dl) as marginally high, and ≥4.14 mmol/L (≥160 mg/dl) as high. HDL-cholesterol <1.04 mmol/L (<40 mg/dl) was considered low and ≥1.04 mmol/L (≥40 mg/dl) as ideal. TG, <1.70 mmol/L (<150mg/dl) was considered ideal, 1.71–2.25 mmol/L (150–199 mg/dl) as marginally high, and ≥2.26 mmol/L (≥200 mg/dl) as high. Dyslipidemia was defined as high TC, and/or high LDL-cholesterol and/or low HDL-cholesterol, and/or high TG, and having received treatment for dyslipidemia in the previous 2 weeks.

### Quality control

To ensure the quality of the survey, the provincial and local Centers for Disease Control and Prevention created quality control networks to monitor survey efficacy for each surveillance point. The responsibilities and roles of all participants were defined. A proposal for quality control was developed and implemented. Qualified instructors trained all investigators before joining the field survey team. The nurses working on this study were also trained and evaluated before they performed the anthropometric measurements. The percentage of displaced or refused samples was kept at less than 6% and percentage of the replaced samples would be the same. National and provincial quality control teams ensured quality control of the data. Team leaders reviewed the completed questionnaires before submitting them to the headquarters for data entry.

### Statistical analysis

The percentage distribution of participants TC, LDL-cholesterol, HDL-cholesterol, and TG levels were calculated stratified by sex and age. χ2 tests and χ2 trend test was used to detect differences in proportions across sex and age, respectively. Differences in serum concentrations of TC, LDL-cholesterol, HDL-cholesterol, and TG by sex and age were determined using t-test and ANOVA. Crude differences in the prevalence of dyslipidemia across participant characteristics (e.g., males versus females) between groups were compared using t-test for continuous and χ2 tests for categorical variables. The data were expressed as mean ± standard deviation (SD) for continuous variables and as percentages for categorical. The adjusted odds ratios (OR) and 95% confidence intervals (CI) of the prevalence of low HDL-cholesterol with different risk factors were determined using multivariable logistic regression model. Sex, age, education, smoking, alcohol, BMI, and waist circumference were expressed as independent variables within the multivariable model. All tests were two-sided and statistical significance was set at *p*< 0.05. Data were analyzed using SAS software (version 9.1; SAS Institute, Cary, NC, USA).

## Results

### General characteristics

Of 3,000 participants who were enrolled in this study, 20 participants (0.7%) were excluded because of incomplete data on bloods leaving 2,980 (1,348 males and 1632 females) for final analyses. The general characteristics of the study population are shown in Table 1. The participants mean age was 46.3 ± 14.2 years, without sex imbalance. Educational levels were low; approximately two-fifths of the participants had elementary school education or no formal education, 43% attended secondary school, and 17% had tertiary education. Males had a higher education attainment (22.9%) than females (12.1%). Higher smoking (60.5%) and drinking (15.5%) was seen for males than in females. Males also had a higher waist circumference (82.6 ± 9.5 cm) than females (78.7 ± 9.2 cm). Roughly, 37.5% of the participants (37.1% males and 37.8% females) were hypertensive and 8.6% were diabetic (9.1% males and 8.2% females).

**Table 1:** General characteristics of study participants (mean ± SD).

Characteristics	Men	Women	P value	Total
	n = 1348	n = 1632		n = 2980
Age (years)	46.6±14.4	46.1±14.0	0.313	46.3±14.2
SBP (mmHg)	133.2±19.7	134.1±22.7	0.801	133.7±21.5
DBP (mmHg)	82.0±11.6	81.8±11.3	0.234	81.9±11.4
BMI (kg/m^2^)	24.0±3.4	23.84±3.4	0.282	23.9±3.4
Waist circumference (cm)	82.6±9.5***	78.7±9.2	< 0.001	80.5±9.5
FBG (mmol/L)	5.2±1.5	5.2±1.3	0.518	5.2±1.4
Education				
≤6 years (elementary)%	25.4***	51.4	< 0.001	39.6
7–12 years (secondary)%	51.7***	36.5	< 0.001	43.4
>12 years (high)%	22.9***	12.1	< 0.001	17.0
Current Smoking (%)	60.5***	0.5	< 0.001	27.7
Current drinking (%)	15.5***	0.6	< 0.001	7.3
Hypertension (%)	36.6	36.8	0.888	36.7
Diabetes (%)	9.1	8.2	0.416	8.6

***, *p*< 0.001 for the difference between men and women.

The results were expressed as mean±SD (standard deviation); BMI, body mass index; SBP, systolic blood pressure; DBP, diastolic blood pressure; FBG, fasting blood glucose.

Concentrations of serum lipids cholesterol TC, LDL-cholesterol, HDL-cholesterol, and TG Serum concentrations of total cholesterol, LDL-cholesterol, HDL-cholesterol, and TG are shown in Table 2. The mean TC for both males and females was 3.59–0.89 mmol/L. Males had a slightly higher LDL-cholesterol (1.95–0.65 mmol/L) than females (1.92–0.68 mmol/L). Males also had a lower HDL-cholesterol (0.95–0.25 mmol/L) than females (1.01–0.26 mmol/L). Males TG levels were also higher (1.56–1.23 mmol/L) than females. TC, LDL-cholesterol, and TG levels increased with age in females but this trend was not observed in males. The mean TG levels of males between age 18–44 year group was slightly than females at the same age, and the reverse was found for participants over 60 years of age.

**Table 2:** Serum concentrations of total cholesterol, LDL-cholesterol, HDL-cholesterol, and TG (Mean ± SD).

Indicators	Men	*P* value	Women	*P* value
TC (mmol/L)	3.59±0.89		3.59±1.00	0.961*
Age (years)		0.291†		<0.001†
18 to 44	3.55±0.93		3.35±0.92	
45 to 59	3.62±0.87		3.75±1.0	
>60	3.63±0.82		3.93±1.1	
LDL-C (mmol/L)	1.95±0.65		1.92±0.68	0.136*
Age (years)		0.069†		<0.001†
18 to 44	1.91±0.25		1.77±0.64	
45 to 59	1.98±0.66		2.04±0.68	
>60	2.00±0.63		2.09±0.74	
HDL-C (mmol/L)	0.95±0.25		1.01±0.26	<0.001*
Age (years)		0.164†		0.515†
18 to 44	0.94±0.25		1.00±0.26	
45 to 59	0.96±0.26		1.02±0.28	
>60	0.98±0.25		1.01±0.24	
TG (mmol/L)	1.56±1.23		1.41±1.19	<0.001*
Age (years)		0.004†		<0.001†
18 to 44	1.66±1.36		1.25±1.23	
45 to 59	1.55±1.23		1.50±1.21	
>60	1.36±0.81		1.67±0.95	

*p value for difference between men and women.

^†^P value across age groups.

The results were expressed as mean±SD (standard deviation). TC, total cholesterol; LDL-C, low-density lipoprotein cholesterol; HDL-C, high-density lipoprotein cholesterol; TG, triglycerides.

### Distribution of serum lipids

The prevalence of serum lipids by sex and age is shown in Table 3. Of 2,980 participants 1.0% had high TC (≥6.22 mmol/L), 0.6% had high LDL-cholesterol (≥4.14 mmol/L), 60.9% had low HDL-cholesterol (<1.04 mmol/L), and 13.7% had high TG (≥2.26 mmol/L), respectively. The prevalence of borderline high TC was 4.6% (5.18–6.21 mmol/L), 2.5% for LDL-cholesterol (3.37–4.13 mmol/L), and 11% for TG (1.70–2.25 mmol/L), respectively. The prevalence of borderline high and high TC was 3.8% and 0.8% in males and 5.5% and 1.1% in females. The proportion of individuals with borderline high and high TC concentrations increased with age in females. The prevalence of borderline high and high TC was higher in female than in males. The prevalence of borderline high and high LDL-cholesterol was 2.6% and 0.5% in males and 2.4% and 0.6% in females. The prevalence of high LDL-cholesterol increased with age in females. The prevalence of low HDL-cholesterol was 67.6% in males and 55.4% in females (*p*<0.001 for the difference between males and females *p*<0.001). The prevalence of borderline high and high TG was 11.5% and 16.2% in males and 10.5% and 11.5% in females (*p*<0.001 for the difference between genders *p*<0.001). The prevalence of borderline high and high TG increased with age in females (*p*<0.001). In proportion of mean with TG levels below 1.70 increased with age (*p*< 0.001).

**Table 3:** Percentage distribution of serum lipids by gender and age.

Indicators	Men (%)			*P* value	Women (%)			* *p* value
TC (mmol/L)	<5.18	5.18–6.21	≥6.22		<5.18	5.18–6.61	≥6.22	
Total	95.4	3.8	0.8		93.4	5.5	1.1	0.07*
Age (years)				0.212†				0.001†
18 to 44	95.8	2.9	1.3		95.8	3.4	0.8	
45 to 59	94.7	4.8	0.4		92.5	6.6	0.9	
>60	95.4	3.8	0.8		88.7	8.8	2.5	
LDL-C (mmol/L)	<3.37	3.37–4.13	≥4.14		<3.37	3.37–4.13	≥4.14	
Total	96.9	2.6	0.5		97	2.4	0.6	0.89*
Age (years)				0.934†				0.083†
18 to 44	97.3	2.3	0.5		97.8	1.9	0.3	
45 to 59	96.5	2.9	0.7		97	2.3	0.7	
>60	96.7	2.9	0.4		94.7	3.9	1.4	
HDL-C (mmol/L)	≥1.04		<1.04		≥1.04		<1.04	
Total	32.4		67.6		44.6		55.4	<0.001*
Age (years)				0.428†				0.818†
18 to 44	31.3		68.7		44.2		55.8	
45 to 59	32		68.0		45.6		54.4	
>60	35.7		64.3		43.7		56.3	
TG (mmol/L)	<1.70	1.70–2.25	≥2.26		<1.70	1.70–2.25	≥2.26	
Total	72.3	11.5	16.2		78	10.5	11.5	<0.001*
Age (years)				<0.001†				<0.001†
18 to 44	68.7	11	20.3		84.8	8.8	6.4	
45 to 59	71.7	13.2	15.1		74.6	10.8	14.6	
>60	72.3	11.5	16.2		65.8	14.4	19.7	

TC, total cholesterol; LDL-C, low-density lipoprotein cholesterol; HDL-C, high-density lipoprotein cholesterol; TG, triglycerides.

*p value for difference between men and women.

^†^P for trend across age groups.

### Factors associated with low HDL-cholesterol

When using WHO recommended BMI cut-off points, prevalence of low HDL-cholesterol by selected characteristics and adjusted odds ratio (95% CI) of low HDL-cholesterol prevalence including hypertension and diabetes is shown in Table 4. The prevalence of low HDL-cholesterol was higher in males than females (*p* = 0.001). A higher prevalence of low HDL-cholesterol was found among current smokers (*p* = 0.001), those with lower educational attainment (*p* = 0.032), those who were overweight or obese (*p*<0.001), and those who had a large waist circumference (*p*<0.001).

**Table 4:** Prevalence of low HDL-cholesterol by selected characteristics and adjusted odds ratio (95% CI) of low HDL-cholesterol prevalence including hypertension and diabetes using WHO recommended BMI cut-off points.

Indicators	Prevalence, †n (%)	*P* value	Adjusted OR (95% CI)	* *P* value	Adjusted OR (95% CI)	* *P* value
Sex		< 0.001‡				
Female	904 (55.4)		1.00(reference)		1.00(reference)	
Male	911 (67.6)		2.10 (1.68–2.61)	<0.001	2.10 (1.68–2.62)	<0.001
Age (years)		0.822&				
18 to 44	864 (61.5)		1.00(reference)		1.00(reference)	
45 to 59	616 (60.5)		0.86 (0.72–1.02)	0.09	0.88(0.74–1.06)	0.17
≥60	335 (60.3)		0.80 (0.64–0.99)	0.04	0.84 (0.67–1.06)	0.13
Education		0.032&				
≤6 years (elementary)	708 (59.9)		1.02 (0.80–1.29)	0.89	1.02 (0.81–1.30)	0.86
7–12 years (secondary)	772 (59.8)		0.82 (0.65–1.02)	0.07	0.82 (0.65–1.02)	0.07
>12 years (high)	335 (66.1)		1.00(reference)		1.00(reference)	
Current smoking		0.001‡				
No	1275 (59.1)		1.00(reference)		1.00(reference)	
Yes	540 (65.5)		0.85 (0.67–1.07)	0.17	0.85 (0.67–1.07)	0.17
Current drinking		0.542‡				
No	1678 (60.8)		1.00(reference)		1.00(reference)	
Yes	137 (62.8)		0.73 (0.54–1.00)	0.05	0.73 (0.54–1.00)	0.05
BMI, kg/m2		<0.001&				
<25	1118 (57.0)		1.00(reference)		1.00(reference)	
25–29	598 (67.6)		1.07 (0.86–1.32)	0.56	1.10(0.88–1.35)	0.43
≥30	99 (74.4)		1.31 (0.81–2.12)	0.27	1.37 (0.85–2.23)	0.20
Waist circumference, cm		<0.001&				
Women <80 and men <85	953 (54.8)		1.00(reference)		1.00(reference)	
Women 80–89 and men 85–94	606 (68.1)		1.79 (1.46–2.18)	<0.001	1.82 (1.48–2.22)	<0.001
Women≥90 and men≥95	256 (72.9)		2.10 (1.51–2.92)	<0.001	2.16 (1.55–3.02)	<0.001
Hypertension		0.717‡	-	-		
No	1130(60.7)		-	-	1.00(reference)	
Yes	685(61.3)		-	-	0.88(0.74–1.05)	0.14
Diabetes		0.680‡	-	-		
No	1656(60.8)		-	-	1.00(reference)	
Yes	159(62.1)		-	-	0.94(0.71–1.24)	0.67

OR, odds ratio; CI, confidence intervals; BMI, body mass index.

*Odds ratio for each variable is adjusted for all other variables.

^†^Number of participants, including those with dyslipidemia.

^‡^ *p* values: females vs. male, no vs. yes (current smoking and current drinking, hypertension, diabetes).

^&^ *p* for trend across age groups, educational levels, BMI categories, and waist circumference categories, respectively.

When using China recommended BMI cut-off points, prevalence of low HDL-cholesterol by selected characteristics and adjusted odds ratio (95% CI) of low HDL-cholesterol prevalence including hypertension and diabetes is shown in Table 5. Results of multivariable regression analysis without including hypertension and diabetes showed that low HDL-cholesterol was associated with male gender (Odds Ratio 2.10, 95%CI 1.68–2.62, *p*<0.001), age ≥60 years (OR 0.80, 95%CI 0.64–0.99, *p* = 0.04), current drinking (OR 0.72, 95%CI 0.53–0.99, *p* = 0.04), BMI(OR 1.27, 95%CI 1.04–1.54, *p* = 0.02 for 24–27.9 kg/m^2^, OR 1.56, 95%CI 1.10–2.20, *p* = 0.01 for >28 kg/m^2^), and enlarger waist circumference (OR 1.58, 95%CI 1.29–1.94, *p*<0.001 for 80-89cm/85-94cm; OR 1.76, 95%CI 1.23–2.43, *p*<0.001 for ≥90cm/≥95cm). When analyzing the factors associated with prevalence of low HDL-cholesterol including hypertension and diabetes (p value was not significant for hypertension and diabetes, however, the association with age attenuated.

**Table 5:** Prevalence of low HDL-cholesterol by selected characteristics and adjusted odds ratio (95% CI) of low HDL-cholesterol prevalence including hypertension and diabetes using China recommended BMI cut-off points.

Indicators	Prevalence, †n (%)	*P* value	Adjusted OR (95% CI)	* *P* value	Adjusted OR (95% CI)	* *P* value
Sex		< 0.001‡				
Female	904 (55.4)		1.00(reference)		1.00(reference)	
Male	911 (67.6)		2.10 (1.68–2.62)	<0.001	2.10 (1.68–2.61)	<0.001
Age (years)		0.822&				
18 to 44	864 (61.5)		1.00(reference)		1.00(reference)	
45 to 59	616 (60.5)		0.85 (0.72–1.01)	0.07	0.88(0.73–1.05)	0.15
≥60	335 (60.3)		0.80 (0.64–0.99)	0.04	0.85 (0.67–1.07)	0.16
Education		0.032&				
≤6 years (elementary)	708 (59.9)		1.03 (0.81–1.31)	0.77	1.04 (0.82–1.32)	0.86
7–12 years (secondary)	772 (59.8)		0.82 (0.66–1.03)	0.08	0.82 (0.66–1.03)	0.08
>12 years (high)	335 (66.1)		1.00(reference)		1.00(reference)	
Current smoking		0.001‡				
No	1275 (59.1)		1.00(reference)		1.00(reference)	
Yes	540 (65.5)		0.85 (0.67–1.08)	0.17	0.85 (0.67–1.08)	0.18
Current drinking		0.542‡				
No	1678 (60.8)		1.00(reference)		1.00(reference)	
Yes	137 (62.8)		0.72 (0.53–0.99)	0.04	0.72 (0.53–0.98)	0.04
BMI, kg/m2		<0.001&				
<23.9	901 (55.0)		1.00(reference)		1.00(reference)	
24–27.9	657 (66.2)		1.27 (1.04–1.54)	0.02	1.29(1.06–1.58)	0.01
≥28	257 (73.6)		1.56 (1.10–2.20)	0.01	1.64(1.15–2.33)	0.006
Waist circumference, cm		<0.001&				
Women <80 and men <85	953 (54.8)		1.00(reference)		1.00(reference)	
Women 80–89 and men 85–94	606 (68.1)		1.58(1.29–1.94)	<0.001	1.61 (1.31–1.97)	<0.001
Women≥90 and men≥95	256 (72.9)		1.76 (1.23–2.43)	0.002	1.78 (1.26–2.50)	0.001
Hypertension		0.717‡	-	-		
No	1130(60.7)		-	-	1.00(reference)	
Yes	685(61.3)		-	-	0.86(0.72–1.02)	0.08
Diabetes		0.680‡	-	-		
No	1656(60.8)		-	-	1.00(reference)	
Yes	159(62.1)		-	-	0.92(0.70–1.22)	0.67

OR, odds ratio; CI, confidence intervals; BMI, body mass index.

*Odds ratio for each variable is adjusted for all other variables.

^†^Number of participants, including those with dyslipidemia.

^‡^ *p* values: females vs. male, no vs. yes (current smoking and current drinking, hypertension, diabetes).

^&^ *p* for trend across age groups, educational levels, BMI categories, and waist circumference categories, respectively.

## Discussions

Our results showed that low HDL-cholesterol was the most prevalent type of dyslipidemia in rural northwestern China affecting nearly two in three adults, being higher in males than in females. Low HDL-cholesterol increased with BMI and an enlarged waist circumference. In recent decades, TC level of adults in China has increased rapidly [5, 20, 22]. Based on a 2008 survey in Beijing, 35.4% of adult participants had at least one type of dyslipidemia [22]. Comparing the results of the 2002 China National Nutrition and Health Survey (CNHS 2002) [20, 22] with findings from the 2010 Chinese Non-Communicable Chronic Disease and Risk Factor Surveillance project [23], the mean TC levels increased from 3.81 mmol/L to 4.04 mmol/L whereas the mean LDL-cholesterol levels increased from 2.12 mmol/L to 2.28 mmol/L, and mean serum TG levels have increased from 1.10 mmol/L to 1.33 mmol/L. Our survey found that the mean total and LDL-cholesterol levels of adults living in rural northwest China are lower than the national averages from CNHS 2002 data. However, the mean TG levels were higher in our survey than in 2010 national data [23] and were close to the levels observed in Beijing [22]. These results indicate that HDL-cholesterol levels in the Chinese have decreased from 1.30 mmol/L in 2002 to 1.11 mmol/L in 2010. Most notably, the mean HDL-cholesterol was lower in our study compared to the Chinese Non-Communicable project. Based on national survey, high plasma or serum TC and LDL-cholesterol prevalence were reported to be the major types of dyslipidemia among residents of Beijing, Shanghai, and other large Chinese cities [22–26]. However, the major type of dyslipidemia in this survey was low HDL-cholesterol, a finding supported by previous studies in China [19, 22, 23]. The decrease of HDL-cholesterol in China during the last decade may be associated with an increased prevalence of obesity, especially the abdominal obesity.

Several epidemiological studies have indicated that the prevalence of low HDL-cholesterol and high TG are on the rise in Chinese [20, 24]. Our results showed that less than 40% of the participants had HDL-cholesterol levels in the desirable range. More than 94.4% and 97.0% of participants had ideal serum TC and LDL-cholesterol levels, respectively. A large difference in dyslipidemia types in different regions may be related to difference in economic development, urbanization, dietary pattern changes in transitional periods [20, 23, 24], and possibly genetic susceptibility. Beijing and other urbanized cities in China have experienced rapid economic growth, which could be accompanied by changes in diet and lifestyle such as a higher intake of sodium and fat, reduced fiber intake, and lower physical activity levels [22, 23, 25]. However, most rural regions in China have not experienced such remarkable economic growth, and have maintained more traditional lifestyle and diet.

HDL-cholesterol has an important role in prevention of CHD have be [27, 28]. HDL-cholesterol is a complex of Apo-A lipoproteins that has anti-oxidative, anti-proliferative, antithrombotic, and anti-inflammatory properties [29, 30]. HDL-cholesterol mediates reverse cholesterol transport, which involves the transfer of excess cholesterol from macrophages in peripheral tissues through the blood stream to the liver, where it is metabolized and excreted into the bile [27, 31]. It has been reported that low HDL-cholesterol combined with high TG could dramatically increase the risk of CHD [32]. Low HDL-cholesterol levels as high in our study could result in serious health problems if not controlled. Low HDL-cholesterol levels are probably attributable to the interaction of genetic factors and lifestyle [33]. Previous studies have indicated that family history of low HDL-cholesterol, excessive intake of refined carbohydrates, malnutrition, obesity, male gender, and age could additionally contribute to low HDL-cholesterol [34]. In our study, the risk of low HDL-cholesterol was twice as high in males as in females. But in western societies usually different cut-off values for men [1.03 mmol/L (40 mg/dL)] and women [1.29 mmol/L (50 mg/dL)] are used to define low HDL-cholesterol, which is not the case in China. This obviously may impact on the gender-specific prevalence. Our results also indicate that education and current smoking were related to low HDL-cholesterol. With regard to the factors that predicted low HDL-cholesterol levels in our study, BMI was a less sensitive predictor than waist circumference; however, both of these closely related variables were associated with low HDL-cholesterol. After adjusting for other potential variables, the OR for low HDL-cholesterol among overweight and obese individuals increased. The prevalence of low HDL-cholesterol increased with increasing waist circumference in both males and females and BMI. Central obesity led to a higher OR for low HDL-cholesterol even after adjusting for other potential confounders. Central obesity is an important independent risk factor for low HDL-cholesterol, suggesting that variation in HDL-cholesterol risk may be related to central obesity even when BMI is within normal range which could contribute to synergy and common influence factor of age for low HDL-cholesterol as well as probably for hypertension and diabetes,

We showed that a significant inverse association with OR between low HDL-cholesterol concentrations and alcohol consumption which could be supported by several studies that alcohol consumption could increase HDL-cholesterol level and frequent drinkers were associated with increased levels of HDL-cholesterol [35–37].

A limitation of our study is its cross-sectional design, which cannot establish causality. Another limitation is the subjective nature of individual data in this survey, which could have resulted in misclassification. Since the retrospective survey method was used to fill in the questionnaire, we could not rule out the subject’s error in recalling memory that could result in underestimation or overestimation of the observed effects, which in turn effect the classification. The strength of our study is the sample size and the analytic methods and that our results could directly be compared with other studies with comparable methodology and laboratory.

In summary, our study found that the major type of dyslipidemia found in adults living in rural northwestern China was low HDL-cholesterol. The prevalence of low HDL-cholesterol was related to male gender BMI and central obesity WC), and non-alcohol drinker were associated with covariate-adjusted low HDL-cholesterol levels. This suggest that renewed efforts are needed to lower the prevalence of low HDL-cholesterol. These efforts should include aggressive promotion of a healthy lifestyle, lowering waist circumference and BMI, and blood lipid monitoring in males to prevent low HDL-cholesterol.

## Supporting Information

S1 File HDL cholesterol levels in rural populations. (DOCX)
