# Supplementary material for: The High Prevalence of Low HDL-Cholesterol Levels and Dyslipidemia in Rural Populations in Northwestern China
Source: PLoS One. 2015 Dec 7;10(12):e0144104. doi: 10.1371/journal.pone.0144104 (PMC4671591; doi:10.1371/journal.pone.0144104)
Supplement: S1 File — (DOCX) [file pone.0144104.s001.docx]

**S1 File.** HDL cholesterol levels in rural populations

Pengfei Ge^1^, Caixia Dong^1^, Xiaolan Ren^1^, Elisabete Weiderpass^2,3,4,5^, Chouji Zhang^1^, Haoqiang Fan^1^, Jing Zhang^1^, Yongrui Zhang^1^, Jinen Xi^1^

^1^Gansu Center for Disease Control and Prevention, 230 Donggang Western Road, Chengguan District, 730000, Lanzhou, Gansu Province, China

^2^ Department of Community Medicine, Faculty of Health Sciences, University of Tromsø, Tromsø, Norway；

^3^ Department of Research, Cancer Registry of Norway, Oslo, Norway；

^4^ Department of Medical Epidemiology and Biostatistics, Karolinska Institutet, Stockholm, Sweden；

| ID | CHOL | HDL |  | LDL | TRIG | AGE | SEX | EDUCATION |  | DEINK | SMKOING | BMI-C | BMI-W | WC |
| --- | --- | --- | --- | --- | --- | --- | --- | --- | --- | --- | --- | --- | --- | --- |
| 6204231102 | 4.47 | 1.11 |  | 2.75 | 1.1 | 2 | 1 | 0 |  | 0 | 1 | 1 | 1 | 1 |
| 6204231106 | 3.84 | 1.25 |  | 2.09 | 0.72 | 2 | 1 | 1 |  | 0 | 1 | 1 | 1 | 1 |
| 6204231107 | 4.95 | 1.08 |  | 3.24 | 1.41 | 2 | 1 | 0 |  | 0 | 0 | 2 | 2 | 1 |
| 6204231115 | 2.98 | 0.81 |  | 1.75 | 0.85 | 1 | 1 | 1 |  | 1 | 0 | 3 | 2 | 3 |
| 6204231122 | 3.61 | 1.37 |  | 1.87 | 0.62 | 0 | 0 | 1 |  | 0 | 0 | 2 | 2 | 1 |
| 6204231137 | 5.37 | 1.17 |  | 3.29 | 2.37 | 1 | 0 | 1 |  | 0 | 0 | 3 | 2 | 3 |
| 6204231140 | 4.07 | 0.98 |  | 2.73 | 0.59 | 1 | 1 | 2 |  | 0 | 1 | 2 | 1 | 1 |
| 6204231145 | 3.34 | 0.91 |  | 2.07 | 0.8 | 1 | 0 | 0 |  | 0 | 0 | 1 | 1 | 1 |
| 6204231149 | 3.49 | 1.14 |  | 1.96 | 0.61 | 1 | 0 | 0 |  | 0 | 0 | 2 | 1 | 2 |
| 6204231204 | 4.7 | 1.57 |  | 2.57 | 0.73 | 1 | 0 | 0 |  | 0 | 0 | 2 | 2 | 2 |
| 6204231211 | 2.95 | 0.76 |  | 1.72 | 0.98 | 1 | 0 | 0 |  | 0 | 0 | 2 | 2 | 2 |
| 6204231220 | 4.52 | 0.96 |  | 2.72 | 2.32 | 1 | 0 | 0 |  | 0 | 0 | 3 | 2 | 3 |
| 6204231228 | 3.39 | 0.92 |  | 1.96 | 1.13 | 1 | 0 | 0 |  | 0 | 0 | 1 | 1 | 1 |
| 6204231229 | 4.35 | 0.77 |  | 3.21 | 0.78 | 1 | 1 | 1 |  | 1 | 1 | 3 | 2 | 2 |
| 6204231242 | 3.18 | 1.05 |  | 1.83 | 0.63 | 0 | 0 | 1 |  | 0 | 0 | 2 | 2 | 1 |
| 6204231244 | 3.93 | 0.91 |  | 2.4 | 1.27 | 1 | 0 | 1 |  | 0 | 0 | 3 | 2 | 3 |
| 6204231251 | 3.98 | 1.14 |  | 2.38 | 1.01 | 1 | 0 | 2 |  | 0 | 0 | 2 | 2 | 2 |
| 6204231252 | 4.33 | 1 |  | 2.54 | 1.88 | 1 | 1 | 1 |  | 0 | 1 | 3 | 3 | 3 |
| 6204231304 | 3.61 | 0.85 |  | 2.16 | 1.62 | 1 | 1 | 0 |  | 0 | 1 | 3 | 2 | 2 |
| 6204231307 | 4.24 | 1.26 |  | 2.62 | 0.71 | 1 | 0 | 0 |  | 0 | 0 | 3 | 2 | 3 |
| 6204231309 | 3.57 | 0.97 |  | 1.97 | 1.36 | 0 | 0 | 0 |  | 0 | 0 | 1 | 1 | 1 |
| 6204231315 | 3.89 | 0.98 |  | 2.24 | 1.25 | 1 | 0 | 1 |  | 0 | 0 | 1 | 1 | 1 |
| 6204231319 | 4.17 | 1.1 |  | 2.55 | 0.81 | 1 | 0 | 0 |  | 0 | 0 | 2 | 2 | 2 |
| 6204231323 | 3.78 | 0.91 |  | 2.55 | 0.74 | 1 | 0 | 0 |  | 0 | 0 | 2 | 2 | 1 |
| 6204231333 | 5.2 | 0.69 |  | 4.14 | 0.83 | 1 | 0 | 0 |  | 0 | 0 | 2 | 1 | 2 |
| 6204231337 | 3.68 | 1.19 |  | 2.01 | 0.94 | 1 | 1 | 1 |  | 0 | 1 | 1 | 1 | 1 |
| 6204231344 | 3.05 | 1.06 |  | 1.61 | 0.52 | 1 | 1 | 1 |  | 0 | 0 | 1 | 1 | 1 |
| 6204231345 | 2.8 | 0.82 |  | 1.37 | 1.63 | 1 | 1 | 1 |  | 0 | 1 | 1 | 1 | 1 |
| 6204231351 | 3.83 | 1.28 |  | 2.28 | 0.62 | 1 | 0 | 1 |  | 0 | 0 | 1 | 1 | 1 |
| 6204231352 | 4.89 | 0.97 |  | 3.65 | 0.89 | 1 | 1 | 1 |  | 0 | 1 | 3 | 3 | 3 |
| 6204232110 | 3.12 | 0.91 |  | 1.21 | 1.61 | 0 | 1 | 1 |  | 0 | 1 | 2 | 1 | 1 |
| 6204232111 | 3.51 | 1.07 |  | 2.28 | 0.52 | 1 | 0 | 1 |  | 0 | 0 | 2 | 2 | 2 |
| 6204232114 | 6.97 | 1.15 |  | 4.26 | 3.63 | 2 | 0 | 0 |  | 0 | 0 | 1 | 1 | 2 |
| 6204232115 | 4.51 | 0.96 |  | 3.23 | 1.03 | 0 | 0 | 2 |  | 0 | 0 | 2 | 1 | 1 |
| 6204232122 | 4.31 | 1.19 |  | 2.66 | 0.9 | 2 | 0 | 1 |  | 0 | 0 | 2 | 2 | 3 |
| 6204232128 | 4.17 | 1.28 |  | 1.28 | 2.13 | 2 | 0 | 0 |  | 0 | 0 | 1 | 1 | 1 |
| 6204232134 | 5.39 | 1.09 |  | 3.54 | 2.01 | 1 | 0 | 0 |  | 0 | 0 | 2 | 2 | 2 |
| 6204232137 | 5.92 | 1.18 |  | 3.31 | 2.93 | 1 | 0 | 0 |  | 0 | 0 | 3 | 2 | 2 |
| 6204232145 | 4.2 | 1.08 |  | 2.53 | 1.51 | 0 | 0 | 1 |  | 0 | 0 | 3 | 3 | 3 |
| 6204232146 | 5.27 | 1.24 |  | 3.35 | 1.41 | 1 | 0 | 0 |  | 0 | 0 | 3 | 2 | 3 |
| 6204232148 | 3.79 | 1.22 |  | 1.83 | 1.42 | 2 | 0 | 1 |  | 0 | 0 | 2 | 2 | 2 |
| 6204232202 | 3.98 | 0.73 |  | 2.45 | 2.46 | 1 | 1 | 1 |  | 0 | 1 | 2 | 2 | 2 |
| 6204232204 | 3.72 | 1.11 |  | 2.17 | 0.94 | 0 | 0 | 1 |  | 0 | 0 | 1 | 1 | 1 |
| 6204232207 | 5.63 | 1.19 |  | 3.77 | 1.58 | 2 | 1 | 0 |  | 0 | 0 | 1 | 1 | 1 |
| 6204232209 | 5.66 | 1 |  | 4.25 | 0.95 | 1 | 1 | 1 |  | 0 | 0 | 2 | 2 | 2 |
| 6204232212 | 4.45 | 1.02 |  | 2.72 | 0.86 | 1 | 0 | 0 |  | 0 | 0 | 1 | 1 | 2 |
| 6204232214 | 5.43 | 1.43 |  | 3.17 | 1.48 | 1 | 0 | 1 |  | 0 | 0 | 3 | 2 | 2 |
| 6204232222 | 3.32 | 1.41 |  | 1.08 | 1.25 | 0 | 0 | 1 |  | 0 | 0 | 3 | 2 | 1 |
| 6204232224 | 3.32 | 0.89 |  | 1.64 | 1.55 | 1 | 0 | 0 |  | 0 | 0 | 3 | 2 | 2 |
| 6204232225 | 3.94 | 1.02 |  | 2.52 | 1.12 | 1 | 1 | 0 |  | 0 | 1 | 2 | 2 | 3 |
| 6204232228 | 5.23 | 1.45 |  | 3.2 | 1.18 | 2 | 0 | 0 |  | 0 | 0 | 2 | 2 | 2 |
| 6204232232 | 2.92 | 0.77 |  | 1.67 | 1.32 | 0 | 1 | 2 |  | 0 | 0 | 2 | 2 | 2 |
| 6204232235 | 4.17 | 0.84 |  | 2.05 | 3.06 | 2 | 0 | 0 |  | 0 | 0 | 1 | 1 | 1 |
| 6204232239 | 3.14 | 1.13 |  | 1.42 | 0.89 | 1 | 1 | 0 |  | 0 | 0 | 1 | 1 | 1 |
| 6204232240 | 6.69 | 1.33 |  | 4.66 | 1.25 | 2 | 1 | 1 |  | 0 | 1 | 1 | 1 | 1 |
| 6204232241 | 5.03 | 2.54 |  | 0.96 | 2.46 | 0 | 1 | 1 |  | 0 | 0 | 1 | 1 | 1 |
| 6204232243 | 4.34 | 0.98 |  | 2.48 | 2.03 | 1 | 1 | 1 |  | 0 | 0 | 2 | 2 | 2 |
| 6204232246 | 5.06 | 1.34 |  | 3.06 | 1.1 | 1 | 0 | 0 |  | 0 | 0 | 2 | 2 | 2 |
| 6204232305 | 2.02 | 0.63 |  | 0.96 | 0.88 | 1 | 1 | 1 |  | 0 | 1 | 2 | 1 | 1 |
| 6204232314 | 4.31 | 0.96 |  | 2.76 | 1.31 | 2 | 1 | 0 |  | 0 | 1 | 1 | 1 | 1 |
| 6204232315 | 5.42 | 1.03 |  | 3.69 | 1.83 | 2 | 1 | 1 |  | 0 | 1 | 1 | 1 | 1 |
| 6204232319 | 3.73 | 1.48 |  | 1.6 | 0.9 | 1 | 0 | 0 |  | 0 | 0 | 3 | 3 | 2 |
| 6204232320 | 4.42 | 1.14 |  | 2.42 | 1.89 | 1 | 0 | 1 |  | 0 | 0 | 2 | 2 | 2 |
| 6204232323 | 3.54 | 0.71 |  | 2 | 2 | 1 | 1 | 1 |  | 0 | 0 | 2 | 2 | 3 |
| 6204232327 | 4.05 | 1.05 |  | 2.03 | 3.07 | 1 | 0 | 2 |  | 0 | 0 | 1 | 1 | 1 |
| 6204232328 | 6.94 | 1.12 |  | 4.44 | 3.48 | 1 | 0 | 0 |  | 0 | 0 | 3 | 3 | 1 |
| 6204232332 | 5.24 | 1.66 |  | 3 | 1.15 | 0 | 0 | 0 |  | 0 | 0 | 1 | 1 | 1 |
| 6204232337 | 4.7 | 1.02 |  | 3.07 | 1.76 | 0 | 1 | 1 |  | 0 | 0 | 3 | 2 | 2 |
| 6204232340 | 4.44 | 1.38 |  | 2.45 | 1.09 | 2 | 1 | 0 |  | 0 | 0 | 1 | 1 | 1 |
| 6204232341 | 5.12 | 1.33 |  | 2.94 | 1.23 | 1 | 0 | 0 |  | 0 | 0 | 1 | 1 | 1 |
| 6204232343 | 4.18 | 1.24 |  | 2.45 | 0.86 | 1 | 0 | 1 |  | 0 | 0 | 3 | 2 | 2 |
| 6204232345 | 4.64 | 0.94 |  | 3.15 | 1.15 | 2 | 1 | 0 |  | 0 | 0 | 1 | 1 | 1 |
| 6204232346 | 3.44 | 1.16 |  | 1.87 | 0.77 | 1 | 1 | 2 |  | 0 | 0 | 1 | 1 | 1 |
| 6204232350 | 4.64 | 0.77 |  | 2.53 | 2.25 | 2 | 0 | 0 |  | 0 | 0 | 2 | 2 | 3 |
| 6204233105 | 4.08 | 1.14 |  | 2.06 | 1.62 | 1 | 0 | 1 |  | 0 | 0 | 3 | 2 | 2 |
| 6204233110 | 5 | 1.48 |  | 2.94 | 1.45 | 1 | 0 | 1 |  | 0 | 0 | 2 | 2 | 2 |
| 6204233112 | 4.4 | 1.06 |  | 2.69 | 2.3 | 1 | 1 | 2 |  | 0 | 0 | 2 | 2 | 2 |
| 6204233114 | 4.42 | 1.02 |  | 2.85 | 1.19 | 2 | 1 | 1 |  | 0 | 1 | 1 | 1 | 1 |
| 6204233115 | 3.64 | 1.01 |  | 2.29 | 1.09 | 2 | 0 | 1 |  | 0 | 0 | 2 | 2 | 2 |
| 6204233116 | 5.35 | 1.41 |  | 2.97 | 2.01 | 2 | 0 | 0 |  | 0 | 0 | 3 | 3 | 3 |
| 6204233118 | 3.08 | 1.07 |  | 1.66 | 0.8 | 2 | 0 | 0 |  | 0 | 0 | 3 | 2 | 3 |
| 6204233120 | 5.11 | 0.92 |  | 3.68 | 1.48 | 0 | 1 | 2 |  | 0 | 0 | 1 | 1 | 2 |
| 6204233122 | 4.8 | 0.85 |  | 2.72 | 3.31 | 0 | 1 | 2 |  | 0 | 1 | 3 | 3 | 2 |
| 6204233125 | 5.44 | 0.95 |  | 3.84 | 1.2 | 2 | 0 | 0 |  | 0 | 0 | 2 | 1 | 2 |
| 6204233127 | 4.03 | 0.82 |  | 2.85 | 1.15 | 0 | 0 | 1 |  | 0 | 0 | 3 | 3 | 3 |
| 6204233129 | 5.41 | 0.82 |  | 4.11 | 1.56 | 1 | 1 | 2 |  | 0 | 0 | 3 | 3 | 3 |
| 6204233134 | 4.39 | 0.94 |  | 2.85 | 1.15 | 1 | 0 | 0 |  | 0 | 0 | 3 | 2 | 2 |
| 6204233137 | 3.72 | 1 |  | 2.18 | 1.35 | 1 | 1 | 2 |  | 0 | 1 | 2 | 2 | 1 |
| 6204233142 | 5.44 | 1.02 |  | 2.81 | 3.75 | 1 | 0 | 2 |  | 0 | 0 | 3 | 2 | 3 |
| 6204233143 | 3.11 | 0.93 |  | 1.37 | 2.52 | 0 | 1 | 1 |  | 1 | 1 | 2 | 1 | 1 |
| 6204233144 | 4.05 | 0.95 |  | 2.83 | 1.08 | 2 | 1 | 1 |  | 0 | 1 | 1 | 1 | 1 |
| 6204233146 | 4.28 | 0.8 |  | 2.58 | 2.85 | 2 | 1 | 2 |  | 0 | 1 | 2 | 2 | 3 |
| 6204233147 | 4.98 | 1.25 |  | 3.01 | 1.73 | 1 | 0 | 2 |  | 0 | 0 | 3 | 3 | 3 |
| 6204233149 | 4.23 | 1.06 |  | 2.72 | 1.61 | 1 | 0 | 1 |  | 0 | 0 | 3 | 2 | 3 |
| 6204233151 | 5.81 | 1.16 |  | 3.23 | 3.44 | 2 | 0 | 0 |  | 0 | 0 | 2 | 2 | 3 |
| 6204233153 | 4.47 | 1.18 |  | 2.27 | 2.27 | 2 | 1 | 0 |  | 0 | 1 | 3 | 2 | 2 |
| 6204233202 | 3.42 | 0.79 |  | 1.74 | 2.07 | 1 | 1 | 2 |  | 0 | 0 | 3 | 3 | 3 |
| 6204233203 | 5.68 | 0.88 |  | 3.01 | 4.08 | 2 | 0 | 0 |  | 0 | 0 | 2 | 2 | 2 |
| 6204233204 | 3.58 | 0.88 |  | 2.38 | 1 | 1 | 0 | 0 |  | 0 | 0 | 1 | 1 | 2 |
| 6204233206 | 4.82 | 1.19 |  | 2.79 | 1.47 | 1 | 0 | 0 |  | 0 | 0 | 1 | 1 | 1 |
| 6204233207 | 4.96 | 1.11 |  | 3.27 | 1.33 | 1 | 0 | 0 |  | 0 | 0 | 2 | 1 | 1 |
| 6204233209 | 4.11 | 1.16 |  | 2.56 | 1.22 | 0 | 1 | 2 |  | 0 | 1 | 2 | 2 | 2 |
| 6204233216 | 3.67 | 1.17 |  | 2.04 | 0.93 | 0 | 0 | 0 |  | 0 | 0 | 1 | 1 | 1 |
| 6204233222 | 4.14 | 1.01 |  | 2.58 | 1.73 | 1 | 0 | 0 |  | 0 | 0 | 3 | 3 | 3 |
| 6204233226 | 2.97 | 1.07 |  | 1.35 | 1.28 | 0 | 1 | 2 |  | 1 | 1 | 1 | 1 | 1 |
| 6204233229 | 5 | 1.11 |  | 3.03 | 2.43 | 1 | 0 | 0 |  | 0 | 0 | 3 | 3 | 3 |
| 6204233232 | 4.92 | 0.86 |  | 3.44 | 1.38 | 2 | 0 | 1 |  | 0 | 0 | 3 | 3 | 3 |
| 6204233233 | 4.17 | 1.7 |  | 1.32 | 1.68 | 1 | 1 | 2 |  | 0 | 1 | 2 | 2 | 2 |
| 6204233235 | 5.59 | 1.09 |  | 3.16 | 4.62 | 1 | 1 | 1 |  | 0 | 0 | 2 | 2 | 3 |
| 6204233237 | 5.24 | 1.67 |  | 2.92 | 1.01 | 1 | 0 | 2 |  | 0 | 0 | 1 | 1 | 1 |
| 6204233242 | 3.78 | 0.95 |  | 2.21 | 2.38 | 0 | 1 | 2 |  | 1 | 0 | 3 | 2 | 2 |
| 6204233302 | 3.82 | 0.88 |  | 2.36 | 1.42 | 2 | 1 | 1 |  | 0 | 0 | 2 | 2 | 2 |
| 6204233310 | 5.18 | 1.08 |  | 3.23 | 2.73 | 1 | 0 | 0 |  | 0 | 0 | 2 | 2 | 2 |
| 6204233311 | 3.01 | 1.1 |  | 1.56 | 0.7 | 0 | 0 | 0 |  | 0 | 0 | 3 | 2 | 3 |
| 6204233315 | 2.37 | 0.91 |  | 1.06 | 1.22 | 1 | 1 | 1 |  | 0 | 1 | 2 | 2 | 1 |
| 6204233317 | 3.93 | 1.06 |  | 2.26 | 1.27 | 1 | 0 | 1 |  | 0 | 0 | 2 | 2 | 2 |
| 6204233322 | 4.17 | 1.01 |  | 2.55 | 1.05 | 1 | 0 | 2 |  | 0 | 0 | 3 | 2 | 2 |
| 6204233328 | 3.32 | 1.17 |  | 1.72 | 0.73 | 1 | 1 | 2 |  | 0 | 0 | 1 | 1 | 1 |
| 6204233329 | 3.01 | 1.12 |  | 1.49 | 0.71 | 0 | 0 | 1 |  | 0 | 0 | 1 | 1 | 2 |
| 6204233331 | 3.87 | 0.69 |  | 1.95 | 2.21 | 1 | 1 | 1 |  | 0 | 1 | 2 | 2 | 2 |
| 6204233334 | 5.17 | 1.13 |  | 2.99 | 2.47 | 2 | 1 | 1 |  | 0 | 1 | 2 | 2 | 2 |
| 6204233338 | 4.67 | 1.18 |  | 2.69 | 2.17 | 1 | 0 | 0 |  | 0 | 0 | 2 | 1 | 1 |
| 6204233339 | 4.18 | 1.08 |  | 2.54 | 0.78 | 1 | 1 | 1 |  | 0 | 1 | 1 | 1 | 1 |
| 6204233341 | 4.47 | 1.72 |  | 2.1 | 1.04 | 2 | 1 | 1 |  | 1 | 1 | 1 | 1 | 1 |
| 6204233347 | 4.86 | 1.72 |  | 2.48 | 1.23 | 1 | 0 | 1 |  | 0 | 0 | 2 | 1 | 1 |
| 6204234101 | 3.02 | 1.54 |  | 1.17 | 0.46 | 2 | 0 | 0 |  | 0 | 0 | 1 | 1 | 1 |
| 6204234108 | 4.88 | 0.99 |  | 3.05 | 1.97 | 1 | 1 | 1 |  | 0 | 1 | 1 | 1 | 1 |
| 6204234112 | 3.73 | 1.08 |  | 1.91 | 1.64 | 2 | 0 | 0 |  | 0 | 0 | 1 | 1 | 1 |
| 6204234121 | 4.33 | 1.18 |  | 2.55 | 2 | 1 | 1 | 1 |  | 0 | 0 | 3 | 3 | 2 |
| 6204234123 | 4.41 | 0.81 |  | 2.98 | 1.65 | 2 | 1 | 1 |  | 0 | 0 | 2 | 1 | 2 |
| 6204234124 | 3.55 | 0.69 |  | 2.12 | 2.91 | 0 | 0 | 1 |  | 0 | 0 | 3 | 2 | 3 |
| 6204234133 | 4.31 | 1.08 |  | 2.68 | 0.92 | 1 | 0 | 1 |  | 0 | 0 | 1 | 1 | 1 |
| 6204234135 | 7.6 | 1.47 |  | 4.66 | 2.65 | 2 | 0 | 0 |  | 0 | 0 | 2 | 2 | 2 |
| 6204234139 | 5.16 | 1.45 |  | 3.05 | 1.51 | 0 | 1 | 0 |  | 0 | 1 | 1 | 1 | 1 |
| 6204234144 | 3.83 | 0.97 |  | 2.21 | 1.15 | 1 | 0 | 0 |  | 0 | 0 | 1 | 1 | 1 |
| 6204234202 | 4.09 | 0.97 |  | 2.82 | 0.84 | 1 | 0 | 1 |  | 0 | 0 | 3 | 2 | 2 |
| 6204234203 | 4.36 | 0.85 |  | 2.61 | 2.49 | 1 | 1 | 1 |  | 1 | 1 | 3 | 2 | 2 |
| 6204234207 | 4.39 | 1.22 |  | 2.69 | 0.69 | 1 | 0 | 0 |  | 0 | 0 | 1 | 1 | 1 |
| 6204234217 | 4.25 | 0.92 |  | 2.69 | 1.92 | 1 | 0 | 1 |  | 0 | 0 | 3 | 2 | 2 |
| 6204234218 | 5.56 | 1.72 |  | 3.1 | 0.88 | 2 | 0 | 1 |  | 0 | 0 | 1 | 1 | 1 |
| 6204234219 | 5.14 | 1.32 |  | 3.01 | 3.18 | 1 | 1 | 1 |  | 1 | 1 | 3 | 3 | 3 |
| 6204234220 | 3.05 | 0.8 |  | 1.63 | 1.44 | 0 | 1 | 1 |  | 0 | 0 | 2 | 2 | 2 |
| 6204234230 | 3.03 | 0.81 |  | 1.62 | 1.51 | 2 | 0 | 0 |  | 0 | 0 | 3 | 2 | 3 |
| 6204234231 | 3.94 | 1.23 |  | 1.8 | 1.48 | 2 | 0 | 0 |  | 0 | 0 | 2 | 1 | 1 |
| 6204234233 | 5.76 | 1.18 |  | 3.68 | 1.88 | 1 | 0 | 0 |  | 0 | 0 | 3 | 3 | 3 |
| 6204234236 | 6.08 | 1.77 |  | 3.22 | 2.22 | 2 | 1 | 0 |  | 0 | 1 | 2 | 1 | 1 |
| 6204234247 | 3.69 | 1.06 |  | 2.22 | 1.27 | 0 | 1 | 1 |  | 1 | 1 | 2 | 2 | 2 |
| 6204234253 | 4.35 | 1.14 |  | 2.58 | 1.13 | 1 | 0 | 1 |  | 0 | 0 | 2 | 2 | 2 |
| 6204234254 | 4.64 | 1.3 |  | 2.41 | 1.95 | 2 | 0 | 0 |  | 0 | 0 | 2 | 2 | 2 |
| 6204234302 | 3.59 | 0.82 |  | 2.19 | 1.12 | 2 | 1 | 1 |  | 0 | 1 | 2 | 2 | 2 |
| 6204234303 | 4.06 | 1.1 |  | 2.18 | 1.11 | 2 | 0 | 0 |  | 0 | 0 | 2 | 2 | 2 |
| 6204234314 | 5.17 | 1.09 |  | 3.29 | 2.34 | 2 | 0 | 0 |  | 0 | 0 | 3 | 2 | 2 |
| 6204234317 | 3.12 | 1.11 |  | 1.37 | 0.89 | 0 | 1 | 0 |  | 0 | 1 | 2 | 1 | 2 |
| 6204234318 | 4.01 | 1.11 |  | 2.23 | 1.1 | 1 | 0 | 0 |  | 0 | 0 | 2 | 1 | 2 |
| 6204234321 | 3.37 | 1.29 |  | 1.43 | 0.91 | 2 | 0 | 0 |  | 0 | 0 | 1 | 1 | 1 |
| 6204234325 | 4.32 | 1.25 |  | 2.36 | 1.22 | 2 | 1 | 0 |  | 0 | 1 | 1 | 1 | 1 |
| 6204234326 | 3.24 | 1.4 |  | 1.34 | 0.77 | 0 | 1 | 1 |  | 1 | 1 | 2 | 2 | 3 |
| 6204234327 | 5.69 | 1.11 |  | 3.71 | 2.08 | 2 | 1 | 0 |  | 0 | 1 | 2 | 2 | 2 |
| 6204234332 | 3.54 | 0.91 |  | 2.42 | 0.64 | 1 | 1 | 0 |  | 0 | 1 | 2 | 2 | 2 |
| 6204234337 | 4.58 | 1.52 |  | 2.4 | 0.65 | 1 | 0 | 0 |  | 0 | 0 | 1 | 1 | 1 |
| 6204234338 | 3 | 0.93 |  | 1.38 | 0.96 | 2 | 0 | 0 |  | 0 | 1 | 1 | 1 | 1 |
| 6204234341 | 4.31 | 0.8 |  | 2.77 | 1.55 | 1 | 0 | 0 |  | 0 | 0 | 1 | 1 | 2 |
| 6204234345 | 4.26 | 1.51 |  | 2.08 | 0.68 | 2 | 1 | 1 |  | 0 | 1 | 1 | 1 | 1 |
| 6204234350 | 4.85 | 1.23 |  | 2.7 | 2.57 | 0 | 0 | 1 |  | 0 | 0 | 3 | 3 | 3 |
| 6204234351 | 4.45 | 1.2 |  | 2.75 | 1.34 | 1 | 1 | 1 |  | 1 | 1 | 2 | 1 | 2 |
| 6204234352 | 4.85 | 1.26 |  | 2.55 | 1.39 | 1 | 0 | 0 |  | 0 | 0 | 2 | 2 | 1 |
| 6205031104 | 3.16 | 0.91 |  | 1.76 | 0.82 | 2 | 0 | 0 |  | 0 | 0 | 2 | 2 | 3 |
| 6205031105 | 3.39 | 0.9 |  | 1.93 | 1.82 | 2 | 0 | 0 |  | 0 | 0 | 3 | 2 | 3 |
| 6205031107 | 3.63 | 1.2 |  | 2 | 0.61 | 1 | 1 | 1 |  | 0 | 1 | 2 | 2 | 1 |
| 6205031108 | 3.9 | 0.87 |  | 2.3 | 1.74 | 1 | 1 | 1 |  | 0 | 1 | 3 | 3 | 3 |
| 6205031110 | 3.42 | 0.9 |  | 2.17 | 0.93 | 2 | 0 | 0 |  | 0 | 0 | 1 | 1 | 2 |
| 6205031112 | 4.42 | 1.59 |  | 2.31 | 1.23 | 2 | 0 | 1 |  | 0 | 0 | 1 | 1 | 1 |
| 6205031114 | 4.74 | 1.42 |  | 2.92 | 0.75 | 1 | 0 | 0 |  | 0 | 0 | 1 | 1 | 2 |
| 6205031117 | 4.14 | 1.22 |  | 2.16 | 1.17 | 2 | 0 | 0 |  | 0 | 0 | 1 | 1 | 1 |
| 6205031119 | 3.48 | 1.01 |  | 2.02 | 0.84 | 1 | 0 | 1 |  | 0 | 0 | 1 | 1 | 1 |
| 6205031123 | 3.91 | 0.74 |  | 2.56 | 1.45 | 1 | 1 | 1 |  | 0 | 0 | 2 | 1 | 2 |
| 6205031124 | 3 | 0.63 |  | 1.9 | 0.93 | 2 | 1 | 1 |  | 0 | 1 | 2 | 2 | 2 |
| 6205031125 | 3.65 | 0.85 |  | 2.25 | 1.1 | 1 | 1 | 0 |  | 0 | 0 | 3 | 2 | 3 |
| 6205031128 | 4.13 | 1.68 |  | 2.48 | 0.74 | 1 | 0 | 1 |  | 0 | 0 | 1 | 1 | 1 |
| 6205031131 | 2.45 | 0.73 |  | 1.01 | 1.55 | 2 | 1 | 1 |  | 0 | 1 | 1 | 1 | 1 |
| 6205031133 | 4.14 | 1.17 |  | 2.33 | 1.64 | 2 | 1 | 1 |  | 0 | 1 | 1 | 1 | 1 |
| 6205031135 | 4.23 | 1.57 |  | 2.21 | 0.85 | 1 | 0 | 1 |  | 0 | 0 | 1 | 1 | 1 |
| 6205031137 | 5.29 | 1.15 |  | 3.07 | 3.16 | 1 | 0 | 0 |  | 0 | 0 | 3 | 2 | 3 |
| 6205031139 | 3.87 | 0.86 |  | 2.57 | 1.65 | 2 | 1 | 1 |  | 0 | 1 | 2 | 2 | 2 |
| 6205031140 | 3.8 | 1.41 |  | 1.58 | 1.55 | 1 | 1 | 2 |  | 0 | 0 | 1 | 1 | 1 |
| 6205031141 | 4.56 | 1 |  | 3.04 | 1.04 | 1 | 1 | 1 |  | 0 | 1 | 2 | 2 | 2 |
| 6205031144 | 3 | 0.84 |  | 1.7 | 0.85 | 2 | 0 | 0 |  | 0 | 0 | 1 | 1 | 1 |
| 6205031146 | 4.5 | 1.1 |  | 2.72 | 1.24 | 1 | 0 | 0 |  | 0 | 0 | 1 | 1 | 1 |
| 6205031147 | 3.42 | 0.96 |  | 2.01 | 0.83 | 1 | 1 | 1 |  | 0 | 1 | 2 | 2 | 2 |
| 6205031148 | 3.9 | 1.08 |  | 2.34 | 1.32 | 1 | 1 | 1 |  | 0 | 1 | 3 | 2 | 2 |
| 6205031202 | 3.91 | 0.7 |  | 1.79 | 2.52 | 1 | 0 | 0 |  | 0 | 0 | 1 | 1 | 2 |
| 6205031206 | 3.82 | 0.85 |  | 2.26 | 1.86 | 2 | 1 | 2 |  | 0 | 0 | 2 | 2 | 2 |
| 6205031209 | 4.08 | 1.1 |  | 2.42 | 0.96 | 2 | 0 | 0 |  | 0 | 0 | 1 | 1 | 2 |
| 6205031210 | 2.91 | 0.87 |  | 1.22 | 1.2 | 2 | 0 | 1 |  | 0 | 0 | 2 | 2 | 1 |
| 6205031213 | 3.35 | 0.99 |  | 1.82 | 1.02 | 2 | 1 | 1 |  | 0 | 1 | 2 | 2 | 1 |
| 6205031218 | 3.84 | 1.35 |  | 1.9 | 1.2 | 1 | 0 | 0 |  | 0 | 0 | 2 | 1 | 1 |
| 6205031223 | 3.13 | 0.72 |  | 1.46 | 2.17 | 2 | 1 | 1 |  | 0 | 1 | 1 | 1 | 1 |
| 6205031226 | 4.22 | 0.87 |  | 2.71 | 1.34 | 1 | 0 | 0 |  | 0 | 0 | 2 | 2 | 3 |
| 6205031227 | 3.09 | 0.71 |  | 1.47 | 2.65 | 1 | 0 | 1 |  | 0 | 0 | 2 | 1 | 2 |
| 6205031234 | 4.13 | 1.08 |  | 2.46 | 1.07 | 2 | 0 | 1 |  | 0 | 0 | 2 | 1 | 2 |
| 6205031235 | 4.29 | 0.97 |  | 2.46 | 2.02 | 1 | 0 | 0 |  | 0 | 0 | 1 | 1 | 1 |
| 6205031236 | 3.26 | 0.97 |  | 1.6 | 1.44 | 0 | 0 | 2 |  | 0 | 0 | 1 | 1 | 1 |
| 6205031237 | 3.45 | 0.85 |  | 2 | 1.2 | 2 | 1 | 1 |  | 0 | 0 | 2 | 1 | 1 |
| 6205031240 | 3.14 | 1.1 |  | 1.29 | 1.55 | 0 | 1 | 1 |  | 0 | 0 | 1 | 1 | 2 |
| 6205031241 | 4.7 | 1.02 |  | 2.33 | 2.84 | 2 | 0 | 0 |  | 0 | 0 | 1 | 1 | 1 |
| 6205031242 | 4.86 | 1.05 |  | 2.78 | 2.78 | 1 | 0 | 1 |  | 0 | 0 | 3 | 2 | 2 |
| 6205031244 | 4.17 | 1.09 |  | 2.37 | 1.22 | 0 | 0 | 1 |  | 0 | 0 | 1 | 1 | 1 |
| 6205031246 | 3.6 | 0.79 |  | 2.14 | 1.63 | 2 | 1 | 1 |  | 0 | 1 | 2 | 1 | 2 |
| 6205031248 | 4.6 | 1.12 |  | 2.55 | 2.7 | 1 | 0 | 0 |  | 0 | 0 | 2 | 2 | 1 |
| 6205031249 | 3.74 | 0.88 |  | 2.08 | 2.51 | 1 | 0 | 2 |  | 0 | 0 | 1 | 1 | 1 |
| 6205031250 | 4.65 | 1.17 |  | 2.82 | 1.29 | 1 | 0 | 1 |  | 0 | 0 | 3 | 2 | 2 |
| 6205031301 | 3.02 | 1 |  | 1.33 | 1.56 | 2 | 0 | 1 |  | 0 | 0 | 2 | 2 | 2 |
| 6205031302 | 3.42 | 1.16 |  | 1.63 | 1.07 | 2 | 0 | 1 |  | 0 | 0 | 1 | 1 | 3 |
| 6205031306 | 3.93 | 0.66 |  | 2.61 | 1.9 | 2 | 1 | 1 |  | 0 | 1 | 3 | 2 | 3 |
| 6205031307 | 3.78 | 0.82 |  | 2.25 | 1.36 | 1 | 0 | 2 |  | 0 | 0 | 3 | 2 | 2 |
| 6205031308 | 2.78 | 0.91 |  | 1.46 | 1.34 | 0 | 0 | 1 |  | 0 | 0 | 1 | 1 | 2 |
| 6205031309 | 4.59 | 1.12 |  | 2.88 | 1.53 | 2 | 0 | 0 |  | 0 | 0 | 3 | 3 | 3 |
| 6205031310 | 4.15 | 0.98 |  | 2.57 | 1.7 | 2 | 1 | 1 |  | 0 | 0 | 2 | 2 | 1 |
| 6205031311 | 5.39 | 1.02 |  | 3.73 | 1.66 | 2 | 1 | 1 |  | 0 | 0 | 2 | 1 | 3 |
| 6205031315 | 2.73 | 0.79 |  | 1.36 | 1.21 | 1 | 0 | 2 |  | 0 | 0 | 1 | 1 | 1 |
| 6205031318 | 3.46 | 0.9 |  | 1.75 | 1.34 | 2 | 0 | 1 |  | 0 | 0 | 2 | 2 | 1 |
| 6205031319 | 3.94 | 1.12 |  | 1.98 | 1.3 | 2 | 0 | 1 |  | 0 | 0 | 1 | 1 | 1 |
| 6205031320 | 4.64 | 1.06 |  | 2.68 | 1.9 | 2 | 1 | 1 |  | 0 | 0 | 2 | 2 | 2 |
| 6205031325 | 2.95 | 0.94 |  | 1.54 | 0.71 | 1 | 0 | 2 |  | 0 | 0 | 1 | 1 | 2 |
| 6205031327 | 3.4 | 0.62 |  | 2.27 | 1.11 | 2 | 1 | 1 |  | 0 | 1 | 1 | 1 | 1 |
| 6205031328 | 4.78 | 1.51 |  | 2.59 | 1.06 | 2 | 1 | 2 |  | 0 | 0 | 1 | 1 | 1 |
| 6205031329 | 3.62 | 1.2 |  | 1.84 | 1.27 | 2 | 1 | 0 |  | 1 | 0 | 1 | 1 | 2 |
| 6205031330 | 3.61 | 1.27 |  | 1.75 | 0.72 | 2 | 1 | 1 |  | 0 | 1 | 2 | 1 | 1 |
| 6205031331 | 4.92 | 0.9 |  | 3.23 | 1.5 | 1 | 0 | 2 |  | 0 | 0 | 3 | 3 | 3 |
| 6205031334 | 3.42 | 0.74 |  | 2.08 | 1.19 | 2 | 0 | 1 |  | 0 | 0 | 2 | 2 | 2 |
| 6205031337 | 3.74 | 0.84 |  | 2.3 | 1.17 | 1 | 1 | 1 |  | 0 | 1 | 3 | 2 | 2 |
| 6205031338 | 3.8 | 0.81 |  | 2.16 | 2.3 | 1 | 1 | 2 |  | 1 | 1 | 2 | 1 | 2 |
| 6205031339 | 5.56 | 1.38 |  | 3.3 | 1.92 | 1 | 0 | 1 |  | 0 | 0 | 3 | 3 | 3 |
| 6205031344 | 4.13 | 0.79 |  | 2.7 | 2 | 1 | 1 | 2 |  | 0 | 1 | 1 | 1 | 2 |
| 6205031347 | 3.76 | 1.02 |  | 2.02 | 1.4 | 0 | 0 | 1 |  | 0 | 0 | 2 | 2 | 2 |
| 6205031349 | 3.81 | 1.15 |  | 2.09 | 0.99 | 0 | 0 | 1 |  | 0 | 0 | 1 | 1 | 2 |
| 6205032101 | 3.75 | 1.62 |  | 1.46 | 1.04 | 1 | 1 | 1 |  | 0 | 1 | 2 | 2 | 1 |
| 6205032104 | 2.84 | 0.8 |  | 1.34 | 1.28 | 2 | 1 | 0 |  | 0 | 1 | 3 | 3 | 3 |
| 6205032109 | 4.41 | 0.74 |  | 1.81 | 3.8 | 1 | 1 | 2 |  | 0 | 0 | 1 | 1 | 1 |
| 6205032110 | 3.51 | 0.81 |  | 1.7 | 1.39 | 2 | 1 | 0 |  | 0 | 1 | 1 | 1 | 1 |
| 6205032113 | 3.76 | 1.04 |  | 1.96 | 1.68 | 1 | 1 | 0 |  | 0 | 1 | 1 | 1 | 1 |
| 6205032114 | 4.13 | 0.98 |  | 2.37 | 1.32 | 0 | 1 | 2 |  | 0 | 0 | 2 | 2 | 2 |
| 6205032115 | 5.08 | 1.13 |  | 2.57 | 2.63 | 2 | 0 | 1 |  | 0 | 0 | 3 | 2 | 3 |
| 6205032119 | 3.08 | 0.89 |  | 1.68 | 1.05 | 1 | 1 | 0 |  | 0 | 1 | 2 | 2 | 1 |
| 6205032120 | 4.08 | 0.89 |  | 2.43 | 1.22 | 2 | 1 | 2 |  | 0 | 1 | 2 | 2 | 2 |
| 6205032125 | 4.15 | 1.39 |  | 1.68 | 1.26 | 2 | 1 | 1 |  | 0 | 1 | 1 | 1 | 1 |
| 6205032128 | 3.5 | 0.82 |  | 1.52 | 2.89 | 1 | 1 | 1 |  | 0 | 0 | 3 | 2 | 2 |
| 6205032134 | 3.61 | 1.05 |  | 1.89 | 1.17 | 0 | 1 | 1 |  | 0 | 1 | 2 | 2 | 3 |
| 6205032138 | 5.86 | 1.65 |  | 3.27 | 1.46 | 1 | 0 | 0 |  | 0 | 0 | 2 | 1 | 2 |
| 6205032140 | 5.07 | 1.23 |  | 2.71 | 2.31 | 1 | 0 | 1 |  | 0 | 0 | 2 | 2 | 1 |
| 6205032142 | 1.52 | 0.5 |  | 0.53 | 1.32 | 2 | 1 | 1 |  | 1 | 1 | 3 | 2 | 2 |
| 6205032146 | 2.16 | 0.86 |  | 0.88 | 0.83 | 0 | 0 | 1 |  | 0 | 0 | 1 | 1 | 1 |
| 6205032148 | 4.26 | 1.24 |  | 2.56 | 1.06 | 0 | 0 | 0 |  | 0 | 0 | 2 | 2 | 2 |
| 6205032149 | 4.56 | 1.21 |  | 2.43 | 2.05 | 1 | 1 | 2 |  | 0 | 1 | 1 | 1 | 1 |
| 6205032150 | 3.77 | 1.18 |  | 1.86 | 1.34 | 0 | 0 | 0 |  | 0 | 0 | 1 | 1 | 1 |
| 6205032203 | 8.33 | 1.54 |  | 5.37 | 3.2 | 2 | 0 | 0 |  | 0 | 0 | 2 | 2 | 3 |
| 6205032207 | 5 | 1.03 |  | 2.75 | 2.01 | 2 | 1 | 0 |  | 0 | 1 | 1 | 1 | 1 |
| 6205032208 | 4.64 | 1.57 |  | 2.43 | 1.09 | 2 | 1 | 0 |  | 0 | 1 | 1 | 1 | 1 |
| 6205032210 | 5.94 | 0.88 |  | 3.55 | 3.75 | 0 | 1 | 2 |  | 0 | 1 | 2 | 2 | 2 |
| 6205032211 | 3.14 | 1.05 |  | 1.42 | 0.92 | 0 | 0 | 1 |  | 0 | 0 | 2 | 1 | 2 |
| 6205032222 | 4.52 | 0.92 |  | 2.95 | 1.69 | 1 | 0 | 0 |  | 0 | 0 | 3 | 2 | 2 |
| 6205032223 | 4.11 | 0.78 |  | 1.99 | 3.06 | 2 | 1 | 0 |  | 0 | 0 | 2 | 2 | 2 |
| 6205032225 | 4.81 | 1.39 |  | 2.15 | 2.96 | 0 | 1 | 1 |  | 0 | 1 | 1 | 1 | 2 |
| 6205032226 | 5.57 | 1.29 |  | 3.18 | 2.51 | 2 | 0 | 0 |  | 0 | 0 | 3 | 3 | 3 |
| 6205032227 | 4.65 | 1.14 |  | 2.46 | 3.1 | 1 | 0 | 0 |  | 0 | 0 | 2 | 2 | 2 |
| 6205032228 | 4.6 | 1.02 |  | 2.08 | 4.7 | 0 | 0 | 1 |  | 0 | 0 | 1 | 1 | 2 |
| 6205032237 | 4.39 | 1.17 |  | 2.42 | 1.34 | 0 | 0 | 1 |  | 0 | 0 | 3 | 2 | 3 |
| 6205032243 | 4.59 | 1.22 |  | 2.19 | 2.33 | 1 | 0 | 0 |  | 0 | 0 | 1 | 1 | 1 |
| 6205032244 | 4.15 | 0.92 |  | 2.18 | 2.54 | 0 | 0 | 1 |  | 0 | 0 | 3 | 2 | 3 |
| 6205032245 | 3.2 | 1.01 |  | 1.52 | 1.83 | 2 | 1 | 0 |  | 0 | 1 | 3 | 2 | 2 |
| 6205032247 | 6.09 | 1.55 |  | 3.32 | 1.19 | 1 | 1 | 1 |  | 0 | 1 | 2 | 2 | 1 |
| 6205032248 | 3.1 | 0.86 |  | 1.85 | 0.67 | 1 | 1 | 1 |  | 1 | 1 | 1 | 1 | 1 |
| 6205032250 | 5.13 | 1.35 |  | 2.5 | 4.89 | 1 | 1 | 1 |  | 0 | 0 | 3 | 3 | 3 |
| 6205032306 | 5.18 | 1.56 |  | 2.62 | 2.09 | 2 | 0 | 1 |  | 0 | 0 | 3 | 2 | 3 |
| 6205032307 | 3.77 | 0.97 |  | 2.3 | 1.13 | 2 | 1 | 0 |  | 0 | 1 | 3 | 2 | 3 |
| 6205032308 | 3.48 | 0.97 |  | 1.79 | 1.43 | 1 | 1 | 1 |  | 0 | 1 | 1 | 1 | 1 |
| 6205032309 | 4.04 | 0.94 |  | 2.11 | 1.72 | 2 | 0 | 0 |  | 0 | 0 | 1 | 1 | 1 |
| 6205032310 | 4.58 | 1 |  | 2.56 | 2.71 | 2 | 0 | 2 |  | 0 | 0 | 2 | 1 | 2 |
| 6205032311 | 3.75 | 0.79 |  | 1.29 | 3.27 | 2 | 0 | 1 |  | 0 | 0 | 2 | 2 | 2 |
| 6205032313 | 4.56 | 0.95 |  | 2.46 | 2.94 | 2 | 0 | 0 |  | 0 | 0 | 2 | 2 | 2 |
| 6205032315 | 3.31 | 0.87 |  | 1.65 | 1.31 | 1 | 1 | 2 |  | 0 | 1 | 2 | 2 | 2 |
| 6205032319 | 3.73 | 0.98 |  | 2.59 | 0.65 | 1 | 0 | 0 |  | 0 | 0 | 1 | 1 | 1 |
| 6205032324 | 4.29 | 1.29 |  | 2.03 | 1.48 | 2 | 0 | 0 |  | 0 | 0 | 1 | 1 | 1 |
| 6205032326 | 2.67 | 0.92 |  | 1.13 | 1.5 | 0 | 0 | 0 |  | 0 | 0 | 3 | 2 | 3 |
| 6205032330 | 4.52 | 1.22 |  | 2.25 | 2.16 | 2 | 1 | 0 |  | 0 | 0 | 1 | 1 | 1 |
| 6205032332 | 3.73 | 1.09 |  | 1.74 | 1.51 | 0 | 1 | 1 |  | 0 | 1 | 1 | 1 | 1 |
| 6205032334 | 3.21 | 0.69 |  | 1.18 | 2.87 | 1 | 0 | 0 |  | 0 | 0 | 1 | 1 | 2 |
| 6205032336 | 4.25 | 1.23 |  | 2.51 | 0.82 | 1 | 0 | 1 |  | 0 | 0 | 1 | 1 | 1 |
| 6205032338 | 3.56 | 1.39 |  | 1.53 | 1.12 | 0 | 1 | 1 |  | 0 | 1 | 2 | 2 | 2 |
| 6205032339 | 3.73 | 1.21 |  | 2.01 | 1.08 | 0 | 1 | 1 |  | 0 | 1 | 2 | 1 | 1 |
| 6205032341 | 4.84 | 1.18 |  | 3.15 | 1.2 | 2 | 0 | 0 |  | 0 | 0 | 3 | 2 | 3 |
| 6205032343 | 4.71 | 1.69 |  | 2.45 | 0.72 | 1 | 0 | 0 |  | 0 | 0 | 2 | 1 | 1 |
| 6205032347 | 3.66 | 1.13 |  | 1.9 | 1.12 | 2 | 0 | 0 |  | 0 | 0 | 1 | 1 | 1 |
| 6205032348 | 4.98 | 1.06 |  | 2.44 | 2.84 | 1 | 0 | 1 |  | 0 | 0 | 1 | 1 | 1 |
| 6205032349 | 4.46 | 0.71 |  | 1.62 | 7.96 | 1 | 0 | 0 |  | 0 | 0 | 1 | 1 | 1 |
| 6205033101 | 4.53 | 1.34 |  | 2.46 | 1.2 | 2 | 0 | 0 |  | 0 | 0 | 1 | 1 | 1 |
| 6205033102 | 3.23 | 1.19 |  | 1.42 | 1.47 | 1 | 0 | 1 |  | 0 | 0 | 2 | 1 | 2 |
| 6205033103 | 5.07 | 1.35 |  | 2.89 | 1.36 | 2 | 0 | 0 |  | 0 | 0 | 1 | 1 | 2 |
| 6205033104 | 4.15 | 1 |  | 2.4 | 1.99 | 1 | 1 | 1 |  | 0 | 1 | 1 | 1 | 1 |
| 6205033106 | 2.72 | 0.96 |  | 1.46 | 0.44 | 2 | 1 | 0 |  | 0 | 1 | 1 | 1 | 1 |
| 6205033107 | 4.52 | 1.35 |  | 2.72 | 0.98 | 1 | 0 | 0 |  | 0 | 0 | 2 | 2 | 3 |
| 6205033108 | 4.27 | 0.99 |  | 1.85 | 3.25 | 1 | 0 | 2 |  | 0 | 0 | 1 | 1 | 2 |
| 6205033109 | 3.51 | 0.93 |  | 2.01 | 1 | 2 | 1 | 0 |  | 0 | 1 | 1 | 1 | 1 |
| 6205033111 | 3.62 | 0.77 |  | 1.85 | 2.06 | 0 | 1 | 1 |  | 0 | 1 | 1 | 1 | 1 |
| 6205033114 | 3.68 | 0.88 |  | 2.29 | 1.23 | 1 | 0 | 0 |  | 0 | 0 | 3 | 2 | 2 |
| 6205033118 | 2.96 | 0.9 |  | 1.22 | 1.19 | 2 | 1 | 2 |  | 0 | 1 | 1 | 1 | 1 |
| 6205033122 | 4.14 | 1.25 |  | 2.32 | 1.06 | 2 | 0 | 0 |  | 0 | 0 | 1 | 1 | 1 |
| 6205033125 | 4.41 | 0.87 |  | 2.34 | 3.2 | 1 | 0 | 0 |  | 0 | 0 | 2 | 2 | 2 |
| 6205033127 | 3.28 | 1.3 |  | 1.57 | 0.49 | 2 | 1 | 1 |  | 0 | 1 | 1 | 1 | 1 |
| 6205033129 | 3.97 | 1.26 |  | 1.94 | 1.78 | 0 | 0 | 1 |  | 0 | 0 | 1 | 1 | 1 |
| 6205033131 | 5.55 | 1.54 |  | 3.02 | 1.83 | 1 | 0 | 0 |  | 0 | 0 | 1 | 1 | 1 |
| 6205033133 | 2.81 | 0.73 |  | 1.43 | 1.58 | 1 | 0 | 1 |  | 0 | 0 | 2 | 2 | 1 |
| 6205033134 | 4.26 | 1.15 |  | 2.08 | 1.74 | 2 | 0 | 0 |  | 0 | 0 | 2 | 1 | 3 |
| 6205033135 | 4.56 | 0.8 |  | 3.06 | 2.02 | 1 | 1 | 2 |  | 0 | 1 | 2 | 2 | 2 |
| 6205033136 | 2.13 | 0.6 |  | 1.03 | 0.72 | 1 | 0 | 0 |  | 0 | 0 | 3 | 2 | 3 |
| 6205033139 | 4.81 | 1.15 |  | 2.97 | 1.09 | 1 | 1 | 2 |  | 1 | 0 | 2 | 2 | 3 |
| 6205033140 | 2.06 | 0.53 |  | 0.8 | 1.68 | 2 | 0 | 1 |  | 0 | 0 | 2 | 2 | 2 |
| 6205033141 | 5.59 | 1.3 |  | 3.09 | 2.29 | 1 | 0 | 0 |  | 0 | 0 | 2 | 1 | 2 |
| 6205033143 | 4.79 | 1.5 |  | 2.29 | 1.73 | 1 | 1 | 2 |  | 0 | 1 | 2 | 2 | 2 |
| 6205033148 | 3.95 | 0.96 |  | 2.19 | 1.66 | 1 | 1 | 2 |  | 0 | 1 | 2 | 2 | 3 |
| 6205033149 | 3.5 | 1.11 |  | 1.91 | 1.06 | 2 | 0 | 0 |  | 0 | 0 | 3 | 2 | 2 |
| 6205033201 | 3.95 | 1.38 |  | 1.85 | 1.45 | 2 | 1 | 0 |  | 0 | 1 | 3 | 2 | 3 |
| 6205033202 | 4.15 | 1.08 |  | 2.55 | 1.25 | 0 | 1 | 0 |  | 0 | 1 | 1 | 1 | 2 |
| 6205033205 | 3.34 | 1.02 |  | 1.52 | 1.53 | 1 | 0 | 0 |  | 0 | 0 | 1 | 1 | 2 |
| 6205033206 | 11.14 | 0.69 |  | 5.28 | 16.05 | 1 | 0 | 0 |  | 0 | 0 | 2 | 2 | 2 |
| 6205033208 | 3.12 | 0.89 |  | 1.11 | 1.76 | 0 | 0 | 1 |  | 0 | 0 | 1 | 1 | 1 |
| 6205033214 | 5.06 | 1.32 |  | 2.83 | 1.82 | 0 | 0 | 0 |  | 0 | 0 | 1 | 1 | 2 |
| 6205033216 | 3.36 | 1.19 |  | 1.66 | 1.27 | 2 | 0 | 0 |  | 0 | 0 | 1 | 1 | 2 |
| 6205033217 | 3.43 | 1.07 |  | 1.66 | 1.57 | 2 | 1 | 0 |  | 0 | 1 | 1 | 1 | 1 |
| 6205033218 | 3.29 | 0.91 |  | 1.61 | 1.15 | 2 | 1 | 1 |  | 0 | 1 | 1 | 1 | 1 |
| 6205033221 | 7.24 | 0.46 |  | 0.53 | 12.21 | 0 | 0 | 0 |  | 0 | 0 | 2 | 2 | 3 |
| 6205033223 | 2.88 | 0.97 |  | 1.42 | 0.82 | 1 | 0 | 0 |  | 0 | 0 | 1 | 1 | 1 |
| 6205033224 | 2.78 | 0.73 |  | 1.74 | 0.9 | 0 | 1 | 1 |  | 0 | 0 | 1 | 1 | 1 |
| 6205033225 | 2.95 | 0.9 |  | 1.56 | 1.05 | 2 | 1 | 0 |  | 0 | 1 | 1 | 1 | 1 |
| 6205033229 | 2.44 | 0.87 |  | 1.09 | 1.42 | 0 | 0 | 1 |  | 0 | 0 | 2 | 1 | 2 |
| 6205033230 | 4.29 | 1.6 |  | 1.95 | 1.08 | 2 | 0 | 1 |  | 0 | 1 | 1 | 1 | 2 |
| 6205033232 | 4.32 | 1.81 |  | 2.14 | 0.67 | 1 | 0 | 0 |  | 0 | 0 | 1 | 1 | 1 |
| 6205033233 | 2.3 | 0.53 |  | 1.21 | 1.15 | 0 | 1 | 0 |  | 0 | 1 | 1 | 1 | 1 |
| 6205033238 | 3.68 | 1 |  | 2.17 | 1.19 | 0 | 0 | 0 |  | 0 | 0 | 2 | 1 | 1 |
| 6205033239 | 4.31 | 1.46 |  | 2.08 | 1.04 | 1 | 0 | 0 |  | 0 | 0 | 1 | 1 | 1 |
| 6205033240 | 3.92 | 1.05 |  | 2.39 | 0.99 | 2 | 1 | 0 |  | 0 | 1 | 3 | 2 | 1 |
| 6205033241 | 2.58 | 1.06 |  | 1.05 | 0.62 | 0 | 1 | 0 |  | 0 | 1 | 1 | 1 | 1 |
| 6205033242 | 3.51 | 0.9 |  | 1.95 | 1.37 | 1 | 1 | 0 |  | 0 | 0 | 2 | 1 | 1 |
| 6205033243 | 5.22 | 2.22 |  | 2.14 | 1.47 | 2 | 1 | 0 |  | 0 | 1 | 2 | 1 | 1 |
| 6205033245 | 5.87 | 1.72 |  | 3.56 | 1.43 | 2 | 0 | 0 |  | 0 | 0 | 2 | 2 | 2 |
| 6205033249 | 3.15 | 0.8 |  | 1.67 | 1.44 | 0 | 1 | 1 |  | 0 | 1 | 1 | 1 | 1 |
| 6205033250 | 4.53 | 1.03 |  | 2.98 | 1.24 | 2 | 1 | 0 |  | 0 | 1 | 2 | 1 | 1 |
| 6205033302 | 3.21 | 1.17 |  | 1.43 | 0.81 | 2 | 1 | 2 |  | 1 | 1 | 1 | 1 | 1 |
| 6205033308 | 3.28 | 1.01 |  | 1.8 | 0.84 | 1 | 1 | 2 |  | 0 | 1 | 1 | 1 | 1 |
| 6205033309 | 3.38 | 0.65 |  | 1.7 | 2.15 | 1 | 1 | 1 |  | 0 | 1 | 2 | 2 | 2 |
| 6205033310 | 3.18 | 1.03 |  | 1.68 | 0.62 | 1 | 1 | 2 |  | 0 | 1 | 1 | 1 | 1 |
| 6205033323 | 4.82 | 1.1 |  | 2.73 | 1.87 | 1 | 0 | 1 |  | 0 | 0 | 2 | 2 | 3 |
| 6205033325 | 4.7 | 0.69 |  | 3.51 | 0.83 | 0 | 1 | 0 |  | 0 | 1 | 2 | 2 | 2 |
| 6205033333 | 2.76 | 0.78 |  | 1.53 | 0.82 | 0 | 1 | 1 |  | 0 | 1 | 2 | 2 | 2 |
| 6205033334 | 3.74 | 1.13 |  | 1.62 | 2.18 | 2 | 0 | 0 |  | 0 | 0 | 2 | 2 | 3 |
| 6205033336 | 3.74 | 1.11 |  | 1.86 | 1.88 | 1 | 0 | 0 |  | 0 | 0 | 2 | 2 | 2 |
| 6205033337 | 3.72 | 1.02 |  | 1.59 | 2.56 | 1 | 0 | 2 |  | 0 | 0 | 3 | 2 | 3 |
| 6205033339 | 4.11 | 1.15 |  | 2.42 | 1.01 | 1 | 1 | 1 |  | 0 | 1 | 1 | 1 | 1 |
| 6205033349 | 3.71 | 0.91 |  | 2.4 | 1.31 | 1 | 0 | 0 |  | 0 | 0 | 2 | 2 | 2 |
| 6205033350 | 2.93 | 0.69 |  | 1.72 | 1.11 | 1 | 1 | 1 |  | 0 | 1 | 2 | 2 | 3 |
| 6205034101 | 4.3 | 1 |  | 2.37 | 2.6 | 1 | 0 | 1 |  | 0 | 0 | 3 | 2 | 3 |
| 6205034103 | 4.65 | 1.14 |  | 2.57 | 1.62 | 0 | 0 | 0 |  | 0 | 0 | 1 | 1 | 1 |
| 6205034105 | 3.07 | 1.07 |  | 1.53 | 1.15 | 2 | 1 | 1 |  | 0 | 0 | 1 | 1 | 1 |
| 6205034109 | 3.72 | 1.09 |  | 1.84 | 1.59 | 1 | 0 | 1 |  | 0 | 0 | 2 | 2 | 3 |
| 6205034110 | 3.67 | 1.07 |  | 1.98 | 1.26 | 1 | 0 | 0 |  | 0 | 0 | 2 | 2 | 3 |
| 6205034111 | 4.49 | 1.02 |  | 2.55 | 2.06 | 2 | 0 | 0 |  | 0 | 0 | 3 | 3 | 3 |
| 6205034113 | 4.07 | 1.05 |  | 2.42 | 1.55 | 2 | 1 | 0 |  | 0 | 1 | 1 | 1 | 1 |
| 6205034114 | 4.4 | 0.92 |  | 2.8 | 1.75 | 1 | 1 | 0 |  | 0 | 0 | 1 | 1 | 1 |
| 6205034116 | 5.21 | 1.09 |  | 2.64 | 3.93 | 2 | 0 | 0 |  | 0 | 0 | 1 | 1 | 3 |
| 6205034117 | 3.16 | 0.89 |  | 1.54 | 1.63 | 1 | 1 | 1 |  | 0 | 0 | 2 | 2 | 2 |
| 6205034119 | 2.2 | 0.57 |  | 1.29 | 0.75 | 0 | 0 | 1 |  | 0 | 0 | 2 | 1 | 2 |
| 6205034121 | 2.71 | 0.75 |  | 1.35 | 0.91 | 1 | 1 | 0 |  | 0 | 1 | 1 | 1 | 1 |
| 6205034123 | 3.02 | 0.75 |  | 1.49 | 1.24 | 0 | 0 | 1 |  | 0 | 0 | 1 | 1 | 1 |
| 6205034124 | 3.18 | 1.03 |  | 1.53 | 1.17 | 1 | 0 | 0 |  | 0 | 0 | 1 | 1 | 1 |
| 6205034126 | 5.65 | 1 |  | 4.2 | 1.42 | 0 | 0 | 0 |  | 0 | 0 | 2 | 1 | 1 |
| 6205034130 | 4.46 | 1.27 |  | 2.46 | 1.21 | 0 | 1 | 1 |  | 1 | 1 | 2 | 2 | 2 |
| 6205034132 | 4.4 | 0.78 |  | 2.39 | 2.33 | 1 | 1 | 1 |  | 0 | 1 | 2 | 1 | 2 |
| 6205034134 | 3.62 | 1.06 |  | 2.12 | 1.11 | 0 | 0 | 2 |  | 0 | 0 | 2 | 1 | 2 |
| 6205034135 | 5.2 | 1.32 |  | 3.09 | 2.08 | 0 | 0 | 1 |  | 0 | 0 | 2 | 1 | 1 |
| 6205034136 | 4.07 | 1.21 |  | 2.07 | 1.41 | 2 | 0 | 0 |  | 0 | 0 | 1 | 1 | 2 |
| 6205034139 | 3.02 | 1.19 |  | 1.2 | 0.9 | 2 | 1 | 0 |  | 0 | 0 | 1 | 1 | 1 |
| 6205034140 | 3.7 | 1.07 |  | 2.07 | 1.13 | 2 | 0 | 0 |  | 0 | 0 | 1 | 1 | 1 |
| 6205034141 | 4.4 | 1.08 |  | 2.34 | 1.94 | 1 | 0 | 0 |  | 0 | 0 | 1 | 1 | 1 |
| 6205034142 | 4.22 | 1.49 |  | 2.07 | 0.76 | 2 | 1 | 1 |  | 0 | 1 | 1 | 1 | 1 |
| 6205034143 | 3.78 | 1.12 |  | 1.63 | 1.43 | 2 | 1 | 1 |  | 0 | 0 | 1 | 1 | 1 |
| 6205034147 | 3.29 | 0.89 |  | 1.84 | 0.95 | 0 | 1 | 0 |  | 0 | 0 | 2 | 2 | 2 |
| 6205034201 | 4.75 | 1.37 |  | 2.64 | 0.9 | 1 | 0 | 0 |  | 0 | 0 | 1 | 1 | 1 |
| 6205034205 | 2.76 | 0.75 |  | 1.3 | 1.77 | 0 | 1 | 1 |  | 0 | 0 | 1 | 1 | 1 |
| 6205034207 | 4.22 | 1.19 |  | 2.43 | 1.17 | 1 | 0 | 0 |  | 0 | 0 | 2 | 2 | 1 |
| 6205034215 | 3.87 | 1.45 |  | 1.88 | 0.88 | 1 | 0 | 1 |  | 0 | 0 | 1 | 1 | 1 |
| 6205034216 | 3.8 | 0.84 |  | 1.61 | 2.84 | 0 | 0 | 0 |  | 0 | 0 | 1 | 1 | 1 |
| 6205034219 | 3.11 | 0.75 |  | 1.53 | 1.63 | 1 | 1 | 0 |  | 0 | 1 | 1 | 1 | 1 |
| 6205034222 | 4.97 | 0.95 |  | 3.18 | 1.88 | 0 | 1 | 2 |  | 0 | 1 | 2 | 2 | 1 |
| 6205034227 | 3.76 | 1.24 |  | 1.73 | 1.12 | 2 | 0 | 0 |  | 0 | 0 | 1 | 1 | 1 |
| 6205034228 | 3.85 | 1.02 |  | 1.86 | 1.97 | 1 | 1 | 1 |  | 0 | 1 | 1 | 1 | 1 |
| 6205034230 | 4.02 | 1.2 |  | 2.1 | 1.27 | 1 | 0 | 0 |  | 0 | 0 | 1 | 1 | 1 |
| 6205034236 | 3.38 | 1.12 |  | 1.86 | 1.24 | 2 | 1 | 0 |  | 0 | 1 | 3 | 3 | 3 |
| 6205034238 | 4.27 | 1.26 |  | 1.92 | 2.62 | 1 | 0 | 2 |  | 0 | 0 | 3 | 3 | 3 |
| 6205034240 | 4.59 | 1.17 |  | 2.72 | 1.69 | 1 | 0 | 0 |  | 0 | 0 | 2 | 2 | 2 |
| 6205034242 | 4.73 | 1.31 |  | 2.64 | 1.35 | 2 | 0 | 0 |  | 0 | 0 | 2 | 2 | 2 |
| 6205034243 | 3.81 | 0.88 |  | 1.82 | 3.6 | 1 | 1 | 2 |  | 0 | 1 | 3 | 2 | 3 |
| 6205034249 | 4.55 | 1.43 |  | 2.29 | 1.8 | 1 | 0 | 0 |  | 0 | 0 | 2 | 2 | 3 |
| 6205034250 | 5.35 | 1.26 |  | 3.29 | 1.34 | 2 | 0 | 0 |  | 0 | 0 | 1 | 1 | 1 |
| 6205034302 | 4.33 | 0.92 |  | 2.58 | 1.84 | 2 | 1 | 1 |  | 0 | 1 | 1 | 1 | 2 |
| 6205034305 | 3.05 | 0.68 |  | 1.79 | 1.15 | 2 | 0 | 0 |  | 0 | 0 | 2 | 2 | 3 |
| 6205034306 | 4.37 | 1.17 |  | 2.5 | 1.45 | 1 | 0 | 0 |  | 0 | 0 | 1 | 1 | 1 |
| 6205034310 | 3.3 | 0.88 |  | 1.78 | 1.05 | 1 | 0 | 0 |  | 0 | 0 | 1 | 1 | 1 |
| 6205034312 | 4.99 | 1.27 |  | 2.96 | 1.98 | 2 | 0 | 0 |  | 0 | 0 | 2 | 2 | 2 |
| 6205034313 | 3.8 | 1.12 |  | 2.14 | 1.5 | 0 | 0 | 1 |  | 0 | 0 | 2 | 2 | 2 |
| 6205034315 | 2.36 | 0.73 |  | 1.36 | 0.83 | 2 | 0 | 0 |  | 0 | 0 | 1 | 1 | 1 |
| 6205034316 | 4.08 | 1.04 |  | 2.26 | 1.7 | 2 | 0 | 1 |  | 0 | 0 | 1 | 1 | 2 |
| 6205034317 | 4.05 | 1.36 |  | 2.09 | 1.03 | 1 | 0 | 0 |  | 0 | 0 | 1 | 1 | 2 |
| 6205034321 | 5 | 0.91 |  | 3.3 | 2.3 | 2 | 0 | 0 |  | 0 | 0 | 3 | 3 | 3 |
| 6205034323 | 5.65 | 0.87 |  | 1.91 | 5.78 | 0 | 0 | 1 |  | 0 | 0 | 2 | 1 | 2 |
| 6205034324 | 3.24 | 0.84 |  | 1.83 | 1.24 | 0 | 1 | 1 |  | 0 | 1 | 1 | 1 | 1 |
| 6205034325 | 3.72 | 0.82 |  | 2.33 | 1.42 | 0 | 1 | 1 |  | 1 | 0 | 2 | 2 | 2 |
| 6205034327 | 3.68 | 1.39 |  | 1.62 | 1.3 | 1 | 1 | 0 |  | 0 | 1 | 1 | 1 | 1 |
| 6205034328 | 5.48 | 1.13 |  | 3.28 | 2.58 | 1 | 0 | 0 |  | 0 | 0 | 2 | 2 | 2 |
| 6205034330 | 3.24 | 0.94 |  | 1.69 | 1.31 | 2 | 0 | 0 |  | 0 | 0 | 1 | 1 | 1 |
| 6205034333 | 3.51 | 1.27 |  | 0.84 | 1.9 | 2 | 1 | 0 |  | 0 | 1 | 1 | 1 | 1 |
| 6205034334 | 3.53 | 1.03 |  | 2.05 | 1.14 | 2 | 1 | 0 |  | 0 | 1 | 1 | 1 | 1 |
| 6205034336 | 4.28 | 0.94 |  | 2.2 | 3.26 | 0 | 1 | 2 |  | 0 | 1 | 1 | 1 | 1 |
| 6205034338 | 5.62 | 1.04 |  | 3.52 | 3.04 | 2 | 0 | 1 |  | 0 | 0 | 2 | 2 | 3 |
| 6205034340 | 3.79 | 0.77 |  | 2.29 | 2.64 | 1 | 0 | 2 |  | 0 | 0 | 2 | 2 | 2 |
| 6205034341 | 3.85 | 1.18 |  | 2.03 | 0.88 | 0 | 1 | 1 |  | 0 | 1 | 1 | 1 | 2 |
| 6205034343 | 3.08 | 0.83 |  | 1.53 | 1.41 | 1 | 1 | 1 |  | 0 | 0 | 3 | 2 | 3 |
| 6205034345 | 5.49 | 1.2 |  | 3.37 | 2.06 | 2 | 1 | 1 |  | 0 | 1 | 1 | 1 | 1 |
| 6205034346 | 3.66 | 0.93 |  | 2.01 | 1.57 | 0 | 0 | 0 |  | 0 | 0 | 2 | 2 | 1 |
| 6205034350 | 6.23 | 0.74 |  | 2.49 | 10.4 | 1 | 0 | 0 |  | 0 | 0 | 2 | 2 | 3 |
| 6221031102 | 4.34 | 1.13 |  | 2.65 | 1.47 | 2 | 0 | 0 |  | 0 | 0 | 3 | 3 | 3 |
| 6221031106 | 2.97 | 0.63 |  | 1.68 | 1.82 | 1 | 1 | 2 |  | 0 | 0 | 3 | 3 | 3 |
| 6221031108 | 3 | 1.14 |  | 1.31 | 1.13 | 1 | 0 | 1 |  | 0 | 0 | 3 | 2 | 2 |
| 6221031110 | 3.94 | 1.12 |  | 2.22 | 1.09 | 0 | 0 | 1 |  | 0 | 0 | 2 | 2 | 1 |
| 6221031112 | 4.12 | 0.88 |  | 2.56 | 1.15 | 0 | 1 | 1 |  | 0 | 0 | 2 | 2 | 2 |
| 6221031116 | 5.33 | 1.06 |  | 2.86 | 3.91 | 2 | 0 | 0 |  | 0 | 0 | 2 | 2 | 1 |
| 6221031117 | 4.35 | 1 |  | 2.34 | 1.77 | 1 | 0 | 2 |  | 0 | 0 | 2 | 1 | 1 |
| 6221031118 | 3.07 | 1.18 |  | 1.54 | 0.53 | 0 | 0 | 1 |  | 0 | 0 | 1 | 1 | 1 |
| 6221031119 | 2.97 | 1.11 |  | 1.56 | 0.45 | 0 | 0 | 1 |  | 0 | 0 | 1 | 1 | 1 |
| 6221031120 | 4.67 | 0.84 |  | 2.14 | 5.46 | 1 | 1 | 0 |  | 1 | 0 | 3 | 2 | 3 |
| 6221031121 | 7.14 | 0.89 |  | 4.54 | 3.42 | 2 | 0 | 0 |  | 0 | 0 | 3 | 3 | 3 |
| 6221031123 | 3.98 | 0.83 |  | 1.9 | 2.15 | 2 | 0 | 0 |  | 0 | 0 | 1 | 1 | 1 |
| 6221031125 | 5.17 | 0.97 |  | 2.59 | 3.6 | 2 | 0 | 0 |  | 0 | 0 | 2 | 2 | 2 |
| 6221031129 | 5.56 | 0.98 |  | 3.67 | 2.04 | 0 | 0 | 1 |  | 0 | 0 | 3 | 3 | 3 |
| 6221031130 | 8.08 | 0.79 |  | 2.92 | 16.05 | 0 | 1 | 1 |  | 1 | 1 | 3 | 2 | 2 |
| 6221031131 | 4.59 | 1.28 |  | 2.56 | 1.66 | 0 | 1 | 1 |  | 0 | 1 | 2 | 2 | 1 |
| 6221031133 | 5 | 1.08 |  | 2.9 | 2.3 | 0 | 0 | 0 |  | 0 | 0 | 2 | 2 | 2 |
| 6221031136 | 5.85 | 1.05 |  | 2.71 | 3.72 | 1 | 0 | 0 |  | 0 | 0 | 2 | 2 | 2 |
| 6221031138 | 4.41 | 1.02 |  | 2.3 | 2.58 | 1 | 1 | 1 |  | 0 | 1 | 1 | 1 | 1 |
| 6221031140 | 4.37 | 1.15 |  | 2.66 | 1.39 | 1 | 0 | 1 |  | 0 | 0 | 3 | 2 | 2 |
| 6221031143 | 3.88 | 0.89 |  | 2.17 | 1.69 | 1 | 0 | 0 |  | 0 | 0 | 3 | 2 | 2 |
| 6221031146 | 3.77 | 0.86 |  | 2.14 | 1.72 | 0 | 1 | 0 |  | 0 | 1 | 2 | 1 | 1 |
| 6221031147 | 4.43 | 0.79 |  | 1.66 | 5.58 | 1 | 1 | 1 |  | 0 | 1 | 2 | 2 | 3 |
| 6221031148 | 3.07 | 0.92 |  | 1.62 | 0.86 | 1 | 0 | 0 |  | 0 | 0 | 1 | 1 | 1 |
| 6221031149 | 4.46 | 1.07 |  | 2.44 | 1.73 | 0 | 0 | 0 |  | 0 | 0 | 2 | 1 | 1 |
| 6221031150 | 3.36 | 0.81 |  | 1.37 | 2.57 | 1 | 0 | 1 |  | 0 | 0 | 3 | 2 | 3 |
| 6221031203 | 5.19 | 0.97 |  | 2.01 | 5.65 | 0 | 1 | 1 |  | 0 | 1 | 2 | 2 | 2 |
| 6221031205 | 3.46 | 0.98 |  | 1.88 | 1.09 | 1 | 0 | 1 |  | 0 | 0 | 2 | 2 | 2 |
| 6221031207 | 3.44 | 0.83 |  | 1.75 | 1.44 | 0 | 0 | 1 |  | 0 | 0 | 2 | 2 | 2 |
| 6221031212 | 5.47 | 1.61 |  | 3.11 | 0.92 | 0 | 1 | 0 |  | 0 | 1 | 1 | 1 | 1 |
| 6221031214 | 2.83 | 0.81 |  | 1.69 | 1.26 | 2 | 1 | 1 |  | 0 | 0 | 3 | 3 | 3 |
| 6221031217 | 3.62 | 0.92 |  | 1.85 | 1.09 | 0 | 0 | 0 |  | 0 | 0 | 1 | 1 | 1 |
| 6221031219 | 2.83 | 1.19 |  | 0.95 | 1.04 | 0 | 1 | 1 |  | 0 | 0 | 1 | 1 | 1 |
| 6221031224 | 3.24 | 1.02 |  | 1.61 | 1.35 | 1 | 1 | 2 |  | 0 | 1 | 2 | 2 | 2 |
| 6221031230 | 3.09 | 0.61 |  | 1.6 | 1.55 | 2 | 0 | 0 |  | 0 | 0 | 2 | 2 | 2 |
| 6221031234 | 3.51 | 0.79 |  | 2.01 | 1.35 | 0 | 1 | 1 |  | 0 | 0 | 2 | 2 | 2 |
| 6221031236 | 3.34 | 1.01 |  | 1.95 | 0.65 | 1 | 1 | 1 |  | 0 | 0 | 3 | 3 | 2 |
| 6221031237 | 3.36 | 0.94 |  | 1.9 | 1.18 | 2 | 1 | 1 |  | 0 | 1 | 1 | 1 | 1 |
| 6221031243 | 4.99 | 0.76 |  | 3.61 | 1.59 | 1 | 1 | 2 |  | 0 | 0 | 2 | 2 | 2 |
| 6221031248 | 3.33 | 0.7 |  | 1.26 | 3.99 | 2 | 0 | 0 |  | 0 | 0 | 2 | 2 | 2 |
| 6221031301 | 3.35 | 1.19 |  | 1.85 | 0.72 | 2 | 1 | 1 |  | 0 | 1 | 2 | 1 | 1 |
| 6221031302 | 4.7 | 1.1 |  | 2.86 | 2.03 | 0 | 1 | 1 |  | 1 | 1 | 2 | 2 | 2 |
| 6221031303 | 3.16 | 0.67 |  | 1.78 | 1.72 | 2 | 1 | 1 |  | 0 | 0 | 2 | 2 | 2 |
| 6221031304 | 2.53 | 0.81 |  | 1.42 | 0.6 | 0 | 0 | 1 |  | 0 | 0 | 1 | 1 | 1 |
| 6221031306 | 4.26 | 0.81 |  | 2.26 | 2.01 | 2 | 0 | 0 |  | 0 | 0 | 2 | 2 | 2 |
| 6221031309 | 2.96 | 0.77 |  | 1.73 | 1.05 | 0 | 1 | 1 |  | 0 | 1 | 2 | 2 | 2 |
| 6221031311 | 3.31 | 0.71 |  | 1.79 | 1.7 | 1 | 0 | 2 |  | 0 | 0 | 2 | 2 | 3 |
| 6221031312 | 5.21 | 1.22 |  | 3.25 | 1.36 | 2 | 1 | 0 |  | 0 | 1 | 1 | 1 | 1 |
| 6221031314 | 4.97 | 1.26 |  | 2.8 | 1.48 | 0 | 0 | 1 |  | 0 | 0 | 1 | 1 | 1 |
| 6221031315 | 2.91 | 0.87 |  | 1.49 | 0.86 | 2 | 0 | 0 |  | 0 | 0 | 2 | 2 | 2 |
| 6221031318 | 4.02 | 1 |  | 2.07 | 1.46 | 0 | 0 | 1 |  | 0 | 0 | 2 | 2 | 2 |
| 6221031320 | 5.75 | 0.98 |  | 2.22 | 6.49 | 2 | 0 | 0 |  | 0 | 0 | 1 | 1 | 1 |
| 6221031329 | 3.4 | 0.7 |  | 2.13 | 1.22 | 0 | 0 | 1 |  | 0 | 0 | 2 | 2 | 2 |
| 6221031332 | 2.58 | 0.64 |  | 1.43 | 0.83 | 1 | 1 | 1 |  | 0 | 1 | 3 | 2 | 2 |
| 6221031333 | 3.46 | 1.1 |  | 1.86 | 0.54 | 0 | 0 | 1 |  | 0 | 0 | 3 | 2 | 2 |
| 6221031335 | 3.3 | 0.59 |  | 1.49 | 4.82 | 0 | 1 | 1 |  | 0 | 1 | 3 | 2 | 3 |
| 6221031337 | 4.28 | 1.18 |  | 2.19 | 3.15 | 2 | 1 | 1 |  | 1 | 1 | 2 | 2 | 2 |
| 6221031344 | 3.36 | 0.73 |  | 1.66 | 2.77 | 0 | 1 | 1 |  | 1 | 1 | 3 | 2 | 3 |
| 6221031345 | 2.17 | 0.55 |  | 0.88 | 1.57 | 0 | 0 | 2 |  | 0 | 0 | 2 | 1 | 2 |
| 6221031347 | 2.86 | 0.95 |  | 1.37 | 0.72 | 1 | 0 | 0 |  | 0 | 0 | 2 | 1 | 1 |
| 6221031348 | 4.14 | 1.22 |  | 2.3 | 1.07 | 0 | 1 | 2 |  | 0 | 0 | 2 | 2 | 2 |
| 6221031349 | 3.5 | 0.88 |  | 2 | 1.57 | 0 | 1 | 1 |  | 1 | 1 | 3 | 2 | 3 |
| 6221031350 | 3.99 | 1.16 |  | 2.28 | 1.09 | 1 | 0 | 1 |  | 0 | 0 | 1 | 1 | 1 |
| 6221032101 | 3 | 0.63 |  | 1.78 | 1.48 | 1 | 1 | 2 |  | 0 | 1 | 3 | 3 | 3 |
| 6221032106 | 3.74 | 0.89 |  | 2.01 | 1.6 | 1 | 1 | 0 |  | 1 | 1 | 3 | 2 | 2 |
| 6221032113 | 4.42 | 1.12 |  | 2.62 | 1.13 | 1 | 0 | 2 |  | 0 | 0 | 1 | 1 | 1 |
| 6221032115 | 3.73 | 1 |  | 2.01 | 1.42 | 2 | 1 | 2 |  | 0 | 0 | 1 | 1 | 1 |
| 6221032124 | 3.21 | 0.68 |  | 2 | 1.13 | 0 | 1 | 1 |  | 0 | 0 | 3 | 3 | 2 |
| 6221032128 | 2.95 | 0.81 |  | 1.74 | 0.59 | 0 | 0 | 1 |  | 0 | 0 | 2 | 1 | 1 |
| 6221032133 | 4.11 | 1.26 |  | 2.31 | 0.92 | 2 | 0 | 0 |  | 0 | 0 | 2 | 2 | 1 |
| 6221032139 | 3.64 | 0.94 |  | 2.24 | 0.93 | 0 | 0 | 2 |  | 0 | 0 | 1 | 1 | 1 |
| 6221032141 | 3.99 | 0.97 |  | 2.32 | 1.08 | 0 | 0 | 1 |  | 0 | 0 | 1 | 1 | 1 |
| 6221032142 | 3.07 | 0.94 |  | 1.7 | 0.92 | 1 | 0 | 1 |  | 0 | 0 | 3 | 3 | 3 |
| 6221032144 | 3.82 | 0.88 |  | 2.13 | 1.16 | 0 | 0 | 0 |  | 0 | 0 | 3 | 3 | 2 |
| 6221032147 | 2.38 | 0.57 |  | 1.26 | 1.4 | 0 | 1 | 1 |  | 0 | 0 | 3 | 3 | 3 |
| 6221032204 | 3.46 | 0.66 |  | 1.73 | 2.98 | 2 | 0 | 0 |  | 0 | 0 | 3 | 2 | 3 |
| 6221032218 | 4.27 | 1.08 |  | 2.07 | 1.14 | 2 | 0 | 0 |  | 0 | 0 | 1 | 1 | 1 |
| 6221032220 | 2.79 | 0.56 |  | 1.18 | 2.32 | 0 | 1 | 2 |  | 0 | 0 | 3 | 3 | 3 |
| 6221032223 | 2.52 | 0.83 |  | 1.25 | 0.52 | 0 | 1 | 1 |  | 1 | 1 | 1 | 1 | 1 |
| 6221032224 | 3.25 | 0.87 |  | 1.62 | 1.37 | 2 | 0 | 0 |  | 0 | 0 | 2 | 2 | 2 |
| 6221032227 | 2.55 | 1.1 |  | 0.98 | 0.45 | 1 | 0 | 0 |  | 0 | 0 | 2 | 1 | 1 |
| 6221032228 | 2.84 | 0.85 |  | 1.58 | 0.54 | 0 | 1 | 0 |  | 0 | 1 | 1 | 1 | 1 |
| 6221032229 | 2.82 | 0.66 |  | 1.65 | 0.83 | 2 | 1 | 1 |  | 0 | 1 | 2 | 2 | 2 |
| 6221032230 | 3.8 | 0.73 |  | 2.1 | 2.93 | 2 | 1 | 0 |  | 0 | 0 | 2 | 2 | 3 |
| 6221032231 | 3.93 | 0.6 |  | 1.86 | 4.82 | 0 | 1 | 1 |  | 0 | 1 | 2 | 2 | 2 |
| 6221032234 | 3.73 | 0.95 |  | 2.37 | 0.84 | 1 | 0 | 0 |  | 0 | 0 | 2 | 1 | 2 |
| 6221032237 | 2.99 | 0.88 |  | 1.58 | 1.07 | 1 | 1 | 1 |  | 0 | 0 | 2 | 1 | 1 |
| 6221032238 | 4 | 1.31 |  | 1.39 | 1.82 | 0 | 1 | 1 |  | 1 | 1 | 3 | 2 | 2 |
| 6221032239 | 3.6 | 1.3 |  | 1.5 | 1.16 | 1 | 0 | 0 |  | 0 | 0 | 1 | 1 | 1 |
| 6221032242 | 4.08 | 0.97 |  | 2.05 | 2.83 | 1 | 0 | 1 |  | 0 | 0 | 3 | 2 | 3 |
| 6221032245 | 2.71 | 1.27 |  | 0.66 | 0.63 | 1 | 1 | 0 |  | 0 | 1 | 2 | 1 | 1 |
| 6221032246 | 1.98 | 0.85 |  | 0.85 | 0.43 | 0 | 1 | 1 |  | 0 | 1 | 2 | 2 | 1 |
| 6221032248 | 2.09 | 0.54 |  | 1.05 | 1.53 | 0 | 0 | 0 |  | 0 | 0 | 2 | 2 | 2 |
| 6221032249 | 3.41 | 0.73 |  | 2 | 2.16 | 1 | 0 | 2 |  | 0 | 0 | 3 | 2 | 2 |
| 6221032250 | 4.36 | 0.77 |  | 1.87 | 3.97 | 1 | 0 | 2 |  | 0 | 0 | 2 | 1 | 2 |
| 6221032302 | 4.04 | 0.82 |  | 2.39 | 2.28 | 0 | 1 | 2 |  | 0 | 0 | 3 | 2 | 3 |
| 6221032303 | 4.72 | 1.01 |  | 2.7 | 2.73 | 1 | 1 | 2 |  | 0 | 0 | 2 | 2 | 2 |
| 6221032304 | 4.19 | 1.06 |  | 2.53 | 2.25 | 0 | 1 | 2 |  | 1 | 0 | 3 | 3 | 2 |
| 6221032314 | 4.15 | 0.84 |  | 2.23 | 3.27 | 1 | 0 | 2 |  | 0 | 0 | 2 | 2 | 3 |
| 6221032321 | 3.01 | 0.7 |  | 1.77 | 1.5 | 0 | 1 | 2 |  | 0 | 0 | 3 | 3 | 3 |
| 6221032325 | 2.7 | 0.77 |  | 1.27 | 1.02 | 1 | 0 | 0 |  | 0 | 0 | 2 | 2 | 2 |
| 6221032326 | 3.4 | 1.03 |  | 1.7 | 2.02 | 1 | 1 | 2 |  | 0 | 1 | 2 | 2 | 3 |
| 6221032327 | 4.95 | 1.08 |  | 3.15 | 1.5 | 2 | 1 | 0 |  | 0 | 0 | 1 | 1 | 1 |
| 6221032328 | 3.45 | 0.84 |  | 1.87 | 2.65 | 0 | 1 | 1 |  | 0 | 0 | 3 | 3 | 3 |
| 6221032330 | 5.82 | 1.14 |  | 2.74 | 5.19 | 1 | 0 | 2 |  | 0 | 0 | 2 | 2 | 2 |
| 6221032337 | 3.42 | 0.88 |  | 2.06 | 1.14 | 0 | 1 | 2 |  | 0 | 1 | 2 | 2 | 2 |
| 6221032345 | 2.99 | 0.71 |  | 1.52 | 1.14 | 2 | 0 | 0 |  | 0 | 0 | 2 | 1 | 1 |
| 6221032349 | 2.37 | 0.78 |  | 1.14 | 0.75 | 0 | 0 | 0 |  | 0 | 0 | 2 | 1 | 1 |
| 6221032350 | 4.63 | 0.99 |  | 3.09 | 1.5 | 1 | 1 | 1 |  | 0 | 0 | 2 | 1 | 2 |
| 6221033102 | 3.91 | 0.76 |  | 1.8 | 3.31 | 2 | 1 | 0 |  | 0 | 1 | 3 | 2 | 3 |
| 6221033103 | 2.29 | 0.98 |  | 0.88 | 0.47 | 1 | 0 | 1 |  | 0 | 0 | 1 | 1 | 1 |
| 6221033104 | 3.16 | 0.81 |  | 1.76 | 1.3 | 0 | 1 | 1 |  | 0 | 0 | 3 | 2 | 2 |
| 6221033109 | 3.13 | 0.87 |  | 1.51 | 2.11 | 0 | 1 | 1 |  | 0 | 0 | 2 | 2 | 2 |
| 6221033111 | 4.28 | 0.84 |  | 2.15 | 2.81 | 0 | 0 | 1 |  | 0 | 0 | 2 | 2 | 1 |
| 6221033113 | 2.93 | 0.71 |  | 1.04 | 1.32 | 0 | 0 | 1 |  | 0 | 1 | 1 | 1 | 2 |
| 6221033116 | 1.92 | 0.65 |  | 0.76 | 0.97 | 1 | 0 | 1 |  | 0 | 0 | 2 | 1 | 2 |
| 6221033118 | 3.76 | 0.67 |  | 1.52 | 4.24 | 1 | 1 | 1 |  | 1 | 0 | 1 | 1 | 1 |
| 6221033119 | 3.76 | 1.03 |  | 2.18 | 1.06 | 2 | 1 | 1 |  | 0 | 0 | 2 | 1 | 2 |
| 6221033120 | 3.91 | 0.99 |  | 2.12 | 2.35 | 0 | 0 | 1 |  | 0 | 0 | 3 | 3 | 3 |
| 6221033121 | 2.35 | 0.79 |  | 0.94 | 1.01 | 1 | 0 | 0 |  | 0 | 0 | 2 | 1 | 1 |
| 6221033122 | 4.79 | 0.94 |  | 2.61 | 3.08 | 2 | 0 | 1 |  | 0 | 0 | 2 | 2 | 3 |
| 6221033129 | 3.72 | 0.76 |  | 1.87 | 3.12 | 0 | 1 | 1 |  | 0 | 1 | 3 | 3 | 3 |
| 6221033132 | 5.56 | 1.03 |  | 2.73 | 5.37 | 0 | 1 | 1 |  | 0 | 1 | 3 | 3 | 3 |
| 6221033133 | 3.08 | 0.9 |  | 1.57 | 1.36 | 1 | 1 | 1 |  | 0 | 1 | 3 | 3 | 3 |
| 6221033134 | 3.93 | 1.15 |  | 1.59 | 2.38 | 0 | 1 | 2 |  | 0 | 1 | 1 | 1 | 2 |
| 6221033136 | 3.34 | 0.69 |  | 1.83 | 1.47 | 1 | 0 | 2 |  | 0 | 0 | 2 | 2 | 1 |
| 6221033137 | 2.5 | 0.8 |  | 1.16 | 1.15 | 0 | 0 | 1 |  | 0 | 0 | 2 | 1 | 1 |
| 6221033141 | 2.69 | 0.91 |  | 1.32 | 0.66 | 1 | 1 | 0 |  | 0 | 1 | 2 | 2 | 1 |
| 6221033143 | 3.99 | 0.8 |  | 2.55 | 1.51 | 1 | 1 | 1 |  | 1 | 1 | 3 | 2 | 3 |
| 6221033144 | 1.82 | 0.43 |  | 0.88 | 0.77 | 1 | 0 | 0 |  | 0 | 0 | 1 | 1 | 2 |
| 6221033145 | 2.98 | 1.13 |  | 1.45 | 0.78 | 0 | 0 | 0 |  | 0 | 0 | 1 | 1 | 1 |
| 6221033148 | 4.46 | 0.87 |  | 2.42 | 2.2 | 2 | 1 | 0 |  | 0 | 0 | 3 | 3 | 3 |
| 6221033149 | 3.22 | 1.13 |  | 1.76 | 0.57 | 0 | 0 | 1 |  | 0 | 0 | 1 | 1 | 1 |
| 6221033150 | 2.64 | 0.76 |  | 1.36 | 1.54 | 0 | 1 | 0 |  | 1 | 0 | 3 | 3 | 3 |
| 6221033201 | 4.45 | 1.26 |  | 1.95 | 3.12 | 0 | 1 | 1 |  | 1 | 1 | 1 | 1 | 1 |
| 6221033202 | 4.16 | 1.06 |  | 2.56 | 1.01 | 1 | 1 | 1 |  | 0 | 0 | 3 | 3 | 3 |
| 6221033205 | 3.4 | 0.87 |  | 2.01 | 0.86 | 1 | 0 | 0 |  | 0 | 0 | 2 | 2 | 2 |
| 6221033208 | 3.15 | 1.25 |  | 1.37 | 1.33 | 0 | 1 | 1 |  | 1 | 1 | 2 | 2 | 2 |
| 6221033209 | 3.51 | 1.12 |  | 1.7 | 2.01 | 0 | 1 | 1 |  | 1 | 1 | 1 | 1 | 1 |
| 6221033211 | 4.07 | 1.77 |  | 1.63 | 0.51 | 1 | 0 | 1 |  | 0 | 0 | 1 | 1 | 1 |
| 6221033214 | 3.05 | 0.93 |  | 1.62 | 0.81 | 1 | 1 | 1 |  | 0 | 1 | 2 | 2 | 2 |
| 6221033215 | 3.93 | 1.13 |  | 2.23 | 1.11 | 0 | 0 | 1 |  | 0 | 0 | 2 | 2 | 3 |
| 6221033216 | 4.63 | 0.77 |  | 1.77 | 6.86 | 0 | 1 | 1 |  | 1 | 1 | 3 | 3 | 3 |
| 6221033218 | 3.26 | 0.68 |  | 1.52 | 2.51 | 1 | 0 | 0 |  | 0 | 0 | 1 | 1 | 1 |
| 6221033222 | 3.55 | 0.93 |  | 1.97 | 1.14 | 2 | 1 | 0 |  | 0 | 0 | 2 | 2 | 2 |
| 6221033223 | 2.59 | 0.82 |  | 1.4 | 0.55 | 0 | 1 | 1 |  | 0 | 0 | 2 | 1 | 1 |
| 6221033224 | 2.96 | 0.76 |  | 1.61 | 1.11 | 1 | 1 | 1 |  | 0 | 0 | 2 | 2 | 3 |
| 6221033225 | 2.95 | 0.56 |  | 1.91 | 0.87 | 0 | 1 | 0 |  | 0 | 1 | 2 | 1 | 1 |
| 6221033226 | 3.06 | 0.7 |  | 1.79 | 1.57 | 1 | 0 | 0 |  | 0 | 0 | 2 | 2 | 2 |
| 6221033228 | 3.03 | 1.13 |  | 1.57 | 0.68 | 0 | 0 | 1 |  | 0 | 0 | 2 | 2 | 1 |
| 6221033230 | 4.34 | 0.79 |  | 2.25 | 3.15 | 2 | 1 | 0 |  | 0 | 0 | 2 | 2 | 2 |
| 6221033231 | 3.98 | 1.07 |  | 1.89 | 2.15 | 1 | 1 | 0 |  | 0 | 1 | 3 | 2 | 2 |
| 6221033232 | 2.46 | 0.59 |  | 1.2 | 1.28 | 2 | 1 | 1 |  | 0 | 0 | 1 | 1 | 1 |
| 6221033234 | 4.89 | 0.95 |  | 2.71 | 2.61 | 0 | 0 | 0 |  | 0 | 0 | 3 | 2 | 3 |
| 6221033236 | 4.5 | 0.75 |  | 2.55 | 2.61 | 2 | 0 | 0 |  | 0 | 0 | 2 | 2 | 2 |
| 6221033239 | 3.22 | 0.77 |  | 1.28 | 3.77 | 0 | 1 | 1 |  | 0 | 0 | 3 | 3 | 3 |
| 6221033240 | 5.04 | 0.87 |  | 2.86 | 3.62 | 0 | 1 | 2 |  | 1 | 0 | 3 | 3 | 3 |
| 6221033241 | 5.23 | 1.23 |  | 2.56 | 3.06 | 1 | 0 | 1 |  | 0 | 0 | 3 | 3 | 3 |
| 6221033242 | 4.95 | 1.55 |  | 2.99 | 0.82 | 0 | 0 | 0 |  | 0 | 0 | 2 | 2 | 2 |
| 6221033244 | 3.04 | 1.01 |  | 1.63 | 0.76 | 0 | 0 | 1 |  | 0 | 0 | 3 | 3 | 3 |
| 6221033245 | 4.59 | 1.12 |  | 2.85 | 1.08 | 0 | 0 | 1 |  | 0 | 0 | 3 | 3 | 3 |
| 6221033248 | 4.46 | 0.97 |  | 2.45 | 2.24 | 1 | 1 | 1 |  | 0 | 1 | 2 | 1 | 2 |
| 6221033250 | 3.14 | 0.79 |  | 1.25 | 4.11 | 0 | 1 | 2 |  | 0 | 0 | 3 | 3 | 2 |
| 6221033301 | 5.72 | 1.45 |  | 3.48 | 1.5 | 0 | 0 | 2 |  | 0 | 0 | 3 | 3 | 3 |
| 6221033302 | 4.24 | 1.11 |  | 2.41 | 1.04 | 0 | 0 | 1 |  | 0 | 0 | 1 | 1 | 1 |
| 6221033303 | 5.41 | 1.57 |  | 3.09 | 1.02 | 2 | 0 | 0 |  | 0 | 0 | 1 | 1 | 1 |
| 6221033305 | 3.5 | 0.96 |  | 1.86 | 1.63 | 2 | 1 | 0 |  | 0 | 0 | 2 | 2 | 3 |
| 6221033309 | 3.42 | 0.8 |  | 1.84 | 2.59 | 1 | 0 | 1 |  | 0 | 0 | 3 | 3 | 3 |
| 6221033313 | 5.17 | 1.34 |  | 3.16 | 1.05 | 1 | 1 | 1 |  | 0 | 0 | 2 | 2 | 2 |
| 6221033315 | 3.39 | 0.94 |  | 1.65 | 1.11 | 2 | 0 | 0 |  | 0 | 0 | 3 | 3 | 3 |
| 6221033317 | 3.07 | 0.62 |  | 1.66 | 1.35 | 2 | 0 | 0 |  | 0 | 0 | 1 | 1 | 1 |
| 6221033318 | 4.36 | 0.77 |  | 2.53 | 2.98 | 1 | 1 | 2 |  | 0 | 1 | 3 | 3 | 2 |
| 6221033320 | 2.13 | 0.7 |  | 0.97 | 0.74 | 0 | 1 | 2 |  | 0 | 0 | 3 | 2 | 3 |
| 6221033322 | 2.7 | 0.51 |  | 1.22 | 1.96 | 1 | 1 | 2 |  | 0 | 1 | 3 | 3 | 3 |
| 6221033323 | 3.98 | 1.05 |  | 2.26 | 1.05 | 0 | 1 | 1 |  | 0 | 0 | 1 | 1 | 1 |
| 6221033324 | 4.52 | 1.1 |  | 2.01 | 3.32 | 1 | 0 | 0 |  | 0 | 0 | 2 | 2 | 2 |
| 6221033325 | 3.21 | 0.93 |  | 1.63 | 1 | 1 | 0 | 0 |  | 0 | 0 | 3 | 3 | 3 |
| 6221033328 | 3.07 | 0.76 |  | 1.94 | 0.77 | 2 | 1 | 1 |  | 0 | 0 | 1 | 1 | 1 |
| 6221033330 | 5.02 | 0.98 |  | 2.9 | 2.8 | 0 | 1 | 1 |  | 0 | 1 | 3 | 3 | 3 |
| 6221033332 | 3.14 | 0.58 |  | 2.07 | 0.9 | 2 | 1 | 1 |  | 0 | 0 | 1 | 1 | 1 |
| 6221033333 | 3.37 | 0.83 |  | 1.87 | 1.42 | 2 | 1 | 0 |  | 0 | 0 | 2 | 1 | 1 |
| 6221033335 | 3.74 | 0.76 |  | 2.16 | 1.69 | 0 | 0 | 0 |  | 0 | 0 | 3 | 3 | 3 |
| 6221033336 | 6.35 | 0.49 |  | 2.66 | 16.05 | 1 | 1 | 1 |  | 0 | 1 | 2 | 2 | 3 |
| 6221033337 | 4.65 | 0.76 |  | 1.99 | 4.04 | 2 | 0 | 0 |  | 0 | 0 | 1 | 1 | 2 |
| 6221033338 | 5.68 | 1.02 |  | 3.15 | 2.62 | 0 | 1 | 1 |  | 0 | 0 | 3 | 2 | 2 |
| 6221033339 | 4.15 | 0.76 |  | 1.52 | 6.32 | 0 | 1 | 1 |  | 0 | 1 | 1 | 1 | 1 |
| 6221033340 | 4.21 | 1.19 |  | 2.54 | 0.93 | 0 | 0 | 0 |  | 0 | 0 | 3 | 3 | 3 |
| 6221033341 | 4.09 | 0.53 |  | 1.84 | 5.27 | 1 | 0 | 0 |  | 0 | 0 | 3 | 3 | 3 |
| 6221033342 | 3.48 | 0.88 |  | 2.11 | 0.78 | 0 | 0 | 1 |  | 0 | 0 | 2 | 2 | 3 |
| 6221033343 | 3.08 | 0.68 |  | 1.73 | 1.01 | 1 | 1 | 1 |  | 0 | 1 | 3 | 3 | 3 |
| 6221033346 | 4.38 | 1.01 |  | 2.72 | 1.77 | 1 | 0 | 0 |  | 0 | 0 | 2 | 2 | 2 |
| 6221033348 | 2.78 | 0.64 |  | 1.74 | 0.93 | 0 | 0 | 2 |  | 0 | 0 | 2 | 2 | 2 |
| 6221033349 | 3.84 | 0.92 |  | 2.54 | 0.99 | 0 | 0 | 0 |  | 0 | 0 | 2 | 2 | 3 |
| 6221033350 | 2.89 | 0.83 |  | 1.81 | 0.66 | 1 | 0 | 1 |  | 0 | 0 | 2 | 2 | 2 |
| 6221034105 | 4.39 | 0.79 |  | 1.97 | 4.31 | 1 | 1 | 2 |  | 0 | 1 | 3 | 2 | 3 |
| 6221034106 | 3.97 | 1.23 |  | 2.1 | 1.21 | 1 | 1 | 2 |  | 1 | 0 | 2 | 2 | 2 |
| 6221034108 | 3.12 | 0.91 |  | 1.79 | 0.89 | 0 | 0 | 1 |  | 0 | 0 | 2 | 2 | 1 |
| 6221034109 | 4.85 | 0.97 |  | 1.94 | 8.05 | 0 | 1 | 2 |  | 0 | 0 | 2 | 2 | 2 |
| 6221034110 | 3.46 | 0.85 |  | 1.94 | 1.66 | 2 | 1 | 2 |  | 0 | 1 | 2 | 2 | 2 |
| 6221034112 | 3.33 | 0.75 |  | 1.59 | 2.97 | 0 | 1 | 2 |  | 0 | 1 | 2 | 2 | 2 |
| 6221034117 | 4.1 | 0.76 |  | 2.58 | 2.32 | 0 | 1 | 2 |  | 0 | 1 | 3 | 2 | 3 |
| 6221034118 | 3.22 | 0.68 |  | 1.58 | 2.4 | 1 | 1 | 2 |  | 1 | 1 | 3 | 2 | 2 |
| 6221034126 | 3.92 | 0.74 |  | 2.14 | 2.89 | 0 | 1 | 2 |  | 0 | 0 | 3 | 2 | 3 |
| 6221034132 | 4.37 | 1.14 |  | 2.78 | 1.24 | 0 | 0 | 2 |  | 0 | 0 | 2 | 2 | 1 |
| 6221034133 | 4.83 | 1.54 |  | 2.75 | 1.57 | 0 | 0 | 2 |  | 0 | 0 | 2 | 2 | 2 |
| 6221034136 | 7.09 | 0.81 |  | 3.01 | 9.21 | 0 | 1 | 2 |  | 1 | 1 | 3 | 2 | 3 |
| 6221034140 | 3.48 | 0.75 |  | 2.11 | 1.46 | 2 | 0 | 1 |  | 0 | 0 | 2 | 2 | 2 |
| 6221034142 | 3.4 | 0.56 |  | 1.95 | 2.02 | 0 | 1 | 2 |  | 1 | 1 | 1 | 1 | 2 |
| 6221034143 | 6.05 | 1.61 |  | 3.87 | 1.16 | 0 | 0 | 1 |  | 0 | 0 | 2 | 1 | 2 |
| 6221034144 | 3.64 | 0.72 |  | 2.17 | 1.35 | 2 | 1 | 1 |  | 0 | 0 | 2 | 2 | 2 |
| 6221034146 | 3.92 | 0.82 |  | 1.9 | 3.88 | 0 | 1 | 2 |  | 0 | 1 | 2 | 2 | 2 |
| 6221034201 | 4.06 | 1.05 |  | 2.06 | 1.49 | 2 | 0 | 0 |  | 0 | 0 | 2 | 2 | 3 |
| 6221034202 | 4.07 | 1.55 |  | 1.74 | 1.06 | 2 | 1 | 2 |  | 0 | 1 | 1 | 1 | 1 |
| 6221034204 | 2.45 | 0.86 |  | 1.23 | 0.88 | 0 | 0 | 1 |  | 0 | 0 | 2 | 2 | 2 |
| 6221034205 | 4.63 | 0.8 |  | 2.38 | 2.8 | 2 | 0 | 1 |  | 0 | 0 | 2 | 1 | 2 |
| 6221034208 | 3.58 | 0.95 |  | 2.08 | 1.6 | 0 | 1 | 1 |  | 0 | 0 | 2 | 1 | 1 |
| 6221034209 | 5.54 | 1.11 |  | 2.93 | 4.46 | 1 | 1 | 2 |  | 0 | 0 | 2 | 2 | 2 |
| 6221034211 | 5.82 | 0.99 |  | 3.49 | 3.62 | 0 | 1 | 1 |  | 0 | 0 | 3 | 3 | 2 |
| 6221034214 | 3.92 | 1.09 |  | 1.92 | 1.69 | 1 | 0 | 2 |  | 0 | 0 | 3 | 2 | 2 |
| 6221034215 | 3.76 | 0.85 |  | 1.95 | 2.51 | 2 | 0 | 0 |  | 0 | 0 | 3 | 3 | 3 |
| 6221034218 | 4.45 | 1 |  | 2.46 | 1.85 | 1 | 1 | 2 |  | 0 | 1 | 1 | 1 | 1 |
| 6221034219 | 3.24 | 0.9 |  | 1.75 | 1.49 | 1 | 0 | 1 |  | 0 | 0 | 3 | 3 | 3 |
| 6221034220 | 4.03 | 0.95 |  | 2.19 | 2.75 | 1 | 1 | 2 |  | 1 | 0 | 3 | 2 | 3 |
| 6221034222 | 5.14 | 0.88 |  | 2.9 | 3.02 | 1 | 0 | 2 |  | 0 | 0 | 2 | 1 | 2 |
| 6221034225 | 4.64 | 1.33 |  | 2.73 | 0.77 | 0 | 0 | 1 |  | 0 | 0 | 1 | 1 | 1 |
| 6221034226 | 4.04 | 1.11 |  | 2.45 | 1.18 | 0 | 1 | 1 |  | 0 | 0 | 3 | 2 | 3 |
| 6221034229 | 4.06 | 0.99 |  | 2.15 | 1.76 | 0 | 0 | 2 |  | 0 | 0 | 3 | 2 | 2 |
| 6221034230 | 4.34 | 1.45 |  | 2.35 | 0.86 | 1 | 0 | 2 |  | 0 | 0 | 2 | 2 | 1 |
| 6221034233 | 4.9 | 1.52 |  | 2.49 | 1.39 | 2 | 0 | 0 |  | 0 | 0 | 3 | 2 | 3 |
| 6221034234 | 3.13 | 0.82 |  | 1.3 | 2.53 | 1 | 1 | 1 |  | 0 | 1 | 1 | 1 | 1 |
| 6221034238 | 6.18 | 1.04 |  | 2.31 | 4.35 | 2 | 0 | 0 |  | 0 | 0 | 1 | 1 | 2 |
| 6221034244 | 5.18 | 1.15 |  | 2.87 | 3.44 | 2 | 0 | 0 |  | 0 | 0 | 3 | 3 | 3 |
| 6221034245 | 4.02 | 1.06 |  | 2.08 | 1.68 | 0 | 0 | 1 |  | 0 | 0 | 1 | 1 | 2 |
| 6221034246 | 3.78 | 1.37 |  | 1.9 | 0.73 | 1 | 1 | 1 |  | 1 | 1 | 2 | 2 | 2 |
| 6221034250 | 3.49 | 0.95 |  | 1.93 | 1.48 | 0 | 1 | 2 |  | 0 | 0 | 3 | 2 | 3 |
| 6221034302 | 5.77 | 1.37 |  | 3.54 | 2.29 | 0 | 0 | 2 |  | 0 | 0 | 3 | 3 | 3 |
| 6221034303 | 5.07 | 1.09 |  | 3.22 | 1.82 | 1 | 0 | 2 |  | 0 | 0 | 3 | 3 | 3 |
| 6221034304 | 5.49 | 1.19 |  | 2.81 | 2.92 | 1 | 0 | 2 |  | 0 | 0 | 3 | 3 | 2 |
| 6221034305 | 4.38 | 1.2 |  | 2.31 | 1.57 | 2 | 0 | 1 |  | 0 | 0 | 2 | 2 | 2 |
| 6221034306 | 3.56 | 0.96 |  | 2 | 1.74 | 1 | 1 | 2 |  | 0 | 1 | 3 | 2 | 2 |
| 6221034307 | 5.01 | 0.98 |  | 2.93 | 3.06 | 0 | 1 | 2 |  | 0 | 0 | 3 | 2 | 2 |
| 6221034309 | 3.25 | 0.76 |  | 1.66 | 2.08 | 1 | 1 | 2 |  | 0 | 0 | 2 | 1 | 1 |
| 6221034311 | 3.46 | 0.89 |  | 1.96 | 1.18 | 0 | 0 | 1 |  | 0 | 0 | 3 | 3 | 3 |
| 6221034313 | 3.68 | 0.86 |  | 1.98 | 2.52 | 1 | 0 | 2 |  | 0 | 0 | 3 | 2 | 3 |
| 6221034314 | 4.58 | 0.77 |  | 2.84 | 2.69 | 2 | 1 | 1 |  | 0 | 0 | 3 | 2 | 3 |
| 6221034315 | 4.05 | 0.66 |  | 2.59 | 1.57 | 1 | 1 | 2 |  | 1 | 1 | 2 | 2 | 2 |
| 6221034318 | 4.95 | 1.06 |  | 3.24 | 1.3 | 1 | 0 | 2 |  | 0 | 0 | 2 | 2 | 2 |
| 6221034319 | 4.55 | 0.83 |  | 2.65 | 2.61 | 1 | 0 | 1 |  | 0 | 0 | 2 | 2 | 2 |
| 6221034321 | 5.39 | 1.7 |  | 2.69 | 1.16 | 2 | 0 | 0 |  | 0 | 0 | 1 | 1 | 2 |
| 6221034322 | 5.18 | 1.14 |  | 3.62 | 1.13 | 1 | 1 | 2 |  | 0 | 1 | 2 | 2 | 2 |
| 6221034323 | 4.57 | 1.07 |  | 2.18 | 5.35 | 0 | 1 | 2 |  | 0 | 1 | 2 | 2 | 2 |
| 6221034324 | 4.95 | 1.01 |  | 2.95 | 2.51 | 1 | 0 | 1 |  | 0 | 0 | 3 | 3 | 3 |
| 6221034326 | 4.28 | 1.01 |  | 2.53 | 1.58 | 1 | 0 | 2 |  | 0 | 0 | 2 | 2 | 3 |
| 6221034327 | 2.93 | 0.73 |  | 1.62 | 1.4 | 0 | 0 | 1 |  | 0 | 0 | 3 | 3 | 3 |
| 6221034329 | 3.06 | 0.93 |  | 1.6 | 1.27 | 0 | 1 | 2 |  | 0 | 0 | 2 | 2 | 2 |
| 6221034330 | 5.25 | 1.15 |  | 2.64 | 4.76 | 0 | 1 | 2 |  | 0 | 1 | 3 | 3 | 3 |
| 6221034331 | 5.04 | 0.93 |  | 3.5 | 1.16 | 1 | 0 | 2 |  | 0 | 0 | 1 | 1 | 2 |
| 6221034333 | 4.64 | 0.71 |  | 2.41 | 4.02 | 0 | 1 | 2 |  | 0 | 1 | 2 | 2 | 2 |
| 6221034334 | 2.78 | 0.86 |  | 1.26 | 1.3 | 1 | 0 | 1 |  | 0 | 0 | 3 | 2 | 3 |
| 6221034335 | 3.93 | 0.85 |  | 2.24 | 1.78 | 1 | 0 | 1 |  | 0 | 0 | 3 | 3 | 3 |
| 6221034336 | 4.39 | 0.94 |  | 2.26 | 3.08 | 1 | 1 | 2 |  | 1 | 0 | 2 | 2 | 3 |
| 6221034337 | 3.94 | 0.99 |  | 2.13 | 1.48 | 2 | 0 | 0 |  | 0 | 0 | 3 | 2 | 3 |
| 6221034338 | 4.53 | 0.9 |  | 2.67 | 1.68 | 2 | 0 | 1 |  | 0 | 0 | 3 | 3 | 3 |
| 6221034339 | 3.39 | 0.72 |  | 1.75 | 3.41 | 1 | 1 | 2 |  | 0 | 1 | 3 | 2 | 2 |
| 6221034340 | 5.42 | 0.95 |  | 2.2 | 6.2 | 1 | 1 | 1 |  | 0 | 0 | 2 | 2 | 2 |
| 6221034341 | 3.79 | 1.09 |  | 1.99 | 1.02 | 2 | 0 | 0 |  | 0 | 0 | 2 | 2 | 2 |
| 6221034346 | 2.47 | 0.83 |  | 1.1 | 1.02 | 0 | 0 | 1 |  | 0 | 0 | 2 | 1 | 1 |
| 6221034347 | 2.64 | 0.73 |  | 1.48 | 0.84 | 1 | 1 | 2 |  | 0 | 0 | 3 | 3 | 1 |
| 6221034348 | 4.06 | 0.85 |  | 2.4 | 2.02 | 0 | 1 | 2 |  | 0 | 0 | 3 | 2 | 3 |
| 6221034350 | 3.02 | 0.6 |  | 1.73 | 2.02 | 1 | 1 | 2 |  | 0 | 1 | 3 | 3 | 3 |
| 6222011101 | 3.89 | 0.84 |  | 1.91 | 1.57 | 2 | 0 | 0 |  | 0 | 0 | 2 | 1 | 1 |
| 6222011103 | 4.34 | 0.86 |  | 2.26 | 2.62 | 2 | 0 | 1 |  | 0 | 0 | 1 | 1 | 2 |
| 6222011108 | 3.35 | 0.73 |  | 1.94 | 1.69 | 2 | 1 | 0 |  | 0 | 0 | 3 | 2 | 2 |
| 6222011109 | 3.52 | 1.19 |  | 1.76 | 0.87 | 0 | 1 | 1 |  | 1 | 1 | 1 | 1 | 1 |
| 6222011115 | 4.31 | 1 |  | 2.26 | 1.96 | 0 | 1 | 1 |  | 0 | 0 | 2 | 2 | 1 |
| 6222011117 | 4.32 | 0.78 |  | 2.15 | 5.75 | 1 | 1 | 0 |  | 0 | 0 | 2 | 2 | 1 |
| 6222011120 | 4.38 | 1.2 |  | 2.21 | 1.56 | 2 | 0 | 0 |  | 0 | 0 | 1 | 1 | 1 |
| 6222011121 | 3.37 | 0.49 |  | 1.79 | 2.8 | 1 | 1 | 1 |  | 1 | 0 | 2 | 2 | 3 |
| 6222011122 | 3.24 | 0.94 |  | 1.69 | 0.88 | 2 | 1 | 0 |  | 0 | 0 | 1 | 1 | 1 |
| 6222011124 | 4.42 | 1 |  | 2.52 | 2.86 | 0 | 1 | 1 |  | 0 | 1 | 3 | 2 | 1 |
| 6222011128 | 3.77 | 1.21 |  | 1.87 | 0.84 | 1 | 0 | 0 |  | 0 | 0 | 1 | 1 | 1 |
| 6222011129 | 3.23 | 0.76 |  | 1.74 | 1.78 | 1 | 1 | 1 |  | 0 | 0 | 1 | 1 | 1 |
| 6222011131 | 3.85 | 1.36 |  | 1.62 | 1.33 | 0 | 1 | 1 |  | 1 | 1 | 1 | 1 | 1 |
| 6222011132 | 3.78 | 0.8 |  | 2.04 | 1.64 | 2 | 1 | 0 |  | 0 | 0 | 1 | 1 | 1 |
| 6222011134 | 2.75 | 0.77 |  | 1.26 | 2.32 | 0 | 1 | 0 |  | 1 | 1 | 3 | 2 | 2 |
| 6222011136 | 3.65 | 1.25 |  | 1.48 | 1.67 | 1 | 0 | 0 |  | 0 | 0 | 1 | 1 | 1 |
| 6222011138 | 3.47 | 1.35 |  | 1.39 | 1.49 | 0 | 1 | 1 |  | 1 | 0 | 2 | 1 | 1 |
| 6222011139 | 2.78 | 0.92 |  | 1.3 | 0.69 | 2 | 1 | 0 |  | 0 | 1 | 1 | 1 | 1 |
| 6222011140 | 4.27 | 1.96 |  | 1.88 | 0.66 | 1 | 1 | 1 |  | 1 | 0 | 1 | 1 | 2 |
| 6222011141 | 4.49 | 1.03 |  | 2.39 | 1.92 | 2 | 1 | 0 |  | 0 | 0 | 2 | 2 | 2 |
| 6222011143 | 3.88 | 1.02 |  | 1.91 | 1.35 | 2 | 0 | 0 |  | 0 | 0 | 1 | 1 | 1 |
| 6222011145 | 3.55 | 1.18 |  | 1.86 | 1.09 | 0 | 0 | 2 |  | 0 | 0 | 1 | 1 | 1 |
| 6222011147 | 2.85 | 0.48 |  | 1.55 | 1.49 | 1 | 0 | 0 |  | 0 | 0 | 1 | 1 | 1 |
| 6222011148 | 4.34 | 1.51 |  | 2.04 | 2.14 | 2 | 1 | 1 |  | 1 | 0 | 3 | 2 | 3 |
| 6222011149 | 4.61 | 1.05 |  | 2.52 | 1.63 | 2 | 1 | 0 |  | 0 | 1 | 1 | 1 | 1 |
| 6222011150 | 3.95 | 1.01 |  | 2.19 | 1.6 | 2 | 1 | 0 |  | 0 | 0 | 1 | 1 | 1 |
| 6222011201 | 4.49 | 1.02 |  | 2.54 | 1.31 | 1 | 0 | 2 |  | 0 | 0 | 1 | 1 | 1 |
| 6222011204 | 2.88 | 1.02 |  | 1.26 | 0.68 | 0 | 1 | 0 |  | 0 | 1 | 1 | 1 | 2 |
| 6222011205 | 3.27 | 1 |  | 1.22 | 2.16 | 2 | 0 | 0 |  | 0 | 0 | 1 | 1 | 1 |
| 6222011207 | 2.25 | 0.67 |  | 1.03 | 0.79 | 1 | 1 | 0 |  | 0 | 1 | 1 | 1 | 2 |
| 6222011208 | 5.44 | 1.26 |  | 3.29 | 1.75 | 0 | 1 | 2 |  | 1 | 0 | 2 | 2 | 1 |
| 6222011211 | 3.42 | 0.71 |  | 1.68 | 2.41 | 0 | 1 | 2 |  | 1 | 1 | 3 | 3 | 3 |
| 6222011212 | 3.27 | 1 |  | 1.63 | 1.25 | 2 | 0 | 0 |  | 0 | 0 | 3 | 2 | 2 |
| 6222011213 | 4.9 | 1.65 |  | 2.45 | 1.01 | 1 | 0 | 1 |  | 0 | 0 | 1 | 1 | 1 |
| 6222011214 | 3.7 | 0.63 |  | 2.15 | 2.03 | 1 | 1 | 0 |  | 0 | 0 | 2 | 2 | 1 |
| 6222011223 | 4.81 | 1.06 |  | 2.82 | 1.64 | 0 | 1 | 1 |  | 1 | 0 | 2 | 2 | 2 |
| 6222011225 | 3.68 | 1.09 |  | 1.89 | 1.33 | 1 | 0 | 1 |  | 0 | 0 | 2 | 2 | 1 |
| 6222011228 | 3.97 | 0.91 |  | 2.32 | 2.2 | 0 | 1 | 1 |  | 0 | 0 | 2 | 2 | 1 |
| 6222011232 | 2.63 | 0.7 |  | 1.31 | 0.8 | 1 | 0 | 0 |  | 0 | 0 | 1 | 1 | 1 |
| 6222011238 | 3.12 | 1.27 |  | 1.35 | 0.91 | 1 | 0 | 1 |  | 0 | 0 | 2 | 2 | 2 |
| 6222011239 | 3.87 | 0.72 |  | 2.31 | 1.7 | 1 | 1 | 2 |  | 1 | 1 | 2 | 2 | 1 |
| 6222011248 | 2.9 | 1.2 |  | 0.91 | 0.59 | 2 | 0 | 0 |  | 0 | 0 | 1 | 1 | 1 |
| 6222011303 | 3.99 | 1.21 |  | 1.92 | 0.79 | 0 | 0 | 0 |  | 0 | 0 | 1 | 1 | 1 |
| 6222011315 | 2.54 | 0.63 |  | 1.43 | 0.62 | 2 | 1 | 0 |  | 0 | 1 | 2 | 2 | 1 |
| 6222011317 | 3.49 | 0.65 |  | 1.87 | 2.5 | 2 | 0 | 0 |  | 0 | 0 | 2 | 2 | 2 |
| 6222011326 | 3.19 | 0.67 |  | 1.4 | 2.87 | 1 | 0 | 1 |  | 0 | 0 | 3 | 3 | 2 |
| 6222011327 | 2.56 | 0.61 |  | 1.32 | 1.08 | 1 | 1 | 2 |  | 0 | 1 | 3 | 2 | 3 |
| 6222011330 | 2.78 | 0.78 |  | 1.3 | 1.12 | 1 | 0 | 2 |  | 0 | 0 | 2 | 1 | 2 |
| 6222011332 | 3.9 | 0.73 |  | 1.93 | 3.08 | 0 | 1 | 2 |  | 0 | 0 | 2 | 2 | 1 |
| 6222011334 | 3.01 | 0.75 |  | 1.57 | 1.22 | 0 | 0 | 1 |  | 0 | 0 | 2 | 1 | 2 |
| 6222011339 | 2.36 | 0.72 |  | 1 | 1.22 | 0 | 0 | 1 |  | 0 | 0 | 2 | 2 | 2 |
| 6222011348 | 3.51 | 1.23 |  | 1.59 | 1.02 | 2 | 0 | 0 |  | 0 | 0 | 3 | 2 | 3 |
| 6222012108 | 3.23 | 0.94 |  | 1.73 | 1.08 | 2 | 0 | 0 |  | 0 | 0 | 2 | 2 | 2 |
| 6222012111 | 3.99 | 0.98 |  | 2.17 | 1.75 | 0 | 1 | 1 |  | 1 | 0 | 3 | 2 | 2 |
| 6222012121 | 4.41 | 0.81 |  | 2.28 | 4.39 | 2 | 1 | 1 |  | 0 | 0 | 2 | 2 | 2 |
| 6222012122 | 4.12 | 0.84 |  | 2.43 | 1.02 | 2 | 0 | 0 |  | 0 | 0 | 3 | 3 | 3 |
| 6222012126 | 3.55 | 0.84 |  | 1.94 | 1.34 | 1 | 1 | 2 |  | 0 | 1 | 3 | 2 | 2 |
| 6222012128 | 4.7 | 0.91 |  | 2.41 | 3.07 | 2 | 0 | 0 |  | 0 | 0 | 1 | 1 | 2 |
| 6222012131 | 6.18 | 0.95 |  | 3.82 | 2.11 | 1 | 0 | 0 |  | 0 | 0 | 2 | 2 | 2 |
| 6222012137 | 3.3 | 0.94 |  | 1.52 | 1.26 | 2 | 0 | 0 |  | 0 | 0 | 1 | 1 | 1 |
| 6222012140 | 4.28 | 1.15 |  | 2.58 | 0.82 | 1 | 0 | 1 |  | 1 | 0 | 1 | 1 | 1 |
| 6222012141 | 4.27 | 1.12 |  | 2.16 | 1.8 | 1 | 1 | 2 |  | 1 | 0 | 2 | 2 | 2 |
| 6222012146 | 4.24 | 1.26 |  | 2.19 | 1.77 | 1 | 0 | 1 |  | 0 | 0 | 1 | 1 | 1 |
| 6222012147 | 1.96 | 0.67 |  | 0.67 | 1.89 | 1 | 0 | 0 |  | 0 | 0 | 3 | 3 | 3 |
| 6222012150 | 3.43 | 1 |  | 1.16 | 1.46 | 2 | 0 | 0 |  | 0 | 0 | 2 | 2 | 2 |
| 6222012202 | 4.41 | 1.06 |  | 2.67 | 1.93 | 1 | 0 | 0 |  | 0 | 0 | 2 | 2 | 1 |
| 6222012204 | 3.44 | 1.13 |  | 1.76 | 0.63 | 0 | 0 | 1 |  | 0 | 0 | 2 | 2 | 1 |
| 6222012206 | 5.12 | 0.93 |  | 4.06 | 1.69 | 2 | 0 | 0 |  | 0 | 0 | 2 | 2 | 1 |
| 6222012215 | 3.39 | 0.83 |  | 1.78 | 1.12 | 1 | 0 | 0 |  | 0 | 0 | 3 | 2 | 1 |
| 6222012232 | 3.21 | 1.12 |  | 1.44 | 0.75 | 2 | 1 | 0 |  | 0 | 1 | 2 | 2 | 2 |
| 6222012236 | 4.88 | 1.17 |  | 2.79 | 1.57 | 1 | 0 | 0 |  | 0 | 0 | 2 | 1 | 3 |
| 6222012240 | 4.82 | 1.08 |  | 2.82 | 1.33 | 1 | 1 | 1 |  | 1 | 1 | 3 | 2 | 2 |
| 6222012243 | 3.88 | 1.09 |  | 2.06 | 1.26 | 1 | 0 | 0 |  | 0 | 0 | 2 | 2 | 1 |
| 6222012245 | 5.1 | 1.47 |  | 2.23 | 2.15 | 0 | 1 | 1 |  | 0 | 1 | 2 | 1 | 1 |
| 6222012246 | 3.05 | 1.07 |  | 1.41 | 0.73 | 0 | 1 | 0 |  | 1 | 1 | 1 | 1 | 1 |
| 6222012250 | 3.99 | 1.17 |  | 1.99 | 1.03 | 2 | 1 | 1 |  | 0 | 0 | 1 | 1 | 1 |
| 6222012306 | 2.22 | 0.63 |  | 0.95 | 1.15 | 1 | 1 | 0 |  | 0 | 0 | 2 | 1 | 2 |
| 6222012309 | 6.49 | 1.24 |  | 3.24 | 3.71 | 1 | 0 | 0 |  | 0 | 0 | 1 | 1 | 2 |
| 6222012310 | 3.4 | 1.02 |  | 1.75 | 1.06 | 1 | 0 | 0 |  | 0 | 0 | 1 | 1 | 2 |
| 6222012311 | 1.02 | 0.28 |  | 0.34 | 0.61 | 2 | 0 | 0 |  | 0 | 0 | 2 | 2 | 2 |
| 6222012313 | 3.34 | 0.75 |  | 1.87 | 1.37 | 1 | 1 | 1 |  | 0 | 0 | 3 | 2 | 2 |
| 6222012317 | 3.44 | 0.78 |  | 1.94 | 1.73 | 1 | 0 | 0 |  | 0 | 0 | 2 | 2 | 2 |
| 6222012318 | 3.06 | 0.69 |  | 1.68 | 1.51 | 2 | 0 | 0 |  | 0 | 0 | 3 | 2 | 3 |
| 6222012324 | 2.91 | 0.68 |  | 1.53 | 1.5 | 0 | 0 | 1 |  | 0 | 0 | 2 | 2 | 2 |
| 6222012325 | 2.79 | 0.76 |  | 1.54 | 0.54 | 0 | 1 | 1 |  | 0 | 1 | 2 | 2 | 2 |
| 6222012326 | 3.99 | 0.99 |  | 2.11 | 2.15 | 0 | 0 | 0 |  | 0 | 0 | 2 | 1 | 2 |
| 6222012328 | 4.01 | 0.82 |  | 2.04 | 2.39 | 2 | 1 | 0 |  | 0 | 0 | 2 | 1 | 2 |
| 6222012329 | 2.08 | 0.5 |  | 1.06 | 1.35 | 0 | 1 | 1 |  | 0 | 1 | 2 | 2 | 3 |
| 6222012330 | 3.75 | 0.66 |  | 2.14 | 2.07 | 0 | 1 | 1 |  | 0 | 1 | 2 | 2 | 2 |
| 6222012332 | 2.34 | 0.51 |  | 0.98 | 2.01 | 1 | 1 | 0 |  | 1 | 0 | 2 | 2 | 2 |
| 6222012336 | 3.34 | 0.9 |  | 1.83 | 0.82 | 1 | 0 | 0 |  | 0 | 0 | 2 | 1 | 2 |
| 6222012337 | 2.03 | 0.49 |  | 0.95 | 0.81 | 1 | 1 | 1 |  | 0 | 0 | 1 | 1 | 1 |
| 6222012338 | 3.09 | 0.71 |  | 1.73 | 1.05 | 2 | 0 | 0 |  | 0 | 0 | 2 | 2 | 2 |
| 6222012344 | 4.12 | 0.69 |  | 1.96 | 3.15 | 1 | 0 | 0 |  | 0 | 0 | 3 | 2 | 2 |
| 6222012345 | 0.8 | 0.13 |  | 0.47 | 0.47 | 1 | 1 | 0 |  | 0 | 1 | 2 | 1 | 1 |
| 6222012347 | 4.24 | 0.99 |  | 2.38 | 1.68 | 1 | 1 | 0 |  | 0 | 0 | 1 | 1 | 1 |
| 6222013107 | 3.1 | 0.58 |  | 1.46 | 2.39 | 0 | 1 | 2 |  | 0 | 1 | 2 | 2 | 2 |
| 6222013108 | 3.92 | 0.68 |  | 2.16 | 1.81 | 2 | 0 | 0 |  | 0 | 0 | 2 | 1 | 2 |
| 6222013110 | 3.7 | 0.8 |  | 2.4 | 1.13 | 2 | 1 | 0 |  | 0 | 1 | 2 | 1 | 3 |
| 6222013112 | 2.58 | 0.47 |  | 1.22 | 1.69 | 1 | 1 | 1 |  | 0 | 0 | 3 | 2 | 3 |
| 6222013115 | 3.9 | 0.77 |  | 2.08 | 1.79 | 2 | 0 | 1 |  | 0 | 0 | 2 | 2 | 2 |
| 6222013117 | 4.07 | 0.68 |  | 1.67 | 3.58 | 2 | 0 | 0 |  | 0 | 0 | 1 | 1 | 2 |
| 6222013118 | 1.88 | 0.38 |  | 1.11 | 0.7 | 1 | 0 | 1 |  | 0 | 0 | 3 | 3 | 3 |
| 6222013121 | 6.13 | 1.11 |  | 3.01 | 4.17 | 2 | 1 | 1 |  | 0 | 0 | 1 | 1 | 2 |
| 6222013124 | 3.27 | 1.12 |  | 1.32 | 1.07 | 0 | 0 | 1 |  | 0 | 0 | 1 | 1 | 1 |
| 6222013125 | 3.79 | 0.64 |  | 2.03 | 3.48 | 2 | 1 | 0 |  | 0 | 1 | 3 | 3 | 3 |
| 6222013142 | 3.84 | 0.63 |  | 1.57 | 3.84 | 2 | 1 | 1 |  | 0 | 0 | 2 | 2 | 2 |
| 6222013203 | 2.85 | 0.69 |  | 1.44 | 1.34 | 1 | 1 | 0 |  | 0 | 1 | 1 | 1 | 1 |
| 6222013206 | 2.81 | 1.03 |  | 1.15 | 0.85 | 1 | 0 | 2 |  | 0 | 0 | 1 | 1 | 2 |
| 6222013209 | 3.51 | 0.78 |  | 1.08 | 3.45 | 1 | 1 | 1 |  | 1 | 0 | 2 | 2 | 2 |
| 6222013212 | 2.97 | 0.93 |  | 1.48 | 0.47 | 1 | 1 | 0 |  | 0 | 1 | 2 | 1 | 1 |
| 6222013218 | 3.38 | 0.91 |  | 1.69 | 0.87 | 2 | 0 | 0 |  | 0 | 0 | 2 | 2 | 2 |
| 6222013219 | 2.25 | 0.39 |  | 1.25 | 1.07 | 2 | 1 | 1 |  | 0 | 0 | 2 | 1 | 1 |
| 6222013220 | 3.64 | 0.57 |  | 2.64 | 1.39 | 1 | 0 | 0 |  | 0 | 0 | 2 | 2 | 2 |
| 6222013221 | 3.56 | 0.91 |  | 2.15 | 1.16 | 2 | 0 | 0 |  | 0 | 0 | 1 | 1 | 2 |
| 6222013227 | 3.38 | 0.91 |  | 1.38 | 1.88 | 1 | 1 | 0 |  | 0 | 0 | 1 | 1 | 1 |
| 6222013229 | 3.24 | 0.89 |  | 1.29 | 2.15 | 2 | 0 | 0 |  | 0 | 0 | 2 | 2 | 3 |
| 6222013232 | 2.35 | 0.8 |  | 1.09 | 0.63 | 0 | 1 | 0 |  | 0 | 0 | 1 | 1 | 1 |
| 6222013233 | 1.96 | 0.43 |  | 1.01 | 1.02 | 1 | 1 | 1 |  | 0 | 1 | 3 | 2 | 2 |
| 6222013241 | 3.5 | 0.5 |  | 1.48 | 4.09 | 1 | 0 | 0 |  | 0 | 0 | 2 | 2 | 2 |
| 6222013242 | 1.26 | 0.43 |  | 0.5 | 0.39 | 2 | 1 | 0 |  | 0 | 1 | 1 | 1 | 1 |
| 6222013302 | 3.68 | 1.03 |  | 1.7 | 2.26 | 1 | 0 | 0 |  | 0 | 0 | 1 | 1 | 1 |
| 6222013303 | 4.01 | 1.06 |  | 1.64 | 1.73 | 2 | 0 | 1 |  | 0 | 0 | 1 | 1 | 1 |
| 6222013304 | 2.9 | 0.95 |  | 1.19 | 0.98 | 0 | 0 | 1 |  | 0 | 0 | 2 | 1 | 2 |
| 6222013306 | 2.58 | 0.45 |  | 1.12 | 0.93 | 1 | 0 | 0 |  | 0 | 1 | 1 | 1 | 1 |
| 6222013308 | 2.71 | 0.65 |  | 1.42 | 0.74 | 2 | 1 | 1 |  | 1 | 0 | 1 | 1 | 2 |
| 6222013310 | 2.54 | 0.84 |  | 1.13 | 1 | 1 | 0 | 0 |  | 0 | 0 | 2 | 2 | 2 |
| 6222013311 | 5.12 | 0.83 |  | 2.39 | 5.38 | 1 | 0 | 0 |  | 0 | 0 | 3 | 2 | 3 |
| 6222013312 | 2.96 | 0.72 |  | 1.68 | 0.59 | 0 | 0 | 0 |  | 0 | 0 | 3 | 2 | 3 |
| 6222013315 | 3.39 | 0.77 |  | 1.93 | 1.26 | 0 | 1 | 2 |  | 1 | 1 | 2 | 2 | 2 |
| 6222013317 | 1.03 | 0.32 |  | 0.48 | 0.51 | 1 | 1 | 1 |  | 1 | 1 | 2 | 1 | 2 |
| 6222013318 | 3.59 | 0.46 |  | 1.21 | 1.55 | 1 | 0 | 0 |  | 0 | 0 | 2 | 2 | 2 |
| 6222013319 | 3.13 | 0.66 |  | 1.38 | 1.83 | 2 | 0 | 0 |  | 0 | 0 | 1 | 1 | 2 |
| 6222013321 | 2.92 | 1.05 |  | 1.06 | 0.82 | 0 | 1 | 1 |  | 0 | 0 | 1 | 1 | 1 |
| 6222013322 | 2.47 | 0.77 |  | 1.07 | 1.13 | 0 | 0 | 0 |  | 0 | 0 | 1 | 1 | 1 |
| 6222013323 | 1.95 | 0.5 |  | 0.86 | 1.15 | 0 | 1 | 1 |  | 1 | 1 | 2 | 2 | 2 |
| 6222013325 | 2.44 | 0.7 |  | 1.14 | 0.84 | 1 | 1 | 1 |  | 0 | 1 | 3 | 3 | 1 |
| 6222013327 | 3.19 | 0.99 |  | 1.39 | 0.93 | 2 | 0 | 0 |  | 0 | 0 | 1 | 1 | 1 |
| 6222013329 | 3.55 | 1.23 |  | 1.46 | 1.31 | 2 | 0 | 0 |  | 0 | 0 | 1 | 1 | 1 |
| 6222013333 | 4.69 | 0.56 |  | 2.24 | 6.48 | 0 | 1 | 0 |  | 0 | 0 | 3 | 2 | 2 |
| 6222013335 | 2.99 | 0.65 |  | 1.45 | 1.66 | 1 | 0 | 0 |  | 0 | 0 | 1 | 1 | 1 |
| 6222013338 | 2.93 | 0.93 |  | 1.18 | 1.43 | 2 | 0 | 0 |  | 0 | 0 | 1 | 1 | 1 |
| 6222013339 | 2.78 | 0.79 |  | 1.36 | 0.69 | 0 | 1 | 1 |  | 0 | 1 | 3 | 2 | 2 |
| 6222013340 | 3.43 | 0.49 |  | 1.53 | 3.74 | 2 | 1 | 1 |  | 1 | 0 | 2 | 1 | 2 |
| 6222013344 | 3.42 | 0.87 |  | 1.87 | 1.32 | 1 | 0 | 0 |  | 0 | 0 | 2 | 1 | 2 |
| 6222013348 | 3.3 | 0.71 |  | 1.59 | 2.09 | 1 | 1 | 2 |  | 0 | 0 | 2 | 2 | 1 |
| 6222013350 | 7.17 | 1.24 |  | 2.76 | 3.62 | 2 | 0 | 0 |  | 0 | 0 | 2 | 2 | 2 |
| 6222014101 | 3.98 | 1.36 |  | 1.92 | 0.79 | 0 | 1 | 1 |  | 1 | 0 | 2 | 2 | 1 |
| 6222014107 | 4.27 | 0.98 |  | 1.62 | 5.57 | 0 | 1 | 1 |  | 0 | 1 | 1 | 1 | 1 |
| 6222014109 | 4 | 1.13 |  | 1.99 | 1.23 | 2 | 1 | 0 |  | 0 | 1 | 1 | 1 | 1 |
| 6222014112 | 6.81 | 1.2 |  | 3.39 | 6.42 | 0 | 0 | 0 |  | 0 | 0 | 3 | 3 | 3 |
| 6222014114 | 4.46 | 0.96 |  | 2.85 | 1.01 | 0 | 1 | 2 |  | 1 | 1 | 2 | 2 | 2 |
| 6222014118 | 6.5 | 1.31 |  | 3.24 | 4.79 | 1 | 0 | 0 |  | 0 | 0 | 2 | 2 | 1 |
| 6222014124 | 5.35 | 1.19 |  | 2.96 | 1.69 | 2 | 0 | 0 |  | 0 | 0 | 2 | 1 | 2 |
| 6222014128 | 4.19 | 1.34 |  | 2.1 | 1.22 | 0 | 0 | 1 |  | 0 | 0 | 1 | 1 | 1 |
| 6222014131 | 3.54 | 0.88 |  | 1.76 | 2.18 | 0 | 1 | 1 |  | 0 | 0 | 3 | 3 | 3 |
| 6222014138 | 4.18 | 1.12 |  | 2.18 | 1.59 | 0 | 1 | 1 |  | 0 | 1 | 1 | 1 | 1 |
| 6222014140 | 3.48 | 0.79 |  | 1.51 | 3.27 | 1 | 1 | 0 |  | 0 | 0 | 2 | 2 | 1 |
| 6222014148 | 3.16 | 1.07 |  | 1.37 | 1.56 | 0 | 0 | 1 |  | 0 | 0 | 3 | 2 | 3 |
| 6222014201 | 3.61 | 0.71 |  | 2.38 | 0.68 | 1 | 0 | 2 |  | 0 | 0 | 1 | 1 | 1 |
| 6222014211 | 5.06 | 0.95 |  | 2.31 | 3.99 | 0 | 1 | 0 |  | 1 | 0 | 1 | 1 | 1 |
| 6222014213 | 3.44 | 1.37 |  | 1.28 | 0.99 | 1 | 1 | 1 |  | 0 | 1 | 1 | 1 | 1 |
| 6222014214 | 4.16 | 1.35 |  | 2 | 1.16 | 2 | 0 | 0 |  | 0 | 0 | 1 | 1 | 1 |
| 6222014215 | 3.39 | 0.57 |  | 1.9 | 2.02 | 0 | 0 | 1 |  | 0 | 0 | 1 | 1 | 1 |
| 6222014216 | 2.63 | 0.41 |  | 1.31 | 2.31 | 1 | 0 | 0 |  | 0 | 0 | 1 | 1 | 1 |
| 6222014218 | 2.69 | 1.29 |  | 0.67 | 0.78 | 0 | 0 | 0 |  | 0 | 0 | 1 | 1 | 1 |
| 6222014219 | 4.7 | 0.61 |  | 2.47 | 7.87 | 0 | 1 | 2 |  | 0 | 0 | 1 | 1 | 1 |
| 6222014221 | 2.97 | 0.74 |  | 1.43 | 2.2 | 0 | 1 | 2 |  | 0 | 1 | 2 | 2 | 2 |
| 6222014224 | 2.41 | 0.54 |  | 1.34 | 0.89 | 1 | 0 | 1 |  | 0 | 0 | 1 | 1 | 2 |
| 6222014225 | 4.07 | 0.98 |  | 2.16 | 1.27 | 2 | 0 | 1 |  | 0 | 0 | 1 | 1 | 1 |
| 6222014226 | 5.16 | 1.13 |  | 2.54 | 3.58 | 0 | 1 | 2 |  | 0 | 0 | 1 | 1 | 2 |
| 6222014227 | 3.12 | 0.86 |  | 1.78 | 0.71 | 1 | 1 | 2 |  | 0 | 1 | 1 | 1 | 2 |
| 6222014237 | 3.41 | 1.04 |  | 1.6 | 0.96 | 2 | 0 | 0 |  | 0 | 0 | 2 | 1 | 2 |
| 6222014243 | 2.2 | 0.44 |  | 1.35 | 1.29 | 1 | 1 | 1 |  | 0 | 0 | 1 | 1 | 2 |
| 6222014308 | 4.04 | 1 |  | 1.9 | 2.61 | 0 | 0 | 1 |  | 0 | 0 | 1 | 1 | 1 |
| 6222014310 | 3.48 | 1.01 |  | 1.79 | 0.97 | 1 | 1 | 0 |  | 0 | 0 | 1 | 1 | 1 |
| 6222014314 | 2.51 | 0.78 |  | 1.22 | 0.71 | 0 | 0 | 2 |  | 0 | 0 | 2 | 1 | 1 |
| 6222014315 | 4.64 | 1.35 |  | 2.55 | 1.02 | 0 | 1 | 2 |  | 0 | 0 | 1 | 1 | 1 |
| 6222014317 | 2.81 | 0.64 |  | 1.17 | 3.04 | 0 | 1 | 2 |  | 1 | 0 | 2 | 2 | 1 |
| 6222014318 | 5.16 | 1.01 |  | 2.43 | 3.18 | 1 | 1 | 1 |  | 0 | 1 | 2 | 2 | 2 |
| 6222014321 | 3.81 | 0.91 |  | 1.64 | 4.11 | 1 | 0 | 0 |  | 0 | 0 | 2 | 2 | 1 |
| 6222014327 | 3.31 | 0.74 |  | 1.66 | 2.81 | 0 | 1 | 1 |  | 0 | 1 | 2 | 1 | 2 |
| 6222014328 | 4.2 | 1.03 |  | 2.03 | 2.18 | 1 | 0 | 1 |  | 0 | 0 | 3 | 2 | 3 |
| 6222014333 | 3.55 | 0.69 |  | 1.65 | 3.35 | 1 | 1 | 0 |  | 0 | 0 | 3 | 2 | 2 |
| 6222014335 | 3.76 | 0.93 |  | 2.03 | 3.27 | 2 | 1 | 0 |  | 0 | 1 | 2 | 1 | 1 |
| 6222014341 | 4.96 | 1.24 |  | 2.76 | 1.42 | 1 | 1 | 0 |  | 0 | 0 | 2 | 2 | 1 |
| 6230211102 | 2.78 | 0.67 |  | 1.64 | 1.16 | 1 | 0 | 2 |  | 0 | 0 | 1 | 1 | 1 |
| 6230211113 | 3.6 | 0.84 |  | 2.38 | 1.13 | 2 | 1 | 2 |  | 0 | 1 | 1 | 1 | 2 |
| 6230211115 | 1.97 | 0.47 |  | 1.03 | 1.23 | 2 | 0 | 0 |  | 0 | 0 | 1 | 1 | 1 |
| 6230211117 | 2.6 | 0.75 |  | 1.68 | 0.8 | 0 | 0 | 0 |  | 0 | 0 | 3 | 2 | 3 |
| 6230211123 | 3.82 | 0.83 |  | 2.12 | 2.57 | 1 | 0 | 0 |  | 0 | 0 | 2 | 2 | 3 |
| 6230211125 | 2.92 | 0.74 |  | 1.96 | 0.77 | 1 | 0 | 0 |  | 0 | 0 | 1 | 1 | 2 |
| 6230211127 | 2.13 | 0.75 |  | 0.92 | 0.82 | 0 | 0 | 1 |  | 0 | 0 | 1 | 1 | 2 |
| 6230211129 | 2.05 | 0.47 |  | 1.19 | 1.18 | 1 | 1 | 1 |  | 0 | 0 | 1 | 1 | 1 |
| 6230211134 | 2.57 | 0.71 |  | 1.13 | 2.32 | 1 | 1 | 2 |  | 0 | 0 | 3 | 2 | 3 |
| 6230211142 | 3.98 | 1 |  | 2.32 | 1.99 | 2 | 0 | 0 |  | 0 | 0 | 2 | 2 | 2 |
| 6230211144 | 2.06 | 0.73 |  | 0.87 | 1.13 | 0 | 0 | 0 |  | 0 | 0 | 1 | 1 | 1 |
| 6230211146 | 3.44 | 0.94 |  | 1.91 | 1.36 | 1 | 0 | 0 |  | 0 | 0 | 2 | 2 | 2 |
| 6230211147 | 4.2 | 0.85 |  | 2.72 | 2.01 | 1 | 0 | 0 |  | 0 | 0 | 2 | 2 | 2 |
| 6230211149 | 2.66 | 0.67 |  | 1.59 | 0.99 | 0 | 1 | 1 |  | 0 | 1 | 1 | 1 | 1 |
| 6230211202 | 4.53 | 1.16 |  | 2.61 | 1.36 | 1 | 0 | 0 |  | 0 | 0 | 2 | 1 | 1 |
| 6230211204 | 5.17 | 1.17 |  | 2.89 | 3.25 | 0 | 1 | 2 |  | 0 | 1 | 2 | 2 | 2 |
| 6230211206 | 2.95 | 1.12 |  | 1.46 | 0.64 | 1 | 1 | 0 |  | 0 | 1 | 1 | 1 | 1 |
| 6230211207 | 3.39 | 1.06 |  | 1.85 | 0.96 | 0 | 0 | 0 |  | 0 | 0 | 3 | 2 | 2 |
| 6230211210 | 3.19 | 0.92 |  | 1.77 | 0.91 | 1 | 1 | 0 |  | 0 | 1 | 1 | 1 | 1 |
| 6230211211 | 3.56 | 1.1 |  | 1.65 | 1.24 | 2 | 1 | 1 |  | 0 | 0 | 1 | 1 | 1 |
| 6230211213 | 4.29 | 1.36 |  | 1.98 | 1.37 | 0 | 0 | 0 |  | 0 | 0 | 2 | 2 | 2 |
| 6230211214 | 3.67 | 0.66 |  | 2.17 | 2.63 | 2 | 0 | 0 |  | 0 | 0 | 2 | 2 | 2 |
| 6230211219 | 2.95 | 0.75 |  | 1.61 | 1.14 | 2 | 0 | 0 |  | 0 | 0 | 3 | 3 | 3 |
| 6230211221 | 3.07 | 1.02 |  | 1.7 | 0.63 | 2 | 1 | 0 |  | 0 | 1 | 1 | 1 | 1 |
| 6230211226 | 3.69 | 0.79 |  | 1.88 | 4.04 | 1 | 1 | 1 |  | 0 | 1 | 1 | 1 | 1 |
| 6230211229 | 2.49 | 0.98 |  | 1.18 | 0.85 | 0 | 0 | 0 |  | 0 | 0 | 1 | 1 | 1 |
| 6230211231 | 2.44 | 0.81 |  | 1.23 | 0.53 | 0 | 1 | 0 |  | 0 | 1 | 1 | 1 | 1 |
| 6230211232 | 2.95 | 0.98 |  | 1.41 | 0.72 | 2 | 0 | 0 |  | 0 | 0 | 1 | 1 | 1 |
| 6230211234 | 3.38 | 1.09 |  | 1.37 | 2.63 | 1 | 1 | 0 |  | 0 | 1 | 1 | 1 | 1 |
| 6230211235 | 2.51 | 1.04 |  | 0.84 | 0.99 | 1 | 0 | 1 |  | 0 | 0 | 1 | 1 | 1 |
| 6230211238 | 2.18 | 0.76 |  | 0.99 | 0.89 | 0 | 1 | 0 |  | 0 | 1 | 2 | 2 | 2 |
| 6230211241 | 3.5 | 1.2 |  | 1.81 | 0.75 | 1 | 0 | 0 |  | 0 | 0 | 1 | 1 | 1 |
| 6230211242 | 4.14 | 0.99 |  | 2.42 | 2.3 | 2 | 0 | 0 |  | 0 | 0 | 1 | 1 | 1 |
| 6230211247 | 3.3 | 0.76 |  | 1.92 | 1.72 | 0 | 1 | 1 |  | 0 | 1 | 1 | 1 | 1 |
| 6230211251 | 2.9 | 0.95 |  | 1.59 | 1.01 | 2 | 0 | 0 |  | 0 | 0 | 1 | 1 | 2 |
| 6230211303 | 3.13 | 1.04 |  | 1.81 | 0.75 | 2 | 1 | 0 |  | 0 | 0 | 1 | 1 | 1 |
| 6230211308 | 2.27 | 0.63 |  | 1.14 | 1.38 | 2 | 0 | 0 |  | 0 | 0 | 2 | 1 | 2 |
| 6230211309 | 4.59 | 0.83 |  | 2.18 | 4 | 2 | 0 | 0 |  | 0 | 0 | 1 | 1 | 1 |
| 6230211312 | 3.21 | 0.93 |  | 1.87 | 1.09 | 1 | 1 | 0 |  | 0 | 1 | 1 | 1 | 1 |
| 6230211314 | 4.87 | 0.93 |  | 2.95 | 2.66 | 2 | 0 | 0 |  | 0 | 0 | 1 | 1 | 1 |
| 6230211315 | 3.77 | 0.87 |  | 2.32 | 1.93 | 1 | 0 | 0 |  | 0 | 0 | 1 | 1 | 1 |
| 6230211316 | 3.71 | 1.36 |  | 1.63 | 1.43 | 1 | 1 | 1 |  | 0 | 1 | 1 | 1 | 1 |
| 6230211320 | 5.34 | 0.91 |  | 3.65 | 1.8 | 2 | 1 | 0 |  | 0 | 0 | 1 | 1 | 1 |
| 6230211321 | 2 | 0.58 |  | 1 | 1.08 | 1 | 0 | 0 |  | 0 | 0 | 2 | 2 | 2 |
| 6230211324 | 3.08 | 1 |  | 1.62 | 1.04 | 1 | 1 | 0 |  | 0 | 1 | 1 | 1 | 1 |
| 6230211325 | 3.8 | 1 |  | 2.22 | 1.22 | 1 | 1 | 1 |  | 0 | 1 | 1 | 1 | 1 |
| 6230211329 | 3.27 | 0.71 |  | 1.92 | 1.42 | 1 | 0 | 0 |  | 0 | 0 | 2 | 2 | 2 |
| 6230211332 | 5.9 | 1.12 |  | 3.39 | 3.75 | 0 | 0 | 0 |  | 0 | 0 | 2 | 2 | 3 |
| 6230211335 | 2.4 | 0.89 |  | 1.13 | 0.89 | 0 | 1 | 1 |  | 0 | 1 | 1 | 1 | 1 |
| 6230211336 | 3.65 | 0.81 |  | 1.96 | 2.92 | 1 | 0 | 1 |  | 0 | 0 | 2 | 1 | 2 |
| 6230211340 | 2.99 | 0.56 |  | 1.89 | 1.76 | 2 | 0 | 0 |  | 0 | 0 | 2 | 2 | 3 |
| 6230211343 | 4.35 | 0.92 |  | 2.06 | 4.85 | 0 | 0 | 0 |  | 0 | 0 | 2 | 1 | 2 |
| 6230211344 | 2.98 | 0.81 |  | 1.74 | 1.17 | 1 | 0 | 0 |  | 0 | 0 | 2 | 2 | 3 |
| 6230211347 | 2.4 | 0.72 |  | 1.41 | 0.75 | 0 | 0 | 0 |  | 0 | 0 | 1 | 1 | 1 |
| 6230211349 | 4.3 | 1.03 |  | 2.76 | 1.34 | 0 | 1 | 1 |  | 0 | 0 | 1 | 1 | 1 |
| 6230212103 | 4.56 | 0.89 |  | 2.04 | 4.77 | 1 | 0 | 0 |  | 0 | 0 | 2 | 2 | 2 |
| 6230212107 | 3.21 | 1.15 |  | 1.57 | 0.88 | 1 | 1 | 0 |  | 0 | 0 | 1 | 1 | 1 |
| 6230212114 | 3.76 | 0.7 |  | 1.36 | 5.5 | 1 | 0 | 0 |  | 0 | 0 | 3 | 2 | 3 |
| 6230212117 | 3.04 | 0.65 |  | 1.73 | 1.74 | 2 | 0 | 0 |  | 0 | 0 | 2 | 2 | 3 |
| 6230212121 | 3.97 | 1.36 |  | 2.01 | 0.95 | 2 | 0 | 0 |  | 0 | 0 | 1 | 1 | 1 |
| 6230212122 | 3.71 | 0.7 |  | 2.37 | 1.58 | 0 | 1 | 1 |  | 0 | 1 | 1 | 1 | 1 |
| 6230212127 | 3.69 | 0.58 |  | 1.89 | 3.21 | 2 | 1 | 1 |  | 0 | 1 | 2 | 2 | 1 |
| 6230212129 | 3.37 | 1.12 |  | 1.68 | 1.26 | 2 | 0 | 0 |  | 0 | 0 | 1 | 1 | 2 |
| 6230212132 | 3.28 | 1.19 |  | 1.35 | 1.16 | 2 | 0 | 0 |  | 0 | 0 | 1 | 1 | 2 |
| 6230212133 | 2.6 | 0.98 |  | 1 | 1.45 | 2 | 1 | 0 |  | 0 | 0 | 2 | 2 | 3 |
| 6230212134 | 3.58 | 1.12 |  | 1.8 | 1.64 | 1 | 0 | 0 |  | 0 | 0 | 1 | 1 | 3 |
| 6230212141 | 3.46 | 0.96 |  | 2.21 | 1 | 2 | 0 | 0 |  | 0 | 0 | 3 | 2 | 3 |
| 6230212143 | 3.47 | 1.13 |  | 1.7 | 1.72 | 2 | 0 | 0 |  | 0 | 0 | 2 | 1 | 1 |
| 6230212146 | 4.01 | 1.05 |  | 2.55 | 1.08 | 0 | 1 | 1 |  | 0 | 0 | 2 | 2 | 2 |
| 6230212153 | 4.33 | 0.83 |  | 2.13 | 3.36 | 2 | 0 | 0 |  | 0 | 0 | 3 | 3 | 3 |
| 6230212204 | 3.25 | 1.01 |  | 2.17 | 0.73 | 0 | 1 | 0 |  | 0 | 1 | 1 | 1 | 1 |
| 6230212212 | 3.89 | 0.95 |  | 2.45 | 1.11 | 1 | 1 | 0 |  | 0 | 1 | 1 | 1 | 1 |
| 6230212223 | 3.85 | 1.01 |  | 2.61 | 0.86 | 2 | 0 | 0 |  | 0 | 0 | 2 | 2 | 2 |
| 6230212225 | 4.26 | 1.01 |  | 2.92 | 1.12 | 0 | 0 | 0 |  | 0 | 0 | 1 | 1 | 1 |
| 6230212231 | 3.6 | 1.13 |  | 2.1 | 1.07 | 0 | 0 | 0 |  | 0 | 0 | 1 | 1 | 2 |
| 6230212241 | 3.09 | 0.78 |  | 1 | 2.81 | 0 | 0 | 0 |  | 0 | 0 | 3 | 2 | 3 |
| 6230212243 | 3.61 | 1.44 |  | 1.64 | 1.1 | 0 | 0 | 0 |  | 0 | 0 | 1 | 1 | 1 |
| 6230212303 | 4.04 | 0.94 |  | 2.67 | 1.43 | 0 | 1 | 1 |  | 0 | 0 | 1 | 1 | 2 |
| 6230212304 | 3.19 | 1.16 |  | 1.67 | 0.81 | 1 | 1 | 0 |  | 0 | 1 | 1 | 1 | 1 |
| 6230212305 | 3.04 | 1.07 |  | 1.46 | 0.96 | 1 | 0 | 0 |  | 0 | 0 | 1 | 1 | 1 |
| 6230212306 | 3.66 | 1.08 |  | 1.97 | 1.32 | 0 | 0 | 1 |  | 0 | 0 | 1 | 1 | 1 |
| 6230212307 | 3.91 | 0.96 |  | 2.12 | 1.95 | 1 | 0 | 0 |  | 0 | 0 | 2 | 1 | 3 |
| 6230212309 | 3.56 | 0.98 |  | 1.99 | 1.39 | 2 | 0 | 0 |  | 0 | 0 | 1 | 1 | 2 |
| 6230212313 | 3.08 | 0.84 |  | 1.67 | 1.74 | 0 | 0 | 0 |  | 0 | 0 | 1 | 1 | 2 |
| 6230212314 | 3.93 | 0.86 |  | 2.4 | 1.97 | 1 | 1 | 0 |  | 0 | 1 | 1 | 1 | 3 |
| 6230212316 | 4.9 | 1.06 |  | 2.61 | 2.99 | 2 | 0 | 0 |  | 0 | 0 | 1 | 1 | 1 |
| 6230212322 | 4.08 | 1.34 |  | 2.31 | 0.84 | 2 | 0 | 0 |  | 0 | 0 | 1 | 1 | 2 |
| 6230212332 | 3.37 | 0.88 |  | 1.98 | 1.19 | 1 | 0 | 0 |  | 0 | 0 | 2 | 2 | 2 |
| 6230212334 | 3.67 | 1.09 |  | 2.18 | 0.93 | 1 | 0 | 0 |  | 0 | 0 | 1 | 1 | 1 |
| 6230212340 | 4.19 | 1.42 |  | 1.95 | 2.11 | 2 | 0 | 0 |  | 0 | 0 | 1 | 1 | 1 |
| 6230212349 | 4.85 | 1.14 |  | 2.99 | 1.92 | 0 | 0 | 0 |  | 0 | 0 | 1 | 1 | 2 |
| 6230213110 | 3.78 | 0.83 |  | 2 | 2.86 | 2 | 0 | 0 |  | 0 | 0 | 3 | 3 | 3 |
| 6230213112 | 2.9 | 1.04 |  | 1.48 | 1.04 | 1 | 0 | 0 |  | 0 | 0 | 1 | 1 | 1 |
| 6230213116 | 3.92 | 1.08 |  | 2.62 | 0.82 | 1 | 1 | 0 |  | 0 | 1 | 1 | 1 | 1 |
| 6230213119 | 1.82 | 0.63 |  | 1.01 | 0.46 | 0 | 1 | 0 |  | 0 | 0 | 1 | 1 | 1 |
| 6230213121 | 2.06 | 0.62 |  | 1.04 | 1.07 | 2 | 0 | 0 |  | 0 | 0 | 1 | 1 | 2 |
| 6230213123 | 3.16 | 0.96 |  | 1.63 | 1.27 | 1 | 0 | 0 |  | 0 | 0 | 1 | 1 | 1 |
| 6230213124 | 3.65 | 1.09 |  | 2.3 | 0.88 | 2 | 1 | 1 |  | 0 | 1 | 1 | 1 | 1 |
| 6230213130 | 5.45 | 0.82 |  | 3.89 | 2.46 | 0 | 0 | 0 |  | 0 | 0 | 1 | 1 | 1 |
| 6230213138 | 2.4 | 1.03 |  | 0.93 | 1.07 | 1 | 0 | 0 |  | 0 | 0 | 2 | 2 | 1 |
| 6230213143 | 1.98 | 0.43 |  | 1.13 | 1.37 | 1 | 1 | 0 |  | 0 | 0 | 2 | 2 | 2 |
| 6230213151 | 5.01 | 1.01 |  | 3.53 | 1.79 | 1 | 1 | 0 |  | 0 | 1 | 2 | 2 | 2 |
| 6230213152 | 2.5 | 1.1 |  | 0.86 | 0.87 | 2 | 0 | 0 |  | 0 | 0 | 1 | 1 | 1 |
| 6230213153 | 2.27 | 0.77 |  | 1.13 | 0.97 | 1 | 1 | 0 |  | 0 | 1 | 1 | 1 | 1 |
| 6230213201 | 2.27 | 0.81 |  | 1.04 | 1.12 | 1 | 0 | 0 |  | 0 | 0 | 1 | 1 | 1 |
| 6230213203 | 3.64 | 0.87 |  | 2.29 | 1.04 | 1 | 0 | 2 |  | 0 | 0 | 3 | 3 | 3 |
| 6230213205 | 2.78 | 0.75 |  | 1.72 | 0.82 | 2 | 1 | 1 |  | 0 | 0 | 2 | 1 | 1 |
| 6230213211 | 2.95 | 0.9 |  | 1.57 | 1.06 | 1 | 0 | 0 |  | 0 | 0 | 1 | 1 | 1 |
| 6230213215 | 3.47 | 1.31 |  | 1.65 | 0.81 | 1 | 1 | 0 |  | 0 | 0 | 1 | 1 | 1 |
| 6230213222 | 5.17 | 1.39 |  | 2.97 | 1.81 | 1 | 1 | 1 |  | 0 | 1 | 1 | 1 | 1 |
| 6230213229 | 3.82 | 0.95 |  | 2.59 | 0.99 | 0 | 1 | 0 |  | 0 | 1 | 1 | 1 | 1 |
| 6230213231 | 3.15 | 0.8 |  | 2.22 | 0.69 | 2 | 0 | 0 |  | 0 | 0 | 2 | 2 | 3 |
| 6230213233 | 2.81 | 0.82 |  | 1.69 | 0.74 | 0 | 0 | 0 |  | 0 | 0 | 1 | 1 | 2 |
| 6230213234 | 3.14 | 1.2 |  | 1.44 | 1.04 | 2 | 0 | 0 |  | 0 | 0 | 1 | 1 | 1 |
| 6230213236 | 1.48 | 0.72 |  | 0.48 | 0.26 | 2 | 0 | 0 |  | 0 | 0 | 1 | 1 | 1 |
| 6230213241 | 2.99 | 0.78 |  | 1.67 | 1.16 | 2 | 0 | 0 |  | 0 | 0 | 2 | 1 | 2 |
| 6230213243 | 3.09 | 0.87 |  | 1.7 | 1.27 | 0 | 0 | 0 |  | 0 | 0 | 1 | 1 | 1 |
| 6230213246 | 2.86 | 0.84 |  | 1.64 | 1.09 | 1 | 1 | 1 |  | 0 | 0 | 2 | 2 | 3 |
| 6230213250 | 2.73 | 1.02 |  | 1.16 | 0.9 | 2 | 0 | 0 |  | 0 | 0 | 1 | 1 | 1 |
| 6230213303 | 2.91 | 0.82 |  | 1.72 | 1.07 | 1 | 1 | 1 |  | 0 | 0 | 2 | 1 | 1 |
| 6230213304 | 4.23 | 0.88 |  | 1.78 | 6.63 | 1 | 1 | 1 |  | 0 | 0 | 1 | 1 | 1 |
| 6230213307 | 3.51 | 0.88 |  | 2.04 | 1.4 | 2 | 1 | 1 |  | 0 | 0 | 1 | 1 | 1 |
| 6230213308 | 3.19 | 0.95 |  | 1.86 | 0.98 | 1 | 0 | 0 |  | 0 | 0 | 1 | 1 | 1 |
| 6230213311 | 2.73 | 0.71 |  | 1.66 | 0.76 | 1 | 0 | 0 |  | 0 | 0 | 1 | 1 | 1 |
| 6230213314 | 3.27 | 0.77 |  | 1.86 | 2.39 | 0 | 1 | 0 |  | 0 | 1 | 2 | 2 | 2 |
| 6230213315 | 2.66 | 0.71 |  | 1.56 | 0.82 | 1 | 0 | 0 |  | 0 | 0 | 1 | 1 | 1 |
| 6230213316 | 2.42 | 0.58 |  | 1.28 | 1.65 | 2 | 0 | 0 |  | 0 | 0 | 3 | 3 | 2 |
| 6230213317 | 3.92 | 0.77 |  | 2.24 | 2.58 | 2 | 0 | 2 |  | 0 | 0 | 2 | 2 | 2 |
| 6230213318 | 2.93 | 1.18 |  | 1.38 | 0.86 | 2 | 0 | 0 |  | 0 | 0 | 1 | 1 | 1 |
| 6230213319 | 3.06 | 0.75 |  | 1.74 | 1.66 | 2 | 1 | 0 |  | 0 | 0 | 1 | 1 | 1 |
| 6230213320 | 2.88 | 0.8 |  | 1.75 | 0.67 | 2 | 1 | 1 |  | 0 | 0 | 2 | 2 | 3 |
| 6230213321 | 2.91 | 0.94 |  | 1.7 | 0.84 | 1 | 1 | 1 |  | 0 | 1 | 1 | 1 | 1 |
| 6230213324 | 3.46 | 0.74 |  | 1.68 | 1.74 | 2 | 0 | 0 |  | 0 | 0 | 1 | 1 | 1 |
| 6230213325 | 3.06 | 0.88 |  | 1.56 | 1.37 | 2 | 0 | 0 |  | 0 | 0 | 1 | 1 | 1 |
| 6230213327 | 2.74 | 1.04 |  | 1.41 | 0.71 | 2 | 0 | 0 |  | 0 | 0 | 1 | 1 | 2 |
| 6230213328 | 3.86 | 1.86 |  | 1.41 | 1.12 | 1 | 1 | 0 |  | 0 | 1 | 1 | 1 | 1 |
| 6230213330 | 2.42 | 0.62 |  | 1.29 | 1.17 | 1 | 0 | 0 |  | 0 | 0 | 1 | 1 | 2 |
| 6230213333 | 2.15 | 0.46 |  | 1.52 | 0.59 | 1 | 0 | 1 |  | 0 | 0 | 3 | 3 | 3 |
| 6230213335 | 3.31 | 1.22 |  | 1.74 | 0.76 | 1 | 1 | 0 |  | 0 | 1 | 1 | 1 | 1 |
| 6230213337 | 3.31 | 1.14 |  | 1.65 | 1.24 | 1 | 1 | 0 |  | 0 | 0 | 3 | 2 | 1 |
| 6230213339 | 2.02 | 0.82 |  | 0.85 | 1.1 | 0 | 0 | 0 |  | 0 | 0 | 2 | 1 | 2 |
| 6230213341 | 2.71 | 0.72 |  | 1.72 | 0.98 | 1 | 0 | 0 |  | 0 | 0 | 2 | 2 | 1 |
| 6230213346 | 2.45 | 0.94 |  | 1.15 | 0.83 | 2 | 1 | 1 |  | 0 | 1 | 1 | 1 | 1 |
| 6230213348 | 3.34 | 1.19 |  | 1.95 | 0.6 | 0 | 0 | 0 |  | 0 | 0 | 2 | 1 | 1 |
| 6230213349 | 2.62 | 0.83 |  | 1.35 | 0.94 | 1 | 0 | 0 |  | 0 | 0 | 1 | 1 | 1 |
| 6230214106 | 4.63 | 1.02 |  | 3.1 | 1.47 | 2 | 0 | 0 |  | 0 | 0 | 1 | 1 | 1 |
| 6230214107 | 2.59 | 1.1 |  | 0.99 | 0.55 | 2 | 1 | 0 |  | 0 | 1 | 1 | 1 | 1 |
| 6230214114 | 3.99 | 1.25 |  | 2.36 | 0.8 | 2 | 1 | 2 |  | 0 | 0 | 1 | 1 | 1 |
| 6230214116 | 3.77 | 1.12 |  | 1.8 | 1.47 | 1 | 0 | 0 |  | 0 | 0 | 1 | 1 | 1 |
| 6230214117 | 3.91 | 1.35 |  | 2.21 | 0.82 | 1 | 0 | 0 |  | 0 | 0 | 2 | 1 | 2 |
| 6230214123 | 3.29 | 1.18 |  | 1.62 | 1.16 | 2 | 0 | 0 |  | 0 | 0 | 1 | 1 | 1 |
| 6230214130 | 3.25 | 0.79 |  | 1.53 | 1.82 | 2 | 0 | 0 |  | 0 | 0 | 2 | 1 | 2 |
| 6230214134 | 3.45 | 1.05 |  | 2 | 0.93 | 1 | 0 | 0 |  | 0 | 0 | 1 | 1 | 1 |
| 6230214145 | 2.14 | 0.56 |  | 1.25 | 0.83 | 2 | 0 | 0 |  | 0 | 0 | 2 | 2 | 2 |
| 6230214146 | 4.72 | 1.16 |  | 3.11 | 1.64 | 2 | 0 | 0 |  | 0 | 0 | 3 | 3 | 2 |
| 6230214202 | 3.64 | 0.98 |  | 2.17 | 1.53 | 2 | 1 | 0 |  | 0 | 1 | 1 | 1 | 1 |
| 6230214210 | 3.47 | 0.84 |  | 2.29 | 1.13 | 0 | 1 | 0 |  | 0 | 0 | 2 | 1 | 1 |
| 6230214216 | 4.66 | 1.16 |  | 2.44 | 3.22 | 0 | 1 | 0 |  | 0 | 1 | 1 | 1 | 1 |
| 6230214221 | 3.08 | 1.09 |  | 1.82 | 0.78 | 1 | 0 | 0 |  | 0 | 0 | 1 | 1 | 2 |
| 6230214235 | 4.08 | 1.25 |  | 2.3 | 1.41 | 2 | 0 | 0 |  | 0 | 0 | 2 | 2 | 2 |
| 6230214240 | 3.89 | 0.89 |  | 2.64 | 1.28 | 2 | 1 | 0 |  | 0 | 1 | 2 | 2 | 2 |
| 6230214241 | 3.14 | 0.93 |  | 1.98 | 0.85 | 1 | 1 | 0 |  | 1 | 0 | 1 | 1 | 1 |
| 6230214302 | 3.27 | 0.68 |  | 1.9 | 2.38 | 0 | 1 | 0 |  | 0 | 0 | 2 | 2 | 3 |
| 6230214308 | 4.22 | 1.24 |  | 2.66 | 0.95 | 1 | 0 | 1 |  | 0 | 0 | 1 | 1 | 3 |
| 6230214314 | 2.62 | 0.8 |  | 1.09 | 2.05 | 0 | 0 | 0 |  | 0 | 0 | 2 | 1 | 1 |
| 6230214319 | 4.37 | 1.18 |  | 2.62 | 1.57 | 2 | 0 | 0 |  | 0 | 1 | 2 | 2 | 3 |
| 6230214328 | 4.13 | 1.75 |  | 1.83 | 1.49 | 0 | 0 | 0 |  | 0 | 0 | 1 | 1 | 1 |
| 6230214335 | 2.98 | 0.96 |  | 1.58 | 1.22 | 1 | 0 | 1 |  | 0 | 0 | 1 | 1 | 1 |
| 6230214340 | 3.36 | 1.32 |  | 1.69 | 0.66 | 0 | 0 | 1 |  | 0 | 0 | 2 | 2 | 1 |
| 6230214341 | 3.23 | 0.97 |  | 1.86 | 0.86 | 2 | 0 | 0 |  | 0 | 0 | 1 | 1 | 1 |
| 6230214349 | 3.6 | 0.93 |  | 2.18 | 1.49 | 2 | 0 | 0 |  | 0 | 0 | 3 | 3 | 3 |
| 6230214352 | 5.23 | 1.46 |  | 3.47 | 1.16 | 0 | 0 | 2 |  | 0 | 0 | 1 | 1 | 1 |
| 6204231246 | 3.91 | 1.06 |  | 2.28 | 1.39 | 0 | 1 | 1 |  | 0 | 1 | 1 | 1 | 1 |
| 6204231329 | 4.09 | 1.07 |  | 2.62 | 0.94 | 1 | 1 | 1 |  | 0 | 0 | 1 | 1 | 2 |
| 6204234315 | 4.49 | 0.97 |  | 2.5 | 2.41 | 1 | 0 | 0 |  | 0 | 1 | 1 | 1 | 1 |
| 6205031348 | 4.09 | 0.85 |  | 2.16 | 2.05 | 2 | 0 | 1 |  | 0 | 0 | 1 | 1 | 1 |
| 6205032302 | 3.38 | 1.25 |  | 1.9 | 0.52 | 0 | 0 | 0 |  | 0 | 0 | 2 | 2 | 3 |
| 6205032316 | 4.8 | 1.89 |  | 2.17 | 1.62 | 1 | 1 | 1 |  | 0 | 1 | 1 | 1 | 1 |
| 6205033231 | 5.65 | 1.01 |  | 3.15 | 3.08 | 1 | 0 | 0 |  | 0 | 0 | 2 | 2 | 3 |
| 6205033237 | 3.41 | 1.16 |  | 1.47 | 1.7 | 1 | 0 | 1 |  | 0 | 0 | 3 | 2 | 3 |
| 6205033338 | 3.42 | 1.16 |  | 1.46 | 1.31 | 1 | 0 | 0 |  | 0 | 0 | 1 | 1 | 1 |
| 6205034211 | 3.72 | 0.92 |  | 2.01 | 1.54 | 0 | 1 | 0 |  | 0 | 1 | 2 | 2 | 2 |
| 6221031124 | 4.36 | 1.3 |  | 2.46 | 0.79 | 2 | 0 | 0 |  | 0 | 0 | 2 | 2 | 2 |
| 6221031127 | 3.79 | 1.25 |  | 2.05 | 0.83 | 0 | 0 | 1 |  | 0 | 0 | 1 | 1 | 1 |
| 6221031137 | 5.04 | 0.83 |  | 3.35 | 1.79 | 2 | 0 | 0 |  | 0 | 0 | 3 | 3 | 3 |
| 6221031216 | 3.3 | 0.92 |  | 1.87 | 1.12 | 0 | 0 | 1 |  | 0 | 0 | 2 | 2 | 1 |
| 6221031229 | 3.7 | 0.9 |  | 1.94 | 1.2 | 1 | 0 | 0 |  | 0 | 0 | 2 | 2 | 2 |
| 6221031231 | 3.41 | 0.93 |  | 1.93 | 1.72 | 0 | 1 | 1 |  | 1 | 1 | 3 | 2 | 3 |
| 6221031310 | 2.91 | 0.69 |  | 1.57 | 1.12 | 0 | 1 | 1 |  | 0 | 1 | 1 | 1 | 1 |
| 6221031317 | 3.39 | 0.85 |  | 1.73 | 1.92 | 1 | 1 | 2 |  | 0 | 0 | 2 | 1 | 1 |
| 6221031321 | 2.59 | 0.71 |  | 1.16 | 1.44 | 1 | 0 | 0 |  | 0 | 0 | 1 | 1 | 2 |
| 6221031322 | 2.96 | 1.08 |  | 1.41 | 0.92 | 0 | 1 | 2 |  | 0 | 0 | 1 | 1 | 1 |
| 6221032110 | 2.61 | 0.86 |  | 1.26 | 1.15 | 1 | 1 | 1 |  | 0 | 0 | 2 | 2 | 2 |
| 6221032208 | 4.27 | 1.23 |  | 2.22 | 0.98 | 0 | 0 | 0 |  | 0 | 0 | 1 | 1 | 1 |
| 6221032339 | 2.76 | 0.89 |  | 1.34 | 0.75 | 1 | 1 | 1 |  | 0 | 1 | 2 | 1 | 1 |
| 6221033115 | 4.89 | 1.31 |  | 2.73 | 2.25 | 0 | 0 | 2 |  | 0 | 0 | 2 | 1 | 1 |
| 6221033123 | 3.11 | 0.98 |  | 1.72 | 0.88 | 0 | 0 | 1 |  | 0 | 0 | 2 | 2 | 2 |
| 6221033127 | 3.62 | 1.18 |  | 1.89 | 1.09 | 0 | 0 | 1 |  | 0 | 0 | 1 | 1 | 1 |
| 6221033206 | 2.39 | 0.81 |  | 1.12 | 0.73 | 0 | 1 | 1 |  | 1 | 0 | 2 | 1 | 1 |
| 6221033227 | 6.07 | 1.04 |  | 3.74 | 2.59 | 2 | 0 | 0 |  | 0 | 0 | 2 | 2 | 3 |
| 6221033229 | 3.57 | 0.96 |  | 1.9 | 1.97 | 0 | 0 | 1 |  | 0 | 0 | 1 | 1 | 1 |
| 6221034210 | 4.08 | 0.97 |  | 1.79 | 4.84 | 0 | 1 | 2 |  | 0 | 0 | 2 | 2 | 2 |
| 6222011144 | 4.58 | 1.28 |  | 2.28 | 1.5 | 0 | 1 | 1 |  | 0 | 1 | 2 | 2 | 1 |
| 6222011307 | 3.88 | 0.9 |  | 1.83 | 1.94 | 2 | 0 | 0 |  | 0 | 0 | 3 | 3 | 3 |
| 6222012116 | 5.94 | 0.51 |  | 2.34 | 9.67 | 1 | 0 | 0 |  | 0 | 0 | 3 | 2 | 3 |
| 6222012132 | 3.04 | 1.24 |  | 1.16 | 0.68 | 0 | 0 | 1 |  | 0 | 0 | 1 | 1 | 1 |
| 6222012203 | 2.68 | 0.66 |  | 1.42 | 0.88 | 0 | 0 | 1 |  | 0 | 0 | 1 | 1 | 1 |
| 6222012301 | 3.48 | 0.63 |  | 1.62 | 3.24 | 1 | 0 | 0 |  | 0 | 0 | 1 | 1 | 2 |
| 6222012341 | 2.11 | 0.73 |  | 0.96 | 0.62 | 0 | 0 | 1 |  | 0 | 0 | 1 | 1 | 2 |
| 6222013114 | 2.8 | 0.96 |  | 1.37 | 0.62 | 1 | 1 | 0 |  | 0 | 0 | 1 | 1 | 1 |
| 6222013236 | 3.68 | 0.57 |  | 1.88 | 2.21 | 0 | 1 | 1 |  | 0 | 0 | 2 | 2 | 2 |
| 6222013239 | 3.81 | 1.08 |  | 2.04 | 0.81 | 0 | 0 | 1 |  | 0 | 0 | 1 | 1 | 1 |
| 6222013249 | 3.4 | 1.01 |  | 1.75 | 0.87 | 2 | 1 | 1 |  | 0 | 1 | 1 | 1 | 1 |
| 6222013320 | 2.71 | 0.52 |  | 1.7 | 0.64 | 0 | 1 | 2 |  | 1 | 1 | 1 | 1 | 3 |
| 6222014212 | 3.81 | 1.41 |  | 1.82 | 0.75 | 0 | 1 | 1 |  | 1 | 0 | 2 | 2 | 2 |
| 6230211111 | 3.33 | 0.81 |  | 1.94 | 1.83 | 1 | 1 | 1 |  | 0 | 1 | 2 | 1 | 1 |
| 6230211152 | 1.98 | 0.83 |  | 0.89 | 0.4 | 1 | 0 | 0 |  | 0 | 0 | 1 | 1 | 1 |
| 6230211225 | 2.47 | 1.01 |  | 1.02 | 0.54 | 0 | 0 | 0 |  | 0 | 0 | 1 | 1 | 1 |
| 6230211249 | 3.72 | 0.96 |  | 2.17 | 1.13 | 0 | 0 | 0 |  | 0 | 0 | 2 | 2 | 2 |
| 6230211319 | 2.1 | 0.63 |  | 1.16 | 0.98 | 0 | 0 | 0 |  | 0 | 0 | 1 | 1 | 2 |
| 6230212125 | 3.3 | 1.09 |  | 1.84 | 0.89 | 0 | 0 | 0 |  | 0 | 0 | 1 | 1 | 2 |
| 6230212142 | 2.82 | 0.67 |  | 1.68 | 1.77 | 2 | 1 | 1 |  | 0 | 0 | 2 | 2 | 3 |
| 6230212229 | 3.3 | 0.94 |  | 1.66 | 1.13 | 0 | 1 | 0 |  | 0 | 0 | 1 | 1 | 1 |
| 6230212252 | 4 | 1.16 |  | 2.9 | 0.99 | 1 | 0 | 0 |  | 0 | 0 | 1 | 1 | 2 |
| 6230212348 | 5 | 1.34 |  | 3.22 | 1.17 | 0 | 1 | 2 |  | 0 | 0 | 1 | 1 | 1 |
| 6230214108 | 3.47 | 0.99 |  | 2.12 | 1.04 | 1 | 0 | 0 |  | 0 | 0 | 1 | 1 | 1 |
| 6230214132 | 1.77 | 0.69 |  | 0.82 | 1.39 | 1 | 0 | 0 |  | 0 | 0 | 1 | 1 | 2 |
| 6230214136 | 3.24 | 1.13 |  | 1.69 | 1.1 | 0 | 1 | 2 |  | 0 | 1 | 1 | 1 | 1 |
| 6230214150 | 2.63 | 0.97 |  | 1.39 | 0.6 | 0 | 0 | 0 |  | 0 | 0 | 2 | 2 | 2 |
| 6230214218 | 4.37 | 0.95 |  | 2.67 | 2.01 | 0 | 0 | 0 |  | 0 | 0 | 3 | 2 | 2 |
| 6230214305 | 2.82 | 1.14 |  | 1.28 | 0.96 | 2 | 0 | 0 |  | 0 | 0 | 1 | 1 | 1 |
| 6230214316 | 2.68 | 0.7 |  | 1.43 | 1.72 | 1 | 1 | 1 |  | 0 | 1 | 1 | 1 | 1 |
| 6204233139 | 5.82 | 1.45 |  | 3.71 | 1.48 | 1 | 0 | 1 |  | 0 | 0 | 1 | 1 | 1 |
| 6204233239 | 5.84 | 1.12 |  | 3.76 | 2.15 | 2 | 0 | 0 |  | 0 | 0 | 3 | 2 | 2 |
| 6204233247 | 6.87 | 1.12 |  | 3.93 | 2.97 | 2 | 0 | 0 |  | 0 | 0 | 1 | 1 | 1 |
| 6204233252 | 3.32 | 0.7 |  | 2.43 | 0.64 | 1 | 1 | 2 |  | 0 | 0 | 1 | 1 | 2 |
| 6204234127 | 2.42 | 0.8 |  | 1.21 | 0.8 | 1 | 0 | 2 |  | 0 | 0 | 2 | 1 | 1 |
| 6204234141 | 3.66 | 1.45 |  | 1.57 | 0.76 | 2 | 0 | 0 |  | 0 | 0 | 2 | 2 | 2 |
| 6204234142 | 3.52 | 0.99 |  | 1.81 | 1.76 | 0 | 0 | 0 |  | 0 | 0 | 2 | 2 | 2 |
| 6204234204 | 3.64 | 0.99 |  | 2.1 | 1.34 | 2 | 1 | 1 |  | 0 | 1 | 1 | 1 | 1 |
| 6204234224 | 3.24 | 0.87 |  | 2.05 | 0.54 | 0 | 0 | 1 |  | 0 | 0 | 1 | 1 | 1 |
| 6204234344 | 2.94 | 1.15 |  | 1.11 | 1.28 | 1 | 0 | 0 |  | 0 | 0 | 1 | 1 | 1 |
| 6205031132 | 4.7 | 1.22 |  | 3.05 | 0.77 | 0 | 1 | 2 |  | 0 | 1 | 1 | 1 | 1 |
| 6205031205 | 2.87 | 0.75 |  | 1.55 | 1.04 | 2 | 1 | 1 |  | 0 | 0 | 1 | 1 | 1 |
| 6205031229 | 3.85 | 0.99 |  | 2.36 | 1.09 | 2 | 1 | 1 |  | 0 | 1 | 2 | 1 | 2 |
| 6205031247 | 4.67 | 0.94 |  | 2.6 | 2.56 | 0 | 1 | 1 |  | 1 | 1 | 1 | 1 | 1 |
| 6205031304 | 5.05 | 0.82 |  | 2.16 | 6.61 | 2 | 1 | 2 |  | 1 | 0 | 2 | 2 | 2 |
| 6205031345 | 4.45 | 0.53 |  | 1.84 | 5.99 | 1 | 1 | 2 |  | 1 | 1 | 2 | 2 | 2 |
| 6205032206 | 4.56 | 1.54 |  | 2.35 | 0.95 | 0 | 0 | 1 |  | 0 | 0 | 2 | 1 | 3 |
| 6205034304 | 3.76 | 1.28 |  | 2.01 | 0.85 | 1 | 1 | 2 |  | 0 | 1 | 1 | 1 | 1 |
| 6205034329 | 3.44 | 0.96 |  | 1.92 | 1.25 | 1 | 0 | 1 |  | 0 | 0 | 1 | 1 | 1 |
| 6221031122 | 3.36 | 1.04 |  | 1.71 | 0.98 | 2 | 1 | 0 |  | 0 | 0 | 2 | 1 | 1 |
| 6221031139 | 3.54 | 1.14 |  | 2.09 | 0.53 | 0 | 1 | 1 |  | 1 | 1 | 1 | 1 | 1 |
| 6221031245 | 4.07 | 0.78 |  | 2.29 | 2.07 | 0 | 1 | 1 |  | 0 | 1 | 3 | 3 | 3 |
| 6221032241 | 3.42 | 0.85 |  | 1.93 | 0.8 | 1 | 0 | 1 |  | 0 | 0 | 2 | 2 | 2 |
| 6221032338 | 3.72 | 0.85 |  | 2.3 | 1.11 | 0 | 1 | 2 |  | 0 | 1 | 2 | 2 | 3 |
| 6221033140 | 2.5 | 0.63 |  | 1.35 | 1.46 | 1 | 0 | 0 |  | 0 | 0 | 1 | 1 | 1 |
| 6222012211 | 3.56 | 0.96 |  | 1.94 | 1.07 | 1 | 0 | 1 |  | 0 | 0 | 1 | 1 | 2 |
| 6222013309 | 2.87 | 0.78 |  | 1.49 | 0.78 | 0 | 1 | 2 |  | 0 | 0 | 1 | 1 | 1 |
| 6230211107 | 3.31 | 0.94 |  | 1.88 | 1.21 | 0 | 0 | 0 |  | 0 | 0 | 2 | 2 | 2 |
| 6230211224 | 3.21 | 0.95 |  | 1.78 | 0.98 | 1 | 0 | 0 |  | 0 | 0 | 2 | 1 | 1 |
| 6230211230 | 3.7 | 0.99 |  | 1.93 | 2.38 | 2 | 0 | 0 |  | 0 | 0 | 2 | 2 | 3 |
| 6230211311 | 2.88 | 0.89 |  | 1.66 | 1.03 | 0 | 0 | 0 |  | 0 | 0 | 1 | 1 | 1 |
| 6230212227 | 2.21 | 0.85 |  | 0.97 | 0.74 | 1 | 1 | 0 |  | 0 | 1 | 1 | 1 | 1 |
| 6230212232 | 3.15 | 1.22 |  | 1.67 | 0.52 | 0 | 0 | 0 |  | 0 | 0 | 1 | 1 | 1 |
| 6230212336 | 3.62 | 0.88 |  | 2.4 | 1.38 | 2 | 0 | 0 |  | 0 | 0 | 1 | 1 | 1 |
| 6230213240 | 2.53 | 1 |  | 0.97 | 1.01 | 0 | 0 | 0 |  | 0 | 0 | 1 | 1 | 1 |
| 6230214121 | 3.29 | 0.87 |  | 1.95 | 1.21 | 1 | 1 | 0 |  | 0 | 1 | 2 | 2 | 3 |
| 6230214133 | 2.33 | 0.96 |  | 1.03 | 0.75 | 2 | 1 | 0 |  | 0 | 1 | 1 | 1 | 1 |
| 6204231101 | 4.24 | 1.13 |  | 2.75 | 0.59 | 1 | 1 | 1 |  | 0 | 1 | 1 | 1 | 1 |
| 6204231103 | 4.39 | 1.43 |  | 2.52 | 0.74 | 1 | 0 | 1 |  | 0 | 0 | 1 | 1 | 1 |
| 6204231104 | 3.78 | 1.22 |  | 2.31 | 0.55 | 0 | 0 | 0 |  | 0 | 0 | 2 | 2 | 2 |
| 6204231105 | 4.16 | 1.79 |  | 1.82 | 0.49 | 1 | 0 | 2 |  | 0 | 0 | 1 | 1 | 1 |
| 6204231108 | 3.74 | 1.15 |  | 2.29 | 0.67 | 0 | 1 | 1 |  | 0 | 1 | 1 | 1 | 1 |
| 6204231109 | 4.52 | 1.54 |  | 2.4 | 1.39 | 2 | 1 | 0 |  | 0 | 1 | 2 | 2 | 1 |
| 6204231110 | 4.44 | 1.3 |  | 2.51 | 1.05 | 1 | 1 | 2 |  | 0 | 1 | 1 | 1 | 1 |
| 6204231111 | 4.65 | 1.09 |  | 2.78 | 1.66 | 2 | 0 | 0 |  | 0 | 0 | 3 | 2 | 3 |
| 6204231112 | 4.34 | 1.51 |  | 2.41 | 0.76 | 0 | 0 | 0 |  | 0 | 0 | 3 | 2 | 2 |
| 6204231113 | 3.9 | 1.18 |  | 2.34 | 0.58 | 0 | 0 | 1 |  | 0 | 0 | 2 | 2 | 1 |
| 6204231114 | 4.73 | 1.15 |  | 2.87 | 1.2 | 1 | 0 | 1 |  | 0 | 0 | 1 | 1 | 1 |
| 6204231116 | 4.75 | 0.98 |  | 3.25 | 1.64 | 1 | 1 | 1 |  | 0 | 0 | 1 | 1 | 2 |
| 6204231117 | 3.4 | 1.06 |  | 1.96 | 0.91 | 1 | 1 | 2 |  | 0 | 1 | 1 | 1 | 1 |
| 6204231118 | 3.96 | 1.35 |  | 2.17 | 0.59 | 1 | 1 | 2 |  | 0 | 1 | 1 | 1 | 1 |
| 6204231119 | 3.16 | 1.46 |  | 1.31 | 0.39 | 0 | 0 | 1 |  | 0 | 0 | 1 | 1 | 1 |
| 6204231120 | 3.93 | 1.58 |  | 2.02 | 0.5 | 0 | 0 | 1 |  | 0 | 0 | 1 | 1 | 1 |
| 6204231121 | 5.26 | 1.25 |  | 3.58 | 1.05 | 1 | 0 | 0 |  | 0 | 0 | 1 | 1 | 2 |
| 6204231124 | 4.54 | 1.03 |  | 3.14 | 1.35 | 0 | 0 | 0 |  | 0 | 0 | 3 | 2 | 1 |
| 6204231125 | 4.31 | 1.06 |  | 2.78 | 1.07 | 1 | 1 | 1 |  | 1 | 1 | 1 | 1 | 1 |
| 6204231126 | 4.56 | 0.92 |  | 2.79 | 1.99 | 0 | 1 | 1 |  | 0 | 1 | 1 | 1 | 1 |
| 6204231127 | 3.5 | 1.09 |  | 2.11 | 0.67 | 1 | 0 | 1 |  | 0 | 0 | 1 | 1 | 1 |
| 6204231129 | 4.33 | 1.22 |  | 2.6 | 1.46 | 1 | 0 | 1 |  | 0 | 0 | 2 | 2 | 2 |
| 6204231130 | 4.34 | 1.04 |  | 3 | 0.91 | 0 | 0 | 1 |  | 0 | 0 | 2 | 2 | 2 |
| 6204231131 | 3.56 | 1.18 |  | 2.07 | 0.64 | 0 | 1 | 1 |  | 0 | 1 | 1 | 1 | 1 |
| 6204231132 | 4.58 | 0.93 |  | 3.29 | 1.22 | 2 | 1 | 0 |  | 0 | 0 | 2 | 2 | 2 |
| 6204231133 | 2.57 | 0.92 |  | 1.46 | 0.39 | 0 | 1 | 1 |  | 1 | 0 | 2 | 2 | 2 |
| 6204231134 | 3.29 | 1.29 |  | 1.46 | 0.68 | 1 | 1 | 0 |  | 0 | 1 | 2 | 2 | 1 |
| 6204231135 | 5.28 | 1.31 |  | 3.6 | 1.17 | 1 | 0 | 2 |  | 0 | 0 | 2 | 2 | 2 |
| 6204231136 | 4.4 | 1 |  | 3.14 | 0.79 | 1 | 0 | 2 |  | 0 | 0 | 1 | 1 | 1 |
| 6204231138 | 3.21 | 1.3 |  | 1.43 | 0.65 | 0 | 0 | 1 |  | 0 | 0 | 1 | 1 | 1 |
| 6204231139 | 3.44 | 0.96 |  | 1.95 | 0.87 | 2 | 0 | 0 |  | 0 | 1 | 1 | 1 | 1 |
| 6204231141 | 2.52 | 0.8 |  | 1.41 | 0.45 | 0 | 1 | 1 |  | 0 | 1 | 1 | 1 | 1 |
| 6204231142 | 2.94 | 0.65 |  | 1.86 | 1.36 | 0 | 1 | 1 |  | 0 | 1 | 3 | 3 | 3 |
| 6204231143 | 5.19 | 1.28 |  | 3.45 | 1 | 1 | 0 | 0 |  | 0 | 0 | 1 | 1 | 1 |
| 6204231144 | 4.93 | 1 |  | 2.79 | 3.05 | 0 | 0 | 0 |  | 0 | 0 | 3 | 2 | 2 |
| 6204231147 | 2.96 | 0.89 |  | 1.68 | 0.89 | 1 | 1 | 2 |  | 0 | 0 | 1 | 1 | 1 |
| 6204231148 | 4.03 | 0.99 |  | 2.77 | 0.79 | 1 | 1 | 1 |  | 1 | 0 | 2 | 2 | 2 |
| 6204231151 | 4.31 | 1.49 |  | 2.43 | 0.45 | 1 | 1 | 0 |  | 0 | 0 | 1 | 1 | 1 |
| 6204231152 | 4.64 | 1.21 |  | 2.86 | 1.16 | 1 | 0 | 1 |  | 0 | 0 | 1 | 1 | 1 |
| 6204231153 | 4.17 | 0.82 |  | 2.79 | 1.45 | 1 | 1 | 2 |  | 0 | 1 | 2 | 2 | 3 |
| 6204231154 | 4.26 | 0.91 |  | 2.46 | 2.62 | 1 | 0 | 0 |  | 0 | 0 | 3 | 3 | 3 |
| 6204231201 | 3.67 | 0.79 |  | 2.25 | 1.08 | 2 | 0 | 0 |  | 0 | 0 | 1 | 1 | 1 |
| 6204231202 | 3.14 | 1.01 |  | 1.76 | 0.71 | 0 | 0 | 1 |  | 0 | 0 | 1 | 1 | 1 |
| 6204231203 | 2.51 | 0.96 |  | 1.3 | 0.4 | 0 | 0 | 0 |  | 0 | 0 | 1 | 1 | 1 |
| 6204231205 | 3.89 | 0.75 |  | 1.96 | 2.82 | 1 | 1 | 2 |  | 0 | 1 | 2 | 1 | 1 |
| 6204231206 | 3.48 | 1.08 |  | 1.92 | 1.04 | 0 | 1 | 1 |  | 0 | 1 | 1 | 1 | 1 |
| 6204231207 | 3.28 | 1.19 |  | 1.75 | 0.8 | 1 | 1 | 1 |  | 0 | 1 | 1 | 1 | 1 |
| 6204231209 | 2.82 | 1.13 |  | 1.42 | 0.46 | 0 | 0 | 1 |  | 0 | 0 | 3 | 2 | 2 |
| 6204231210 | 4.05 | 1.16 |  | 2.61 | 0.59 | 1 | 0 | 1 |  | 0 | 0 | 3 | 2 | 3 |
| 6204231212 | 3.71 | 1.43 |  | 1.75 | 0.64 | 1 | 0 | 0 |  | 0 | 0 | 1 | 1 | 1 |
| 6204231213 | 3.16 | 0.78 |  | 2.1 | 0.74 | 1 | 1 | 1 |  | 0 | 0 | 2 | 2 | 2 |
| 6204231214 | 2.66 | 0.79 |  | 1.39 | 0.85 | 1 | 1 | 2 |  | 1 | 1 | 2 | 1 | 2 |
| 6204231215 | 2.97 | 0.8 |  | 1.83 | 0.69 | 1 | 1 | 1 |  | 0 | 1 | 1 | 1 | 1 |
| 6204231216 | 4.46 | 1.12 |  | 2.84 | 0.87 | 1 | 1 | 1 |  | 0 | 1 | 1 | 1 | 1 |
| 6204231217 | 3.05 | 1.12 |  | 1.47 | 1.02 | 0 | 0 | 1 |  | 0 | 0 | 1 | 1 | 1 |
| 6204231219 | 3.52 | 1.23 |  | 1.94 | 0.7 | 0 | 0 | 2 |  | 0 | 0 | 2 | 2 | 2 |
| 6204231221 | 2.23 | 0.78 |  | 1.15 | 0.52 | 1 | 0 | 0 |  | 0 | 0 | 1 | 1 | 1 |
| 6204231222 | 4 | 1 |  | 2.51 | 0.87 | 0 | 1 | 1 |  | 0 | 1 | 2 | 2 | 1 |
| 6204231223 | 3.72 | 0.94 |  | 2.54 | 0.97 | 1 | 1 | 1 |  | 0 | 1 | 1 | 1 | 1 |
| 6204231224 | 3.17 | 0.99 |  | 1.86 | 0.7 | 0 | 1 | 1 |  | 0 | 1 | 1 | 1 | 1 |
| 6204231225 | 3.86 | 1.3 |  | 2.04 | 1.18 | 1 | 0 | 0 |  | 0 | 0 | 1 | 1 | 1 |
| 6204231226 | 3.37 | 1.05 |  | 1.97 | 0.56 | 1 | 0 | 1 |  | 0 | 0 | 1 | 1 | 1 |
| 6204231227 | 2.19 | 0.76 |  | 1.07 | 1.04 | 0 | 0 | 1 |  | 0 | 0 | 1 | 1 | 1 |
| 6204231230 | 3.06 | 0.95 |  | 1.75 | 0.65 | 0 | 1 | 1 |  | 0 | 1 | 1 | 1 | 1 |
| 6204231231 | 4 | 1.29 |  | 2.22 | 1.1 | 0 | 1 | 1 |  | 0 | 1 | 1 | 1 | 1 |
| 6204231232 | 3.58 | 0.82 |  | 2.34 | 0.96 | 0 | 1 | 1 |  | 0 | 1 | 1 | 1 | 1 |
| 6204231233 | 3.33 | 1.17 |  | 1.8 | 0.42 | 0 | 0 | 1 |  | 0 | 0 | 1 | 1 | 1 |
| 6204231234 | 3.9 | 1.24 |  | 1.93 | 1.31 | 1 | 0 | 2 |  | 0 | 0 | 1 | 1 | 1 |
| 6204231235 | 2.84 | 1.05 |  | 1.33 | 0.81 | 1 | 0 | 0 |  | 0 | 0 | 1 | 1 | 1 |
| 6204231236 | 3.67 | 1.27 |  | 2.01 | 0.87 | 1 | 1 | 1 |  | 0 | 1 | 1 | 1 | 1 |
| 6204231237 | 5.34 | 1.38 |  | 3.63 | 0.63 | 0 | 1 | 1 |  | 0 | 1 | 2 | 1 | 1 |
| 6204231238 | 3.26 | 1.04 |  | 1.78 | 1 | 0 | 1 | 1 |  | 0 | 1 | 1 | 1 | 1 |
| 6204231239 | 3.14 | 0.74 |  | 1.74 | 1.48 | 0 | 1 | 1 |  | 0 | 1 | 2 | 2 | 1 |
| 6204231240 | 3.75 | 0.8 |  | 2.5 | 1.01 | 0 | 1 | 1 |  | 0 | 1 | 1 | 1 | 1 |
| 6204231241 | 3.73 | 1.06 |  | 2.31 | 0.73 | 1 | 0 | 0 |  | 0 | 0 | 1 | 1 | 1 |
| 6204231243 | 2.81 | 0.83 |  | 1.86 | 0.48 | 0 | 0 | 2 |  | 0 | 0 | 1 | 1 | 1 |
| 6204231245 | 3.7 | 0.95 |  | 2.14 | 1.45 | 1 | 1 | 1 |  | 0 | 1 | 1 | 1 | 1 |
| 6204231247 | 3.07 | 0.99 |  | 1.77 | 0.57 | 0 | 1 | 1 |  | 0 | 1 | 1 | 1 | 1 |
| 6204231248 | 3.65 | 1.17 |  | 2.03 | 0.7 | 1 | 0 | 1 |  | 0 | 0 | 1 | 1 | 1 |
| 6204231249 | 3.67 | 1.16 |  | 1.86 | 0.99 | 0 | 0 | 1 |  | 0 | 0 | 1 | 1 | 1 |
| 6204231250 | 3.28 | 0.98 |  | 1.73 | 0.89 | 1 | 0 | 0 |  | 0 | 0 | 2 | 1 | 1 |
| 6204231301 | 3 | 0.82 |  | 1.83 | 0.79 | 0 | 0 | 0 |  | 0 | 0 | 2 | 1 | 1 |
| 6204231302 | 3.12 | 1.17 |  | 1.69 | 0.28 | 1 | 0 | 1 |  | 0 | 0 | 1 | 1 | 1 |
| 6204231303 | 3.01 | 0.82 |  | 1.85 | 0.69 | 1 | 1 | 2 |  | 0 | 0 | 2 | 2 | 2 |
| 6204231305 | 3.43 | 0.87 |  | 2.1 | 1.22 | 0 | 1 | 2 |  | 0 | 1 | 2 | 2 | 3 |
| 6204231306 | 3.86 | 1.15 |  | 2.23 | 1.17 | 0 | 0 | 1 |  | 0 | 0 | 2 | 2 | 2 |
| 6204231308 | 3.88 | 1.43 |  | 2.02 | 0.53 | 0 | 0 | 1 |  | 0 | 0 | 1 | 1 | 1 |
| 6204231310 | 4.74 | 1.45 |  | 2.95 | 0.5 | 0 | 0 | 1 |  | 0 | 0 | 2 | 2 | 1 |
| 6204231311 | 2.88 | 0.72 |  | 1.9 | 0.61 | 2 | 1 | 1 |  | 0 | 0 | 1 | 1 | 1 |
| 6204231312 | 4.04 | 1.06 |  | 2.5 | 0.82 | 2 | 1 | 0 |  | 1 | 0 | 1 | 1 | 1 |
| 6204231313 | 4.39 | 0.75 |  | 3.16 | 1.36 | 1 | 1 | 1 |  | 0 | 1 | 2 | 2 | 2 |
| 6204231314 | 2.66 | 1.27 |  | 1.02 | 0.48 | 1 | 0 | 1 |  | 0 | 0 | 1 | 1 | 1 |
| 6204231316 | 5.6 | 1.52 |  | 3.63 | 0.87 | 1 | 0 | 0 |  | 0 | 0 | 2 | 2 | 2 |
| 6204231318 | 3.6 | 1.01 |  | 2.22 | 0.76 | 0 | 0 | 1 |  | 0 | 0 | 2 | 2 | 2 |
| 6204231320 | 4.27 | 1.16 |  | 2.9 | 0.5 | 1 | 1 | 0 |  | 0 | 0 | 1 | 1 | 1 |
| 6204231321 | 3.49 | 0.69 |  | 2.22 | 1.1 | 1 | 1 | 1 |  | 0 | 1 | 2 | 2 | 3 |
| 6204231322 | 3.42 | 0.76 |  | 2.1 | 0.71 | 0 | 1 | 1 |  | 0 | 1 | 2 | 2 | 2 |
| 6204231324 | 3.15 | 1.18 |  | 1.54 | 0.62 | 0 | 0 | 1 |  | 0 | 0 | 1 | 1 | 1 |
| 6204231325 | 4.01 | 0.99 |  | 2.63 | 1.14 | 0 | 0 | 0 |  | 0 | 0 | 3 | 2 | 3 |
| 6204231326 | 4.19 | 1.06 |  | 2.59 | 1.32 | 0 | 0 | 1 |  | 0 | 0 | 2 | 1 | 2 |
| 6204231328 | 4.2 | 0.94 |  | 2.57 | 1.59 | 1 | 0 | 0 |  | 0 | 0 | 2 | 2 | 2 |
| 6204231330 | 3.46 | 1.23 |  | 1.83 | 0.46 | 0 | 1 | 1 |  | 1 | 1 | 1 | 1 | 1 |
| 6204231331 | 2.31 | 1.04 |  | 0.88 | 0.59 | 0 | 0 | 0 |  | 0 | 0 | 1 | 1 | 1 |
| 6204231332 | 2.95 | 0.83 |  | 1.87 | 0.49 | 0 | 0 | 0 |  | 0 | 0 | 1 | 1 | 1 |
| 6204231334 | 3.33 | 0.99 |  | 2.04 | 0.57 | 0 | 0 | 1 |  | 0 | 0 | 1 | 1 | 1 |
| 6204231335 | 2.71 | 0.9 |  | 1.46 | 0.74 | 0 | 1 | 1 |  | 0 | 0 | 1 | 1 | 2 |
| 6204231336 | 4 | 1.3 |  | 2.38 | 0.79 | 1 | 1 | 1 |  | 1 | 1 | 1 | 1 | 1 |
| 6204231338 | 4.65 | 1.58 |  | 2.46 | 0.84 | 0 | 1 | 1 |  | 0 | 1 | 1 | 1 | 1 |
| 6204231339 | 4.11 | 1.22 |  | 2.49 | 0.68 | 1 | 1 | 1 |  | 0 | 1 | 1 | 1 | 1 |
| 6204231341 | 4.59 | 1.34 |  | 2.63 | 0.71 | 2 | 0 | 0 |  | 0 | 0 | 1 | 1 | 1 |
| 6204231342 | 3.46 | 1.41 |  | 1.66 | 0.64 | 0 | 0 | 2 |  | 0 | 0 | 1 | 1 | 1 |
| 6204231343 | 3.17 | 1.23 |  | 1.66 | 0.5 | 1 | 1 | 0 |  | 0 | 0 | 2 | 1 | 1 |
| 6204231346 | 3.46 | 1 |  | 2.17 | 0.64 | 1 | 1 | 1 |  | 0 | 1 | 2 | 1 | 2 |
| 6204231347 | 5.26 | 1.24 |  | 3.14 | 1.71 | 1 | 0 | 0 |  | 0 | 0 | 1 | 1 | 2 |
| 6204231348 | 3.85 | 1.2 |  | 2 | 0.96 | 1 | 0 | 1 |  | 0 | 0 | 3 | 3 | 3 |
| 6204231349 | 3.83 | 1.37 |  | 1.89 | 1.08 | 1 | 0 | 0 |  | 0 | 0 | 1 | 1 | 1 |
| 6204231350 | 3.33 | 0.96 |  | 1.97 | 1.01 | 1 | 0 | 0 |  | 0 | 0 | 3 | 2 | 2 |
| 6204232101 | 4.15 | 1.47 |  | 2.08 | 0.93 | 1 | 0 | 0 |  | 0 | 0 | 1 | 1 | 1 |
| 6204232102 | 4.33 | 1.4 |  | 2.24 | 1.04 | 0 | 0 | 1 |  | 0 | 0 | 1 | 1 | 1 |
| 6204232103 | 3.97 | 1.37 |  | 2.15 | 0.91 | 0 | 1 | 1 |  | 0 | 1 | 1 | 1 | 1 |
| 6204232104 | 4.73 | 1.21 |  | 2.96 | 1.01 | 1 | 0 | 0 |  | 0 | 0 | 1 | 1 | 1 |
| 6204232105 | 2.76 | 1.32 |  | 1.06 | 0.37 | 0 | 0 | 1 |  | 0 | 0 | 1 | 1 | 1 |
| 6204232106 | 5.19 | 1.7 |  | 2.92 | 0.73 | 0 | 0 | 1 |  | 0 | 0 | 2 | 1 | 1 |
| 6204232108 | 4.81 | 1.4 |  | 2.85 | 1.45 | 1 | 0 | 0 |  | 0 | 0 | 2 | 2 | 2 |
| 6204232109 | 3.33 | 1.04 |  | 1.97 | 0.42 | 1 | 1 | 1 |  | 0 | 1 | 2 | 1 | 2 |
| 6204232112 | 2.8 | 1.01 |  | 1.52 | 0.58 | 0 | 1 | 2 |  | 0 | 0 | 1 | 1 | 1 |
| 6204232113 | 3.56 | 1.27 |  | 1.8 | 0.84 | 1 | 0 | 0 |  | 0 | 0 | 1 | 1 | 1 |
| 6204232116 | 4.01 | 1.3 |  | 2.33 | 0.68 | 1 | 0 | 0 |  | 0 | 0 | 1 | 1 | 2 |
| 6204232117 | 4.07 | 0.91 |  | 2.95 | 0.72 | 0 | 1 | 1 |  | 0 | 1 | 2 | 2 | 2 |
| 6204232118 | 4.08 | 0.84 |  | 2.78 | 1 | 0 | 1 | 1 |  | 0 | 1 | 2 | 2 | 1 |
| 6204232119 | 3.49 | 1.62 |  | 1.45 | 0.9 | 0 | 0 | 1 |  | 0 | 0 | 1 | 1 | 1 |
| 6204232120 | 3.8 | 1.02 |  | 2.28 | 1.14 | 0 | 1 | 1 |  | 0 | 1 | 2 | 2 | 3 |
| 6204232121 | 4.56 | 1.03 |  | 3.04 | 1.08 | 0 | 1 | 2 |  | 0 | 1 | 1 | 1 | 1 |
| 6204232123 | 4.27 | 1.86 |  | 1.75 | 1.19 | 0 | 0 | 0 |  | 0 | 0 | 1 | 1 | 1 |
| 6204232124 | 3.82 | 0.93 |  | 2.25 | 1.73 | 0 | 0 | 1 |  | 0 | 0 | 2 | 1 | 2 |
| 6204232125 | 4.06 | 1.55 |  | 1.94 | 1.48 | 0 | 1 | 1 |  | 0 | 1 | 1 | 1 | 1 |
| 6204232126 | 4.94 | 1.25 |  | 3.12 | 1.66 | 0 | 1 | 1 |  | 0 | 1 | 1 | 1 | 1 |
| 6204232127 | 3.78 | 0.81 |  | 2.46 | 1.28 | 1 | 1 | 0 |  | 0 | 1 | 1 | 1 | 1 |
| 6204232129 | 2.94 | 0.87 |  | 0.91 | 3.36 | 2 | 0 | 0 |  | 0 | 0 | 2 | 1 | 2 |
| 6204232130 | 3.51 | 1.17 |  | 1.84 | 0.95 | 0 | 0 | 1 |  | 0 | 0 | 1 | 1 | 1 |
| 6204232131 | 4.93 | 1.25 |  | 2.95 | 1.45 | 0 | 0 | 0 |  | 0 | 0 | 1 | 1 | 1 |
| 6204232132 | 3.9 | 1.22 |  | 2.16 | 0.73 | 1 | 0 | 0 |  | 0 | 0 | 1 | 1 | 1 |
| 6204232133 | 3.46 | 0.87 |  | 2.04 | 1.54 | 1 | 1 | 1 |  | 0 | 1 | 2 | 1 | 1 |
| 6204232135 | 3.46 | 0.75 |  | 2.16 | 1.49 | 1 | 1 | 1 |  | 0 | 1 | 3 | 2 | 2 |
| 6204232136 | 5.02 | 1.12 |  | 3.16 | 1.68 | 1 | 0 | 0 |  | 0 | 0 | 2 | 2 | 1 |
| 6204232138 | 3.5 | 0.79 |  | 2.24 | 1.08 | 0 | 0 | 2 |  | 0 | 0 | 2 | 2 | 2 |
| 6204232139 | 5 | 1.07 |  | 2.24 | 3.83 | 1 | 0 | 0 |  | 0 | 0 | 2 | 2 | 2 |
| 6204232140 | 3.59 | 1.07 |  | 2.23 | 0.68 | 0 | 0 | 1 |  | 0 | 0 | 2 | 1 | 1 |
| 6204232141 | 4.42 | 1.21 |  | 2.89 | 0.92 | 0 | 1 | 1 |  | 0 | 1 | 1 | 1 | 1 |
| 6204232142 | 4.08 | 0.65 |  | 2.28 | 3.3 | 0 | 1 | 1 |  | 0 | 1 | 2 | 2 | 3 |
| 6204232143 | 3.78 | 1.44 |  | 2.04 | 0.52 | 1 | 1 | 1 |  | 0 | 1 | 1 | 1 | 1 |
| 6204232144 | 6.11 | 1.63 |  | 3.75 | 1.7 | 0 | 1 | 0 |  | 1 | 1 | 2 | 2 | 1 |
| 6204232147 | 4.77 | 0.99 |  | 2.84 | 2.81 | 0 | 0 | 0 |  | 0 | 0 | 2 | 2 | 2 |
| 6204232149 | 4.16 | 1.22 |  | 2.38 | 1.01 | 2 | 1 | 1 |  | 0 | 0 | 1 | 1 | 1 |
| 6204232150 | 4.1 | 1.31 |  | 2.32 | 1.17 | 1 | 1 | 2 |  | 0 | 1 | 2 | 1 | 1 |
| 6204232151 | 5.6 | 1.6 |  | 3.41 | 0.97 | 1 | 0 | 0 |  | 0 | 0 | 1 | 1 | 1 |
| 6204232201 | 2.73 | 1 |  | 1.06 | 1.59 | 0 | 1 | 1 |  | 0 | 1 | 1 | 1 | 1 |
| 6204232203 | 3.44 | 0.67 |  | 1.38 | 4.07 | 1 | 0 | 0 |  | 0 | 0 | 2 | 1 | 2 |
| 6204232205 | 2.53 | 1.21 |  | 0.88 | 0.52 | 0 | 0 | 1 |  | 0 | 0 | 1 | 1 | 1 |
| 6204232206 | 4.14 | 1.21 |  | 2.63 | 0.76 | 0 | 0 | 2 |  | 0 | 0 | 1 | 1 | 1 |
| 6204232210 | 3.7 | 1.36 |  | 1.8 | 1.29 | 0 | 1 | 2 |  | 0 | 0 | 1 | 1 | 1 |
| 6204232211 | 2.8 | 0.8 |  | 1.65 | 0.82 | 0 | 1 | 2 |  | 0 | 0 | 1 | 1 | 1 |
| 6204232213 | 3.6 | 1.75 |  | 1.09 | 1.16 | 0 | 0 | 1 |  | 0 | 0 | 1 | 1 | 1 |
| 6204232215 | 4.25 | 1.24 |  | 2.51 | 1.21 | 0 | 1 | 2 |  | 0 | 1 | 1 | 1 | 1 |
| 6204232216 | 4.5 | 0.89 |  | 2.18 | 3.22 | 0 | 1 | 2 |  | 0 | 0 | 2 | 2 | 3 |
| 6204232218 | 4.08 | 1.03 |  | 2.31 | 1.25 | 0 | 0 | 2 |  | 0 | 0 | 1 | 1 | 1 |
| 6204232219 | 3.05 | 1.32 |  | 1.42 | 0.58 | 0 | 0 | 0 |  | 0 | 0 | 1 | 1 | 1 |
| 6204232220 | 3.16 | 1.46 |  | 1.07 | 0.85 | 0 | 0 | 1 |  | 0 | 0 | 1 | 1 | 1 |
| 6204232221 | 2.95 | 0.99 |  | 1.61 | 0.58 | 0 | 0 | 1 |  | 0 | 0 | 1 | 1 | 1 |
| 6204232223 | 4.02 | 1.09 |  | 2.64 | 0.56 | 2 | 1 | 0 |  | 1 | 1 | 2 | 2 | 1 |
| 6204232226 | 3.86 | 0.78 |  | 2.39 | 1.78 | 0 | 1 | 1 |  | 0 | 0 | 2 | 2 | 2 |
| 6204232227 | 2.93 | 0.73 |  | 1.37 | 2.27 | 0 | 1 | 1 |  | 0 | 1 | 1 | 1 | 1 |
| 6204232229 | 5.28 | 1.26 |  | 3.34 | 1.82 | 0 | 0 | 2 |  | 0 | 0 | 1 | 1 | 1 |
| 6204232230 | 4.14 | 1.29 |  | 2.57 | 0.5 | 2 | 1 | 0 |  | 0 | 1 | 1 | 1 | 1 |
| 6204232231 | 3.74 | 0.97 |  | 2.21 | 1.03 | 1 | 1 | 1 |  | 0 | 0 | 1 | 1 | 1 |
| 6204232233 | 3.64 | 1.07 |  | 2.2 | 0.65 | 0 | 1 | 1 |  | 0 | 0 | 2 | 1 | 1 |
| 6204232234 | 5.26 | 1.14 |  | 3.57 | 1.16 | 0 | 1 | 0 |  | 0 | 1 | 2 | 2 | 1 |
| 6204232236 | 5.07 | 1.2 |  | 2.68 | 2.28 | 0 | 0 | 1 |  | 0 | 0 | 2 | 2 | 1 |
| 6204232237 | 3.31 | 1.35 |  | 1.61 | 0.68 | 1 | 0 | 1 |  | 0 | 0 | 1 | 1 | 1 |
| 6204232238 | 4.31 | 1.74 |  | 1.95 | 0.83 | 1 | 0 | 1 |  | 0 | 0 | 2 | 2 | 3 |
| 6204232242 | 4.1 | 1.12 |  | 2.31 | 1.2 | 1 | 0 | 1 |  | 0 | 0 | 1 | 1 | 1 |
| 6204232244 | 4.17 | 1.37 |  | 2.18 | 0.78 | 2 | 0 | 0 |  | 0 | 0 | 1 | 1 | 1 |
| 6204232245 | 3.13 | 1.24 |  | 1.4 | 0.85 | 0 | 0 | 2 |  | 0 | 0 | 1 | 1 | 1 |
| 6204232247 | 4.17 | 1.04 |  | 2.15 | 3.28 | 0 | 1 | 1 |  | 0 | 1 | 2 | 2 | 3 |
| 6204232248 | 4.68 | 0.86 |  | 3.34 | 1.36 | 1 | 1 | 2 |  | 1 | 1 | 2 | 1 | 2 |
| 6204232249 | 3.8 | 1.24 |  | 1.98 | 1.12 | 2 | 1 | 0 |  | 1 | 0 | 1 | 1 | 1 |
| 6204232250 | 4.7 | 0.85 |  | 2.08 | 6.26 | 0 | 1 | 1 |  | 1 | 1 | 3 | 3 | 3 |
| 6204232251 | 4.71 | 1.34 |  | 2.69 | 1.31 | 2 | 1 | 0 |  | 0 | 1 | 1 | 1 | 1 |
| 6204232301 | 3.9 | 1.22 |  | 2.33 | 0.74 | 1 | 0 | 0 |  | 0 | 0 | 1 | 1 | 1 |
| 6204232302 | 5.22 | 2 |  | 2.67 | 1.41 | 0 | 0 | 1 |  | 0 | 0 | 2 | 2 | 2 |
| 6204232304 | 2.89 | 0.9 |  | 1.36 | 1.2 | 1 | 0 | 1 |  | 0 | 0 | 2 | 2 | 2 |
| 6204232306 | 3.3 | 1.34 |  | 1.54 | 0.69 | 2 | 1 | 0 |  | 0 | 1 | 1 | 1 | 1 |
| 6204232308 | 3.82 | 0.99 |  | 2.23 | 1.05 | 2 | 1 | 0 |  | 0 | 0 | 1 | 1 | 2 |
| 6204232309 | 3.62 | 1.66 |  | 1.25 | 1.32 | 1 | 1 | 0 |  | 1 | 1 | 1 | 1 | 1 |
| 6204232310 | 3.84 | 1.18 |  | 1.9 | 1.02 | 1 | 0 | 0 |  | 0 | 0 | 1 | 1 | 1 |
| 6204232312 | 3.84 | 1.41 |  | 1.86 | 0.7 | 1 | 0 | 0 |  | 0 | 0 | 2 | 2 | 1 |
| 6204232313 | 4.66 | 1.02 |  | 2.78 | 2.49 | 1 | 1 | 0 |  | 1 | 1 | 2 | 2 | 2 |
| 6204232316 | 3.86 | 1.25 |  | 2.26 | 0.81 | 1 | 1 | 0 |  | 0 | 0 | 1 | 1 | 1 |
| 6204232317 | 3.65 | 0.85 |  | 2.48 | 0.91 | 0 | 0 | 0 |  | 0 | 0 | 2 | 2 | 1 |
| 6204232318 | 5.85 | 1.58 |  | 3.65 | 1.31 | 2 | 0 | 0 |  | 0 | 0 | 1 | 1 | 2 |
| 6204232321 | 4.65 | 1 |  | 3.25 | 0.83 | 1 | 1 | 2 |  | 0 | 0 | 3 | 2 | 3 |
| 6204232322 | 4.73 | 1.21 |  | 2.56 | 1.66 | 1 | 1 | 2 |  | 0 | 0 | 1 | 1 | 1 |
| 6204232324 | 4.43 | 1.31 |  | 2.4 | 1.43 | 2 | 0 | 0 |  | 0 | 0 | 2 | 2 | 2 |
| 6204232325 | 3.27 | 1 |  | 1.91 | 0.78 | 1 | 0 | 2 |  | 0 | 0 | 2 | 2 | 2 |
| 6204232326 | 5.02 | 1.22 |  | 3.18 | 1.67 | 2 | 0 | 0 |  | 0 | 0 | 1 | 1 | 1 |
| 6204232329 | 3.09 | 0.77 |  | 1.81 | 1.06 | 1 | 1 | 1 |  | 0 | 1 | 2 | 2 | 3 |
| 6204232330 | 3.49 | 1.15 |  | 1.8 | 0.77 | 0 | 1 | 1 |  | 0 | 1 | 1 | 1 | 1 |
| 6204232331 | 4.18 | 1.13 |  | 2.52 | 1.16 | 1 | 1 | 1 |  | 0 | 0 | 2 | 1 | 2 |
| 6204232334 | 3.51 | 1.3 |  | 1.79 | 1.07 | 1 | 0 | 0 |  | 0 | 0 | 2 | 2 | 1 |
| 6204232335 | 5.1 | 1.3 |  | 2.69 | 1.59 | 1 | 0 | 0 |  | 0 | 0 | 2 | 1 | 2 |
| 6204232336 | 5.24 | 1.3 |  | 3.46 | 1.39 | 1 | 1 | 0 |  | 1 | 1 | 1 | 1 | 1 |
| 6204232338 | 3.57 | 1.25 |  | 1.99 | 0.72 | 0 | 1 | 1 |  | 0 | 1 | 1 | 1 | 1 |
| 6204232339 | 3.21 | 0.97 |  | 1.8 | 0.99 | 0 | 0 | 1 |  | 0 | 0 | 1 | 1 | 1 |
| 6204232342 | 6.44 | 1.33 |  | 4.42 | 2.26 | 0 | 0 | 1 |  | 0 | 0 | 2 | 2 | 3 |
| 6204232344 | 3.93 | 1.54 |  | 1.89 | 0.65 | 1 | 0 | 0 |  | 0 | 0 | 1 | 1 | 1 |
| 6204232347 | 3.62 | 0.85 |  | 2.51 | 0.75 | 0 | 1 | 2 |  | 0 | 0 | 2 | 2 | 2 |
| 6204232348 | 4.19 | 1.12 |  | 2.53 | 1.44 | 2 | 1 | 1 |  | 0 | 1 | 1 | 1 | 1 |
| 6204232349 | 2.51 | 0.83 |  | 1.33 | 1.27 | 0 | 0 | 0 |  | 0 | 0 | 1 | 1 | 1 |
| 6204232351 | 5.65 | 1.08 |  | 3.69 | 2.14 | 0 | 0 | 1 |  | 0 | 0 | 3 | 2 | 2 |
| 6204232352 | 4.16 | 1.07 |  | 2.63 | 0.9 | 0 | 1 | 1 |  | 0 | 0 | 1 | 1 | 1 |
| 6204232353 | 4.51 | 1.15 |  | 3.14 | 0.73 | 0 | 0 | 1 |  | 0 | 0 | 2 | 2 | 1 |
| 6204232354 | 4.37 | 1.09 |  | 2.76 | 0.88 | 2 | 1 | 0 |  | 0 | 1 | 1 | 1 | 1 |
| 6204233101 | 6.09 | 0.96 |  | 3.58 | 2.62 | 1 | 0 | 1 |  | 0 | 0 | 2 | 2 | 2 |
| 6204233102 | 2.92 | 0.91 |  | 1.74 | 0.7 | 0 | 0 | 2 |  | 0 | 0 | 2 | 2 | 2 |
| 6204233103 | 4.49 | 0.75 |  | 2.93 | 2.7 | 0 | 1 | 1 |  | 0 | 0 | 3 | 2 | 3 |
| 6204233104 | 4.36 | 1 |  | 2.87 | 1.66 | 0 | 1 | 2 |  | 0 | 0 | 2 | 2 | 2 |
| 6204233106 | 4.14 | 1.13 |  | 2.48 | 0.82 | 0 | 0 | 2 |  | 0 | 0 | 1 | 1 | 1 |
| 6204233107 | 3.63 | 1.2 |  | 2.06 | 0.9 | 1 | 0 | 0 |  | 0 | 0 | 1 | 1 | 1 |
| 6204233108 | 3.48 | 0.89 |  | 1.82 | 1.74 | 0 | 0 | 1 |  | 0 | 0 | 2 | 1 | 3 |
| 6204233109 | 4.12 | 1.61 |  | 2.07 | 0.63 | 0 | 0 | 2 |  | 0 | 0 | 1 | 1 | 1 |
| 6204233111 | 6.5 | 0.94 |  | 4.34 | 4.36 | 0 | 1 | 1 |  | 0 | 1 | 2 | 2 | 2 |
| 6204233113 | 3.43 | 1.18 |  | 1.72 | 1.08 | 0 | 1 | 1 |  | 0 | 0 | 1 | 1 | 1 |
| 6204233117 | 3.71 | 1.44 |  | 1.77 | 1.67 | 2 | 0 | 0 |  | 0 | 0 | 1 | 1 | 1 |
| 6204233119 | 3.27 | 0.93 |  | 1.97 | 1.06 | 0 | 1 | 1 |  | 0 | 0 | 1 | 1 | 1 |
| 6204233121 | 4.14 | 1.01 |  | 2.27 | 2.97 | 0 | 1 | 1 |  | 0 | 1 | 3 | 3 | 3 |
| 6204233126 | 3.27 | 0.94 |  | 1.77 | 1.07 | 1 | 0 | 0 |  | 0 | 0 | 1 | 1 | 1 |
| 6204233130 | 4.41 | 1.26 |  | 2.89 | 0.55 | 0 | 0 | 2 |  | 0 | 0 | 1 | 1 | 1 |
| 6204233131 | 4.73 | 1.14 |  | 2.73 | 1.54 | 2 | 0 | 0 |  | 0 | 0 | 2 | 2 | 2 |
| 6204233132 | 4.38 | 1.63 |  | 2.13 | 0.98 | 1 | 0 | 0 |  | 0 | 0 | 1 | 1 | 1 |
| 6204233133 | 3.22 | 1.18 |  | 1.33 | 1.72 | 0 | 0 | 2 |  | 0 | 0 | 2 | 1 | 1 |
| 6204233135 | 3.41 | 0.87 |  | 1.93 | 1.65 | 1 | 1 | 1 |  | 1 | 0 | 3 | 2 | 3 |
| 6204233138 | 3.62 | 0.76 |  | 2.3 | 1.68 | 0 | 1 | 2 |  | 0 | 1 | 1 | 1 | 1 |
| 6204233140 | 4.7 | 1.44 |  | 2.69 | 0.97 | 2 | 0 | 1 |  | 0 | 0 | 2 | 1 | 2 |
| 6204233141 | 4.25 | 0.88 |  | 2.59 | 2 | 0 | 1 | 2 |  | 0 | 1 | 2 | 2 | 2 |
| 6204233145 | 3.55 | 1.22 |  | 2.05 | 0.46 | 0 | 0 | 2 |  | 0 | 0 | 1 | 1 | 1 |
| 6204233148 | 3.87 | 1.31 |  | 2.25 | 0.46 | 0 | 0 | 2 |  | 0 | 0 | 1 | 1 | 1 |
| 6204233150 | 4.26 | 1.14 |  | 2.65 | 1.08 | 0 | 0 | 1 |  | 0 | 0 | 2 | 1 | 1 |
| 6204233152 | 2.85 | 1.32 |  | 1.08 | 0.96 | 2 | 1 | 2 |  | 0 | 0 | 2 | 2 | 2 |
| 6204233154 | 3.21 | 1.05 |  | 1.79 | 0.74 | 0 | 0 | 1 |  | 0 | 0 | 1 | 1 | 2 |
| 6204233201 | 4.24 | 1.48 |  | 2.3 | 0.71 | 2 | 1 | 0 |  | 0 | 0 | 1 | 1 | 1 |
| 6204233205 | 3.45 | 1.28 |  | 1.77 | 0.71 | 0 | 0 | 2 |  | 0 | 0 | 1 | 1 | 1 |
| 6204233210 | 5.23 | 1.02 |  | 3.78 | 0.84 | 1 | 1 | 2 |  | 0 | 1 | 1 | 1 | 1 |
| 6204233211 | 4.13 | 0.78 |  | 2.47 | 2.79 | 0 | 1 | 1 |  | 1 | 1 | 3 | 3 | 3 |
| 6204233213 | 4.65 | 0.97 |  | 3.04 | 1.4 | 0 | 0 | 1 |  | 0 | 0 | 1 | 1 | 2 |
| 6204233214 | 3.64 | 0.83 |  | 2.03 | 1.94 | 0 | 1 | 2 |  | 0 | 0 | 1 | 1 | 1 |
| 6204233215 | 3.26 | 0.84 |  | 2.07 | 0.73 | 0 | 0 | 2 |  | 0 | 0 | 2 | 1 | 1 |
| 6204233217 | 4.74 | 1.62 |  | 2.5 | 1.08 | 1 | 1 | 1 |  | 0 | 0 | 1 | 1 | 1 |
| 6204233218 | 3.61 | 0.8 |  | 2.36 | 1.83 | 2 | 1 | 2 |  | 0 | 1 | 2 | 1 | 2 |
| 6204233219 | 3.41 | 1.09 |  | 1.87 | 1 | 1 | 1 | 2 |  | 0 | 0 | 1 | 1 | 1 |
| 6204233220 | 4.14 | 1.26 |  | 2.42 | 1.06 | 1 | 0 | 2 |  | 0 | 0 | 2 | 2 | 2 |
| 6204233221 | 2.66 | 0.96 |  | 1.56 | 0.36 | 0 | 0 | 2 |  | 0 | 0 | 1 | 1 | 1 |
| 6204233223 | 2.57 | 0.89 |  | 1.16 | 1.19 | 0 | 0 | 1 |  | 0 | 0 | 1 | 1 | 1 |
| 6204233224 | 3.15 | 1.28 |  | 1.48 | 0.38 | 0 | 0 | 2 |  | 0 | 0 | 1 | 1 | 1 |
| 6204233225 | 3.57 | 0.93 |  | 2.28 | 0.99 | 1 | 1 | 0 |  | 0 | 1 | 2 | 2 | 2 |
| 6204233227 | 3.61 | 0.79 |  | 1.79 | 2.27 | 0 | 1 | 2 |  | 0 | 0 | 2 | 2 | 2 |
| 6204233228 | 4.88 | 1.08 |  | 3.45 | 1.16 | 0 | 0 | 2 |  | 0 | 0 | 2 | 1 | 1 |
| 6204233230 | 3.77 | 1.22 |  | 2.2 | 0.96 | 0 | 0 | 2 |  | 0 | 0 | 2 | 1 | 1 |
| 6204233231 | 2.91 | 1.1 |  | 1.45 | 0.7 | 0 | 0 | 2 |  | 0 | 0 | 1 | 1 | 1 |
| 6204233234 | 3.91 | 0.93 |  | 2.62 | 1.19 | 0 | 1 | 2 |  | 1 | 0 | 2 | 2 | 1 |
| 6204233236 | 3.1 | 1.04 |  | 1.67 | 0.76 | 0 | 0 | 2 |  | 0 | 0 | 1 | 1 | 1 |
| 6204233238 | 3.98 | 1.03 |  | 2.39 | 1.25 | 1 | 0 | 0 |  | 0 | 0 | 2 | 2 | 3 |
| 6204233240 | 3.61 | 1.53 |  | 1.72 | 0.5 | 0 | 0 | 2 |  | 0 | 0 | 1 | 1 | 1 |
| 6204233241 | 4.29 | 0.96 |  | 2.6 | 1.78 | 1 | 1 | 2 |  | 0 | 0 | 1 | 1 | 1 |
| 6204233243 | 3.59 | 0.74 |  | 1.88 | 1.5 | 2 | 1 | 1 |  | 0 | 1 | 1 | 1 | 1 |
| 6204233245 | 3.37 | 1.02 |  | 1.92 | 1.02 | 0 | 0 | 2 |  | 0 | 0 | 2 | 2 | 2 |
| 6204233246 | 3.27 | 1.19 |  | 1.69 | 0.84 | 0 | 0 | 2 |  | 0 | 0 | 2 | 1 | 2 |
| 6204233248 | 3.81 | 1.15 |  | 2.26 | 1.25 | 1 | 0 | 1 |  | 0 | 0 | 2 | 2 | 2 |
| 6204233249 | 3.39 | 1.43 |  | 1.5 | 0.79 | 0 | 0 | 2 |  | 0 | 0 | 1 | 1 | 1 |
| 6204233250 | 3.3 | 0.88 |  | 1.66 | 1.76 | 0 | 1 | 2 |  | 0 | 0 | 2 | 2 | 2 |
| 6204233251 | 2.88 | 0.78 |  | 1.45 | 1.97 | 0 | 1 | 1 |  | 1 | 1 | 3 | 2 | 2 |
| 6204233253 | 2.85 | 0.84 |  | 1.52 | 1.28 | 0 | 0 | 2 |  | 0 | 0 | 2 | 2 | 2 |
| 6204233301 | 2.98 | 0.76 |  | 1.97 | 0.48 | 0 | 1 | 1 |  | 1 | 1 | 2 | 2 | 2 |
| 6204233303 | 3.74 | 0.94 |  | 2.19 | 1.22 | 0 | 0 | 0 |  | 0 | 0 | 2 | 2 | 2 |
| 6204233304 | 4.57 | 1.11 |  | 1.81 | 2.17 | 1 | 0 | 1 |  | 0 | 0 | 1 | 1 | 1 |
| 6204233305 | 4.45 | 1.7 |  | 2.34 | 0.74 | 0 | 0 | 1 |  | 0 | 0 | 1 | 1 | 1 |
| 6204233306 | 4 | 1.61 |  | 1.82 | 1.02 | 0 | 1 | 2 |  | 1 | 1 | 1 | 1 | 1 |
| 6204233307 | 4.8 | 1.62 |  | 2.5 | 1.29 | 1 | 1 | 0 |  | 0 | 1 | 1 | 1 | 1 |
| 6204233308 | 3.55 | 1.2 |  | 1.85 | 0.72 | 0 | 1 | 0 |  | 0 | 0 | 2 | 2 | 2 |
| 6204233309 | 4.66 | 1.2 |  | 2.8 | 1.34 | 1 | 1 | 2 |  | 0 | 1 | 1 | 1 | 1 |
| 6204233312 | 3.18 | 1.24 |  | 1.64 | 0.49 | 0 | 0 | 1 |  | 0 | 0 | 1 | 1 | 1 |
| 6204233313 | 4.62 | 1.39 |  | 2.53 | 1.25 | 1 | 0 | 0 |  | 0 | 0 | 1 | 1 | 1 |
| 6204233314 | 3.4 | 1.12 |  | 1.75 | 0.67 | 0 | 0 | 1 |  | 0 | 0 | 2 | 2 | 1 |
| 6204233316 | 4.7 | 1.18 |  | 3.18 | 0.71 | 0 | 0 | 2 |  | 0 | 0 | 1 | 1 | 1 |
| 6204233318 | 6.23 | 1.51 |  | 3.88 | 0.91 | 0 | 1 | 0 |  | 0 | 0 | 1 | 1 | 1 |
| 6204233319 | 3.97 | 1.23 |  | 2.44 | 0.56 | 0 | 0 | 2 |  | 0 | 0 | 1 | 1 | 1 |
| 6204233320 | 3.92 | 1.11 |  | 2.51 | 1.07 | 0 | 0 | 0 |  | 0 | 0 | 1 | 1 | 1 |
| 6204233321 | 2.84 | 0.92 |  | 1.38 | 1.13 | 0 | 0 | 0 |  | 0 | 0 | 2 | 2 | 1 |
| 6204233323 | 3.47 | 0.94 |  | 2.23 | 0.98 | 1 | 1 | 1 |  | 0 | 1 | 1 | 1 | 1 |
| 6204233324 | 3.02 | 0.96 |  | 1.57 | 1.32 | 1 | 0 | 1 |  | 0 | 0 | 2 | 1 | 1 |
| 6204233325 | 3.72 | 0.88 |  | 2.07 | 1.82 | 0 | 1 | 2 |  | 0 | 1 | 1 | 1 | 1 |
| 6204233326 | 4.33 | 0.98 |  | 2.65 | 1.64 | 1 | 1 | 2 |  | 0 | 1 | 1 | 1 | 1 |
| 6204233327 | 3.91 | 1.07 |  | 2.42 | 0.97 | 2 | 0 | 0 |  | 0 | 0 | 2 | 2 | 2 |
| 6204233330 | 5.23 | 1.59 |  | 3.13 | 1.39 | 0 | 1 | 1 |  | 1 | 1 | 1 | 1 | 1 |
| 6204233332 | 3.71 | 0.95 |  | 2.29 | 0.99 | 2 | 1 | 1 |  | 0 | 0 | 1 | 1 | 1 |
| 6204233333 | 3.46 | 1.3 |  | 1.66 | 0.72 | 1 | 0 | 1 |  | 0 | 0 | 1 | 1 | 1 |
| 6204233335 | 4.1 | 1.64 |  | 1.95 | 0.99 | 0 | 0 | 2 |  | 0 | 0 | 1 | 1 | 1 |
| 6204233336 | 3.5 | 1.43 |  | 1.71 | 0.42 | 0 | 0 | 1 |  | 0 | 0 | 1 | 1 | 1 |
| 6204233337 | 3.81 | 0.93 |  | 2.12 | 2.21 | 0 | 0 | 0 |  | 0 | 0 | 2 | 1 | 1 |
| 6204233340 | 5.94 | 0.91 |  | 3.13 | 4.48 | 1 | 1 | 1 |  | 0 | 1 | 3 | 2 | 3 |
| 6204233342 | 3.45 | 0.91 |  | 1.73 | 2.27 | 1 | 0 | 1 |  | 0 | 0 | 1 | 1 | 1 |
| 6204233343 | 4.75 | 1.15 |  | 2.88 | 1.06 | 0 | 0 | 1 |  | 0 | 0 | 1 | 1 | 1 |
| 6204233344 | 4.38 | 1.05 |  | 2.58 | 2.34 | 0 | 0 | 0 |  | 0 | 0 | 1 | 1 | 1 |
| 6204233345 | 4.14 | 1.53 |  | 1.87 | 1.37 | 0 | 0 | 1 |  | 0 | 0 | 1 | 1 | 1 |
| 6204233346 | 5.06 | 2.31 |  | 2 | 1.28 | 1 | 0 | 0 |  | 0 | 0 | 1 | 1 | 1 |
| 6204233348 | 4.29 | 1.15 |  | 2.7 | 0.78 | 1 | 1 | 1 |  | 0 | 1 | 1 | 1 | 1 |
| 6204233349 | 3.55 | 1.37 |  | 1.76 | 1.31 | 0 | 1 | 1 |  | 0 | 1 | 1 | 1 | 1 |
| 6204233350 | 4.6 | 1.38 |  | 2.69 | 0.95 | 0 | 0 | 0 |  | 0 | 0 | 2 | 1 | 2 |
| 6204234102 | 3.77 | 1.07 |  | 2.23 | 0.92 | 1 | 0 | 1 |  | 0 | 0 | 1 | 1 | 2 |
| 6204234103 | 4.61 | 1.22 |  | 2.84 | 1.35 | 1 | 0 | 1 |  | 0 | 0 | 2 | 2 | 1 |
| 6204234104 | 2.83 | 1.03 |  | 1.32 | 0.96 | 0 | 0 | 0 |  | 0 | 0 | 1 | 1 | 1 |
| 6204234105 | 4.63 | 1.23 |  | 2.63 | 1.16 | 1 | 1 | 0 |  | 0 | 1 | 1 | 1 | 1 |
| 6204234106 | 3.6 | 1.21 |  | 1.75 | 1.25 | 2 | 1 | 1 |  | 0 | 1 | 1 | 1 | 1 |
| 6204234109 | 4.28 | 1.35 |  | 2.03 | 2.45 | 0 | 0 | 0 |  | 0 | 0 | 2 | 2 | 1 |
| 6204234110 | 4.02 | 1.33 |  | 2.01 | 1.25 | 0 | 0 | 0 |  | 0 | 0 | 2 | 2 | 1 |
| 6204234111 | 2.79 | 0.78 |  | 1.5 | 1.4 | 0 | 0 | 0 |  | 0 | 0 | 1 | 1 | 1 |
| 6204234113 | 3.68 | 1.08 |  | 2.03 | 1.32 | 0 | 1 | 0 |  | 0 | 1 | 1 | 1 | 1 |
| 6204234115 | 5.4 | 1.22 |  | 3.35 | 1.65 | 1 | 1 | 0 |  | 0 | 0 | 1 | 1 | 1 |
| 6204234116 | 3.2 | 0.86 |  | 1.76 | 1.62 | 1 | 1 | 2 |  | 0 | 1 | 2 | 1 | 2 |
| 6204234117 | 4.71 | 1.36 |  | 2.69 | 2.01 | 0 | 1 | 2 |  | 0 | 1 | 2 | 1 | 2 |
| 6204234118 | 3.24 | 1.76 |  | 0.95 | 0.85 | 1 | 0 | 0 |  | 0 | 0 | 1 | 1 | 1 |
| 6204234119 | 4.15 | 1.3 |  | 2.19 | 1.81 | 0 | 0 | 1 |  | 0 | 0 | 1 | 1 | 1 |
| 6204234120 | 3.8 | 1.49 |  | 1.94 | 0.5 | 1 | 0 | 0 |  | 0 | 0 | 2 | 2 | 1 |
| 6204234122 | 3.69 | 1.88 |  | 1.17 | 0.68 | 0 | 1 | 1 |  | 0 | 1 | 1 | 1 | 1 |
| 6204234125 | 3.28 | 1.01 |  | 1.9 | 0.69 | 1 | 1 | 1 |  | 0 | 1 | 3 | 2 | 2 |
| 6204234126 | 3.36 | 1.28 |  | 1.46 | 1.34 | 0 | 0 | 0 |  | 0 | 0 | 1 | 1 | 1 |
| 6204234128 | 4.83 | 1.41 |  | 2.37 | 2.42 | 1 | 0 | 0 |  | 0 | 0 | 1 | 1 | 1 |
| 6204234129 | 3.41 | 1.18 |  | 1.79 | 1.07 | 1 | 1 | 0 |  | 0 | 0 | 1 | 1 | 1 |
| 6204234130 | 4.74 | 1.56 |  | 2.8 | 0.84 | 2 | 1 | 0 |  | 0 | 1 | 1 | 1 | 2 |
| 6204234131 | 3.55 | 0.73 |  | 2.34 | 1.21 | 0 | 1 | 0 |  | 0 | 1 | 2 | 2 | 2 |
| 6204234132 | 3.6 | 1.49 |  | 1.56 | 0.75 | 2 | 1 | 1 |  | 1 | 0 | 1 | 1 | 1 |
| 6204234134 | 5.45 | 1.49 |  | 3.17 | 1.31 | 0 | 0 | 0 |  | 0 | 0 | 1 | 1 | 1 |
| 6204234136 | 3.1 | 1.13 |  | 1.43 | 1.52 | 0 | 0 | 1 |  | 0 | 0 | 1 | 1 | 1 |
| 6204234137 | 6.21 | 0.92 |  | 4.11 | 2.95 | 1 | 1 | 2 |  | 0 | 1 | 2 | 1 | 2 |
| 6204234138 | 2.95 | 1.36 |  | 1.12 | 1.16 | 0 | 1 | 1 |  | 0 | 1 | 1 | 1 | 1 |
| 6204234140 | 4.03 | 1.25 |  | 2.01 | 1.5 | 1 | 0 | 0 |  | 0 | 0 | 2 | 2 | 1 |
| 6204234143 | 3.04 | 1.09 |  | 1.42 | 1.07 | 1 | 0 | 1 |  | 0 | 0 | 2 | 1 | 1 |
| 6204234145 | 3.75 | 0.95 |  | 2.15 | 1.66 | 2 | 1 | 0 |  | 0 | 1 | 2 | 2 | 2 |
| 6204234146 | 3.55 | 1.29 |  | 1.84 | 0.88 | 0 | 1 | 1 |  | 0 | 0 | 1 | 1 | 1 |
| 6204234147 | 4.16 | 1.36 |  | 2.31 | 1.03 | 1 | 1 | 0 |  | 0 | 1 | 1 | 1 | 1 |
| 6204234148 | 3.84 | 1.14 |  | 2.15 | 1.28 | 2 | 1 | 1 |  | 0 | 1 | 2 | 1 | 2 |
| 6204234149 | 4.47 | 1.46 |  | 2.38 | 1.88 | 1 | 0 | 0 |  | 0 | 0 | 1 | 1 | 1 |
| 6204234150 | 3.54 | 1.37 |  | 1.54 | 1.26 | 1 | 0 | 0 |  | 0 | 0 | 1 | 1 | 1 |
| 6204234151 | 3.77 | 1.62 |  | 1.8 | 0.68 | 1 | 1 | 1 |  | 0 | 0 | 1 | 1 | 1 |
| 6204234152 | 3.11 | 0.95 |  | 1.6 | 1.24 | 1 | 1 | 1 |  | 0 | 1 | 1 | 1 | 1 |
| 6204234201 | 4.14 | 1.38 |  | 2.43 | 0.57 | 1 | 0 | 1 |  | 0 | 0 | 1 | 1 | 1 |
| 6204234205 | 5.31 | 0.92 |  | 3.66 | 2.6 | 1 | 1 | 1 |  | 0 | 1 | 2 | 2 | 2 |
| 6204234206 | 3.78 | 1.35 |  | 2.11 | 0.63 | 1 | 1 | 1 |  | 0 | 1 | 1 | 1 | 1 |
| 6204234209 | 3.07 | 0.93 |  | 1.88 | 0.53 | 1 | 0 | 1 |  | 0 | 0 | 1 | 1 | 1 |
| 6204234212 | 4.28 | 0.81 |  | 2.72 | 1.99 | 2 | 1 | 0 |  | 0 | 0 | 3 | 3 | 3 |
| 6204234213 | 3.34 | 1.8 |  | 0.97 | 0.84 | 1 | 1 | 0 |  | 0 | 0 | 1 | 1 | 1 |
| 6204234214 | 2.25 | 1.11 |  | 0.59 | 0.69 | 0 | 1 | 2 |  | 0 | 1 | 2 | 1 | 2 |
| 6204234215 | 3.95 | 1.27 |  | 2.24 | 0.82 | 1 | 1 | 2 |  | 0 | 0 | 3 | 2 | 2 |
| 6204234216 | 4 | 1.29 |  | 2.36 | 0.68 | 0 | 0 | 0 |  | 0 | 0 | 3 | 2 | 2 |
| 6204234221 | 5.35 | 1.22 |  | 3.47 | 1.14 | 2 | 1 | 1 |  | 0 | 1 | 1 | 1 | 1 |
| 6204234222 | 4.16 | 0.94 |  | 2.6 | 1.19 | 0 | 1 | 2 |  | 0 | 0 | 1 | 1 | 1 |
| 6204234223 | 3.17 | 1.16 |  | 1.68 | 0.73 | 0 | 1 | 1 |  | 1 | 1 | 1 | 1 | 1 |
| 6204234225 | 3.41 | 1.2 |  | 1.77 | 0.94 | 0 | 0 | 1 |  | 0 | 0 | 1 | 1 | 1 |
| 6204234226 | 2.86 | 0.79 |  | 1.71 | 0.74 | 0 | 0 | 1 |  | 0 | 0 | 1 | 1 | 1 |
| 6204234227 | 3.99 | 0.9 |  | 2.56 | 1.29 | 1 | 1 | 1 |  | 0 | 0 | 1 | 1 | 1 |
| 6204234228 | 3.37 | 0.67 |  | 2.35 | 0.93 | 1 | 1 | 0 |  | 0 | 1 | 1 | 1 | 1 |
| 6204234232 | 3.46 | 0.9 |  | 1.91 | 1.23 | 1 | 0 | 1 |  | 0 | 0 | 2 | 2 | 2 |
| 6204234234 | 4.43 | 1.14 |  | 2.71 | 1.06 | 2 | 0 | 0 |  | 0 | 0 | 3 | 2 | 3 |
| 6204234235 | 3.49 | 1.03 |  | 2.04 | 0.82 | 0 | 1 | 1 |  | 0 | 1 | 1 | 1 | 1 |
| 6204234237 | 3.84 | 1.28 |  | 1.94 | 0.93 | 1 | 1 | 1 |  | 0 | 1 | 1 | 1 | 1 |
| 6204234238 | 4.15 | 1.37 |  | 2.36 | 0.63 | 1 | 1 | 2 |  | 0 | 1 | 1 | 1 | 1 |
| 6204234239 | 4.4 | 0.87 |  | 2.81 | 1.34 | 2 | 0 | 0 |  | 0 | 0 | 1 | 1 | 1 |
| 6204234240 | 2.53 | 0.66 |  | 1.31 | 1.26 | 0 | 0 | 1 |  | 0 | 0 | 2 | 2 | 2 |
| 6204234241 | 5.15 | 1.3 |  | 3.35 | 1.76 | 0 | 0 | 1 |  | 0 | 0 | 3 | 3 | 3 |
| 6204234242 | 4.43 | 0.88 |  | 3.18 | 1.1 | 1 | 0 | 0 |  | 0 | 0 | 2 | 2 | 2 |
| 6204234243 | 4.56 | 1.01 |  | 3.03 | 0.93 | 2 | 1 | 1 |  | 0 | 1 | 1 | 1 | 1 |
| 6204234244 | 5.2 | 0.96 |  | 3.13 | 2.74 | 1 | 1 | 1 |  | 0 | 0 | 1 | 1 | 1 |
| 6204234245 | 3.43 | 1.42 |  | 1.67 | 0.67 | 0 | 1 | 1 |  | 0 | 1 | 1 | 1 | 1 |
| 6204234248 | 4.27 | 0.86 |  | 2.65 | 1.5 | 2 | 0 | 0 |  | 0 | 0 | 3 | 3 | 3 |
| 6204234249 | 3.25 | 1.2 |  | 1.57 | 0.7 | 1 | 0 | 0 |  | 0 | 0 | 1 | 1 | 1 |
| 6204234250 | 3.93 | 0.82 |  | 2.48 | 1.35 | 1 | 0 | 0 |  | 0 | 0 | 2 | 1 | 2 |
| 6204234251 | 3.52 | 0.84 |  | 2.04 | 1.93 | 0 | 1 | 1 |  | 1 | 1 | 2 | 1 | 2 |
| 6204234252 | 3.68 | 0.92 |  | 2.25 | 1.14 | 1 | 0 | 0 |  | 0 | 0 | 2 | 2 | 2 |
| 6204234255 | 3.55 | 0.92 |  | 2.03 | 1.11 | 2 | 0 | 0 |  | 0 | 1 | 1 | 1 | 1 |
| 6204234301 | 3.69 | 0.9 |  | 1.47 | 2.71 | 1 | 1 | 2 |  | 0 | 1 | 3 | 2 | 3 |
| 6204234304 | 3.17 | 0.84 |  | 1.87 | 1.08 | 1 | 1 | 0 |  | 1 | 1 | 1 | 1 | 1 |
| 6204234305 | 3.14 | 1.14 |  | 1.48 | 1.1 | 1 | 1 | 0 |  | 0 | 1 | 1 | 1 | 1 |
| 6204234306 | 5.44 | 1.09 |  | 3.38 | 1.7 | 1 | 0 | 0 |  | 0 | 0 | 2 | 2 | 2 |
| 6204234307 | 3.17 | 1 |  | 1.59 | 1.55 | 0 | 0 | 0 |  | 0 | 0 | 1 | 1 | 3 |
| 6204234308 | 4.45 | 1.69 |  | 2.06 | 0.62 | 1 | 0 | 0 |  | 0 | 0 | 1 | 1 | 1 |
| 6204234309 | 2.96 | 0.9 |  | 1.74 | 0.7 | 0 | 1 | 1 |  | 0 | 1 | 1 | 1 | 1 |
| 6204234310 | 3.39 | 0.88 |  | 2.07 | 0.78 | 2 | 1 | 1 |  | 0 | 1 | 1 | 1 | 1 |
| 6204234311 | 4.18 | 1.21 |  | 2.41 | 1.5 | 0 | 0 | 1 |  | 0 | 0 | 1 | 1 | 1 |
| 6204234313 | 3.57 | 0.86 |  | 1.82 | 2.75 | 1 | 1 | 0 |  | 1 | 1 | 1 | 1 | 1 |
| 6204234319 | 3.18 | 0.96 |  | 1.74 | 1.01 | 0 | 1 | 1 |  | 0 | 1 | 2 | 2 | 3 |
| 6204234320 | 3.37 | 0.99 |  | 2.01 | 0.96 | 0 | 0 | 1 |  | 0 | 0 | 3 | 2 | 2 |
| 6204234322 | 3.55 | 0.81 |  | 2.49 | 0.6 | 2 | 1 | 0 |  | 0 | 1 | 1 | 1 | 2 |
| 6204234323 | 3.11 | 1.15 |  | 1.45 | 0.56 | 1 | 1 | 1 |  | 0 | 1 | 1 | 1 | 1 |
| 6204234324 | 3.93 | 1.18 |  | 2.29 | 0.41 | 0 | 1 | 0 |  | 0 | 1 | 1 | 1 | 1 |
| 6204234328 | 3.67 | 1.4 |  | 1.69 | 0.66 | 1 | 1 | 0 |  | 0 | 1 | 1 | 1 | 1 |
| 6204234329 | 3.35 | 0.93 |  | 1.71 | 1.55 | 0 | 0 | 1 |  | 0 | 0 | 1 | 1 | 2 |
| 6204234330 | 3.81 | 1.09 |  | 2.15 | 1.13 | 1 | 0 | 0 |  | 0 | 0 | 1 | 1 | 2 |
| 6204234331 | 3.78 | 0.87 |  | 2.01 | 1.99 | 0 | 0 | 1 |  | 0 | 0 | 3 | 3 | 3 |
| 6204234333 | 3.33 | 0.96 |  | 1.82 | 1.17 | 1 | 1 | 2 |  | 0 | 1 | 1 | 1 | 1 |
| 6204234334 | 3.99 | 0.93 |  | 2.11 | 1.35 | 1 | 1 | 1 |  | 0 | 1 | 1 | 1 | 1 |
| 6204234335 | 3.21 | 0.81 |  | 1.73 | 1.88 | 0 | 1 | 2 |  | 1 | 1 | 2 | 2 | 3 |
| 6204234336 | 4.22 | 1.06 |  | 2.31 | 2.68 | 1 | 1 | 1 |  | 0 | 0 | 2 | 2 | 2 |
| 6204234339 | 2.92 | 0.9 |  | 1.7 | 0.55 | 0 | 1 | 0 |  | 1 | 0 | 1 | 1 | 1 |
| 6204234340 | 2.98 | 1.08 |  | 1.48 | 0.62 | 0 | 0 | 1 |  | 0 | 0 | 1 | 1 | 1 |
| 6204234342 | 2.3 | 0.6 |  | 1.3 | 0.93 | 1 | 1 | 0 |  | 1 | 1 | 1 | 1 | 1 |
| 6204234343 | 4.63 | 1.34 |  | 2.92 | 1.31 | 0 | 1 | 0 |  | 0 | 1 | 1 | 1 | 1 |
| 6204234346 | 3.47 | 0.76 |  | 2.44 | 0.55 | 0 | 1 | 0 |  | 0 | 1 | 1 | 1 | 2 |
| 6204234347 | 5.71 | 1.06 |  | 4.29 | 1.09 | 1 | 0 | 0 |  | 0 | 0 | 2 | 2 | 1 |
| 6204234348 | 3.43 | 0.87 |  | 1.69 | 2.05 | 0 | 0 | 1 |  | 0 | 0 | 2 | 1 | 2 |
| 6204234349 | 3.61 | 0.8 |  | 2.06 | 2.04 | 0 | 0 | 0 |  | 0 | 0 | 3 | 3 | 3 |
| 6205031101 | 2.87 | 0.72 |  | 1.68 | 0.85 | 0 | 1 | 2 |  | 0 | 1 | 2 | 2 | 2 |
| 6205031102 | 1.68 | 0.52 |  | 0.81 | 0.56 | 0 | 1 | 2 |  | 0 | 0 | 1 | 1 | 1 |
| 6205031103 | 2.81 | 0.78 |  | 1.47 | 0.93 | 1 | 0 | 0 |  | 0 | 0 | 1 | 1 | 1 |
| 6205031106 | 3.58 | 0.87 |  | 1.7 | 2.43 | 0 | 0 | 1 |  | 0 | 0 | 1 | 1 | 1 |
| 6205031109 | 2.96 | 0.81 |  | 1.69 | 0.95 | 1 | 1 | 1 |  | 0 | 1 | 1 | 1 | 1 |
| 6205031113 | 3.53 | 0.99 |  | 2.22 | 0.8 | 0 | 0 | 0 |  | 0 | 0 | 2 | 2 | 2 |
| 6205031115 | 5.08 | 1.19 |  | 2.4 | 3.72 | 1 | 1 | 2 |  | 0 | 1 | 1 | 1 | 1 |
| 6205031116 | 3.55 | 0.66 |  | 1.47 | 3.44 | 0 | 1 | 1 |  | 0 | 1 | 2 | 2 | 2 |
| 6205031118 | 3.12 | 0.88 |  | 1.77 | 1.17 | 0 | 1 | 2 |  | 1 | 1 | 1 | 1 | 1 |
| 6205031120 | 3.23 | 0.88 |  | 1.52 | 1.44 | 0 | 1 | 2 |  | 0 | 0 | 1 | 1 | 1 |
| 6205031121 | 4.58 | 1.13 |  | 2.85 | 1.42 | 1 | 0 | 0 |  | 0 | 0 | 1 | 1 | 2 |
| 6205031122 | 4.27 | 1.38 |  | 2.36 | 1.32 | 0 | 0 | 2 |  | 0 | 0 | 2 | 1 | 2 |
| 6205031126 | 3.86 | 0.85 |  | 1.99 | 2.36 | 0 | 1 | 2 |  | 1 | 0 | 1 | 1 | 1 |
| 6205031127 | 2.82 | 0.79 |  | 1.43 | 1.19 | 0 | 0 | 0 |  | 0 | 0 | 1 | 1 | 1 |
| 6205031129 | 3.09 | 1.11 |  | 1.37 | 0.71 | 0 | 0 | 2 |  | 0 | 0 | 2 | 1 | 1 |
| 6205031130 | 3.12 | 0.94 |  | 1.41 | 1.4 | 0 | 0 | 1 |  | 0 | 0 | 1 | 1 | 1 |
| 6205031134 | 4.83 | 1.03 |  | 2.32 | 3.77 | 0 | 1 | 1 |  | 0 | 1 | 3 | 2 | 3 |
| 6205031136 | 4.04 | 1.11 |  | 2.45 | 1.37 | 0 | 0 | 2 |  | 0 | 0 | 3 | 2 | 3 |
| 6205031138 | 3.64 | 0.74 |  | 2.42 | 0.93 | 0 | 0 | 1 |  | 0 | 0 | 1 | 1 | 2 |
| 6205031142 | 5.39 | 1.2 |  | 3.76 | 0.9 | 1 | 1 | 1 |  | 0 | 0 | 1 | 1 | 1 |
| 6205031145 | 2.79 | 0.81 |  | 1.45 | 1.08 | 0 | 0 | 2 |  | 0 | 0 | 1 | 1 | 1 |
| 6205031149 | 4.08 | 1.12 |  | 2.33 | 1.37 | 0 | 1 | 2 |  | 0 | 1 | 1 | 1 | 1 |
| 6205031150 | 2.71 | 0.99 |  | 1.26 | 0.77 | 1 | 0 | 1 |  | 0 | 0 | 2 | 2 | 2 |
| 6205031201 | 2.72 | 0.76 |  | 1.6 | 0.82 | 0 | 1 | 0 |  | 0 | 1 | 1 | 1 | 2 |
| 6205031203 | 5.14 | 0.86 |  | 2.8 | 4.46 | 0 | 0 | 2 |  | 0 | 0 | 2 | 1 | 2 |
| 6205031204 | 2.79 | 0.79 |  | 1.61 | 0.89 | 0 | 0 | 1 |  | 0 | 0 | 1 | 1 | 1 |
| 6205031207 | 2.8 | 0.64 |  | 1.23 | 2.2 | 0 | 1 | 2 |  | 0 | 0 | 2 | 2 | 1 |
| 6205031208 | 2.98 | 0.62 |  | 1.48 | 1.28 | 0 | 1 | 2 |  | 0 | 0 | 2 | 2 | 2 |
| 6205031211 | 5.03 | 0.97 |  | 3.24 | 1.93 | 0 | 0 | 1 |  | 0 | 0 | 1 | 1 | 2 |
| 6205031212 | 4.32 | 1.44 |  | 2.15 | 1.18 | 0 | 0 | 1 |  | 0 | 0 | 1 | 1 | 1 |
| 6205031214 | 3.64 | 0.79 |  | 2.41 | 0.98 | 1 | 1 | 2 |  | 1 | 1 | 3 | 2 | 2 |
| 6205031215 | 3.56 | 0.84 |  | 2.1 | 1.54 | 0 | 1 | 1 |  | 0 | 1 | 2 | 1 | 1 |
| 6205031216 | 6.94 | 1.33 |  | 4.58 | 2.19 | 0 | 1 | 1 |  | 0 | 0 | 2 | 2 | 1 |
| 6205031217 | 3.54 | 0.74 |  | 1.66 | 2.95 | 0 | 0 | 1 |  | 0 | 0 | 2 | 1 | 2 |
| 6205031219 | 4.97 | 1.18 |  | 2.82 | 1.81 | 1 | 0 | 1 |  | 0 | 0 | 2 | 2 | 1 |
| 6205031220 | 5.15 | 1.44 |  | 2.88 | 1.56 | 0 | 0 | 2 |  | 0 | 0 | 1 | 1 | 1 |
| 6205031221 | 3.7 | 0.97 |  | 2.2 | 1.14 | 1 | 1 | 1 |  | 0 | 1 | 1 | 1 | 1 |
| 6205031222 | 3.85 | 0.99 |  | 2.26 | 1.56 | 1 | 1 | 0 |  | 0 | 1 | 2 | 1 | 2 |
| 6205031224 | 3.03 | 0.98 |  | 1.48 | 1.01 | 2 | 1 | 1 |  | 0 | 0 | 2 | 1 | 2 |
| 6205031225 | 2.15 | 0.67 |  | 0.95 | 1.05 | 0 | 1 | 2 |  | 0 | 0 | 2 | 1 | 1 |
| 6205031228 | 3.72 | 1.01 |  | 2.15 | 0.92 | 0 | 0 | 2 |  | 0 | 0 | 1 | 1 | 1 |
| 6205031230 | 3.14 | 0.93 |  | 1.83 | 0.69 | 0 | 1 | 1 |  | 0 | 0 | 1 | 1 | 1 |
| 6205031231 | 3.19 | 0.98 |  | 1.8 | 0.91 | 0 | 1 | 2 |  | 0 | 0 | 1 | 1 | 1 |
| 6205031232 | 3.58 | 1.1 |  | 2.11 | 0.96 | 0 | 0 | 1 |  | 0 | 0 | 1 | 1 | 1 |
| 6205031233 | 3.19 | 0.86 |  | 1.85 | 0.97 | 0 | 1 | 1 |  | 0 | 1 | 1 | 1 | 1 |
| 6205031238 | 4.94 | 0.78 |  | 3.18 | 2.83 | 0 | 1 | 2 |  | 0 | 1 | 2 | 1 | 1 |
| 6205031239 | 3.8 | 0.84 |  | 2.3 | 0.91 | 1 | 1 | 2 |  | 0 | 0 | 1 | 1 | 1 |
| 6205031243 | 4.63 | 1.48 |  | 2.33 | 1.12 | 0 | 0 | 1 |  | 0 | 0 | 1 | 1 | 1 |
| 6205031245 | 3.41 | 0.68 |  | 1.99 | 1.77 | 2 | 1 | 0 |  | 0 | 1 | 1 | 1 | 1 |
| 6205031303 | 4.72 | 0.91 |  | 3.38 | 0.88 | 2 | 1 | 1 |  | 0 | 0 | 1 | 1 | 1 |
| 6205031305 | 5.01 | 1.16 |  | 3.16 | 1.64 | 2 | 1 | 1 |  | 0 | 1 | 1 | 1 | 1 |
| 6205031312 | 3.32 | 1.25 |  | 1.49 | 0.75 | 2 | 1 | 0 |  | 1 | 1 | 1 | 1 | 1 |
| 6205031313 | 3.56 | 0.95 |  | 1.89 | 1.48 | 2 | 1 | 1 |  | 0 | 0 | 2 | 1 | 2 |
| 6205031314 | 3.14 | 0.75 |  | 1.47 | 2.14 | 0 | 0 | 1 |  | 0 | 0 | 2 | 2 | 2 |
| 6205031316 | 2.16 | 1 |  | 0.58 | 1.03 | 1 | 0 | 2 |  | 0 | 0 | 2 | 2 | 1 |
| 6205031317 | 4.47 | 0.96 |  | 2.43 | 1.59 | 2 | 0 | 1 |  | 0 | 0 | 1 | 1 | 2 |
| 6205031321 | 3.85 | 1.12 |  | 2.25 | 0.83 | 1 | 1 | 1 |  | 0 | 1 | 2 | 2 | 2 |
| 6205031322 | 3.49 | 0.95 |  | 1.98 | 1.68 | 2 | 1 | 1 |  | 0 | 1 | 2 | 2 | 2 |
| 6205031323 | 4.54 | 1.44 |  | 2.12 | 1.34 | 1 | 0 | 2 |  | 0 | 0 | 1 | 1 | 1 |
| 6205031324 | 5.65 | 1.06 |  | 2.81 | 3.36 | 1 | 0 | 2 |  | 0 | 0 | 2 | 1 | 2 |
| 6205031326 | 2.63 | 0.71 |  | 1.48 | 0.59 | 2 | 0 | 1 |  | 0 | 0 | 1 | 1 | 1 |
| 6205031332 | 3.17 | 1 |  | 1.51 | 1 | 0 | 0 | 1 |  | 0 | 0 | 1 | 1 | 1 |
| 6205031333 | 3 | 0.63 |  | 1.3 | 3.02 | 0 | 0 | 2 |  | 0 | 0 | 1 | 1 | 1 |
| 6205031335 | 4.66 | 1.48 |  | 2.71 | 0.81 | 1 | 1 | 1 |  | 0 | 0 | 1 | 1 | 1 |
| 6205031336 | 4.09 | 1.7 |  | 1.62 | 1.48 | 1 | 1 | 2 |  | 0 | 1 | 2 | 2 | 2 |
| 6205031340 | 3.01 | 1.4 |  | 1.19 | 0.46 | 0 | 0 | 1 |  | 0 | 0 | 1 | 1 | 1 |
| 6205031341 | 4.28 | 0.88 |  | 3 | 1.11 | 1 | 0 | 1 |  | 0 | 0 | 2 | 2 | 1 |
| 6205031342 | 4.77 | 1.02 |  | 3.38 | 1.79 | 1 | 1 | 2 |  | 1 | 0 | 2 | 2 | 2 |
| 6205031343 | 5.06 | 1.17 |  | 3.05 | 3.31 | 1 | 1 | 2 |  | 0 | 1 | 1 | 1 | 1 |
| 6205031346 | 3.11 | 1.05 |  | 1.62 | 1.04 | 1 | 0 | 2 |  | 0 | 0 | 1 | 1 | 1 |
| 6205031350 | 3 | 0.97 |  | 1.48 | 1.03 | 0 | 0 | 2 |  | 0 | 0 | 1 | 1 | 1 |
| 6205032102 | 2.97 | 0.88 |  | 1.75 | 0.8 | 0 | 1 | 1 |  | 0 | 1 | 1 | 1 | 1 |
| 6205032103 | 5.05 | 1.03 |  | 3.1 | 1.58 | 1 | 1 | 2 |  | 0 | 0 | 2 | 2 | 1 |
| 6205032105 | 2.27 | 0.8 |  | 1.11 | 0.4 | 1 | 0 | 0 |  | 0 | 0 | 1 | 1 | 1 |
| 6205032106 | 1.66 | 0.62 |  | 0.77 | 0.42 | 0 | 0 | 1 |  | 0 | 0 | 1 | 1 | 1 |
| 6205032107 | 2.97 | 0.97 |  | 1.51 | 0.94 | 0 | 0 | 0 |  | 0 | 0 | 2 | 1 | 1 |
| 6205032108 | 6.6 | 0.73 |  | 2.68 | 11.05 | 0 | 0 | 0 |  | 0 | 0 | 1 | 1 | 1 |
| 6205032111 | 2.08 | 0.52 |  | 1.03 | 0.95 | 0 | 1 | 1 |  | 0 | 1 | 1 | 1 | 1 |
| 6205032112 | 5.29 | 1.11 |  | 2.7 | 3.31 | 1 | 1 | 2 |  | 0 | 1 | 3 | 2 | 3 |
| 6205032116 | 3.09 | 0.85 |  | 1.56 | 0.77 | 1 | 1 | 2 |  | 0 | 1 | 1 | 1 | 1 |
| 6205032117 | 3.43 | 0.99 |  | 1.49 | 2.21 | 0 | 1 | 1 |  | 0 | 1 | 2 | 2 | 2 |
| 6205032118 | 3.66 | 1.35 |  | 1.72 | 0.91 | 0 | 1 | 1 |  | 0 | 1 | 1 | 1 | 1 |
| 6205032121 | 2.91 | 1.03 |  | 1.47 | 0.8 | 1 | 0 | 1 |  | 0 | 0 | 2 | 2 | 2 |
| 6205032122 | 3.31 | 0.8 |  | 1.69 | 1.3 | 1 | 0 | 0 |  | 0 | 0 | 1 | 1 | 1 |
| 6205032123 | 4.62 | 1.1 |  | 2.28 | 2.14 | 0 | 0 | 1 |  | 0 | 0 | 2 | 2 | 2 |
| 6205032124 | 3.36 | 0.69 |  | 1.83 | 2.11 | 1 | 0 | 0 |  | 0 | 0 | 1 | 1 | 1 |
| 6205032126 | 2.33 | 0.68 |  | 1.22 | 0.71 | 1 | 1 | 1 |  | 0 | 0 | 1 | 1 | 1 |
| 6205032127 | 4.57 | 0.92 |  | 2.58 | 2.82 | 0 | 1 | 1 |  | 0 | 0 | 1 | 1 | 1 |
| 6205032129 | 2.86 | 1.05 |  | 1.34 | 0.88 | 0 | 1 | 1 |  | 0 | 0 | 1 | 1 | 2 |
| 6205032130 | 3.61 | 0.93 |  | 1.8 | 1.6 | 1 | 0 | 0 |  | 0 | 0 | 1 | 1 | 1 |
| 6205032131 | 4.24 | 1.13 |  | 3.79 | 2.03 | 0 | 0 | 0 |  | 0 | 0 | 2 | 2 | 2 |
| 6205032132 | 3.2 | 1.22 |  | 1.31 | 0.86 | 0 | 0 | 1 |  | 0 | 0 | 1 | 1 | 1 |
| 6205032133 | 3.19 | 0.96 |  | 1.6 | 1.26 | 0 | 1 | 1 |  | 0 | 1 | 1 | 1 | 2 |
| 6205032135 | 3.27 | 1.21 |  | 1.37 | 0.75 | 2 | 1 | 0 |  | 0 | 1 | 1 | 1 | 1 |
| 6205032136 | 3.2 | 1.07 |  | 1.29 | 1.18 | 1 | 1 | 2 |  | 0 | 1 | 1 | 1 | 1 |
| 6205032137 | 3.67 | 0.6 |  | 1.84 | 2.21 | 1 | 0 | 0 |  | 0 | 0 | 1 | 1 | 1 |
| 6205032139 | 5.34 | 1.38 |  | 3.22 | 1.28 | 0 | 0 | 2 |  | 0 | 0 | 1 | 1 | 1 |
| 6205032141 | 4.32 | 1.52 |  | 2.47 | 0.61 | 0 | 1 | 2 |  | 1 | 1 | 1 | 1 | 1 |
| 6205032143 | 3.51 | 1.17 |  | 1.69 | 1.03 | 0 | 1 | 1 |  | 0 | 0 | 2 | 2 | 2 |
| 6205032144 | 2.18 | 0.59 |  | 1.09 | 1.14 | 1 | 0 | 0 |  | 0 | 0 | 2 | 2 | 3 |
| 6205032145 | 2.64 | 0.79 |  | 1.59 | 0.84 | 0 | 0 | 0 |  | 0 | 0 | 1 | 1 | 2 |
| 6205032147 | 4.08 | 1.26 |  | 1.91 | 0.88 | 0 | 0 | 1 |  | 0 | 0 | 1 | 1 | 1 |
| 6205032201 | 3.96 | 1.24 |  | 1.7 | 1.46 | 2 | 1 | 0 |  | 0 | 1 | 1 | 1 | 1 |
| 6205032202 | 4.81 | 1.14 |  | 2.55 | 2.62 | 0 | 1 | 1 |  | 0 | 1 | 3 | 2 | 3 |
| 6205032204 | 2.99 | 1.14 |  | 1.43 | 0.69 | 0 | 0 | 1 |  | 0 | 0 | 1 | 1 | 1 |
| 6205032205 | 2.68 | 0.89 |  | 1.25 | 0.98 | 0 | 0 | 1 |  | 0 | 0 | 2 | 2 | 3 |
| 6205032209 | 4.04 | 0.73 |  | 1.77 | 3.54 | 0 | 1 | 1 |  | 0 | 0 | 2 | 2 | 3 |
| 6205032212 | 3.38 | 1.34 |  | 1.65 | 0.76 | 0 | 0 | 1 |  | 0 | 0 | 1 | 1 | 1 |
| 6205032213 | 3.11 | 1.35 |  | 1.28 | 0.66 | 0 | 0 | 1 |  | 0 | 0 | 1 | 1 | 1 |
| 6205032214 | 4.39 | 1.43 |  | 2.38 | 0.97 | 0 | 0 | 1 |  | 0 | 0 | 1 | 1 | 1 |
| 6205032215 | 3.82 | 1.01 |  | 1.78 | 2.25 | 0 | 1 | 1 |  | 0 | 1 | 2 | 1 | 3 |
| 6205032216 | 4.57 | 1.23 |  | 2.55 | 1.57 | 0 | 1 | 1 |  | 0 | 1 | 2 | 2 | 3 |
| 6205032217 | 3.99 | 1.17 |  | 2.11 | 1.99 | 0 | 1 | 1 |  | 0 | 1 | 3 | 2 | 3 |
| 6205032218 | 4.56 | 1.27 |  | 2.09 | 7.22 | 1 | 1 | 2 |  | 0 | 1 | 2 | 2 | 3 |
| 6205032219 | 4.57 | 1.31 |  | 2.41 | 1.38 | 0 | 0 | 1 |  | 0 | 0 | 1 | 1 | 1 |
| 6205032220 | 3.35 | 1.04 |  | 1.57 | 0.88 | 1 | 0 | 0 |  | 0 | 0 | 1 | 1 | 1 |
| 6205032221 | 4.52 | 1.46 |  | 2.18 | 1.63 | 0 | 0 | 1 |  | 0 | 0 | 1 | 1 | 1 |
| 6205032224 | 3.02 | 0.9 |  | 1.78 | 0.82 | 0 | 1 | 1 |  | 0 | 0 | 2 | 2 | 3 |
| 6205032229 | 5.57 | 1.29 |  | 3.04 | 2.16 | 0 | 0 | 1 |  | 0 | 0 | 2 | 1 | 2 |
| 6205032230 | 4.25 | 1.05 |  | 2.13 | 2.09 | 0 | 0 | 0 |  | 0 | 0 | 1 | 1 | 2 |
| 6205032231 | 4.57 | 1.06 |  | 2.61 | 1.76 | 0 | 1 | 1 |  | 0 | 1 | 1 | 1 | 1 |
| 6205032232 | 5.29 | 1.49 |  | 2.81 | 1.3 | 1 | 1 | 2 |  | 0 | 1 | 1 | 1 | 2 |
| 6205032233 | 3.91 | 1.17 |  | 2.19 | 1.02 | 0 | 1 | 2 |  | 0 | 0 | 2 | 2 | 3 |
| 6205032234 | 2.64 | 0.71 |  | 1.05 | 2.39 | 0 | 1 | 1 |  | 0 | 1 | 3 | 2 | 3 |
| 6205032235 | 3.28 | 0.88 |  | 1.54 | 1.46 | 0 | 0 | 1 |  | 0 | 0 | 1 | 1 | 2 |
| 6205032236 | 4.45 | 1.17 |  | 2.55 | 1.26 | 1 | 0 | 1 |  | 0 | 0 | 1 | 1 | 1 |
| 6205032238 | 4.74 | 1.44 |  | 2.24 | 2.12 | 0 | 0 | 1 |  | 0 | 0 | 1 | 1 | 1 |
| 6205032239 | 4.05 | 1.07 |  | 2.14 | 1.42 | 0 | 1 | 1 |  | 0 | 1 | 2 | 1 | 2 |
| 6205032240 | 4.13 | 1.46 |  | 1.96 | 1.12 | 1 | 1 | 2 |  | 0 | 1 | 1 | 1 | 1 |
| 6205032241 | 6.25 | 1.27 |  | 4.03 | 2.34 | 0 | 1 | 1 |  | 0 | 0 | 1 | 1 | 1 |
| 6205032242 | 5.19 | 1.47 |  | 2.57 | 3.91 | 0 | 0 | 1 |  | 0 | 0 | 3 | 2 | 3 |
| 6205032246 | 5.21 | 1.71 |  | 2.55 | 1.25 | 1 | 0 | 0 |  | 0 | 0 | 1 | 1 | 1 |
| 6205032249 | 3.98 | 1.4 |  | 1.76 | 1.4 | 0 | 1 | 1 |  | 0 | 0 | 1 | 1 | 1 |
| 6205032301 | 4.04 | 1.11 |  | 2.45 | 0.85 | 0 | 0 | 0 |  | 0 | 0 | 1 | 1 | 1 |
| 6205032303 | 3 | 0.81 |  | 1.63 | 1.15 | 0 | 0 | 0 |  | 0 | 0 | 3 | 2 | 2 |
| 6205032304 | 2.84 | 1.22 |  | 1.15 | 0.87 | 1 | 0 | 0 |  | 0 | 0 | 1 | 1 | 2 |
| 6205032305 | 2.69 | 0.91 |  | 1.15 | 1.13 | 0 | 0 | 0 |  | 0 | 0 | 1 | 1 | 1 |
| 6205032312 | 3.71 | 1.23 |  | 1.78 | 1.59 | 0 | 0 | 1 |  | 0 | 0 | 2 | 2 | 2 |
| 6205032314 | 5.07 | 1.11 |  | 2.91 | 3.77 | 1 | 1 | 1 |  | 1 | 1 | 1 | 1 | 1 |
| 6205032317 | 3.82 | 1.36 |  | 1.93 | 1.42 | 0 | 0 | 1 |  | 0 | 0 | 1 | 1 | 1 |
| 6205032318 | 5.1 | 1.58 |  | 2.79 | 1.42 | 0 | 0 | 1 |  | 0 | 0 | 3 | 3 | 3 |
| 6205032320 | 2.88 | 0.84 |  | 1.3 | 1.2 | 0 | 0 | 1 |  | 0 | 0 | 1 | 1 | 1 |
| 6205032321 | 3.52 | 0.69 |  | 1.66 | 2.58 | 0 | 1 | 1 |  | 0 | 0 | 2 | 2 | 1 |
| 6205032322 | 4.19 | 1.29 |  | 2.17 | 0.84 | 1 | 1 | 2 |  | 0 | 0 | 1 | 1 | 1 |
| 6205032323 | 3.89 | 1.27 |  | 2.1 | 0.86 | 0 | 1 | 1 |  | 0 | 1 | 2 | 2 | 1 |
| 6205032325 | 2.85 | 0.79 |  | 1.6 | 1.07 | 0 | 0 | 0 |  | 0 | 0 | 1 | 1 | 1 |
| 6205032327 | 3.42 | 1.23 |  | 1.66 | 0.88 | 0 | 0 | 1 |  | 0 | 0 | 1 | 1 | 1 |
| 6205032328 | 3.85 | 1 |  | 2.29 | 1.02 | 1 | 0 | 0 |  | 0 | 0 | 1 | 1 | 1 |
| 6205032329 | 4.56 | 1.42 |  | 2.58 | 0.81 | 0 | 1 | 1 |  | 0 | 1 | 2 | 2 | 1 |
| 6205032331 | 3.15 | 0.97 |  | 1.64 | 0.88 | 0 | 1 | 1 |  | 0 | 0 | 1 | 1 | 1 |
| 6205032333 | 3.46 | 1.42 |  | 1.64 | 0.49 | 0 | 0 | 1 |  | 0 | 0 | 2 | 2 | 1 |
| 6205032335 | 4.83 | 1.46 |  | 2.64 | 1.55 | 1 | 0 | 1 |  | 0 | 0 | 1 | 1 | 1 |
| 6205032337 | 2.67 | 0.87 |  | 1.28 | 0.97 | 0 | 1 | 1 |  | 0 | 1 | 1 | 1 | 1 |
| 6205032340 | 2.51 | 0.81 |  | 1.13 | 1.42 | 0 | 1 | 1 |  | 0 | 1 | 1 | 1 | 1 |
| 6205032342 | 4.11 | 0.94 |  | 2.73 | 0.81 | 1 | 0 | 0 |  | 0 | 0 | 1 | 1 | 1 |
| 6205032344 | 3.86 | 1.07 |  | 2.22 | 1.72 | 0 | 0 | 1 |  | 0 | 0 | 1 | 1 | 2 |
| 6205032345 | 4 | 1.1 |  | 2.34 | 1.2 | 0 | 1 | 0 |  | 0 | 1 | 1 | 1 | 2 |
| 6205032346 | 3.58 | 0.85 |  | 1.84 | 1.67 | 1 | 0 | 0 |  | 0 | 0 | 1 | 1 | 1 |
| 6205032350 | 3.76 | 1.14 |  | 1.91 | 1.03 | 0 | 0 | 1 |  | 0 | 0 | 1 | 1 | 1 |
| 6205033105 | 3.2 | 1.07 |  | 1.55 | 1.27 | 0 | 1 | 1 |  | 1 | 1 | 1 | 1 | 1 |
| 6205033110 | 3.87 | 0.92 |  | 2.5 | 1.09 | 1 | 0 | 0 |  | 0 | 0 | 1 | 1 | 1 |
| 6205033112 | 2.72 | 0.67 |  | 1.6 | 1.18 | 1 | 1 | 1 |  | 1 | 1 | 1 | 1 | 1 |
| 6205033113 | 2.76 | 1.23 |  | 1.19 | 0.4 | 2 | 0 | 0 |  | 0 | 0 | 1 | 1 | 1 |
| 6205033115 | 3.65 | 0.94 |  | 2.22 | 0.76 | 1 | 0 | 1 |  | 0 | 0 | 2 | 1 | 2 |
| 6205033116 | 3.37 | 1.29 |  | 1.56 | 0.94 | 0 | 0 | 0 |  | 0 | 0 | 1 | 1 | 1 |
| 6205033117 | 2.65 | 0.89 |  | 1.24 | 1.21 | 0 | 0 | 0 |  | 0 | 0 | 1 | 1 | 1 |
| 6205033119 | 6.15 | 0.68 |  | 2.63 | 9.18 | 0 | 0 | 2 |  | 0 | 0 | 1 | 1 | 1 |
| 6205033120 | 3.65 | 1.29 |  | 1.78 | 1.07 | 0 | 0 | 1 |  | 0 | 0 | 1 | 1 | 1 |
| 6205033121 | 2.29 | 1.13 |  | 0.91 | 0.65 | 0 | 0 | 1 |  | 0 | 0 | 1 | 1 | 1 |
| 6205033123 | 3.35 | 0.91 |  | 1.92 | 1.08 | 1 | 0 | 1 |  | 0 | 0 | 2 | 1 | 2 |
| 6205033124 | 3.47 | 1.06 |  | 1.84 | 1.37 | 0 | 0 | 1 |  | 0 | 0 | 2 | 1 | 1 |
| 6205033126 | 4.82 | 0.9 |  | 2 | 9.41 | 1 | 0 | 1 |  | 0 | 0 | 1 | 1 | 1 |
| 6205033128 | 3.89 | 0.81 |  | 2.35 | 1.58 | 1 | 0 | 0 |  | 0 | 0 | 3 | 3 | 3 |
| 6205033130 | 2.46 | 0.84 |  | 1.25 | 0.53 | 1 | 1 | 1 |  | 0 | 1 | 1 | 1 | 1 |
| 6205033132 | 3.53 | 0.8 |  | 1.67 | 2.14 | 1 | 0 | 0 |  | 0 | 0 | 2 | 2 | 2 |
| 6205033137 | 4.06 | 1.25 |  | 1.98 | 1.15 | 0 | 1 | 1 |  | 0 | 1 | 1 | 1 | 1 |
| 6205033138 | 3.04 | 1 |  | 1.59 | 0.74 | 1 | 0 | 0 |  | 0 | 0 | 1 | 1 | 1 |
| 6205033142 | 3.9 | 1 |  | 2.14 | 1.39 | 2 | 1 | 1 |  | 1 | 0 | 1 | 1 | 1 |
| 6205033144 | 2.7 | 0.7 |  | 1.43 | 1.44 | 1 | 1 | 2 |  | 1 | 1 | 2 | 1 | 1 |
| 6205033145 | 3.12 | 1.01 |  | 1.73 | 0.91 | 1 | 0 | 1 |  | 0 | 0 | 3 | 2 | 2 |
| 6205033146 | 3.35 | 0.92 |  | 1.96 | 1.55 | 1 | 1 | 1 |  | 0 | 1 | 2 | 2 | 2 |
| 6205033147 | 2.7 | 0.7 |  | 1.59 | 0.81 | 1 | 1 | 1 |  | 0 | 1 | 2 | 1 | 2 |
| 6205033150 | 3.63 | 0.84 |  | 2.03 | 1.72 | 0 | 0 | 1 |  | 0 | 0 | 1 | 1 | 2 |
| 6205033203 | 2.05 | 0.67 |  | 0.92 | 1.27 | 1 | 1 | 1 |  | 0 | 1 | 1 | 1 | 1 |
| 6205033204 | 3.34 | 0.85 |  | 1.81 | 1.58 | 0 | 1 | 0 |  | 0 | 1 | 1 | 1 | 1 |
| 6205033207 | 3.45 | 0.97 |  | 1.88 | 1.01 | 1 | 1 | 1 |  | 0 | 1 | 1 | 1 | 1 |
| 6205033209 | 5.06 | 1.2 |  | 2.88 | 1.74 | 0 | 1 | 1 |  | 0 | 1 | 1 | 1 | 1 |
| 6205033210 | 2.77 | 0.89 |  | 1.6 | 0.67 | 0 | 0 | 0 |  | 0 | 0 | 1 | 1 | 1 |
| 6205033211 | 4.34 | 0.99 |  | 2.69 | 1.51 | 1 | 1 | 0 |  | 0 | 1 | 3 | 2 | 3 |
| 6205033212 | 3.11 | 1.01 |  | 1.58 | 0.99 | 1 | 1 | 1 |  | 0 | 0 | 1 | 1 | 1 |
| 6205033213 | 3.8 | 1.15 |  | 2.29 | 0.89 | 1 | 0 | 0 |  | 0 | 0 | 2 | 1 | 2 |
| 6205033215 | 4.45 | 0.8 |  | 1.92 | 8.85 | 0 | 0 | 0 |  | 0 | 0 | 2 | 2 | 2 |
| 6205033219 | 4.04 | 1.14 |  | 2.15 | 1.46 | 2 | 1 | 1 |  | 0 | 1 | 1 | 1 | 1 |
| 6205033220 | 2.83 | 1.03 |  | 1.38 | 1.19 | 0 | 0 | 0 |  | 0 | 0 | 2 | 2 | 3 |
| 6205033222 | 4.06 | 0.92 |  | 2.21 | 2.31 | 1 | 0 | 0 |  | 0 | 0 | 3 | 3 | 3 |
| 6205033226 | 3.71 | 1.15 |  | 2.24 | 1.02 | 0 | 1 | 0 |  | 0 | 1 | 2 | 1 | 2 |
| 6205033227 | 3.76 | 1.39 |  | 1.86 | 0.9 | 1 | 1 | 1 |  | 1 | 1 | 1 | 1 | 1 |
| 6205033228 | 3.26 | 1.5 |  | 1.17 | 1.03 | 1 | 1 | 1 |  | 0 | 1 | 1 | 1 | 1 |
| 6205033234 | 4.21 | 1.22 |  | 2.45 | 1.03 | 0 | 1 | 1 |  | 0 | 1 | 1 | 1 | 1 |
| 6205033235 | 3.64 | 1.21 |  | 2.07 | 0.96 | 0 | 1 | 0 |  | 0 | 1 | 2 | 1 | 1 |
| 6205033236 | 3.9 | 1.08 |  | 1.95 | 1.71 | 1 | 1 | 1 |  | 0 | 1 | 2 | 2 | 2 |
| 6205033244 | 2.84 | 0.87 |  | 1.79 | 0.76 | 0 | 1 | 0 |  | 0 | 0 | 1 | 1 | 2 |
| 6205033246 | 3.74 | 1.27 |  | 1.91 | 0.76 | 2 | 0 | 0 |  | 0 | 0 | 1 | 1 | 1 |
| 6205033247 | 4.64 | 1.26 |  | 2.66 | 1.52 | 1 | 0 | 0 |  | 0 | 0 | 1 | 1 | 1 |
| 6205033248 | 2.92 | 0.82 |  | 1.8 | 0.59 | 0 | 0 | 0 |  | 0 | 0 | 1 | 1 | 2 |
| 6205033301 | 3.31 | 1.16 |  | 1.71 | 0.69 | 0 | 0 | 1 |  | 0 | 0 | 1 | 1 | 1 |
| 6205033303 | 3.4 | 0.81 |  | 2.14 | 0.96 | 1 | 0 | 1 |  | 0 | 0 | 2 | 1 | 2 |
| 6205033304 | 3.16 | 0.76 |  | 2.09 | 0.93 | 1 | 0 | 0 |  | 0 | 0 | 1 | 1 | 1 |
| 6205033305 | 3.42 | 0.58 |  | 2.21 | 1.29 | 0 | 1 | 1 |  | 0 | 1 | 2 | 2 | 3 |
| 6205033306 | 4.67 | 0.99 |  | 3.42 | 1.57 | 0 | 1 | 1 |  | 0 | 0 | 1 | 1 | 1 |
| 6205033307 | 3.87 | 1.04 |  | 2.43 | 0.68 | 0 | 0 | 1 |  | 0 | 0 | 1 | 1 | 1 |
| 6205033311 | 2.61 | 0.92 |  | 1.32 | 0.6 | 0 | 0 | 1 |  | 0 | 0 | 1 | 1 | 1 |
| 6205033312 | 3.66 | 1.1 |  | 2 | 1.04 | 1 | 0 | 0 |  | 0 | 0 | 1 | 1 | 1 |
| 6205033313 | 3.31 | 0.82 |  | 2.01 | 1.01 | 0 | 0 | 1 |  | 0 | 0 | 2 | 1 | 3 |
| 6205033314 | 3.28 | 0.78 |  | 2.17 | 0.95 | 0 | 0 | 1 |  | 0 | 0 | 1 | 1 | 1 |
| 6205033315 | 3.53 | 0.95 |  | 1.95 | 1.5 | 1 | 1 | 1 |  | 0 | 1 | 1 | 1 | 1 |
| 6205033316 | 3.04 | 0.71 |  | 1.9 | 0.94 | 2 | 1 | 0 |  | 0 | 0 | 1 | 1 | 1 |
| 6205033317 | 3.74 | 1.06 |  | 2.17 | 1.44 | 0 | 1 | 1 |  | 0 | 0 | 1 | 1 | 1 |
| 6205033318 | 3.67 | 0.77 |  | 1.59 | 3.19 | 1 | 1 | 1 |  | 0 | 1 | 3 | 2 | 3 |
| 6205033319 | 3.53 | 0.85 |  | 2.3 | 0.97 | 0 | 0 | 1 |  | 0 | 0 | 1 | 1 | 1 |
| 6205033321 | 4.69 | 1.41 |  | 2.64 | 1.9 | 0 | 0 | 1 |  | 0 | 0 | 2 | 1 | 1 |
| 6205033322 | 4.46 | 1.33 |  | 2.5 | 1.73 | 0 | 0 | 1 |  | 0 | 0 | 1 | 1 | 2 |
| 6205033324 | 3.66 | 0.81 |  | 1.54 | 2.58 | 1 | 1 | 2 |  | 0 | 1 | 1 | 1 | 1 |
| 6205033326 | 4.05 | 1.17 |  | 2.21 | 1.69 | 0 | 0 | 1 |  | 0 | 0 | 1 | 1 | 1 |
| 6205033327 | 3.9 | 0.83 |  | 2.45 | 1.25 | 0 | 0 | 1 |  | 0 | 0 | 2 | 2 | 3 |
| 6205033328 | 2.61 | 0.86 |  | 1.35 | 0.73 | 0 | 1 | 1 |  | 0 | 0 | 1 | 1 | 1 |
| 6205033329 | 2.67 | 0.95 |  | 2.02 | 0.81 | 0 | 0 | 1 |  | 0 | 0 | 1 | 1 | 1 |
| 6205033330 | 2.23 | 0.58 |  | 1.32 | 0.56 | 1 | 1 | 0 |  | 0 | 0 | 1 | 1 | 1 |
| 6205033331 | 4.03 | 0.97 |  | 2.69 | 1.15 | 1 | 1 | 1 |  | 0 | 1 | 1 | 1 | 1 |
| 6205033332 | 2.33 | 0.72 |  | 1.23 | 0.87 | 0 | 0 | 1 |  | 0 | 0 | 1 | 1 | 1 |
| 6205033335 | 2.93 | 1.05 |  | 1.35 | 0.92 | 0 | 0 | 1 |  | 0 | 0 | 1 | 1 | 1 |
| 6205033340 | 3.22 | 1.09 |  | 1.57 | 1.61 | 2 | 1 | 0 |  | 0 | 1 | 1 | 1 | 1 |
| 6205033341 | 4.08 | 1.21 |  | 2.26 | 1.66 | 0 | 1 | 1 |  | 0 | 0 | 1 | 1 | 1 |
| 6205033342 | 3.02 | 1.05 |  | 1.46 | 1.19 | 1 | 1 | 1 |  | 1 | 1 | 2 | 2 | 2 |
| 6205033343 | 3.15 | 1.09 |  | 1.33 | 1.71 | 1 | 0 | 0 |  | 0 | 0 | 1 | 1 | 1 |
| 6205033344 | 8.08 | 0.87 |  | 4.06 | 3.49 | 2 | 0 | 1 |  | 0 | 0 | 1 | 1 | 2 |
| 6205033345 | 4.44 | 1.3 |  | 2.46 | 1.76 | 0 | 0 | 1 |  | 0 | 0 | 1 | 1 | 1 |
| 6205033346 | 2.96 | 0.72 |  | 1.12 | 1.46 | 1 | 1 | 1 |  | 1 | 1 | 1 | 1 | 1 |
| 6205033347 | 3.78 | 0.92 |  | 2.28 | 1.25 | 2 | 1 | 1 |  | 1 | 1 | 1 | 1 | 1 |
| 6205033348 | 4.95 | 0.78 |  | 2.72 | 3.56 | 0 | 1 | 2 |  | 0 | 0 | 2 | 2 | 2 |
| 6205034102 | 3.99 | 1.23 |  | 2.14 | 1.13 | 0 | 0 | 1 |  | 0 | 0 | 1 | 1 | 1 |
| 6205034104 | 2.67 | 1 |  | 1.22 | 1.11 | 0 | 0 | 0 |  | 0 | 0 | 1 | 1 | 1 |
| 6205034106 | 4.44 | 1.32 |  | 2.52 | 1.66 | 0 | 1 | 1 |  | 0 | 0 | 2 | 1 | 2 |
| 6205034107 | 4.65 | 0.94 |  | 3.07 | 1.21 | 2 | 1 | 1 |  | 0 | 0 | 2 | 1 | 1 |
| 6205034108 | 3.46 | 0.93 |  | 2.12 | 0.78 | 1 | 1 | 1 |  | 0 | 1 | 2 | 2 | 1 |
| 6205034112 | 4.02 | 1.07 |  | 2.42 | 0.95 | 0 | 0 | 1 |  | 0 | 0 | 2 | 2 | 3 |
| 6205034115 | 2.63 | 0.61 |  | 1.39 | 1.43 | 1 | 1 | 1 |  | 0 | 0 | 2 | 1 | 2 |
| 6205034118 | 4.45 | 1 |  | 2.95 | 1.49 | 0 | 0 | 0 |  | 0 | 0 | 2 | 2 | 3 |
| 6205034120 | 3.25 | 1.2 |  | 1.38 | 1.07 | 2 | 0 | 0 |  | 0 | 0 | 1 | 1 | 1 |
| 6205034122 | 4.23 | 1.96 |  | 1.75 | 0.69 | 0 | 1 | 1 |  | 0 | 0 | 1 | 1 | 1 |
| 6205034125 | 2.61 | 1.07 |  | 1.01 | 1.4 | 1 | 0 | 1 |  | 0 | 0 | 2 | 2 | 2 |
| 6205034127 | 4.23 | 0.94 |  | 2.32 | 1.9 | 1 | 0 | 1 |  | 0 | 0 | 2 | 2 | 3 |
| 6205034128 | 6.12 | 1.55 |  | 3.85 | 2.02 | 0 | 0 | 1 |  | 0 | 0 | 2 | 2 | 1 |
| 6205034129 | 5.4 | 0.74 |  | 2.53 | 4.47 | 1 | 1 | 1 |  | 0 | 1 | 2 | 2 | 2 |
| 6205034131 | 4.43 | 0.85 |  | 2.54 | 2.74 | 0 | 1 | 1 |  | 0 | 0 | 1 | 1 | 1 |
| 6205034133 | 3.45 | 0.7 |  | 1.45 | 3.13 | 1 | 0 | 1 |  | 0 | 0 | 1 | 1 | 1 |
| 6205034137 | 3.91 | 0.81 |  | 1.78 | 3.17 | 0 | 1 | 2 |  | 0 | 0 | 2 | 2 | 1 |
| 6205034138 | 5.13 | 1.59 |  | 2.82 | 0.87 | 2 | 1 | 1 |  | 0 | 0 | 1 | 1 | 1 |
| 6205034144 | 1.96 | 0.63 |  | 1.02 | 0.54 | 0 | 0 | 0 |  | 0 | 0 | 2 | 2 | 3 |
| 6205034145 | 3.58 | 0.78 |  | 2.17 | 1.62 | 0 | 1 | 0 |  | 0 | 1 | 2 | 2 | 2 |
| 6205034146 | 4.14 | 1.15 |  | 2.43 | 0.97 | 0 | 1 | 1 |  | 0 | 1 | 2 | 2 | 2 |
| 6205034148 | 2.82 | 1.02 |  | 1.27 | 0.91 | 1 | 1 | 0 |  | 0 | 1 | 1 | 1 | 1 |
| 6205034149 | 2.96 | 0.56 |  | 1.64 | 1.55 | 0 | 0 | 1 |  | 0 | 0 | 2 | 1 | 2 |
| 6205034150 | 3.35 | 0.86 |  | 1.87 | 1.62 | 0 | 1 | 1 |  | 0 | 0 | 2 | 1 | 2 |
| 6205034202 | 4.71 | 1.17 |  | 3.06 | 0.94 | 1 | 1 | 0 |  | 0 | 1 | 1 | 1 | 1 |
| 6205034203 | 4.01 | 1.47 |  | 2.02 | 1.04 | 1 | 0 | 1 |  | 0 | 0 | 2 | 2 | 2 |
| 6205034204 | 4.08 | 1.11 |  | 2.4 | 1.47 | 0 | 1 | 1 |  | 0 | 1 | 1 | 1 | 1 |
| 6205034206 | 2.65 | 0.93 |  | 1.43 | 0.54 | 0 | 1 | 2 |  | 0 | 0 | 1 | 1 | 2 |
| 6205034208 | 3.27 | 0.88 |  | 2 | 0.79 | 0 | 0 | 1 |  | 0 | 0 | 1 | 1 | 1 |
| 6205034209 | 3.82 | 1.08 |  | 2.24 | 0.82 | 0 | 0 | 2 |  | 0 | 0 | 1 | 1 | 1 |
| 6205034210 | 4.5 | 1.27 |  | 2.3 | 1.76 | 1 | 0 | 0 |  | 0 | 0 | 3 | 2 | 3 |
| 6205034212 | 4.42 | 1.11 |  | 2.37 | 1.91 | 0 | 0 | 2 |  | 0 | 0 | 2 | 1 | 1 |
| 6205034213 | 3.78 | 1.45 |  | 1.89 | 0.94 | 0 | 0 | 1 |  | 0 | 0 | 1 | 1 | 1 |
| 6205034214 | 3.23 | 0.98 |  | 1.88 | 0.62 | 0 | 0 | 1 |  | 0 | 0 | 1 | 1 | 1 |
| 6205034217 | 2.48 | 0.81 |  | 0.98 | 1.82 | 0 | 0 | 0 |  | 0 | 0 | 1 | 1 | 1 |
| 6205034218 | 5.59 | 1.37 |  | 3.58 | 1.24 | 1 | 1 | 2 |  | 0 | 1 | 1 | 1 | 1 |
| 6205034220 | 2.22 | 0.75 |  | 1.12 | 0.8 | 0 | 0 | 1 |  | 0 | 0 | 2 | 1 | 2 |
| 6205034221 | 2.24 | 0.72 |  | 0.83 | 1.62 | 0 | 0 | 1 |  | 0 | 0 | 1 | 1 | 1 |
| 6205034223 | 2.89 | 0.95 |  | 1.58 | 0.58 | 0 | 0 | 0 |  | 0 | 0 | 1 | 1 | 1 |
| 6205034224 | 7 | 0.65 |  | 3.1 | 22.03 | 0 | 0 | 1 |  | 0 | 0 | 2 | 2 | 2 |
| 6205034225 | 5.04 | 1.39 |  | 3.02 | 1.11 | 1 | 0 | 1 |  | 0 | 0 | 1 | 1 | 1 |
| 6205034226 | 3.36 | 1.46 |  | 1.49 | 0.64 | 0 | 0 | 1 |  | 0 | 0 | 1 | 1 | 1 |
| 6205034229 | 3.17 | 0.77 |  | 1.11 | 2.69 | 1 | 0 | 2 |  | 0 | 0 | 3 | 2 | 2 |
| 6205034231 | 4.39 | 0.97 |  | 2.36 | 2.03 | 1 | 0 | 0 |  | 0 | 0 | 1 | 1 | 1 |
| 6205034232 | 3.81 | 1.68 |  | 1.57 | 0.66 | 0 | 0 | 2 |  | 0 | 0 | 2 | 2 | 1 |
| 6205034233 | 5.09 | 1.62 |  | 2.55 | 1.28 | 1 | 0 | 0 |  | 0 | 0 | 1 | 1 | 2 |
| 6205034234 | 4.81 | 1.28 |  | 2.58 | 1.42 | 1 | 0 | 0 |  | 0 | 0 | 1 | 1 | 1 |
| 6205034235 | 3.64 | 1.09 |  | 2.07 | 0.84 | 0 | 1 | 1 |  | 0 | 1 | 1 | 1 | 1 |
| 6205034237 | 3 | 1.01 |  | 1.44 | 1.28 | 0 | 0 | 2 |  | 0 | 0 | 1 | 1 | 1 |
| 6205034239 | 3.08 | 1.16 |  | 1.27 | 1.08 | 0 | 0 | 2 |  | 0 | 0 | 1 | 1 | 1 |
| 6205034241 | 3.56 | 1.34 |  | 1.66 | 1.49 | 0 | 0 | 0 |  | 0 | 0 | 1 | 1 | 1 |
| 6205034244 | 2.75 | 0.85 |  | 1.63 | 0.52 | 0 | 1 | 2 |  | 0 | 1 | 1 | 1 | 1 |
| 6205034245 | 1.93 | 0.9 |  | 0.69 | 0.57 | 0 | 1 | 2 |  | 0 | 1 | 1 | 1 | 1 |
| 6205034246 | 4.31 | 1.3 |  | 2.52 | 0.92 | 1 | 1 | 2 |  | 1 | 1 | 2 | 1 | 1 |
| 6205034247 | 2.34 | 0.84 |  | 1.03 | 0.62 | 2 | 0 | 0 |  | 0 | 0 | 1 | 1 | 1 |
| 6205034248 | 2.91 | 0.63 |  | 1.33 | 2.48 | 1 | 0 | 1 |  | 0 | 0 | 1 | 1 | 1 |
| 6205034301 | 4.13 | 1.3 |  | 2.37 | 1.06 | 1 | 1 | 1 |  | 1 | 1 | 1 | 1 | 1 |
| 6205034303 | 4.06 | 1.13 |  | 2.24 | 1.2 | 2 | 1 | 0 |  | 0 | 1 | 1 | 1 | 1 |
| 6205034308 | 4 | 1.11 |  | 1.95 | 2.16 | 1 | 0 | 1 |  | 0 | 0 | 2 | 2 | 2 |
| 6205034309 | 4.54 | 0.97 |  | 2.12 | 2.54 | 0 | 0 | 1 |  | 0 | 0 | 1 | 1 | 2 |
| 6205034311 | 2.87 | 0.7 |  | 1.83 | 0.86 | 0 | 1 | 1 |  | 0 | 1 | 2 | 2 | 1 |
| 6205034314 | 3.19 | 1.15 |  | 1.48 | 0.83 | 0 | 0 | 1 |  | 0 | 0 | 1 | 1 | 2 |
| 6205034318 | 4 | 1.44 |  | 2.05 | 1.1 | 0 | 1 | 1 |  | 0 | 1 | 1 | 1 | 1 |
| 6205034319 | 3.62 | 1.58 |  | 1.53 | 1.3 | 0 | 0 | 1 |  | 0 | 0 | 1 | 1 | 1 |
| 6205034320 | 4.86 | 1.06 |  | 3.05 | 1.44 | 0 | 1 | 2 |  | 0 | 0 | 1 | 1 | 1 |
| 6205034322 | 3.51 | 0.84 |  | 1.6 | 2.08 | 1 | 0 | 1 |  | 0 | 0 | 2 | 1 | 2 |
| 6205034326 | 2.99 | 0.98 |  | 1.43 | 0.74 | 1 | 1 | 2 |  | 0 | 1 | 1 | 1 | 1 |
| 6205034331 | 3.53 | 1.01 |  | 1.74 | 1.38 | 0 | 0 | 2 |  | 0 | 0 | 1 | 1 | 2 |
| 6205034332 | 3 | 0.77 |  | 1.37 | 2.19 | 0 | 0 | 1 |  | 0 | 0 | 1 | 1 | 2 |
| 6205034335 | 2.98 | 1.09 |  | 1.45 | 1.01 | 0 | 0 | 2 |  | 0 | 0 | 1 | 1 | 1 |
| 6205034337 | 3.48 | 0.87 |  | 2.01 | 1.47 | 0 | 0 | 1 |  | 0 | 0 | 2 | 1 | 1 |
| 6205034339 | 4.14 | 1.25 |  | 2.32 | 0.87 | 0 | 0 | 2 |  | 0 | 0 | 1 | 1 | 2 |
| 6205034342 | 3.61 | 0.75 |  | 1.96 | 1.93 | 2 | 1 | 1 |  | 0 | 1 | 2 | 1 | 3 |
| 6205034344 | 2 | 0.74 |  | 0.88 | 0.63 | 0 | 0 | 1 |  | 0 | 0 | 1 | 1 | 1 |
| 6205034347 | 4.86 | 1.14 |  | 2.54 | 1.81 | 2 | 0 | 1 |  | 0 | 0 | 2 | 2 | 2 |
| 6205034348 | 4.12 | 1.07 |  | 2.6 | 0.89 | 1 | 0 | 0 |  | 0 | 0 | 1 | 1 | 2 |
| 6205034349 | 4.93 | 1 |  | 3.19 | 2.09 | 0 | 1 | 1 |  | 0 | 0 | 2 | 2 | 2 |
| 6221031101 | 3.12 | 0.85 |  | 1.54 | 1.42 | 1 | 0 | 1 |  | 0 | 0 | 1 | 1 | 1 |
| 6221031103 | 3.11 | 1.1 |  | 1.59 | 0.57 | 0 | 0 | 2 |  | 0 | 0 | 1 | 1 | 1 |
| 6221031104 | 2.63 | 0.85 |  | 1.28 | 0.94 | 0 | 1 | 1 |  | 0 | 1 | 1 | 1 | 1 |
| 6221031105 | 3.98 | 1.12 |  | 2.44 | 1.17 | 0 | 1 | 1 |  | 0 | 1 | 1 | 1 | 1 |
| 6221031107 | 2.34 | 0.71 |  | 1.21 | 0.52 | 2 | 1 | 1 |  | 0 | 0 | 1 | 1 | 2 |
| 6221031109 | 3.41 | 1.32 |  | 1.42 | 1.27 | 1 | 0 | 1 |  | 0 | 0 | 2 | 1 | 1 |
| 6221031111 | 3.6 | 1.11 |  | 1.84 | 1.29 | 0 | 0 | 1 |  | 0 | 0 | 1 | 1 | 1 |
| 6221031113 | 4.55 | 1.43 |  | 2.46 | 0.83 | 0 | 0 | 1 |  | 0 | 0 | 1 | 1 | 1 |
| 6221031114 | 3.05 | 1.21 |  | 1.35 | 0.46 | 1 | 1 | 1 |  | 1 | 1 | 1 | 1 | 1 |
| 6221031115 | 2.86 | 1.05 |  | 1.46 | 0.51 | 2 | 1 | 1 |  | 0 | 1 | 1 | 1 | 1 |
| 6221031126 | 2.63 | 0.8 |  | 1.39 | 0.99 | 1 | 0 | 1 |  | 0 | 0 | 2 | 1 | 1 |
| 6221031128 | 4.06 | 1.07 |  | 2.19 | 1.38 | 0 | 0 | 1 |  | 0 | 0 | 2 | 1 | 1 |
| 6221031132 | 3.34 | 1.07 |  | 1.73 | 1.17 | 0 | 0 | 1 |  | 0 | 0 | 2 | 2 | 2 |
| 6221031134 | 2.72 | 1.19 |  | 1.19 | 0.6 | 0 | 0 | 1 |  | 0 | 0 | 1 | 1 | 1 |
| 6221031135 | 3.33 | 0.94 |  | 1.5 | 1.38 | 0 | 0 | 1 |  | 0 | 0 | 1 | 1 | 1 |
| 6221031141 | 3.64 | 1.15 |  | 1.96 | 0.88 | 0 | 0 | 1 |  | 0 | 0 | 2 | 2 | 2 |
| 6221031142 | 3.31 | 0.61 |  | 2.02 | 1.86 | 0 | 1 | 1 |  | 0 | 1 | 3 | 3 | 3 |
| 6221031144 | 3.95 | 0.85 |  | 2.13 | 1.83 | 1 | 1 | 0 |  | 0 | 1 | 2 | 2 | 1 |
| 6221031145 | 2.26 | 0.7 |  | 0.77 | 1.22 | 2 | 1 | 1 |  | 0 | 1 | 2 | 1 | 1 |
| 6221031201 | 3.77 | 1.29 |  | 1.89 | 1.3 | 0 | 0 | 2 |  | 0 | 0 | 1 | 1 | 1 |
| 6221031202 | 3.9 | 1.16 |  | 2.01 | 1.27 | 2 | 1 | 0 |  | 0 | 0 | 1 | 1 | 1 |
| 6221031204 | 3.56 | 0.82 |  | 1.46 | 3.1 | 0 | 1 | 1 |  | 0 | 0 | 2 | 1 | 1 |
| 6221031206 | 3.2 | 1.08 |  | 1.51 | 0.94 | 0 | 0 | 1 |  | 0 | 0 | 1 | 1 | 1 |
| 6221031208 | 3.44 | 1.02 |  | 1.93 | 0.85 | 0 | 0 | 1 |  | 0 | 0 | 2 | 2 | 2 |
| 6221031209 | 3.44 | 1.23 |  | 1.63 | 1 | 0 | 0 | 2 |  | 0 | 0 | 2 | 1 | 1 |
| 6221031210 | 3.75 | 1.15 |  | 2.12 | 1.2 | 1 | 1 | 2 |  | 0 | 0 | 2 | 2 | 2 |
| 6221031211 | 3.17 | 0.56 |  | 1.33 | 5.73 | 0 | 1 | 1 |  | 1 | 1 | 3 | 2 | 2 |
| 6221031213 | 3 | 0.86 |  | 1.82 | 0.39 | 0 | 1 | 2 |  | 0 | 0 | 1 | 1 | 1 |
| 6221031215 | 2.76 | 0.5 |  | 1.68 | 1 | 0 | 0 | 1 |  | 0 | 0 | 3 | 2 | 3 |
| 6221031218 | 4.01 | 0.91 |  | 2.6 | 1.07 | 0 | 1 | 2 |  | 0 | 1 | 3 | 2 | 2 |
| 6221031221 | 3.55 | 0.77 |  | 1.99 | 1.8 | 1 | 1 | 1 |  | 0 | 0 | 3 | 3 | 3 |
| 6221031222 | 3.86 | 0.69 |  | 1.97 | 2.78 | 1 | 0 | 0 |  | 0 | 0 | 3 | 3 | 3 |
| 6221031223 | 2.98 | 0.91 |  | 1.54 | 0.93 | 1 | 0 | 0 |  | 0 | 0 | 2 | 2 | 1 |
| 6221031225 | 4.87 | 1.03 |  | 1.89 | 8.23 | 0 | 1 | 2 |  | 0 | 1 | 2 | 2 | 2 |
| 6221031226 | 3.98 | 0.85 |  | 1.82 | 2.89 | 1 | 1 | 1 |  | 0 | 1 | 3 | 2 | 2 |
| 6221031227 | 3.34 | 1.1 |  | 1.83 | 0.87 | 0 | 1 | 1 |  | 0 | 0 | 1 | 1 | 1 |
| 6221031228 | 4.61 | 1.18 |  | 3.04 | 0.97 | 0 | 1 | 1 |  | 0 | 0 | 1 | 1 | 1 |
| 6221031232 | 3.22 | 1 |  | 1.7 | 0.79 | 0 | 0 | 1 |  | 0 | 0 | 2 | 1 | 1 |
| 6221031233 | 2.02 | 0.79 |  | 0.77 | 0.79 | 0 | 0 | 1 |  | 0 | 0 | 1 | 1 | 1 |
| 6221031235 | 3.59 | 0.78 |  | 2.3 | 1.65 | 0 | 1 | 2 |  | 0 | 1 | 2 | 2 | 3 |
| 6221031238 | 3.89 | 0.97 |  | 2.17 | 1.08 | 0 | 0 | 2 |  | 0 | 0 | 1 | 1 | 1 |
| 6221031239 | 4.17 | 1.16 |  | 2.52 | 1.28 | 0 | 0 | 2 |  | 0 | 0 | 1 | 1 | 2 |
| 6221031240 | 2.45 | 0.7 |  | 1.19 | 0.96 | 1 | 0 | 1 |  | 0 | 0 | 2 | 1 | 1 |
| 6221031241 | 3.06 | 1.09 |  | 1.49 | 0.67 | 0 | 0 | 2 |  | 0 | 0 | 1 | 1 | 1 |
| 6221031242 | 2.65 | 1.05 |  | 1.16 | 1.41 | 0 | 1 | 1 |  | 0 | 0 | 1 | 1 | 1 |
| 6221031244 | 3.29 | 0.64 |  | 1.47 | 3.28 | 0 | 0 | 1 |  | 0 | 0 | 2 | 2 | 2 |
| 6221031246 | 2.64 | 0.85 |  | 1.27 | 0.51 | 0 | 0 | 1 |  | 0 | 0 | 3 | 2 | 2 |
| 6221031247 | 3.59 | 0.89 |  | 2.06 | 1.23 | 1 | 0 | 1 |  | 0 | 0 | 1 | 1 | 1 |
| 6221031249 | 2.13 | 0.66 |  | 0.95 | 0.73 | 0 | 0 | 1 |  | 0 | 0 | 1 | 1 | 1 |
| 6221031250 | 2.48 | 1 |  | 1 | 0.55 | 0 | 1 | 1 |  | 0 | 0 | 1 | 1 | 1 |
| 6221031305 | 2.65 | 0.59 |  | 1.27 | 1.81 | 1 | 0 | 1 |  | 0 | 0 | 2 | 1 | 1 |
| 6221031307 | 4.52 | 0.77 |  | 2.37 | 3.67 | 2 | 0 | 0 |  | 0 | 0 | 2 | 2 | 2 |
| 6221031308 | 3.97 | 0.88 |  | 2.37 | 1.71 | 1 | 1 | 2 |  | 0 | 1 | 1 | 1 | 1 |
| 6221031313 | 3.02 | 0.67 |  | 2.08 | 1.07 | 2 | 0 | 1 |  | 0 | 0 | 2 | 1 | 2 |
| 6221031316 | 2.47 | 0.61 |  | 1.33 | 1.09 | 2 | 1 | 1 |  | 0 | 1 | 1 | 1 | 1 |
| 6221031319 | 3.79 | 0.69 |  | 1.39 | 4.82 | 0 | 1 | 1 |  | 0 | 1 | 1 | 1 | 1 |
| 6221031323 | 4.33 | 1.19 |  | 2.18 | 1.66 | 0 | 0 | 1 |  | 0 | 0 | 1 | 1 | 1 |
| 6221031324 | 2.66 | 1.1 |  | 1.22 | 0.33 | 2 | 1 | 1 |  | 0 | 0 | 1 | 1 | 1 |
| 6221031325 | 3.75 | 0.94 |  | 2.13 | 1.38 | 1 | 1 | 1 |  | 1 | 1 | 3 | 2 | 2 |
| 6221031326 | 3.51 | 0.93 |  | 2.02 | 0.98 | 1 | 1 | 1 |  | 0 | 1 | 2 | 2 | 2 |
| 6221031327 | 2.05 | 0.99 |  | 0.83 | 0.34 | 0 | 1 | 2 |  | 1 | 1 | 1 | 1 | 1 |
| 6221031328 | 3.54 | 1.4 |  | 1.71 | 0.67 | 1 | 0 | 1 |  | 0 | 0 | 1 | 1 | 1 |
| 6221031330 | 2.8 | 0.86 |  | 1.44 | 0.64 | 0 | 0 | 2 |  | 0 | 0 | 2 | 2 | 2 |
| 6221031331 | 3.34 | 1.11 |  | 1.55 | 1.91 | 0 | 0 | 1 |  | 0 | 0 | 1 | 1 | 1 |
| 6221031334 | 3.61 | 1.18 |  | 1.88 | 0.98 | 0 | 1 | 2 |  | 0 | 0 | 2 | 2 | 2 |
| 6221031336 | 2.45 | 0.56 |  | 1.02 | 1.01 | 1 | 0 | 0 |  | 0 | 0 | 1 | 1 | 1 |
| 6221031338 | 3.57 | 1.01 |  | 1.98 | 1.34 | 1 | 0 | 2 |  | 0 | 0 | 3 | 2 | 2 |
| 6221031339 | 2.81 | 0.8 |  | 1.34 | 1.77 | 0 | 0 | 1 |  | 0 | 0 | 2 | 2 | 2 |
| 6221031340 | 3.68 | 1.07 |  | 1.96 | 0.84 | 2 | 1 | 1 |  | 0 | 1 | 1 | 1 | 1 |
| 6221031341 | 3.42 | 0.92 |  | 1.83 | 0.98 | 0 | 1 | 2 |  | 0 | 1 | 1 | 1 | 1 |
| 6221031342 | 3.89 | 1.5 |  | 1.88 | 0.77 | 0 | 1 | 2 |  | 0 | 0 | 1 | 1 | 1 |
| 6221031343 | 3.69 | 0.66 |  | 2.08 | 2.75 | 0 | 0 | 1 |  | 0 | 0 | 3 | 2 | 2 |
| 6221031346 | 2.99 | 1.12 |  | 1.21 | 1.16 | 0 | 1 | 1 |  | 0 | 0 | 1 | 1 | 1 |
| 6221032102 | 4.22 | 0.88 |  | 2.17 | 2.76 | 1 | 1 | 2 |  | 0 | 0 | 2 | 1 | 1 |
| 6221032103 | 2.8 | 1.23 |  | 1.13 | 0.78 | 0 | 1 | 1 |  | 0 | 1 | 1 | 1 | 1 |
| 6221032104 | 2.32 | 0.62 |  | 1.22 | 0.79 | 0 | 0 | 1 |  | 0 | 0 | 1 | 1 | 1 |
| 6221032105 | 2.02 | 0.7 |  | 0.92 | 0.59 | 0 | 0 | 1 |  | 0 | 0 | 1 | 1 | 1 |
| 6221032107 | 3.01 | 0.77 |  | 1.76 | 0.84 | 2 | 1 | 1 |  | 0 | 1 | 3 | 3 | 2 |
| 6221032108 | 2.32 | 0.7 |  | 1.1 | 0.78 | 0 | 1 | 0 |  | 0 | 1 | 1 | 1 | 1 |
| 6221032109 | 2.45 | 0.81 |  | 1.15 | 0.8 | 0 | 0 | 1 |  | 0 | 0 | 1 | 1 | 1 |
| 6221032112 | 2.94 | 0.93 |  | 1.69 | 0.52 | 0 | 0 | 1 |  | 0 | 0 | 1 | 1 | 1 |
| 6221032114 | 1.96 | 0.62 |  | 0.94 | 0.67 | 0 | 1 | 2 |  | 0 | 1 | 1 | 1 | 1 |
| 6221032116 | 3.48 | 1.18 |  | 1.89 | 0.45 | 0 | 1 | 0 |  | 0 | 1 | 1 | 1 | 1 |
| 6221032117 | 3.28 | 0.82 |  | 1.91 | 1.24 | 0 | 1 | 1 |  | 0 | 0 | 2 | 1 | 1 |
| 6221032118 | 3.36 | 0.7 |  | 1.97 | 1.73 | 2 | 1 | 2 |  | 0 | 0 | 2 | 1 | 1 |
| 6221032119 | 3.72 | 1.16 |  | 2.01 | 1.13 | 0 | 1 | 1 |  | 0 | 1 | 1 | 1 | 1 |
| 6221032120 | 3.15 | 0.78 |  | 1.38 | 1.93 | 0 | 0 | 1 |  | 0 | 0 | 1 | 1 | 1 |
| 6221032121 | 4.91 | 1.19 |  | 2.76 | 2.08 | 1 | 0 | 2 |  | 0 | 0 | 2 | 2 | 2 |
| 6221032122 | 3.19 | 0.95 |  | 1.79 | 1.12 | 2 | 1 | 1 |  | 0 | 1 | 1 | 1 | 2 |
| 6221032123 | 4.37 | 1.08 |  | 2.76 | 0.76 | 0 | 0 | 2 |  | 0 | 0 | 2 | 1 | 1 |
| 6221032125 | 2.45 | 0.51 |  | 1.29 | 1.17 | 0 | 1 | 1 |  | 0 | 1 | 2 | 1 | 2 |
| 6221032126 | 2.95 | 1.05 |  | 1.32 | 0.87 | 0 | 0 | 1 |  | 0 | 0 | 1 | 1 | 1 |
| 6221032127 | 2.64 | 0.57 |  | 1.64 | 0.84 | 2 | 0 | 0 |  | 0 | 0 | 2 | 2 | 3 |
| 6221032129 | 3.28 | 0.89 |  | 1.88 | 0.69 | 0 | 0 | 2 |  | 0 | 0 | 1 | 1 | 1 |
| 6221032130 | 2.91 | 0.95 |  | 1.44 | 0.91 | 1 | 1 | 1 |  | 0 | 0 | 2 | 1 | 1 |
| 6221032131 | 4.23 | 0.83 |  | 2.56 | 2.23 | 1 | 1 | 2 |  | 0 | 1 | 1 | 1 | 1 |
| 6221032132 | 3.92 | 0.9 |  | 2.15 | 1.54 | 0 | 1 | 1 |  | 0 | 0 | 2 | 1 | 2 |
| 6221032134 | 3.67 | 0.98 |  | 2.38 | 1.01 | 0 | 0 | 1 |  | 0 | 0 | 1 | 1 | 2 |
| 6221032135 | 3.17 | 1.05 |  | 1.62 | 0.89 | 0 | 0 | 1 |  | 0 | 0 | 1 | 1 | 1 |
| 6221032136 | 2.49 | 0.81 |  | 1.2 | 0.56 | 0 | 0 | 1 |  | 0 | 0 | 2 | 2 | 2 |
| 6221032137 | 3.74 | 0.87 |  | 1.65 | 3.92 | 0 | 1 | 1 |  | 0 | 0 | 2 | 2 | 2 |
| 6221032138 | 3.29 | 1.23 |  | 1.4 | 0.98 | 0 | 1 | 0 |  | 0 | 0 | 1 | 1 | 1 |
| 6221032140 | 3.92 | 1.11 |  | 2.19 | 1.33 | 2 | 1 | 1 |  | 0 | 0 | 2 | 2 | 2 |
| 6221032143 | 2.19 | 0.59 |  | 1.09 | 0.64 | 1 | 0 | 1 |  | 0 | 0 | 3 | 2 | 2 |
| 6221032145 | 3.32 | 0.7 |  | 1.85 | 1.23 | 0 | 0 | 1 |  | 0 | 0 | 1 | 1 | 1 |
| 6221032146 | 3.03 | 0.84 |  | 1.58 | 1.04 | 0 | 1 | 1 |  | 0 | 1 | 2 | 1 | 1 |
| 6221032148 | 3.32 | 1.07 |  | 1.71 | 0.51 | 0 | 1 | 2 |  | 0 | 0 | 1 | 1 | 1 |
| 6221032149 | 4.42 | 1.11 |  | 2.79 | 1.09 | 0 | 1 | 1 |  | 0 | 0 | 2 | 2 | 2 |
| 6221032150 | 2.97 | 1.07 |  | 1.4 | 0.61 | 0 | 1 | 1 |  | 0 | 1 | 1 | 1 | 1 |
| 6221032201 | 3.27 | 0.99 |  | 1.75 | 1.11 | 0 | 0 | 2 |  | 0 | 0 | 1 | 1 | 1 |
| 6221032202 | 2.05 | 0.71 |  | 0.89 | 0.58 | 0 | 0 | 0 |  | 0 | 0 | 1 | 1 | 1 |
| 6221032203 | 1.91 | 0.68 |  | 0.73 | 0.7 | 0 | 0 | 1 |  | 0 | 0 | 2 | 1 | 1 |
| 6221032205 | 2.79 | 0.81 |  | 1.56 | 0.75 | 0 | 1 | 2 |  | 1 | 1 | 1 | 1 | 1 |
| 6221032206 | 3.87 | 0.96 |  | 2.51 | 0.87 | 0 | 1 | 1 |  | 1 | 0 | 2 | 1 | 1 |
| 6221032207 | 2.7 | 0.85 |  | 1.28 | 0.56 | 0 | 1 | 1 |  | 0 | 0 | 1 | 1 | 1 |
| 6221032209 | 2.52 | 0.87 |  | 1.38 | 0.4 | 0 | 1 | 2 |  | 0 | 0 | 1 | 1 | 1 |
| 6221032210 | 2.66 | 0.81 |  | 1.25 | 0.81 | 0 | 0 | 1 |  | 0 | 0 | 1 | 1 | 1 |
| 6221032211 | 2.5 | 0.96 |  | 0.95 | 1.64 | 0 | 0 | 1 |  | 0 | 0 | 1 | 1 | 1 |
| 6221032212 | 3.02 | 1.01 |  | 1.34 | 1.32 | 0 | 1 | 1 |  | 1 | 1 | 1 | 1 | 1 |
| 6221032213 | 2.64 | 0.79 |  | 1.33 | 0.73 | 0 | 1 | 2 |  | 0 | 1 | 1 | 1 | 1 |
| 6221032214 | 2.71 | 0.83 |  | 1.49 | 0.62 | 0 | 1 | 1 |  | 0 | 1 | 2 | 2 | 3 |
| 6221032215 | 2.87 | 0.72 |  | 1.73 | 0.88 | 0 | 1 | 2 |  | 0 | 1 | 1 | 1 | 1 |
| 6221032216 | 2.4 | 0.61 |  | 1.16 | 1.5 | 0 | 0 | 1 |  | 0 | 0 | 1 | 1 | 1 |
| 6221032217 | 4.04 | 1.18 |  | 2.09 | 1.11 | 2 | 0 | 0 |  | 0 | 0 | 1 | 1 | 1 |
| 6221032219 | 2.84 | 0.73 |  | 1.61 | 0.75 | 0 | 0 | 1 |  | 0 | 0 | 3 | 2 | 2 |
| 6221032221 | 2.94 | 1.03 |  | 1.59 | 0.5 | 1 | 1 | 1 |  | 0 | 1 | 1 | 1 | 1 |
| 6221032222 | 3.49 | 0.7 |  | 1.87 | 2.57 | 1 | 1 | 2 |  | 0 | 1 | 3 | 3 | 3 |
| 6221032225 | 3.04 | 0.75 |  | 1.8 | 0.48 | 0 | 1 | 1 |  | 0 | 0 | 1 | 1 | 1 |
| 6221032226 | 2.67 | 0.79 |  | 1.39 | 0.73 | 2 | 0 | 0 |  | 0 | 0 | 1 | 1 | 2 |
| 6221032232 | 3.26 | 0.98 |  | 1.85 | 0.64 | 0 | 1 | 0 |  | 0 | 1 | 1 | 1 | 1 |
| 6221032233 | 3.15 | 1.14 |  | 1.56 | 0.47 | 0 | 0 | 1 |  | 0 | 0 | 2 | 2 | 2 |
| 6221032235 | 2.31 | 0.82 |  | 0.94 | 0.44 | 0 | 0 | 1 |  | 0 | 0 | 1 | 1 | 1 |
| 6221032236 | 2.76 | 0.62 |  | 1.33 | 1.24 | 0 | 1 | 1 |  | 0 | 0 | 2 | 2 | 2 |
| 6221032240 | 2.07 | 0.69 |  | 1.14 | 0.44 | 0 | 0 | 1 |  | 0 | 0 | 2 | 2 | 1 |
| 6221032243 | 2.48 | 0.65 |  | 1.28 | 0.8 | 0 | 0 | 2 |  | 0 | 0 | 1 | 1 | 1 |
| 6221032244 | 4.84 | 1.06 |  | 2.55 | 1.93 | 2 | 1 | 0 |  | 0 | 1 | 2 | 2 | 2 |
| 6221032247 | 3.05 | 0.7 |  | 1.5 | 1.56 | 0 | 0 | 1 |  | 0 | 0 | 3 | 2 | 3 |
| 6221032301 | 2.91 | 0.79 |  | 1.78 | 0.79 | 0 | 0 | 1 |  | 0 | 0 | 1 | 1 | 1 |
| 6221032305 | 4.75 | 1.14 |  | 2.71 | 1.47 | 0 | 1 | 2 |  | 1 | 1 | 1 | 1 | 1 |
| 6221032306 | 2.62 | 0.91 |  | 1.24 | 0.67 | 0 | 0 | 2 |  | 0 | 0 | 2 | 1 | 2 |
| 6221032307 | 1.83 | 0.51 |  | 0.75 | 0.49 | 0 | 0 | 2 |  | 0 | 0 | 1 | 1 | 1 |
| 6221032308 | 2.54 | 0.79 |  | 1.35 | 0.52 | 0 | 0 | 1 |  | 0 | 0 | 1 | 1 | 1 |
| 6221032309 | 3.09 | 1.06 |  | 1.52 | 0.82 | 0 | 0 | 2 |  | 0 | 0 | 2 | 1 | 1 |
| 6221032310 | 2.35 | 0.82 |  | 1.04 | 1.3 | 2 | 1 | 2 |  | 0 | 0 | 1 | 1 | 1 |
| 6221032311 | 3.04 | 0.77 |  | 1.43 | 1.59 | 1 | 1 | 1 |  | 0 | 0 | 1 | 1 | 1 |
| 6221032312 | 2.96 | 0.96 |  | 1.59 | 0.64 | 0 | 1 | 1 |  | 1 | 1 | 1 | 1 | 1 |
| 6221032313 | 3.12 | 0.78 |  | 1.76 | 1.11 | 1 | 1 | 2 |  | 0 | 0 | 1 | 1 | 1 |
| 6221032315 | 3.4 | 1 |  | 1.67 | 1.19 | 0 | 0 | 2 |  | 0 | 0 | 2 | 2 | 1 |
| 6221032316 | 4.64 | 1.1 |  | 2.79 | 1.57 | 1 | 1 | 2 |  | 0 | 1 | 3 | 2 | 3 |
| 6221032317 | 4.91 | 1.65 |  | 2.57 | 1.03 | 1 | 0 | 2 |  | 0 | 0 | 1 | 1 | 1 |
| 6221032318 | 2.19 | 0.57 |  | 1.07 | 0.78 | 2 | 1 | 1 |  | 0 | 1 | 1 | 1 | 1 |
| 6221032319 | 3.14 | 0.87 |  | 1.75 | 0.81 | 0 | 1 | 1 |  | 0 | 1 | 2 | 2 | 1 |
| 6221032320 | 2.24 | 0.54 |  | 1.16 | 0.89 | 0 | 1 | 1 |  | 0 | 0 | 1 | 1 | 1 |
| 6221032322 | 2.65 | 1.06 |  | 1.2 | 0.42 | 0 | 0 | 2 |  | 0 | 0 | 2 | 2 | 1 |
| 6221032323 | 3.45 | 0.9 |  | 1.95 | 1.14 | 0 | 0 | 0 |  | 0 | 0 | 2 | 2 | 2 |
| 6221032324 | 1.63 | 0.63 |  | 0.77 | 0.41 | 0 | 1 | 2 |  | 0 | 0 | 1 | 1 | 1 |
| 6221032329 | 3.37 | 0.91 |  | 1.96 | 0.71 | 0 | 0 | 2 |  | 0 | 0 | 1 | 1 | 1 |
| 6221032331 | 3.49 | 1.06 |  | 1.72 | 0.77 | 2 | 0 | 0 |  | 0 | 0 | 1 | 1 | 1 |
| 6221032332 | 2.69 | 1.17 |  | 1.03 | 0.63 | 0 | 0 | 2 |  | 1 | 0 | 1 | 1 | 1 |
| 6221032333 | 2.11 | 0.75 |  | 1.02 | 0.38 | 0 | 0 | 1 |  | 0 | 0 | 2 | 1 | 1 |
| 6221032334 | 3.21 | 0.98 |  | 1.84 | 0.63 | 2 | 1 | 0 |  | 0 | 1 | 1 | 1 | 1 |
| 6221032335 | 1.94 | 0.72 |  | 0.87 | 0.54 | 0 | 1 | 2 |  | 0 | 0 | 1 | 1 | 1 |
| 6221032336 | 2.88 | 0.79 |  | 1.63 | 0.65 | 0 | 1 | 1 |  | 0 | 0 | 1 | 1 | 1 |
| 6221032340 | 2.56 | 0.96 |  | 1.09 | 0.69 | 0 | 0 | 1 |  | 0 | 0 | 3 | 2 | 3 |
| 6221032341 | 1.98 | 0.82 |  | 0.8 | 0.69 | 0 | 0 | 2 |  | 0 | 0 | 1 | 1 | 1 |
| 6221032342 | 2.73 | 0.86 |  | 1.35 | 0.63 | 0 | 1 | 1 |  | 0 | 1 | 2 | 1 | 1 |
| 6221032343 | 3.05 | 0.76 |  | 1.68 | 0.97 | 2 | 1 | 0 |  | 0 | 1 | 2 | 2 | 1 |
| 6221032344 | 3.46 | 0.76 |  | 1.89 | 2.34 | 0 | 1 | 2 |  | 0 | 0 | 2 | 1 | 1 |
| 6221032346 | 2.24 | 0.87 |  | 0.92 | 0.51 | 0 | 0 | 1 |  | 0 | 0 | 1 | 1 | 1 |
| 6221032347 | 3.4 | 0.97 |  | 1.83 | 1.18 | 0 | 1 | 2 |  | 0 | 1 | 1 | 1 | 1 |
| 6221032348 | 2.03 | 0.76 |  | 0.76 | 0.88 | 1 | 1 | 1 |  | 0 | 1 | 1 | 1 | 1 |
| 6221033101 | 2.78 | 0.73 |  | 1.47 | 0.83 | 0 | 1 | 1 |  | 0 | 1 | 1 | 1 | 1 |
| 6221033105 | 3.2 | 1.06 |  | 1.6 | 0.8 | 0 | 0 | 1 |  | 0 | 0 | 2 | 1 | 2 |
| 6221033106 | 2.93 | 0.86 |  | 1.57 | 0.65 | 0 | 0 | 1 |  | 0 | 0 | 2 | 2 | 2 |
| 6221033107 | 3.13 | 0.99 |  | 1.61 | 0.71 | 1 | 0 | 0 |  | 0 | 0 | 2 | 2 | 3 |
| 6221033108 | 3.81 | 1.38 |  | 1.8 | 1.73 | 0 | 0 | 1 |  | 0 | 0 | 1 | 1 | 1 |
| 6221033110 | 2.32 | 0.68 |  | 1.17 | 1.18 | 0 | 1 | 1 |  | 0 | 1 | 1 | 1 | 1 |
| 6221033112 | 2.22 | 0.71 |  | 0.98 | 0.87 | 1 | 0 | 1 |  | 0 | 0 | 1 | 1 | 1 |
| 6221033114 | 2.44 | 0.82 |  | 1.11 | 0.89 | 0 | 0 | 1 |  | 0 | 0 | 2 | 1 | 2 |
| 6221033117 | 2.44 | 0.64 |  | 1.09 | 1.71 | 0 | 1 | 2 |  | 1 | 1 | 2 | 2 | 2 |
| 6221033124 | 4.58 | 1.31 |  | 2.95 | 1.27 | 0 | 1 | 1 |  | 0 | 1 | 3 | 2 | 2 |
| 6221033125 | 3.22 | 0.84 |  | 2.1 | 0.99 | 0 | 0 | 2 |  | 0 | 0 | 2 | 2 | 2 |
| 6221033126 | 2.49 | 0.92 |  | 0.92 | 0.92 | 0 | 1 | 2 |  | 0 | 1 | 2 | 1 | 2 |
| 6221033128 | 3.32 | 0.99 |  | 1.81 | 0.48 | 0 | 1 | 2 |  | 0 | 1 | 1 | 1 | 1 |
| 6221033130 | 2.66 | 0.99 |  | 1.3 | 0.51 | 0 | 0 | 2 |  | 0 | 0 | 1 | 1 | 1 |
| 6221033131 | 4.94 | 0.97 |  | 3.27 | 1.94 | 1 | 0 | 2 |  | 0 | 0 | 1 | 1 | 1 |
| 6221033135 | 4.66 | 0.95 |  | 2.22 | 5.7 | 0 | 1 | 2 |  | 1 | 1 | 3 | 2 | 3 |
| 6221033138 | 3.29 | 0.96 |  | 1.81 | 0.97 | 0 | 0 | 1 |  | 0 | 0 | 2 | 1 | 1 |
| 6221033142 | 3.03 | 0.93 |  | 1.76 | 0.71 | 0 | 1 | 1 |  | 0 | 1 | 1 | 1 | 1 |
| 6221033146 | 3.44 | 1.21 |  | 1.82 | 0.88 | 0 | 0 | 1 |  | 0 | 0 | 3 | 2 | 2 |
| 6221033147 | 4.99 | 0.94 |  | 2.91 | 2.04 | 1 | 0 | 2 |  | 0 | 0 | 2 | 2 | 2 |
| 6221033203 | 2.37 | 0.9 |  | 1.07 | 0.54 | 0 | 0 | 1 |  | 0 | 0 | 1 | 1 | 1 |
| 6221033204 | 3.88 | 1.44 |  | 2.04 | 0.71 | 0 | 0 | 1 |  | 0 | 0 | 2 | 1 | 2 |
| 6221033210 | 2.74 | 0.8 |  | 1.08 | 1.84 | 0 | 0 | 1 |  | 0 | 0 | 2 | 2 | 2 |
| 6221033212 | 3.87 | 0.73 |  | 2.12 | 1.76 | 1 | 0 | 0 |  | 0 | 0 | 3 | 2 | 3 |
| 6221033213 | 2.82 | 0.89 |  | 1.36 | 0.93 | 0 | 0 | 1 |  | 0 | 0 | 2 | 1 | 2 |
| 6221033217 | 3.3 | 0.74 |  | 1.99 | 0.85 | 0 | 1 | 1 |  | 1 | 0 | 2 | 2 | 1 |
| 6221033219 | 3.65 | 1.21 |  | 1.89 | 0.83 | 0 | 0 | 1 |  | 0 | 0 | 1 | 1 | 1 |
| 6221033220 | 4.28 | 1.32 |  | 2.49 | 1.15 | 0 | 0 | 1 |  | 1 | 0 | 2 | 2 | 2 |
| 6221033221 | 3.68 | 1.04 |  | 1.96 | 1.47 | 0 | 0 | 1 |  | 0 | 0 | 2 | 2 | 2 |
| 6221033233 | 4.24 | 0.74 |  | 1.93 | 5.08 | 0 | 1 | 1 |  | 0 | 0 | 3 | 3 | 3 |
| 6221033235 | 4.8 | 1.48 |  | 2.39 | 1.82 | 0 | 0 | 1 |  | 0 | 0 | 1 | 1 | 1 |
| 6221033237 | 2.64 | 0.59 |  | 1.47 | 1.03 | 0 | 0 | 1 |  | 0 | 0 | 3 | 2 | 1 |
| 6221033238 | 2.63 | 0.71 |  | 1.31 | 1.03 | 2 | 1 | 0 |  | 0 | 0 | 1 | 1 | 1 |
| 6221033243 | 1.56 | 0.63 |  | 0.62 | 0.42 | 0 | 0 | 1 |  | 0 | 0 | 2 | 2 | 1 |
| 6221033246 | 3.93 | 1.02 |  | 2.24 | 1.47 | 2 | 1 | 1 |  | 1 | 1 | 2 | 2 | 2 |
| 6221033247 | 3.86 | 1.34 |  | 1.97 | 0.95 | 0 | 0 | 1 |  | 0 | 0 | 2 | 1 | 1 |
| 6221033249 | 3.69 | 0.85 |  | 2.09 | 1.16 | 0 | 1 | 1 |  | 0 | 0 | 2 | 2 | 1 |
| 6221033304 | 3.23 | 0.71 |  | 1.66 | 1.17 | 1 | 1 | 0 |  | 0 | 1 | 2 | 2 | 2 |
| 6221033306 | 2.67 | 0.88 |  | 1.38 | 0.58 | 0 | 1 | 1 |  | 0 | 1 | 2 | 2 | 1 |
| 6221033307 | 3.94 | 0.79 |  | 1.86 | 5.07 | 0 | 1 | 1 |  | 0 | 1 | 2 | 1 | 2 |
| 6221033308 | 3.83 | 1.57 |  | 1.8 | 0.8 | 0 | 1 | 1 |  | 1 | 1 | 1 | 1 | 1 |
| 6221033310 | 2.99 | 0.82 |  | 1.39 | 1.92 | 0 | 0 | 1 |  | 0 | 0 | 1 | 1 | 1 |
| 6221033311 | 2.19 | 0.5 |  | 1.05 | 1.83 | 1 | 1 | 2 |  | 0 | 1 | 3 | 2 | 1 |
| 6221033312 | 3.82 | 1.54 |  | 1.61 | 1.14 | 1 | 1 | 1 |  | 1 | 1 | 2 | 2 | 2 |
| 6221033314 | 4.79 | 0.84 |  | 2.98 | 1.48 | 0 | 1 | 2 |  | 0 | 0 | 2 | 2 | 2 |
| 6221033316 | 2.3 | 0.63 |  | 1.12 | 1.21 | 0 | 0 | 1 |  | 0 | 0 | 1 | 1 | 2 |
| 6221033319 | 3.43 | 1.24 |  | 1.66 | 0.97 | 0 | 1 | 1 |  | 0 | 0 | 1 | 1 | 1 |
| 6221033321 | 3.63 | 1.2 |  | 1.87 | 1.17 | 0 | 0 | 2 |  | 0 | 0 | 1 | 1 | 1 |
| 6221033326 | 3.7 | 1.32 |  | 1.9 | 0.87 | 0 | 0 | 1 |  | 0 | 0 | 1 | 1 | 1 |
| 6221033327 | 2.44 | 0.98 |  | 0.93 | 0.77 | 0 | 0 | 2 |  | 0 | 0 | 2 | 2 | 2 |
| 6221033329 | 0.94 | 0.28 |  | 0.37 | 0.54 | 1 | 1 | 0 |  | 0 | 0 | 1 | 1 | 1 |
| 6221033331 | 3.87 | 0.91 |  | 2.24 | 2.56 | 1 | 0 | 0 |  | 0 | 0 | 2 | 2 | 2 |
| 6221033334 | 2.35 | 0.85 |  | 1.21 | 0.57 | 0 | 0 | 2 |  | 0 | 0 | 1 | 1 | 1 |
| 6221033344 | 4.96 | 1.48 |  | 2.99 | 0.9 | 0 | 1 | 1 |  | 1 | 1 | 1 | 1 | 1 |
| 6221033345 | 4.12 | 1.26 |  | 2.17 | 1.46 | 1 | 0 | 1 |  | 0 | 0 | 1 | 1 | 1 |
| 6221033347 | 2.32 | 0.58 |  | 1.26 | 0.86 | 2 | 0 | 0 |  | 0 | 0 | 1 | 1 | 2 |
| 6221034101 | 4.22 | 1.31 |  | 2.41 | 1.21 | 0 | 0 | 1 |  | 0 | 0 | 1 | 1 | 1 |
| 6221034102 | 2.78 | 0.57 |  | 1.37 | 2.43 | 0 | 1 | 2 |  | 1 | 1 | 3 | 2 | 3 |
| 6221034103 | 2.51 | 1.06 |  | 0.7 | 1.06 | 0 | 0 | 1 |  | 0 | 0 | 1 | 1 | 1 |
| 6221034104 | 4.47 | 1.21 |  | 2.66 | 1.32 | 0 | 0 | 1 |  | 0 | 0 | 2 | 2 | 2 |
| 6221034107 | 3.97 | 1.34 |  | 1.95 | 0.87 | 0 | 0 | 2 |  | 0 | 0 | 1 | 1 | 1 |
| 6221034111 | 3.24 | 1.56 |  | 1.28 | 0.63 | 1 | 0 | 2 |  | 0 | 0 | 1 | 1 | 1 |
| 6221034113 | 3.55 | 0.8 |  | 1.74 | 2.53 | 0 | 1 | 2 |  | 1 | 1 | 1 | 1 | 1 |
| 6221034114 | 3.06 | 1.23 |  | 1.21 | 0.6 | 2 | 0 | 0 |  | 0 | 0 | 1 | 1 | 1 |
| 6221034115 | 4.06 | 1.25 |  | 2.19 | 1.21 | 0 | 0 | 1 |  | 0 | 0 | 1 | 1 | 1 |
| 6221034116 | 5.02 | 1.15 |  | 3.08 | 1.36 | 0 | 0 | 1 |  | 0 | 0 | 2 | 2 | 2 |
| 6221034119 | 4.39 | 1.16 |  | 2.6 | 1.82 | 0 | 1 | 2 |  | 1 | 1 | 2 | 2 | 2 |
| 6221034120 | 3.79 | 1.02 |  | 1.83 | 2.95 | 0 | 1 | 2 |  | 1 | 1 | 1 | 1 | 1 |
| 6221034121 | 3.27 | 0.72 |  | 1.53 | 2.75 | 2 | 1 | 2 |  | 0 | 0 | 2 | 2 | 2 |
| 6221034122 | 4.4 | 0.85 |  | 2.5 | 2.44 | 1 | 1 | 2 |  | 0 | 1 | 2 | 1 | 1 |
| 6221034123 | 4.39 | 0.97 |  | 2.85 | 1.45 | 0 | 0 | 2 |  | 0 | 0 | 2 | 1 | 1 |
| 6221034124 | 2.99 | 0.95 |  | 1.55 | 1.46 | 0 | 0 | 2 |  | 0 | 0 | 2 | 1 | 2 |
| 6221034125 | 4.16 | 1.6 |  | 2.02 | 0.64 | 1 | 0 | 2 |  | 0 | 0 | 2 | 1 | 1 |
| 6221034127 | 4.45 | 0.8 |  | 3.14 | 1.68 | 0 | 1 | 2 |  | 1 | 1 | 3 | 3 | 3 |
| 6221034128 | 2.88 | 0.9 |  | 1.5 | 1.45 | 0 | 1 | 2 |  | 1 | 1 | 3 | 2 | 2 |
| 6221034129 | 2.89 | 1.09 |  | 1.33 | 0.5 | 0 | 0 | 2 |  | 0 | 0 | 2 | 1 | 1 |
| 6221034130 | 3.33 | 1.17 |  | 1.84 | 0.59 | 0 | 0 | 2 |  | 0 | 0 | 2 | 1 | 1 |
| 6221034131 | 5.22 | 1.11 |  | 3.38 | 1.32 | 1 | 0 | 2 |  | 1 | 0 | 1 | 1 | 1 |
| 6221034134 | 3.05 | 0.86 |  | 1.5 | 1.28 | 1 | 1 | 2 |  | 0 | 0 | 1 | 1 | 2 |
| 6221034135 | 3.42 | 0.83 |  | 1.91 | 1.61 | 0 | 1 | 2 |  | 1 | 0 | 3 | 2 | 3 |
| 6221034137 | 3.43 | 1.08 |  | 1.74 | 1.28 | 0 | 0 | 2 |  | 0 | 0 | 2 | 2 | 1 |
| 6221034138 | 3.29 | 1.38 |  | 1.42 | 0.57 | 1 | 0 | 2 |  | 0 | 0 | 1 | 1 | 1 |
| 6221034139 | 3.47 | 0.96 |  | 1.66 | 1.94 | 0 | 1 | 2 |  | 0 | 0 | 1 | 1 | 1 |
| 6221034141 | 3.27 | 1.2 |  | 1.54 | 0.44 | 0 | 0 | 2 |  | 0 | 0 | 1 | 1 | 2 |
| 6221034145 | 3.49 | 0.79 |  | 1.77 | 2.35 | 1 | 1 | 2 |  | 1 | 0 | 3 | 3 | 3 |
| 6221034147 | 3.71 | 1.51 |  | 1.66 | 0.83 | 1 | 0 | 2 |  | 0 | 0 | 2 | 2 | 3 |
| 6221034148 | 4.22 | 0.98 |  | 1.98 | 3.7 | 0 | 1 | 1 |  | 0 | 1 | 2 | 2 | 2 |
| 6221034149 | 5.54 | 1.05 |  | 3.94 | 1.2 | 0 | 0 | 1 |  | 0 | 0 | 1 | 1 | 1 |
| 6221034150 | 4.88 | 1.03 |  | 3.17 | 1.98 | 2 | 0 | 2 |  | 0 | 0 | 3 | 2 | 3 |
| 6221034203 | 3.45 | 1.03 |  | 1.98 | 1.19 | 0 | 0 | 0 |  | 0 | 0 | 3 | 2 | 3 |
| 6221034206 | 4.33 | 0.99 |  | 2.62 | 1.68 | 0 | 1 | 0 |  | 0 | 0 | 2 | 2 | 2 |
| 6221034207 | 4.32 | 1.23 |  | 2 | 1.53 | 1 | 0 | 0 |  | 0 | 0 | 2 | 2 | 2 |
| 6221034212 | 1.85 | 0.67 |  | 0.81 | 0.65 | 0 | 0 | 2 |  | 0 | 0 | 2 | 1 | 2 |
| 6221034213 | 3.57 | 1.28 |  | 1.55 | 1.1 | 2 | 0 | 1 |  | 0 | 0 | 3 | 3 | 3 |
| 6221034216 | 2.97 | 1.08 |  | 1.49 | 0.96 | 0 | 0 | 2 |  | 0 | 0 | 1 | 1 | 1 |
| 6221034217 | 4.26 | 1.03 |  | 2.26 | 1.93 | 0 | 1 | 1 |  | 0 | 1 | 2 | 2 | 2 |
| 6221034221 | 3.73 | 1.25 |  | 2.07 | 0.82 | 0 | 0 | 2 |  | 0 | 0 | 1 | 1 | 1 |
| 6221034223 | 4.71 | 0.91 |  | 3.09 | 1.64 | 0 | 1 | 1 |  | 0 | 0 | 3 | 2 | 3 |
| 6221034224 | 4.25 | 1.11 |  | 2.63 | 1.18 | 0 | 1 | 1 |  | 0 | 1 | 1 | 1 | 1 |
| 6221034227 | 5.58 | 0.93 |  | 3.81 | 1.87 | 1 | 1 | 2 |  | 0 | 1 | 2 | 2 | 2 |
| 6221034228 | 3.77 | 1.39 |  | 1.79 | 0.67 | 1 | 0 | 1 |  | 0 | 0 | 1 | 1 | 1 |
| 6221034231 | 4.29 | 1.27 |  | 2.56 | 1.4 | 0 | 0 | 2 |  | 0 | 0 | 2 | 2 | 3 |
| 6221034232 | 1.88 | 0.75 |  | 0.87 | 0.43 | 0 | 1 | 2 |  | 1 | 1 | 1 | 1 | 1 |
| 6221034235 | 2.37 | 0.78 |  | 1.06 | 1.28 | 1 | 0 | 2 |  | 0 | 0 | 1 | 1 | 1 |
| 6221034236 | 3.2 | 1.26 |  | 1.41 | 0.81 | 1 | 0 | 1 |  | 0 | 0 | 1 | 1 | 1 |
| 6221034237 | 3.18 | 1.35 |  | 1.38 | 0.58 | 0 | 0 | 2 |  | 0 | 0 | 1 | 1 | 1 |
| 6221034239 | 4.24 | 1.37 |  | 2.28 | 1.24 | 0 | 0 | 1 |  | 0 | 0 | 2 | 2 | 2 |
| 6221034240 | 3.02 | 0.68 |  | 1.6 | 1.09 | 2 | 1 | 1 |  | 1 | 1 | 2 | 1 | 2 |
| 6221034241 | 4.18 | 0.82 |  | 2.55 | 2.35 | 0 | 1 | 2 |  | 1 | 0 | 3 | 2 | 3 |
| 6221034242 | 4.88 | 1.13 |  | 3.21 | 1.11 | 1 | 0 | 2 |  | 0 | 0 | 1 | 1 | 2 |
| 6221034243 | 3.51 | 0.98 |  | 2.24 | 0.74 | 0 | 0 | 2 |  | 0 | 0 | 2 | 1 | 2 |
| 6221034247 | 3.64 | 1.05 |  | 1.88 | 1.18 | 0 | 0 | 2 |  | 0 | 0 | 1 | 1 | 2 |
| 6221034248 | 2.21 | 0.64 |  | 1.06 | 0.85 | 1 | 1 | 2 |  | 1 | 1 | 1 | 1 | 1 |
| 6221034249 | 2.94 | 0.81 |  | 1.62 | 0.77 | 0 | 1 | 2 |  | 0 | 0 | 2 | 1 | 1 |
| 6221034301 | 5.02 | 1.43 |  | 3.54 | 1.27 | 2 | 0 | 1 |  | 0 | 0 | 1 | 1 | 1 |
| 6221034308 | 3.77 | 0.84 |  | 2.1 | 1.89 | 1 | 1 | 1 |  | 0 | 1 | 2 | 2 | 2 |
| 6221034310 | 3.25 | 0.92 |  | 1.72 | 1.05 | 1 | 0 | 2 |  | 0 | 0 | 1 | 1 | 1 |
| 6221034312 | 2.81 | 0.94 |  | 1.37 | 0.92 | 0 | 0 | 2 |  | 0 | 0 | 2 | 1 | 1 |
| 6221034316 | 6.71 | 0.76 |  | 3.39 | 5.39 | 0 | 1 | 2 |  | 0 | 1 | 2 | 2 | 2 |
| 6221034317 | 3.07 | 1.01 |  | 1.5 | 0.97 | 2 | 1 | 2 |  | 0 | 0 | 2 | 2 | 1 |
| 6221034320 | 3.72 | 1.01 |  | 2.1 | 0.59 | 1 | 0 | 1 |  | 0 | 0 | 1 | 1 | 1 |
| 6221034328 | 4.19 | 0.87 |  | 2.61 | 1.44 | 1 | 1 | 2 |  | 0 | 1 | 2 | 2 | 2 |
| 6221034332 | 4.76 | 0.94 |  | 2.67 | 3.48 | 2 | 1 | 1 |  | 0 | 0 | 2 | 1 | 2 |
| 6221034342 | 3.38 | 0.98 |  | 1.6 | 1.6 | 0 | 0 | 1 |  | 0 | 0 | 2 | 2 | 2 |
| 6221034343 | 3.69 | 0.95 |  | 2.31 | 1 | 0 | 0 | 2 |  | 0 | 0 | 1 | 1 | 1 |
| 6221034344 | 4.05 | 1.29 |  | 2.25 | 1.25 | 0 | 0 | 2 |  | 0 | 0 | 1 | 1 | 1 |
| 6221034345 | 3.95 | 1.09 |  | 2.26 | 1.03 | 1 | 0 | 2 |  | 0 | 0 | 2 | 1 | 1 |
| 6221034349 | 3.57 | 1.03 |  | 2.04 | 0.94 | 2 | 1 | 0 |  | 0 | 0 | 1 | 1 | 1 |
| 6222011102 | 3.52 | 0.98 |  | 1.84 | 1.12 | 0 | 1 | 1 |  | 0 | 0 | 1 | 1 | 1 |
| 6222011104 | 3.38 | 1.2 |  | 1.61 | 0.72 | 0 | 0 | 0 |  | 0 | 0 | 1 | 1 | 1 |
| 6222011105 | 5.05 | 0.94 |  | 2.44 | 5.04 | 0 | 0 | 1 |  | 0 | 0 | 2 | 2 | 2 |
| 6222011106 | 3 | 0.85 |  | 1.33 | 1.44 | 0 | 0 | 1 |  | 0 | 0 | 2 | 2 | 2 |
| 6222011107 | 2.21 | 0.74 |  | 0.9 | 1.45 | 0 | 1 | 0 |  | 0 | 1 | 1 | 1 | 1 |
| 6222011110 | 3.45 | 0.92 |  | 1.83 | 1.11 | 0 | 1 | 1 |  | 0 | 0 | 1 | 1 | 1 |
| 6222011111 | 2.48 | 0.84 |  | 1.12 | 0.99 | 0 | 0 | 2 |  | 0 | 0 | 1 | 1 | 1 |
| 6222011112 | 4.72 | 1.34 |  | 2.77 | 0.8 | 1 | 0 | 1 |  | 0 | 0 | 2 | 2 | 3 |
| 6222011113 | 4.33 | 1.13 |  | 2.15 | 1.49 | 1 | 0 | 0 |  | 0 | 0 | 2 | 1 | 1 |
| 6222011114 | 2.22 | 0.78 |  | 0.83 | 1.47 | 0 | 1 | 2 |  | 0 | 0 | 2 | 1 | 1 |
| 6222011116 | 4 | 1.06 |  | 2.4 | 0.69 | 1 | 1 | 1 |  | 0 | 1 | 1 | 1 | 1 |
| 6222011118 | 3.77 | 1.1 |  | 1.91 | 1.27 | 0 | 1 | 1 |  | 1 | 1 | 1 | 1 | 1 |
| 6222011119 | 2.55 | 1.06 |  | 1.02 | 0.58 | 0 | 0 | 1 |  | 0 | 0 | 1 | 1 | 1 |
| 6222011123 | 3.49 | 0.94 |  | 1.94 | 0.91 | 1 | 1 | 0 |  | 0 | 1 | 1 | 1 | 1 |
| 6222011125 | 1.92 | 0.75 |  | 0.7 | 0.51 | 0 | 1 | 1 |  | 1 | 1 | 2 | 1 | 1 |
| 6222011126 | 2.96 | 1.05 |  | 1.42 | 1.04 | 0 | 1 | 1 |  | 1 | 1 | 2 | 2 | 2 |
| 6222011127 | 2.87 | 1 |  | 1.49 | 0.73 | 0 | 0 | 1 |  | 0 | 0 | 1 | 1 | 1 |
| 6222011130 | 3.46 | 0.93 |  | 1.89 | 1.88 | 1 | 1 | 1 |  | 1 | 1 | 2 | 2 | 1 |
| 6222011133 | 4.33 | 1.16 |  | 2.58 | 1.11 | 0 | 1 | 1 |  | 1 | 1 | 2 | 1 | 2 |
| 6222011135 | 3.31 | 0.85 |  | 1.81 | 1.77 | 0 | 1 | 2 |  | 1 | 1 | 3 | 2 | 3 |
| 6222011137 | 3.18 | 0.95 |  | 1.51 | 1.55 | 0 | 0 | 1 |  | 0 | 0 | 1 | 1 | 1 |
| 6222011142 | 3.74 | 1.23 |  | 1.94 | 0.93 | 0 | 1 | 1 |  | 1 | 0 | 1 | 1 | 1 |
| 6222011146 | 5.91 | 1.03 |  | 2.8 | 4.77 | 2 | 0 | 0 |  | 0 | 0 | 2 | 2 | 1 |
| 6222011202 | 1.66 | 0.34 |  | 0.8 | 2.36 | 0 | 0 | 0 |  | 0 | 0 | 2 | 1 | 1 |
| 6222011203 | 5.13 | 1.65 |  | 2.78 | 1.23 | 0 | 0 | 1 |  | 0 | 0 | 2 | 2 | 2 |
| 6222011206 | 4.24 | 1.27 |  | 1.98 | 1.28 | 0 | 0 | 1 |  | 0 | 0 | 1 | 1 | 1 |
| 6222011209 | 3.51 | 1.15 |  | 1.82 | 0.64 | 0 | 0 | 1 |  | 1 | 0 | 2 | 1 | 1 |
| 6222011210 | 3.68 | 1.19 |  | 1.92 | 0.9 | 1 | 0 | 0 |  | 0 | 0 | 1 | 1 | 1 |
| 6222011215 | 2.88 | 0.9 |  | 1.44 | 0.81 | 2 | 1 | 0 |  | 1 | 0 | 1 | 1 | 1 |
| 6222011216 | 4.5 | 1.25 |  | 2.33 | 1.28 | 2 | 0 | 0 |  | 0 | 0 | 2 | 2 | 1 |
| 6222011217 | 2.2 | 0.72 |  | 1.02 | 1 | 0 | 0 | 1 |  | 0 | 0 | 1 | 1 | 1 |
| 6222011218 | 3.52 | 1.39 |  | 1.44 | 0.57 | 0 | 0 | 1 |  | 0 | 0 | 1 | 1 | 1 |
| 6222011219 | 3.34 | 1.39 |  | 1.39 | 0.55 | 0 | 0 | 1 |  | 0 | 0 | 1 | 1 | 1 |
| 6222011220 | 4.41 | 1.09 |  | 2.54 | 1.13 | 0 | 0 | 0 |  | 1 | 0 | 2 | 1 | 1 |
| 6222011221 | 3.2 | 1.13 |  | 1.19 | 0.83 | 0 | 0 | 1 |  | 0 | 0 | 1 | 1 | 1 |
| 6222011222 | 2.66 | 0.65 |  | 1.35 | 1.82 | 1 | 1 | 0 |  | 0 | 0 | 1 | 1 | 1 |
| 6222011224 | 4.27 | 0.87 |  | 2.45 | 2.37 | 2 | 0 | 0 |  | 0 | 0 | 1 | 1 | 2 |
| 6222011226 | 2.75 | 0.91 |  | 1.44 | 0.61 | 0 | 0 | 1 |  | 0 | 0 | 2 | 1 | 1 |
| 6222011227 | 2.08 | 0.82 |  | 0.81 | 0.61 | 0 | 0 | 1 |  | 0 | 0 | 2 | 2 | 2 |
| 6222011229 | 3.05 | 1.17 |  | 1.36 | 0.72 | 0 | 0 | 1 |  | 0 | 0 | 2 | 2 | 2 |
| 6222011230 | 3.11 | 0.68 |  | 1.66 | 1.66 | 0 | 1 | 1 |  | 0 | 0 | 1 | 1 | 1 |
| 6222011231 | 2.6 | 0.65 |  | 1.25 | 1.21 | 0 | 0 | 1 |  | 0 | 0 | 2 | 1 | 1 |
| 6222011233 | 2.48 | 0.75 |  | 1.24 | 1 | 0 | 0 | 0 |  | 0 | 0 | 3 | 2 | 2 |
| 6222011234 | 5.22 | 1.2 |  | 2.51 | 3.11 | 0 | 0 | 1 |  | 0 | 0 | 1 | 1 | 2 |
| 6222011235 | 2.83 | 1.04 |  | 1.2 | 1.06 | 0 | 0 | 1 |  | 0 | 0 | 1 | 1 | 1 |
| 6222011236 | 2.8 | 0.9 |  | 1.29 | 1.17 | 0 | 0 | 1 |  | 0 | 0 | 1 | 1 | 2 |
| 6222011237 | 4.18 | 0.96 |  | 2.38 | 1.34 | 2 | 1 | 0 |  | 0 | 1 | 2 | 2 | 1 |
| 6222011240 | 4.81 | 2.16 |  | 1.76 | 0.68 | 0 | 0 | 1 |  | 0 | 0 | 1 | 1 | 1 |
| 6222011241 | 4.18 | 1.18 |  | 1.96 | 2.63 | 1 | 0 | 0 |  | 0 | 0 | 1 | 1 | 1 |
| 6222011242 | 3.35 | 1.16 |  | 1.58 | 1.16 | 0 | 0 | 1 |  | 0 | 0 | 1 | 1 | 1 |
| 6222011243 | 4.76 | 1.45 |  | 2.58 | 0.93 | 0 | 0 | 1 |  | 0 | 0 | 1 | 1 | 2 |
| 6222011244 | 2.9 | 0.99 |  | 1.34 | 1.12 | 0 | 0 | 1 |  | 0 | 0 | 2 | 1 | 2 |
| 6222011245 | 3.02 | 0.98 |  | 1.5 | 0.57 | 0 | 1 | 0 |  | 0 | 1 | 2 | 1 | 1 |
| 6222011246 | 4 | 1.26 |  | 2.07 | 0.95 | 0 | 0 | 0 |  | 0 | 0 | 2 | 2 | 1 |
| 6222011247 | 3.01 | 0.99 |  | 1.36 | 1.41 | 0 | 1 | 1 |  | 1 | 1 | 1 | 1 | 1 |
| 6222011249 | 2.24 | 0.58 |  | 1.26 | 0.59 | 0 | 0 | 2 |  | 0 | 0 | 1 | 1 | 1 |
| 6222011250 | 3.72 | 0.93 |  | 1.73 | 1.83 | 2 | 0 | 0 |  | 0 | 0 | 1 | 1 | 1 |
| 6222011301 | 3.82 | 0.89 |  | 2.22 | 1.44 | 1 | 0 | 1 |  | 0 | 0 | 1 | 1 | 1 |
| 6222011302 | 3.18 | 0.93 |  | 1.39 | 1.22 | 0 | 0 | 1 |  | 0 | 0 | 1 | 1 | 1 |
| 6222011304 | 3.43 | 1.09 |  | 1.69 | 1.27 | 0 | 0 | 1 |  | 0 | 0 | 1 | 1 | 1 |
| 6222011305 | 3.65 | 0.86 |  | 1.93 | 1.32 | 2 | 0 | 0 |  | 0 | 0 | 2 | 2 | 2 |
| 6222011306 | 5.2 | 1.38 |  | 2.8 | 1.08 | 2 | 0 | 1 |  | 0 | 0 | 1 | 1 | 1 |
| 6222011308 | 2.48 | 0.74 |  | 1.24 | 0.66 | 1 | 1 | 0 |  | 0 | 1 | 1 | 1 | 1 |
| 6222011309 | 3.3 | 0.79 |  | 2.01 | 0.76 | 0 | 1 | 1 |  | 0 | 1 | 2 | 2 | 2 |
| 6222011310 | 2.76 | 0.99 |  | 1.23 | 0.85 | 2 | 1 | 0 |  | 0 | 1 | 2 | 2 | 2 |
| 6222011311 | 2.83 | 1.12 |  | 1.14 | 0.79 | 1 | 1 | 1 |  | 0 | 1 | 1 | 1 | 1 |
| 6222011312 | 3.11 | 1.24 |  | 1.15 | 0.64 | 2 | 1 | 0 |  | 0 | 0 | 1 | 1 | 1 |
| 6222011313 | 3.14 | 0.6 |  | 1.56 | 2.46 | 0 | 0 | 1 |  | 0 | 0 | 3 | 2 | 2 |
| 6222011314 | 3.12 | 0.86 |  | 1.63 | 0.68 | 2 | 1 | 0 |  | 0 | 0 | 1 | 1 | 1 |
| 6222011316 | 4.01 | 1.27 |  | 2.3 | 1.02 | 1 | 0 | 1 |  | 0 | 0 | 2 | 1 | 1 |
| 6222011318 | 4.35 | 1.07 |  | 2.18 | 2.04 | 1 | 1 | 1 |  | 0 | 0 | 1 | 1 | 1 |
| 6222011319 | 2.46 | 0.69 |  | 1.25 | 0.54 | 0 | 1 | 1 |  | 0 | 0 | 2 | 1 | 1 |
| 6222011320 | 4.65 | 0.97 |  | 2.6 | 1.25 | 2 | 0 | 1 |  | 0 | 0 | 1 | 1 | 1 |
| 6222011321 | 3.49 | 1.49 |  | 1.37 | 0.89 | 0 | 0 | 1 |  | 0 | 0 | 1 | 1 | 1 |
| 6222011322 | 2.83 | 0.82 |  | 1.27 | 1.63 | 1 | 0 | 0 |  | 0 | 0 | 1 | 1 | 1 |
| 6222011323 | 3.94 | 1.18 |  | 1.75 | 1.37 | 2 | 0 | 0 |  | 0 | 0 | 1 | 1 | 1 |
| 6222011324 | 3.79 | 1.04 |  | 2.21 | 0.68 | 0 | 0 | 1 |  | 0 | 0 | 2 | 1 | 2 |
| 6222011325 | 2.92 | 0.89 |  | 1.55 | 1.01 | 0 | 0 | 1 |  | 0 | 0 | 1 | 1 | 1 |
| 6222011328 | 3.82 | 1.45 |  | 1.63 | 1.17 | 1 | 0 | 1 |  | 0 | 0 | 1 | 1 | 1 |
| 6222011329 | 3.51 | 1.4 |  | 1.51 | 0.64 | 1 | 0 | 0 |  | 0 | 0 | 1 | 1 | 1 |
| 6222011331 | 5.51 | 1.14 |  | 3.04 | 1.36 | 0 | 1 | 1 |  | 0 | 1 | 1 | 1 | 1 |
| 6222011333 | 3.06 | 0.86 |  | 1.33 | 1.43 | 0 | 0 | 1 |  | 0 | 0 | 3 | 3 | 2 |
| 6222011335 | 2.36 | 0.95 |  | 0.91 | 0.52 | 0 | 0 | 0 |  | 0 | 0 | 1 | 1 | 1 |
| 6222011336 | 3.56 | 1.39 |  | 1.35 | 1.06 | 0 | 0 | 0 |  | 0 | 0 | 1 | 1 | 1 |
| 6222011337 | 3.56 | 0.73 |  | 1.82 | 2.28 | 1 | 1 | 1 |  | 1 | 1 | 2 | 2 | 1 |
| 6222011338 | 4.27 | 1.39 |  | 1.83 | 1.75 | 1 | 0 | 2 |  | 0 | 0 | 1 | 1 | 1 |
| 6222011340 | 3.17 | 0.94 |  | 1.55 | 1.55 | 0 | 0 | 1 |  | 0 | 0 | 2 | 2 | 2 |
| 6222011341 | 2.83 | 0.74 |  | 1.54 | 1.17 | 1 | 1 | 1 |  | 0 | 1 | 1 | 1 | 1 |
| 6222011342 | 3.63 | 0.88 |  | 1.83 | 1.38 | 2 | 1 | 1 |  | 0 | 0 | 2 | 1 | 2 |
| 6222011343 | 3.98 | 1.49 |  | 1.74 | 0.96 | 0 | 0 | 1 |  | 0 | 0 | 1 | 1 | 1 |
| 6222011344 | 2.91 | 0.84 |  | 1.36 | 1.07 | 0 | 0 | 1 |  | 0 | 0 | 1 | 1 | 1 |
| 6222011345 | 4.4 | 1.07 |  | 2.25 | 1.64 | 1 | 0 | 0 |  | 0 | 0 | 2 | 2 | 2 |
| 6222011346 | 5.04 | 1.24 |  | 2.78 | 1.44 | 2 | 0 | 1 |  | 0 | 0 | 2 | 1 | 1 |
| 6222011347 | 3.79 | 1.38 |  | 1.85 | 0.55 | 0 | 0 | 1 |  | 0 | 0 | 1 | 1 | 1 |
| 6222011349 | 2.38 | 0.7 |  | 1.14 | 0.96 | 1 | 1 | 2 |  | 0 | 1 | 1 | 1 | 1 |
| 6222011350 | 2.5 | 0.78 |  | 1.03 | 0.92 | 0 | 1 | 1 |  | 0 | 1 | 1 | 1 | 1 |
| 6222012101 | 3.4 | 0.68 |  | 2.01 | 1.21 | 1 | 1 | 1 |  | 0 | 1 | 3 | 2 | 2 |
| 6222012102 | 3.72 | 1.05 |  | 2.07 | 1.17 | 1 | 1 | 1 |  | 1 | 0 | 2 | 2 | 2 |
| 6222012103 | 2.81 | 0.89 |  | 1.28 | 0.76 | 1 | 1 | 2 |  | 0 | 0 | 1 | 1 | 1 |
| 6222012104 | 3.09 | 0.83 |  | 1.42 | 1.9 | 2 | 1 | 0 |  | 0 | 1 | 2 | 1 | 1 |
| 6222012105 | 3.5 | 1.07 |  | 1.82 | 0.72 | 0 | 0 | 1 |  | 0 | 0 | 2 | 2 | 2 |
| 6222012106 | 5.34 | 0.86 |  | 2.63 | 4.44 | 0 | 0 | 0 |  | 0 | 0 | 1 | 1 | 2 |
| 6222012107 | 3.4 | 0.98 |  | 1.81 | 0.79 | 0 | 0 | 1 |  | 0 | 0 | 1 | 1 | 1 |
| 6222012109 | 3.56 | 1.16 |  | 1.63 | 1.06 | 0 | 1 | 1 |  | 0 | 1 | 2 | 1 | 1 |
| 6222012110 | 2.88 | 0.87 |  | 1.39 | 0.61 | 1 | 1 | 0 |  | 0 | 1 | 1 | 1 | 1 |
| 6222012112 | 4.77 | 1 |  | 2.71 | 2.28 | 1 | 1 | 2 |  | 0 | 0 | 2 | 2 | 1 |
| 6222012113 | 3.3 | 0.74 |  | 1.72 | 1.61 | 1 | 1 | 1 |  | 0 | 0 | 2 | 2 | 2 |
| 6222012114 | 4.33 | 1.07 |  | 2.59 | 1.56 | 0 | 1 | 1 |  | 0 | 1 | 1 | 1 | 1 |
| 6222012115 | 2.71 | 0.83 |  | 1.4 | 0.6 | 1 | 0 | 2 |  | 0 | 0 | 1 | 1 | 1 |
| 6222012117 | 4.32 | 1.35 |  | 2.25 | 1.48 | 1 | 1 | 1 |  | 0 | 1 | 1 | 1 | 1 |
| 6222012118 | 3.69 | 0.79 |  | 1.71 | 3.75 | 1 | 1 | 1 |  | 0 | 1 | 2 | 1 | 2 |
| 6222012119 | 3.89 | 1.05 |  | 2.07 | 1.22 | 0 | 1 | 1 |  | 0 | 1 | 2 | 1 | 1 |
| 6222012120 | 3.32 | 0.68 |  | 1.62 | 2.01 | 1 | 0 | 1 |  | 0 | 0 | 2 | 2 | 2 |
| 6222012123 | 3.31 | 0.93 |  | 1.49 | 1.65 | 0 | 1 | 2 |  | 1 | 1 | 3 | 2 | 2 |
| 6222012124 | 4.38 | 0.92 |  | 2.35 | 1.7 | 1 | 0 | 0 |  | 0 | 0 | 1 | 1 | 1 |
| 6222012125 | 3.33 | 0.73 |  | 1.76 | 2 | 1 | 1 | 1 |  | 0 | 1 | 1 | 1 | 1 |
| 6222012127 | 2.74 | 1.19 |  | 0.98 | 0.65 | 1 | 1 | 2 |  | 1 | 0 | 1 | 1 | 1 |
| 6222012129 | 4.5 | 0.79 |  | 2.14 | 4.1 | 2 | 0 | 0 |  | 0 | 0 | 1 | 1 | 2 |
| 6222012130 | 4.35 | 1.01 |  | 2.13 | 1.9 | 1 | 0 | 0 |  | 0 | 0 | 1 | 1 | 1 |
| 6222012133 | 3.64 | 0.87 |  | 1.82 | 1.68 | 0 | 1 | 1 |  | 0 | 0 | 2 | 1 | 1 |
| 6222012134 | 4.31 | 0.88 |  | 2.15 | 2.74 | 1 | 1 | 0 |  | 1 | 1 | 3 | 3 | 1 |
| 6222012135 | 2.5 | 0.73 |  | 1.32 | 0.54 | 1 | 1 | 1 |  | 1 | 1 | 1 | 1 | 1 |
| 6222012136 | 3.16 | 0.78 |  | 1.57 | 1.18 | 0 | 1 | 2 |  | 1 | 1 | 2 | 2 | 2 |
| 6222012138 | 3.78 | 1.21 |  | 1.86 | 1 | 1 | 0 | 0 |  | 0 | 0 | 2 | 2 | 2 |
| 6222012139 | 3.17 | 1.04 |  | 1.54 | 0.9 | 0 | 0 | 1 |  | 0 | 0 | 1 | 1 | 1 |
| 6222012142 | 3 | 1.12 |  | 1.3 | 1.16 | 1 | 1 | 1 |  | 1 | 1 | 1 | 1 | 1 |
| 6222012143 | 3.16 | 1.11 |  | 1.56 | 0.79 | 1 | 0 | 0 |  | 1 | 0 | 1 | 1 | 1 |
| 6222012144 | 2.84 | 0.68 |  | 1.52 | 0.91 | 2 | 1 | 1 |  | 0 | 1 | 1 | 1 | 2 |
| 6222012145 | 3.95 | 0.73 |  | 2 | 2.44 | 1 | 0 | 0 |  | 0 | 0 | 2 | 1 | 1 |
| 6222012148 | 3.85 | 0.59 |  | 1.95 | 4.09 | 1 | 1 | 0 |  | 0 | 1 | 2 | 1 | 1 |
| 6222012149 | 4.33 | 1.11 |  | 2.2 | 2.33 | 0 | 1 | 2 |  | 0 | 1 | 1 | 1 | 1 |
| 6222012201 | 3.51 | 0.67 |  | 2.19 | 0.82 | 1 | 1 | 1 |  | 0 | 1 | 1 | 1 | 1 |
| 6222012205 | 2.54 | 0.72 |  | 1.24 | 1.02 | 0 | 0 | 1 |  | 0 | 0 | 2 | 2 | 1 |
| 6222012207 | 3.65 | 0.88 |  | 1.85 | 2.42 | 0 | 1 | 1 |  | 0 | 1 | 3 | 2 | 2 |
| 6222012208 | 3.34 | 0.84 |  | 2.22 | 0.61 | 0 | 1 | 1 |  | 0 | 0 | 1 | 1 | 1 |
| 6222012209 | 3.88 | 1.14 |  | 2.18 | 0.88 | 0 | 0 | 1 |  | 0 | 0 | 1 | 1 | 1 |
| 6222012210 | 4.29 | 0.87 |  | 2.25 | 2.29 | 2 | 0 | 0 |  | 0 | 0 | 1 | 1 | 1 |
| 6222012212 | 3.19 | 0.88 |  | 1.48 | 1.3 | 2 | 0 | 0 |  | 0 | 0 | 1 | 1 | 1 |
| 6222012213 | 2.58 | 0.74 |  | 1.31 | 1.14 | 0 | 0 | 0 |  | 0 | 0 | 1 | 1 | 1 |
| 6222012214 | 3.44 | 0.9 |  | 1.84 | 1.26 | 2 | 0 | 0 |  | 0 | 0 | 2 | 2 | 2 |
| 6222012216 | 4.43 | 0.95 |  | 2.01 | 2.96 | 0 | 0 | 1 |  | 0 | 0 | 1 | 1 | 2 |
| 6222012217 | 4.21 | 0.88 |  | 2.66 | 1.46 | 0 | 1 | 1 |  | 0 | 0 | 2 | 2 | 2 |
| 6222012218 | 2.38 | 0.88 |  | 0.94 | 0.52 | 0 | 1 | 1 |  | 0 | 0 | 1 | 1 | 1 |
| 6222012219 | 3.66 | 0.96 |  | 1.98 | 1.26 | 1 | 1 | 2 |  | 0 | 1 | 1 | 1 | 1 |
| 6222012220 | 3.63 | 1.08 |  | 1.86 | 1.42 | 1 | 0 | 0 |  | 0 | 0 | 1 | 1 | 1 |
| 6222012221 | 2.56 | 0.76 |  | 1.32 | 0.78 | 0 | 0 | 1 |  | 0 | 0 | 1 | 1 | 1 |
| 6222012222 | 2.62 | 0.86 |  | 1.24 | 1.1 | 0 | 1 | 1 |  | 0 | 0 | 1 | 1 | 1 |
| 6222012223 | 3.35 | 0.89 |  | 1.71 | 0.89 | 0 | 0 | 1 |  | 0 | 0 | 1 | 1 | 1 |
| 6222012224 | 3.97 | 1.25 |  | 2.12 | 0.91 | 1 | 0 | 0 |  | 0 | 0 | 2 | 2 | 2 |
| 6222012225 | 3.14 | 1.02 |  | 1.7 | 0.99 | 0 | 0 | 0 |  | 0 | 0 | 1 | 1 | 1 |
| 6222012227 | 2.46 | 0.65 |  | 1.27 | 1.06 | 0 | 0 | 2 |  | 0 | 0 | 1 | 1 | 3 |
| 6222012228 | 3.08 | 0.86 |  | 1.68 | 1.1 | 2 | 0 | 0 |  | 0 | 0 | 1 | 1 | 1 |
| 6222012229 | 3.53 | 1.15 |  | 1.9 | 0.78 | 0 | 0 | 1 |  | 0 | 0 | 2 | 2 | 1 |
| 6222012230 | 2.79 | 1.07 |  | 1.3 | 0.76 | 0 | 0 | 0 |  | 0 | 0 | 1 | 1 | 1 |
| 6222012231 | 3.73 | 0.9 |  | 1.96 | 2.6 | 0 | 1 | 1 |  | 0 | 1 | 2 | 2 | 2 |
| 6222012233 | 3.57 | 1.31 |  | 1.51 | 0.79 | 1 | 0 | 1 |  | 0 | 0 | 1 | 1 | 2 |
| 6222012234 | 2.72 | 0.64 |  | 1.26 | 1.94 | 0 | 1 | 1 |  | 0 | 1 | 2 | 1 | 1 |
| 6222012235 | 3.37 | 0.92 |  | 1.72 | 1.27 | 0 | 0 | 1 |  | 0 | 0 | 1 | 1 | 1 |
| 6222012237 | 2.97 | 0.7 |  | 1.47 | 1.51 | 1 | 1 | 2 |  | 0 | 0 | 1 | 1 | 1 |
| 6222012238 | 2.15 | 0.71 |  | 1.08 | 0.89 | 0 | 0 | 1 |  | 0 | 0 | 2 | 1 | 2 |
| 6222012239 | 4.76 | 1.07 |  | 2.65 | 5.06 | 0 | 0 | 1 |  | 0 | 0 | 2 | 2 | 1 |
| 6222012241 | 2.87 | 0.68 |  | 1.68 | 1.05 | 0 | 0 | 0 |  | 0 | 0 | 2 | 2 | 2 |
| 6222012242 | 4.08 | 1.24 |  | 2.15 | 0.78 | 0 | 1 | 1 |  | 0 | 0 | 2 | 2 | 1 |
| 6222012244 | 2.45 | 0.8 |  | 0.96 | 1.03 | 1 | 0 | 0 |  | 0 | 0 | 1 | 1 | 2 |
| 6222012247 | 3.66 | 0.89 |  | 1.92 | 2.22 | 0 | 1 | 1 |  | 0 | 1 | 1 | 1 | 1 |
| 6222012248 | 3.61 | 0.81 |  | 1.91 | 1.57 | 0 | 1 | 1 |  | 1 | 0 | 1 | 1 | 1 |
| 6222012249 | 3.44 | 1.11 |  | 1.69 | 0.97 | 0 | 1 | 1 |  | 0 | 0 | 2 | 1 | 1 |
| 6222012302 | 2.36 | 0.71 |  | 1.24 | 0.74 | 0 | 0 | 2 |  | 0 | 0 | 1 | 1 | 1 |
| 6222012303 | 2.93 | 0.73 |  | 1.65 | 0.78 | 1 | 0 | 1 |  | 1 | 0 | 2 | 1 | 2 |
| 6222012304 | 1.67 | 0.47 |  | 0.7 | 0.9 | 0 | 0 | 1 |  | 0 | 0 | 2 | 1 | 1 |
| 6222012305 | 4.03 | 0.8 |  | 1.87 | 2.58 | 1 | 0 | 0 |  | 0 | 0 | 1 | 1 | 1 |
| 6222012307 | 3.33 | 0.97 |  | 1.89 | 0.68 | 0 | 1 | 1 |  | 1 | 1 | 1 | 1 | 1 |
| 6222012308 | 1.96 | 0.55 |  | 0.94 | 0.76 | 0 | 1 | 1 |  | 1 | 1 | 2 | 2 | 2 |
| 6222012312 | 3.3 | 0.76 |  | 1.74 | 1.43 | 2 | 1 | 1 |  | 0 | 1 | 1 | 1 | 2 |
| 6222012314 | 3.72 | 0.89 |  | 1.99 | 1.19 | 0 | 0 | 0 |  | 0 | 0 | 1 | 1 | 1 |
| 6222012315 | 2.51 | 1.01 |  | 1.01 | 0.58 | 0 | 1 | 1 |  | 0 | 1 | 1 | 1 | 2 |
| 6222012316 | 2.44 | 0.7 |  | 1.17 | 0.69 | 0 | 1 | 1 |  | 0 | 0 | 2 | 2 | 2 |
| 6222012319 | 1.99 | 0.7 |  | 0.91 | 0.62 | 0 | 1 | 1 |  | 0 | 0 | 1 | 1 | 1 |
| 6222012320 | 5.69 | 1.44 |  | 2.8 | 3.55 | 1 | 0 | 0 |  | 0 | 0 | 2 | 1 | 1 |
| 6222012321 | 3.57 | 0.76 |  | 2.18 | 1.26 | 0 | 1 | 1 |  | 0 | 0 | 1 | 1 | 1 |
| 6222012322 | 2.92 | 1.26 |  | 1.03 | 0.64 | 2 | 1 | 1 |  | 1 | 0 | 1 | 1 | 1 |
| 6222012323 | 2.85 | 0.87 |  | 1.35 | 0.96 | 0 | 1 | 1 |  | 0 | 0 | 1 | 1 | 1 |
| 6222012327 | 3.11 | 0.66 |  | 1.83 | 1.57 | 0 | 0 | 1 |  | 0 | 0 | 1 | 1 | 2 |
| 6222012331 | 3.28 | 0.91 |  | 1.68 | 1.48 | 0 | 1 | 1 |  | 0 | 0 | 1 | 1 | 1 |
| 6222012333 | 1.98 | 0.67 |  | 0.87 | 0.55 | 0 | 0 | 1 |  | 0 | 0 | 1 | 1 | 1 |
| 6222012334 | 2.91 | 1.16 |  | 1.22 | 0.45 | 2 | 0 | 0 |  | 0 | 0 | 1 | 1 | 1 |
| 6222012335 | 2.8 | 0.76 |  | 1.42 | 1.07 | 1 | 1 | 0 |  | 1 | 1 | 1 | 1 | 1 |
| 6222012339 | 4.78 | 1.15 |  | 2.34 | 1.99 | 1 | 0 | 0 |  | 0 | 0 | 1 | 1 | 1 |
| 6222012340 | 2.95 | 0.73 |  | 1.49 | 0.95 | 1 | 1 | 0 |  | 0 | 1 | 2 | 1 | 2 |
| 6222012342 | 1.29 | 0.38 |  | 0.57 | 0.4 | 0 | 0 | 1 |  | 0 | 0 | 1 | 1 | 1 |
| 6222012343 | 0.67 | 0.2 |  | 0.29 | 0.25 | 0 | 1 | 1 |  | 0 | 0 | 1 | 1 | 2 |
| 6222012346 | 1.15 | 0.28 |  | 0.39 | 0.83 | 1 | 0 | 1 |  | 0 | 0 | 1 | 1 | 1 |
| 6222012348 | 1.68 | 0.6 |  | 0.72 | 0.94 | 0 | 0 | 1 |  | 0 | 0 | 1 | 1 | 1 |
| 6222012349 | 2.89 | 0.66 |  | 1.74 | 1.01 | 0 | 1 | 1 |  | 0 | 0 | 2 | 2 | 2 |
| 6222012350 | 2.48 | 0.98 |  | 1.07 | 0.49 | 0 | 1 | 2 |  | 0 | 0 | 1 | 1 | 1 |
| 6222013101 | 1.71 | 0.73 |  | 0.58 | 0.46 | 0 | 0 | 1 |  | 0 | 0 | 1 | 1 | 1 |
| 6222013102 | 3.28 | 0.98 |  | 1.45 | 1.63 | 1 | 0 | 2 |  | 0 | 0 | 1 | 1 | 1 |
| 6222013103 | 2.91 | 0.89 |  | 1.65 | 0.62 | 2 | 1 | 1 |  | 0 | 0 | 1 | 1 | 1 |
| 6222013104 | 2.57 | 0.84 |  | 1.12 | 0.55 | 2 | 1 | 0 |  | 0 | 0 | 1 | 1 | 1 |
| 6222013105 | 3.41 | 1.13 |  | 1.82 | 0.79 | 1 | 0 | 1 |  | 0 | 0 | 2 | 1 | 1 |
| 6222013106 | 4.46 | 1 |  | 2.38 | 2.48 | 0 | 0 | 1 |  | 0 | 0 | 1 | 1 | 1 |
| 6222013109 | 1.83 | 0.59 |  | 0.79 | 0.75 | 0 | 0 | 1 |  | 0 | 0 | 1 | 1 | 2 |
| 6222013111 | 2.74 | 0.92 |  | 1.17 | 1.25 | 2 | 1 | 1 |  | 0 | 1 | 1 | 1 | 1 |
| 6222013113 | 2.82 | 0.56 |  | 1.28 | 2.56 | 1 | 1 | 2 |  | 0 | 0 | 2 | 2 | 2 |
| 6222013116 | 4.44 | 0.85 |  | 2.29 | 2.97 | 0 | 1 | 2 |  | 0 | 1 | 3 | 2 | 3 |
| 6222013119 | 4.04 | 0.76 |  | 2.04 | 3.01 | 0 | 1 | 2 |  | 0 | 1 | 2 | 2 | 2 |
| 6222013120 | 2.03 | 0.64 |  | 0.96 | 0.85 | 1 | 1 | 2 |  | 0 | 0 | 1 | 1 | 1 |
| 6222013122 | 3.08 | 0.67 |  | 1.52 | 2.08 | 0 | 1 | 1 |  | 0 | 0 | 1 | 1 | 1 |
| 6222013123 | 3.58 | 0.5 |  | 1.88 | 3.38 | 0 | 1 | 1 |  | 0 | 0 | 2 | 2 | 3 |
| 6222013126 | 2.66 | 0.88 |  | 1.2 | 1.68 | 1 | 0 | 1 |  | 0 | 0 | 1 | 1 | 1 |
| 6222013127 | 2.64 | 0.78 |  | 1.35 | 0.74 | 1 | 1 | 1 |  | 0 | 0 | 2 | 2 | 1 |
| 6222013128 | 2.38 | 0.81 |  | 0.96 | 0.99 | 2 | 1 | 1 |  | 0 | 0 | 1 | 1 | 1 |
| 6222013129 | 2.43 | 0.73 |  | 1.28 | 0.73 | 1 | 0 | 0 |  | 0 | 0 | 2 | 1 | 1 |
| 6222013130 | 2.32 | 0.7 |  | 1.06 | 0.56 | 0 | 0 | 1 |  | 0 | 0 | 2 | 1 | 1 |
| 6222013131 | 2.24 | 0.61 |  | 1.19 | 0.71 | 0 | 0 | 1 |  | 0 | 0 | 1 | 1 | 1 |
| 6222013132 | 4.82 | 0.65 |  | 2.25 | 6.43 | 1 | 1 | 1 |  | 0 | 0 | 2 | 2 | 1 |
| 6222013133 | 1.68 | 0.48 |  | 0.81 | 0.32 | 0 | 0 | 1 |  | 0 | 0 | 2 | 2 | 1 |
| 6222013134 | 2.67 | 0.67 |  | 1.18 | 1.63 | 1 | 0 | 1 |  | 0 | 0 | 1 | 1 | 1 |
| 6222013135 | 3.38 | 0.94 |  | 1.79 | 1.24 | 0 | 1 | 0 |  | 1 | 0 | 1 | 1 | 2 |
| 6222013136 | 2.73 | 0.51 |  | 1.37 | 2.16 | 2 | 0 | 0 |  | 0 | 0 | 2 | 2 | 3 |
| 6222013137 | 2.05 | 0.56 |  | 0.98 | 0.74 | 0 | 0 | 1 |  | 0 | 0 | 1 | 1 | 1 |
| 6222013138 | 1.6 | 0.54 |  | 0.72 | 0.43 | 1 | 0 | 0 |  | 0 | 0 | 1 | 1 | 1 |
| 6222013140 | 2.28 | 0.75 |  | 0.87 | 1.09 | 2 | 1 | 0 |  | 0 | 1 | 1 | 1 | 1 |
| 6222013141 | 3.83 | 0.77 |  | 2.16 | 1.91 | 2 | 0 | 1 |  | 0 | 0 | 2 | 1 | 1 |
| 6222013143 | 1.85 | 0.4 |  | 0.95 | 0.92 | 0 | 0 | 0 |  | 0 | 0 | 2 | 2 | 2 |
| 6222013144 | 3.22 | 1.24 |  | 1.34 | 0.72 | 2 | 1 | 0 |  | 0 | 0 | 1 | 1 | 1 |
| 6222013145 | 1.47 | 0.48 |  | 0.68 | 0.61 | 0 | 0 | 1 |  | 0 | 0 | 1 | 1 | 1 |
| 6222013146 | 1.47 | 0.44 |  | 0.74 | 0.56 | 0 | 1 | 2 |  | 0 | 0 | 1 | 1 | 1 |
| 6222013147 | 1.6 | 0.6 |  | 0.68 | 0.51 | 0 | 0 | 1 |  | 0 | 0 | 1 | 1 | 1 |
| 6222013148 | 3.23 | 0.61 |  | 1.61 | 2.99 | 0 | 1 | 1 |  | 0 | 0 | 2 | 2 | 2 |
| 6222013149 | 2.47 | 0.65 |  | 1.31 | 0.84 | 0 | 0 | 2 |  | 0 | 0 | 1 | 1 | 1 |
| 6222013150 | 3.42 | 1.14 |  | 1.66 | 0.76 | 0 | 0 | 1 |  | 0 | 0 | 2 | 1 | 1 |
| 6222013201 | 2.45 | 0.89 |  | 1.14 | 0.52 | 0 | 1 | 1 |  | 1 | 1 | 1 | 1 | 1 |
| 6222013204 | 3 | 0.79 |  | 1.57 | 0.57 | 1 | 1 | 0 |  | 1 | 1 | 1 | 1 | 1 |
| 6222013205 | 3.22 | 1.06 |  | 1.56 | 1.02 | 1 | 1 | 1 |  | 0 | 0 | 1 | 1 | 1 |
| 6222013207 | 1.13 | 0.28 |  | 0.67 | 0.37 | 0 | 0 | 1 |  | 0 | 0 | 1 | 1 | 1 |
| 6222013208 | 1.84 | 0.82 |  | 0.61 | 0.49 | 0 | 0 | 1 |  | 0 | 0 | 1 | 1 | 2 |
| 6222013210 | 1.75 | 0.53 |  | 0.81 | 0.82 | 0 | 1 | 1 |  | 0 | 1 | 2 | 1 | 2 |
| 6222013211 | 3.23 | 0.6 |  | 1.53 | 2.85 | 0 | 1 | 1 |  | 0 | 1 | 2 | 2 | 2 |
| 6222013213 | 2.55 | 0.76 |  | 1.34 | 0.73 | 0 | 0 | 1 |  | 0 | 0 | 1 | 1 | 1 |
| 6222013214 | 2.64 | 0.61 |  | 1.62 | 0.56 | 0 | 1 | 1 |  | 0 | 1 | 1 | 1 | 2 |
| 6222013215 | 2.64 | 0.75 |  | 1.29 | 0.59 | 0 | 0 | 1 |  | 0 | 0 | 1 | 1 | 2 |
| 6222013216 | 2.7 | 0.88 |  | 1.27 | 0.6 | 1 | 0 | 0 |  | 0 | 0 | 1 | 1 | 1 |
| 6222013217 | 1.64 | 0.76 |  | 0.59 | 0.37 | 0 | 1 | 1 |  | 0 | 0 | 2 | 2 | 1 |
| 6222013222 | 2.7 | 0.52 |  | 1.54 | 1.78 | 0 | 1 | 1 |  | 1 | 1 | 1 | 1 | 1 |
| 6222013223 | 1.98 | 0.56 |  | 0.96 | 0.68 | 1 | 0 | 1 |  | 0 | 0 | 2 | 1 | 2 |
| 6222013224 | 2.13 | 0.41 |  | 1.05 | 0.4 | 1 | 1 | 0 |  | 0 | 0 | 1 | 1 | 1 |
| 6222013225 | 2.72 | 0.8 |  | 1.35 | 1.04 | 0 | 1 | 1 |  | 0 | 0 | 1 | 1 | 1 |
| 6222013226 | 1.99 | 0.56 |  | 1.06 | 0.69 | 1 | 1 | 1 |  | 0 | 1 | 2 | 2 | 2 |
| 6222013228 | 3.02 | 0.86 |  | 1.49 | 0.9 | 2 | 1 | 1 |  | 0 | 0 | 1 | 1 | 1 |
| 6222013231 | 3.91 | 1.1 |  | 2.28 | 0.85 | 2 | 1 | 1 |  | 0 | 1 | 1 | 1 | 1 |
| 6222013234 | 3.01 | 0.85 |  | 1.6 | 0.79 | 2 | 1 | 0 |  | 0 | 1 | 1 | 1 | 1 |
| 6222013235 | 2.88 | 0.77 |  | 1.57 | 0.73 | 2 | 1 | 1 |  | 1 | 1 | 2 | 1 | 2 |
| 6222013238 | 2.85 | 0.9 |  | 1.51 | 1.06 | 1 | 0 | 0 |  | 0 | 0 | 2 | 2 | 2 |
| 6222013240 | 3 | 1.13 |  | 1.22 | 1.17 | 0 | 1 | 2 |  | 0 | 1 | 1 | 1 | 1 |
| 6222013243 | 4 | 0.86 |  | 2.35 | 1.09 | 1 | 1 | 2 |  | 0 | 1 | 1 | 1 | 2 |
| 6222013244 | 2.78 | 0.72 |  | 1.53 | 1.47 | 0 | 1 | 2 |  | 0 | 1 | 1 | 1 | 2 |
| 6222013245 | 5.91 | 0.85 |  | 4 | 1.51 | 0 | 1 | 1 |  | 0 | 0 | 1 | 1 | 1 |
| 6222013246 | 2.25 | 0.54 |  | 0.96 | 1.63 | 0 | 0 | 1 |  | 0 | 0 | 1 | 1 | 2 |
| 6222013247 | 1.65 | 0.6 |  | 0.91 | 0.24 | 0 | 0 | 1 |  | 0 | 0 | 1 | 1 | 1 |
| 6222013248 | 1.98 | 0.79 |  | 0.81 | 0.55 | 0 | 0 | 1 |  | 0 | 0 | 1 | 1 | 1 |
| 6222013250 | 3.87 | 0.76 |  | 2.32 | 0.88 | 2 | 1 | 0 |  | 0 | 1 | 1 | 1 | 1 |
| 6222013251 | 2.94 | 1.13 |  | 1.31 | 0.46 | 1 | 1 | 1 |  | 0 | 1 | 1 | 1 | 1 |
| 6222013252 | 2.86 | 0.64 |  | 1.55 | 0.8 | 1 | 1 | 1 |  | 1 | 1 | 1 | 1 | 2 |
| 6222013253 | 2.76 | 0.55 |  | 1.39 | 1.04 | 0 | 0 | 2 |  | 0 | 0 | 1 | 1 | 3 |
| 6222013301 | 3.66 | 0.97 |  | 1.87 | 1.4 | 1 | 0 | 0 |  | 0 | 0 | 2 | 1 | 2 |
| 6222013305 | 2.12 | 0.65 |  | 1.03 | 0.5 | 0 | 0 | 1 |  | 0 | 0 | 1 | 1 | 1 |
| 6222013307 | 2.18 | 0.81 |  | 0.79 | 0.65 | 0 | 1 | 1 |  | 0 | 0 | 1 | 1 | 1 |
| 6222013313 | 2.52 | 0.94 |  | 0.98 | 0.61 | 2 | 0 | 1 |  | 0 | 0 | 1 | 1 | 1 |
| 6222013314 | 1.08 | 0.36 |  | 0.47 | 0.35 | 0 | 0 | 0 |  | 0 | 0 | 1 | 1 | 1 |
| 6222013316 | 3.14 | 0.83 |  | 1.52 | 2.16 | 1 | 1 | 0 |  | 0 | 1 | 1 | 1 | 1 |
| 6222013324 | 2.6 | 0.64 |  | 1.26 | 1.91 | 1 | 0 | 1 |  | 0 | 0 | 1 | 1 | 2 |
| 6222013326 | 2.05 | 0.53 |  | 0.9 | 1.06 | 0 | 1 | 0 |  | 0 | 0 | 1 | 1 | 1 |
| 6222013328 | 2.58 | 0.7 |  | 1.27 | 0.91 | 0 | 0 | 2 |  | 0 | 0 | 2 | 1 | 1 |
| 6222013330 | 2 | 0.84 |  | 0.69 | 0.39 | 0 | 0 | 1 |  | 0 | 0 | 1 | 1 | 1 |
| 6222013331 | 2.08 | 0.63 |  | 0.94 | 1.16 | 0 | 0 | 2 |  | 0 | 0 | 2 | 1 | 2 |
| 6222013332 | 2.61 | 0.67 |  | 1.38 | 0.91 | 1 | 1 | 1 |  | 0 | 0 | 3 | 2 | 3 |
| 6222013334 | 2.91 | 0.93 |  | 1.29 | 1.49 | 0 | 1 | 1 |  | 1 | 0 | 1 | 1 | 1 |
| 6222013336 | 3.29 | 1.04 |  | 1.68 | 1.19 | 1 | 1 | 1 |  | 0 | 1 | 1 | 1 | 1 |
| 6222013337 | 2.68 | 0.69 |  | 1.24 | 1.87 | 2 | 0 | 1 |  | 0 | 0 | 1 | 1 | 1 |
| 6222013341 | 2.65 | 0.89 |  | 1.24 | 0.65 | 0 | 0 | 1 |  | 0 | 0 | 2 | 2 | 1 |
| 6222013342 | 3.46 | 0.79 |  | 1.9 | 0.88 | 2 | 1 | 0 |  | 0 | 1 | 1 | 1 | 1 |
| 6222013343 | 3.25 | 0.88 |  | 1.75 | 0.75 | 2 | 1 | 0 |  | 1 | 1 | 1 | 1 | 1 |
| 6222013345 | 3.83 | 1.31 |  | 1.71 | 0.87 | 0 | 0 | 0 |  | 0 | 0 | 1 | 1 | 1 |
| 6222013346 | 3.4 | 0.74 |  | 1.6 | 2.1 | 1 | 1 | 0 |  | 0 | 0 | 1 | 1 | 1 |
| 6222013347 | 1.81 | 0.56 |  | 0.75 | 1.18 | 0 | 1 | 1 |  | 1 | 1 | 1 | 1 | 1 |
| 6222013349 | 2.32 | 0.65 |  | 1.22 | 0.66 | 2 | 1 | 0 |  | 0 | 0 | 2 | 2 | 1 |
| 6222014102 | 3.64 | 0.99 |  | 2.17 | 0.96 | 0 | 0 | 0 |  | 0 | 0 | 1 | 1 | 1 |
| 6222014103 | 4.09 | 1.39 |  | 1.79 | 1.55 | 0 | 0 | 0 |  | 0 | 0 | 1 | 1 | 1 |
| 6222014104 | 2.25 | 0.79 |  | 1.02 | 0.86 | 0 | 0 | 1 |  | 0 | 0 | 1 | 1 | 1 |
| 6222014105 | 4.2 | 1.17 |  | 2.34 | 1.19 | 0 | 0 | 0 |  | 0 | 0 | 2 | 1 | 2 |
| 6222014106 | 3.02 | 1.03 |  | 1.31 | 0.75 | 1 | 1 | 0 |  | 0 | 0 | 1 | 1 | 1 |
| 6222014108 | 4.09 | 1.24 |  | 2.29 | 0.91 | 0 | 1 | 1 |  | 0 | 1 | 1 | 1 | 1 |
| 6222014110 | 4.37 | 1.33 |  | 2.38 | 0.96 | 0 | 0 | 1 |  | 0 | 0 | 1 | 1 | 2 |
| 6222014111 | 3.79 | 1.17 |  | 1.93 | 0.86 | 0 | 0 | 1 |  | 0 | 0 | 1 | 1 | 1 |
| 6222014113 | 4.4 | 1.27 |  | 2.07 | 2.22 | 0 | 1 | 1 |  | 0 | 0 | 1 | 1 | 1 |
| 6222014115 | 3.68 | 1.35 |  | 1.53 | 1.03 | 1 | 1 | 1 |  | 0 | 0 | 1 | 1 | 2 |
| 6222014117 | 3.66 | 0.76 |  | 1.6 | 3.65 | 0 | 1 | 1 |  | 1 | 1 | 1 | 1 | 2 |
| 6222014119 | 3.08 | 0.8 |  | 1.49 | 1.48 | 0 | 0 | 1 |  | 0 | 0 | 1 | 1 | 1 |
| 6222014120 | 5.22 | 1.63 |  | 2.48 | 1.36 | 0 | 0 | 0 |  | 0 | 0 | 1 | 1 | 1 |
| 6222014121 | 2.93 | 0.96 |  | 1.16 | 1.34 | 0 | 1 | 1 |  | 1 | 1 | 1 | 1 | 1 |
| 6222014122 | 5.04 | 0.87 |  | 2.93 | 2.7 | 0 | 1 | 1 |  | 0 | 1 | 2 | 2 | 2 |
| 6222014123 | 2.63 | 1.01 |  | 1.06 | 0.69 | 1 | 1 | 0 |  | 0 | 1 | 1 | 1 | 1 |
| 6222014125 | 4.78 | 1.07 |  | 1.82 | 1.43 | 0 | 0 | 2 |  | 0 | 0 | 1 | 1 | 1 |
| 6222014126 | 5.2 | 0.81 |  | 1.98 | 4.91 | 1 | 1 | 1 |  | 1 | 0 | 2 | 2 | 2 |
| 6222014127 | 3.48 | 1.1 |  | 1.58 | 0.84 | 2 | 1 | 0 |  | 1 | 0 | 1 | 1 | 1 |
| 6222014129 | 3.49 | 0.85 |  | 1.32 | 3.41 | 2 | 1 | 2 |  | 0 | 0 | 3 | 2 | 2 |
| 6222014130 | 3.01 | 0.87 |  | 1.54 | 0.66 | 0 | 0 | 1 |  | 0 | 0 | 1 | 1 | 1 |
| 6222014132 | 3.2 | 1.26 |  | 1.3 | 0.55 | 0 | 1 | 2 |  | 1 | 0 | 1 | 1 | 1 |
| 6222014133 | 3.35 | 0.86 |  | 1.48 | 1.89 | 1 | 0 | 0 |  | 0 | 0 | 2 | 2 | 2 |
| 6222014134 | 3.59 | 1.16 |  | 1.79 | 1.19 | 1 | 0 | 0 |  | 0 | 0 | 1 | 1 | 1 |
| 6222014135 | 3.3 | 0.95 |  | 1.69 | 1.08 | 0 | 1 | 1 |  | 0 | 1 | 1 | 1 | 1 |
| 6222014136 | 4.49 | 0.9 |  | 2.77 | 2.1 | 0 | 1 | 0 |  | 0 | 1 | 1 | 1 | 2 |
| 6222014137 | 4.18 | 0.98 |  | 2.54 | 1.12 | 0 | 1 | 1 |  | 0 | 1 | 1 | 1 | 2 |
| 6222014139 | 6.04 | 0.68 |  | 3 | 5.29 | 0 | 1 | 1 |  | 0 | 0 | 2 | 2 | 2 |
| 6222014141 | 6.66 | 1.19 |  | 4.25 | 1.92 | 1 | 1 | 1 |  | 0 | 0 | 2 | 1 | 2 |
| 6222014142 | 4.68 | 1.48 |  | 2.03 | 1.6 | 0 | 0 | 0 |  | 0 | 0 | 2 | 2 | 2 |
| 6222014143 | 4.07 | 1.15 |  | 2.01 | 2.04 | 0 | 0 | 0 |  | 0 | 0 | 2 | 2 | 2 |
| 6222014144 | 4.14 | 0.96 |  | 1.72 | 2.84 | 0 | 1 | 1 |  | 1 | 1 | 2 | 2 | 2 |
| 6222014145 | 3.11 | 0.96 |  | 1.32 | 1.14 | 0 | 1 | 1 |  | 1 | 1 | 1 | 1 | 1 |
| 6222014146 | 3.52 | 0.93 |  | 2.02 | 0.84 | 0 | 1 | 1 |  | 0 | 1 | 2 | 1 | 2 |
| 6222014147 | 4.08 | 1.18 |  | 2.2 | 0.84 | 1 | 0 | 0 |  | 0 | 0 | 1 | 1 | 1 |
| 6222014149 | 2.18 | 0.68 |  | 1.06 | 0.61 | 0 | 1 | 2 |  | 0 | 0 | 1 | 1 | 1 |
| 6222014150 | 5.18 | 1.1 |  | 2.11 | 3.6 | 0 | 1 | 1 |  | 0 | 1 | 3 | 2 | 2 |
| 6222014202 | 2.49 | 0.68 |  | 1.14 | 0.71 | 0 | 0 | 0 |  | 0 | 0 | 1 | 1 | 1 |
| 6222014203 | 3.63 | 0.6 |  | 1.98 | 3.06 | 0 | 1 | 2 |  | 0 | 1 | 1 | 1 | 2 |
| 6222014204 | 2.76 | 0.71 |  | 1.3 | 1.58 | 0 | 1 | 2 |  | 0 | 1 | 2 | 2 | 3 |
| 6222014205 | 2.93 | 0.7 |  | 1.69 | 0.83 | 0 | 1 | 1 |  | 0 | 1 | 1 | 1 | 2 |
| 6222014206 | 2.96 | 0.8 |  | 1.55 | 1.41 | 1 | 0 | 0 |  | 0 | 0 | 1 | 1 | 1 |
| 6222014207 | 2.87 | 1.13 |  | 1.22 | 1 | 0 | 0 | 0 |  | 0 | 0 | 1 | 1 | 1 |
| 6222014208 | 3.83 | 1 |  | 1.43 | 5.31 | 0 | 1 | 2 |  | 0 | 0 | 2 | 1 | 2 |
| 6222014209 | 1.94 | 0.77 |  | 0.61 | 1.55 | 0 | 1 | 1 |  | 0 | 1 | 1 | 1 | 3 |
| 6222014210 | 2.2 | 0.67 |  | 0.97 | 0.96 | 0 | 0 | 2 |  | 0 | 0 | 1 | 1 | 1 |
| 6222014217 | 3.28 | 0.87 |  | 1.9 | 0.68 | 0 | 0 | 0 |  | 0 | 0 | 2 | 1 | 2 |
| 6222014220 | 3.02 | 0.47 |  | 1.44 | 2.75 | 0 | 1 | 2 |  | 1 | 1 | 2 | 1 | 2 |
| 6222014222 | 1.52 | 0.46 |  | 0.7 | 0.91 | 1 | 0 | 1 |  | 0 | 0 | 3 | 2 | 3 |
| 6222014223 | 3.53 | 0.93 |  | 2.04 | 0.87 | 0 | 0 | 2 |  | 0 | 0 | 1 | 1 | 1 |
| 6222014228 | 3.35 | 0.72 |  | 2.11 | 0.88 | 0 | 1 | 1 |  | 0 | 0 | 2 | 2 | 2 |
| 6222014229 | 3.22 | 0.64 |  | 2.08 | 0.88 | 0 | 1 | 2 |  | 0 | 0 | 2 | 2 | 2 |
| 6222014230 | 1.82 | 0.49 |  | 0.91 | 0.72 | 0 | 0 | 2 |  | 0 | 0 | 1 | 1 | 1 |
| 6222014231 | 2.33 | 0.81 |  | 0.94 | 1.37 | 0 | 0 | 1 |  | 0 | 0 | 1 | 1 | 2 |
| 6222014232 | 2.4 | 0.61 |  | 1.11 | 0.95 | 0 | 0 | 1 |  | 0 | 0 | 1 | 1 | 1 |
| 6222014233 | 3.41 | 0.66 |  | 1.94 | 1.62 | 0 | 0 | 1 |  | 0 | 0 | 1 | 1 | 1 |
| 6222014234 | 2.97 | 0.88 |  | 1.46 | 0.94 | 1 | 0 | 1 |  | 0 | 0 | 1 | 1 | 1 |
| 6222014235 | 3.8 | 0.76 |  | 1.92 | 3.75 | 0 | 1 | 2 |  | 0 | 0 | 1 | 1 | 1 |
| 6222014236 | 4.09 | 0.99 |  | 2.05 | 1.71 | 1 | 1 | 2 |  | 0 | 1 | 1 | 1 | 1 |
| 6222014238 | 2.25 | 0.57 |  | 1.09 | 0.94 | 0 | 0 | 0 |  | 0 | 0 | 2 | 2 | 1 |
| 6222014239 | 2.88 | 0.6 |  | 1.64 | 0.99 | 1 | 0 | 0 |  | 0 | 0 | 2 | 1 | 2 |
| 6222014240 | 2.31 | 0.63 |  | 1.12 | 0.8 | 0 | 0 | 1 |  | 0 | 0 | 1 | 1 | 2 |
| 6222014241 | 2.71 | 0.76 |  | 1.45 | 0.66 | 0 | 1 | 2 |  | 1 | 0 | 1 | 1 | 1 |
| 6222014242 | 3.87 | 0.81 |  | 2.01 | 2.33 | 2 | 1 | 0 |  | 0 | 0 | 1 | 1 | 2 |
| 6222014244 | 3.2 | 1.1 |  | 1.4 | 0.62 | 0 | 1 | 1 |  | 1 | 1 | 1 | 1 | 1 |
| 6222014245 | 3.14 | 0.78 |  | 1.4 | 1.49 | 0 | 1 | 2 |  | 0 | 0 | 1 | 1 | 2 |
| 6222014246 | 3.03 | 0.51 |  | 1.58 | 3.89 | 0 | 1 | 1 |  | 0 | 0 | 2 | 1 | 2 |
| 6222014247 | 2.82 | 0.59 |  | 1.31 | 1.35 | 0 | 0 | 0 |  | 1 | 0 | 1 | 1 | 2 |
| 6222014248 | 3.08 | 0.59 |  | 1.8 | 1.54 | 0 | 0 | 0 |  | 0 | 0 | 2 | 2 | 2 |
| 6222014249 | 2.28 | 0.8 |  | 3.18 | 1.23 | 0 | 0 | 1 |  | 0 | 0 | 2 | 2 | 2 |
| 6222014250 | 2.07 | 0.54 |  | 0.87 | 1.07 | 0 | 0 | 1 |  | 0 | 0 | 1 | 1 | 1 |
| 6222014301 | 4.78 | 1.22 |  | 2.58 | 1.5 | 0 | 1 | 1 |  | 1 | 1 | 1 | 1 | 1 |
| 6222014302 | 4.45 | 0.89 |  | 2.66 | 2.95 | 0 | 1 | 2 |  | 1 | 0 | 3 | 2 | 2 |
| 6222014303 | 3.54 | 1.01 |  | 1.87 | 0.89 | 0 | 1 | 2 |  | 0 | 1 | 2 | 2 | 2 |
| 6222014305 | 2.4 | 0.78 |  | 1.01 | 3.11 | 0 | 0 | 2 |  | 0 | 0 | 2 | 2 | 2 |
| 6222014307 | 3.49 | 1.03 |  | 1.59 | 1.89 | 0 | 1 | 0 |  | 0 | 1 | 1 | 1 | 1 |
| 6222014309 | 4.36 | 0.96 |  | 1.89 | 3.68 | 0 | 1 | 1 |  | 0 | 0 | 2 | 1 | 2 |
| 6222014311 | 4.48 | 1.05 |  | 2.56 | 1.12 | 0 | 1 | 0 |  | 0 | 1 | 1 | 1 | 1 |
| 6222014312 | 4.02 | 0.82 |  | 2.11 | 2.93 | 0 | 1 | 1 |  | 0 | 1 | 2 | 2 | 2 |
| 6222014313 | 3.37 | 1.01 |  | 1.69 | 1.04 | 0 | 1 | 1 |  | 0 | 1 | 1 | 1 | 1 |
| 6222014316 | 3.61 | 0.85 |  | 1.93 | 1.26 | 2 | 1 | 0 |  | 0 | 1 | 1 | 1 | 1 |
| 6222014319 | 2.77 | 0.67 |  | 1.27 | 1.41 | 1 | 1 | 1 |  | 0 | 1 | 1 | 1 | 1 |
| 6222014320 | 3.11 | 0.97 |  | 1.34 | 1.16 | 0 | 1 | 2 |  | 1 | 1 | 3 | 3 | 2 |
| 6222014322 | 3.76 | 1.16 |  | 1.86 | 0.88 | 0 | 0 | 0 |  | 0 | 0 | 1 | 1 | 1 |
| 6222014323 | 3.06 | 1.13 |  | 1.19 | 0.62 | 0 | 0 | 1 |  | 0 | 0 | 1 | 1 | 2 |
| 6222014324 | 3.85 | 0.86 |  | 2.08 | 1.74 | 0 | 0 | 1 |  | 0 | 0 | 1 | 1 | 2 |
| 6222014325 | 3.3 | 1.28 |  | 1.1 | 1.46 | 1 | 1 | 0 |  | 0 | 0 | 1 | 1 | 1 |
| 6222014326 | 3.77 | 0.98 |  | 1.76 | 1.52 | 0 | 1 | 2 |  | 1 | 1 | 1 | 1 | 1 |
| 6222014329 | 3.04 | 0.86 |  | 1.46 | 1.09 | 0 | 0 | 1 |  | 0 | 0 | 3 | 2 | 2 |
| 6222014330 | 3.44 | 1.05 |  | 1.75 | 0.83 | 0 | 0 | 1 |  | 0 | 0 | 2 | 1 | 1 |
| 6222014331 | 6.22 | 1.24 |  | 3.56 | 1.95 | 0 | 0 | 1 |  | 0 | 0 | 1 | 1 | 1 |
| 6222014332 | 3.75 | 0.86 |  | 1.94 | 2.28 | 0 | 1 | 2 |  | 0 | 0 | 1 | 1 | 2 |
| 6222014334 | 2.94 | 1.22 |  | 1.1 | 0.9 | 0 | 1 | 0 |  | 0 | 0 | 1 | 1 | 1 |
| 6222014336 | 3.6 | 1.13 |  | 1.55 | 1.27 | 0 | 1 | 1 |  | 0 | 0 | 1 | 1 | 2 |
| 6222014337 | 3.46 | 1.3 |  | 1.56 | 0.72 | 0 | 0 | 0 |  | 0 | 0 | 1 | 1 | 2 |
| 6222014338 | 3.46 | 1.02 |  | 1.72 | 1.15 | 0 | 0 | 1 |  | 0 | 0 | 1 | 1 | 1 |
| 6222014339 | 3.14 | 0.92 |  | 1.69 | 1.04 | 0 | 1 | 2 |  | 0 | 0 | 1 | 1 | 2 |
| 6222014340 | 4.17 | 1.29 |  | 2.13 | 0.76 | 0 | 0 | 2 |  | 0 | 0 | 1 | 1 | 1 |
| 6222014342 | 1.98 | 0.79 |  | 0.78 | 0.69 | 1 | 1 | 2 |  | 0 | 1 | 1 | 1 | 1 |
| 6222014343 | 2.86 | 0.91 |  | 1.14 | 1.07 | 1 | 1 | 1 |  | 0 | 0 | 1 | 1 | 1 |
| 6222014344 | 3.28 | 1.05 |  | 1.7 | 0.76 | 0 | 1 | 2 |  | 0 | 0 | 1 | 1 | 1 |
| 6222014345 | 3.52 | 0.85 |  | 1.74 | 2.02 | 0 | 0 | 1 |  | 0 | 0 | 2 | 2 | 1 |
| 6222014346 | 3.34 | 1.01 |  | 1.54 | 1.28 | 0 | 0 | 0 |  | 0 | 0 | 1 | 1 | 1 |
| 6222014347 | 4.34 | 1.18 |  | 2.14 | 1.95 | 0 | 0 | 1 |  | 0 | 0 | 1 | 1 | 1 |
| 6222014348 | 4.85 | 1.3 |  | 2.84 | 1.43 | 1 | 0 | 1 |  | 0 | 0 | 2 | 1 | 1 |
| 6222014349 | 3.51 | 1.39 |  | 1.36 | 0.92 | 0 | 1 | 1 |  | 0 | 0 | 1 | 1 | 1 |
| 6222014350 | 2.52 | 0.74 |  | 1.51 | 0.69 | 0 | 1 | 1 |  | 0 | 1 | 1 | 1 | 2 |
| 6230211101 | 3.31 | 1.25 |  | 1.72 | 1.05 | 1 | 0 | 0 |  | 0 | 0 | 1 | 1 | 1 |
| 6230211103 | 3.09 | 0.95 |  | 1.76 | 0.62 | 0 | 1 | 1 |  | 0 | 1 | 1 | 1 | 1 |
| 6230211104 | 3.31 | 1.07 |  | 1.91 | 0.56 | 2 | 1 | 1 |  | 0 | 0 | 1 | 1 | 1 |
| 6230211105 | 3.08 | 0.82 |  | 1.99 | 1.11 | 1 | 0 | 0 |  | 0 | 0 | 2 | 2 | 2 |
| 6230211106 | 2.11 | 0.58 |  | 1.33 | 0.54 | 0 | 1 | 0 |  | 0 | 0 | 1 | 1 | 1 |
| 6230211108 | 2.14 | 0.6 |  | 1.22 | 0.92 | 0 | 0 | 1 |  | 0 | 0 | 1 | 1 | 1 |
| 6230211109 | 2.06 | 0.57 |  | 0.83 | 2.16 | 0 | 1 | 0 |  | 0 | 1 | 1 | 1 | 2 |
| 6230211110 | 1.74 | 0.63 |  | 0.7 | 0.95 | 0 | 0 | 1 |  | 0 | 0 | 1 | 1 | 1 |
| 6230211112 | 3.4 | 0.82 |  | 2.24 | 1.09 | 0 | 0 | 0 |  | 0 | 0 | 1 | 1 | 1 |
| 6230211114 | 2.04 | 0.64 |  | 1.3 | 0.48 | 0 | 0 | 0 |  | 0 | 0 | 1 | 1 | 1 |
| 6230211116 | 1.8 | 0.57 |  | 0.78 | 0.94 | 1 | 0 | 0 |  | 0 | 0 | 1 | 1 | 1 |
| 6230211118 | 2.9 | 1 |  | 1.43 | 0.69 | 0 | 0 | 0 |  | 0 | 0 | 1 | 1 | 1 |
| 6230211119 | 2.24 | 0.91 |  | 1.05 | 0.72 | 1 | 1 | 1 |  | 0 | 1 | 1 | 1 | 1 |
| 6230211120 | 2.2 | 0.71 |  | 1.15 | 0.53 | 0 | 1 | 0 |  | 0 | 1 | 2 | 1 | 1 |
| 6230211121 | 3.6 | 0.8 |  | 2.32 | 1.01 | 0 | 1 | 1 |  | 0 | 0 | 1 | 1 | 1 |
| 6230211122 | 2.5 | 0.82 |  | 1.36 | 0.91 | 0 | 0 | 0 |  | 0 | 0 | 1 | 1 | 1 |
| 6230211126 | 2.75 | 0.64 |  | 1.69 | 1.12 | 2 | 1 | 1 |  | 0 | 1 | 1 | 1 | 1 |
| 6230211128 | 2.97 | 0.61 |  | 1.86 | 0.77 | 1 | 0 | 0 |  | 0 | 0 | 1 | 1 | 1 |
| 6230211130 | 2.39 | 0.86 |  | 1.19 | 0.67 | 1 | 1 | 1 |  | 0 | 1 | 1 | 1 | 1 |
| 6230211131 | 2.53 | 0.92 |  | 1.27 | 0.8 | 0 | 0 | 1 |  | 0 | 0 | 1 | 1 | 1 |
| 6230211132 | 2.89 | 0.88 |  | 1.77 | 0.71 | 1 | 0 | 0 |  | 0 | 0 | 1 | 1 | 2 |
| 6230211133 | 2.41 | 0.9 |  | 1.18 | 0.77 | 0 | 0 | 1 |  | 0 | 0 | 1 | 1 | 1 |
| 6230211135 | 3.64 | 0.91 |  | 2.43 | 0.92 | 1 | 0 | 0 |  | 0 | 0 | 1 | 1 | 1 |
| 6230211137 | 2.62 | 0.78 |  | 1.4 | 1.03 | 0 | 1 | 1 |  | 0 | 0 | 1 | 1 | 2 |
| 6230211138 | 2.37 | 0.61 |  | 1.41 | 1.05 | 0 | 0 | 0 |  | 0 | 0 | 1 | 1 | 1 |
| 6230211139 | 2.26 | 0.55 |  | 1.2 | 1.47 | 0 | 0 | 0 |  | 0 | 0 | 1 | 1 | 1 |
| 6230211140 | 2.24 | 0.77 |  | 1.4 | 0.49 | 0 | 0 | 1 |  | 0 | 0 | 1 | 1 | 1 |
| 6230211141 | 2.96 | 0.98 |  | 1.63 | 0.75 | 0 | 0 | 1 |  | 0 | 0 | 1 | 1 | 2 |
| 6230211143 | 2.83 | 0.72 |  | 1.47 | 1.34 | 1 | 0 | 0 |  | 0 | 0 | 1 | 1 | 1 |
| 6230211148 | 1.89 | 0.69 |  | 0.84 | 0.92 | 0 | 1 | 1 |  | 1 | 1 | 2 | 2 | 2 |
| 6230211150 | 3.03 | 1.05 |  | 1.54 | 0.86 | 0 | 0 | 0 |  | 0 | 0 | 1 | 1 | 1 |
| 6230211151 | 1.61 | 0.69 |  | 0.79 | 0.43 | 2 | 1 | 0 |  | 0 | 1 | 1 | 1 | 1 |
| 6230211201 | 4.47 | 1.32 |  | 2.33 | 2.02 | 0 | 0 | 0 |  | 0 | 0 | 1 | 1 | 1 |
| 6230211203 | 3.16 | 0.74 |  | 1.8 | 1.42 | 1 | 1 | 1 |  | 0 | 0 | 1 | 1 | 1 |
| 6230211205 | 2.87 | 0.69 |  | 1.64 | 1.56 | 0 | 0 | 1 |  | 0 | 0 | 1 | 1 | 1 |
| 6230211209 | 2.86 | 1.01 |  | 1.49 | 0.65 | 0 | 0 | 0 |  | 0 | 0 | 1 | 1 | 1 |
| 6230211212 | 2.94 | 1.13 |  | 1.17 | 1.24 | 0 | 1 | 1 |  | 0 | 1 | 1 | 1 | 1 |
| 6230211215 | 2.8 | 1.12 |  | 1.25 | 0.82 | 0 | 0 | 0 |  | 0 | 0 | 2 | 2 | 1 |
| 6230211216 | 3.1 | 1.05 |  | 1.83 | 0.66 | 0 | 0 | 0 |  | 0 | 0 | 2 | 1 | 1 |
| 6230211217 | 2.55 | 0.77 |  | 1.48 | 0.81 | 0 | 0 | 0 |  | 0 | 0 | 1 | 1 | 1 |
| 6230211218 | 2.76 | 1.05 |  | 1.23 | 1.05 | 0 | 1 | 1 |  | 0 | 1 | 1 | 1 | 1 |
| 6230211220 | 2.93 | 1.19 |  | 1.4 | 0.54 | 1 | 1 | 0 |  | 0 | 1 | 1 | 1 | 1 |
| 6230211222 | 2.74 | 0.98 |  | 1.23 | 1.08 | 0 | 0 | 0 |  | 0 | 0 | 1 | 1 | 1 |
| 6230211223 | 2.65 | 0.93 |  | 1.55 | 0.66 | 0 | 0 | 1 |  | 0 | 0 | 1 | 1 | 1 |
| 6230211227 | 2.84 | 1.25 |  | 0.91 | 1.2 | 0 | 0 | 0 |  | 0 | 0 | 1 | 1 | 1 |
| 6230211228 | 3.38 | 1.27 |  | 1.46 | 0.95 | 1 | 1 | 1 |  | 0 | 1 | 1 | 1 | 1 |
| 6230211233 | 2.7 | 0.61 |  | 1.71 | 1 | 0 | 1 | 0 |  | 0 | 0 | 1 | 1 | 1 |
| 6230211236 | 3.1 | 1.08 |  | 1.48 | 0.58 | 1 | 1 | 1 |  | 0 | 0 | 1 | 1 | 1 |
| 6230211237 | 1.85 | 0.99 |  | 0.48 | 0.4 | 0 | 1 | 1 |  | 0 | 1 | 1 | 1 | 1 |
| 6230211239 | 3.43 | 1.04 |  | 1.69 | 2.4 | 0 | 1 | 0 |  | 0 | 1 | 1 | 1 | 1 |
| 6230211240 | 3.1 | 1.27 |  | 1.37 | 0.8 | 0 | 0 | 0 |  | 0 | 0 | 1 | 1 | 1 |
| 6230211243 | 3.51 | 1.3 |  | 1.64 | 0.87 | 0 | 0 | 0 |  | 0 | 0 | 2 | 2 | 1 |
| 6230211244 | 3.11 | 0.87 |  | 1.88 | 1.11 | 1 | 1 | 1 |  | 0 | 1 | 1 | 1 | 1 |
| 6230211245 | 2.76 | 0.94 |  | 1.35 | 1.18 | 1 | 0 | 0 |  | 0 | 0 | 2 | 2 | 2 |
| 6230211246 | 3.84 | 1.18 |  | 2.01 | 1.45 | 0 | 1 | 0 |  | 0 | 0 | 1 | 1 | 2 |
| 6230211248 | 5.76 | 1.6 |  | 2.72 | 4 | 0 | 0 | 0 |  | 0 | 0 | 1 | 1 | 2 |
| 6230211250 | 2.67 | 1.11 |  | 1.14 | 0.96 | 0 | 1 | 1 |  | 0 | 1 | 1 | 1 | 1 |
| 6230211301 | 5.32 | 1.31 |  | 3.47 | 1.35 | 0 | 1 | 1 |  | 0 | 0 | 1 | 1 | 2 |
| 6230211302 | 3.03 | 0.64 |  | 1.75 | 1.8 | 0 | 0 | 0 |  | 0 | 0 | 2 | 1 | 1 |
| 6230211304 | 2.3 | 0.62 |  | 1.43 | 0.66 | 0 | 0 | 0 |  | 0 | 0 | 1 | 1 | 1 |
| 6230211305 | 2.67 | 0.83 |  | 1.39 | 0.87 | 0 | 0 | 2 |  | 0 | 0 | 1 | 1 | 1 |
| 6230211306 | 2.23 | 0.58 |  | 1.32 | 0.94 | 0 | 0 | 0 |  | 0 | 0 | 1 | 1 | 1 |
| 6230211307 | 2.25 | 0.64 |  | 1.31 | 1.07 | 0 | 0 | 0 |  | 0 | 0 | 1 | 1 | 1 |
| 6230211310 | 1.86 | 0.58 |  | 1.07 | 0.53 | 0 | 1 | 0 |  | 0 | 1 | 1 | 1 | 1 |
| 6230211313 | 3.42 | 0.76 |  | 1.95 | 1.46 | 2 | 0 | 0 |  | 0 | 0 | 1 | 1 | 1 |
| 6230211317 | 2.36 | 0.66 |  | 1.25 | 1.04 | 1 | 0 | 0 |  | 0 | 0 | 1 | 1 | 1 |
| 6230211318 | 4.55 | 1.12 |  | 2.95 | 1.72 | 1 | 1 | 1 |  | 0 | 1 | 1 | 1 | 1 |
| 6230211322 | 2.93 | 0.78 |  | 1.42 | 1.96 | 0 | 0 | 0 |  | 0 | 0 | 1 | 1 | 1 |
| 6230211323 | 2.28 | 0.52 |  | 1.06 | 2.2 | 0 | 0 | 0 |  | 0 | 0 | 2 | 2 | 2 |
| 6230211326 | 2.87 | 0.77 |  | 1.77 | 0.9 | 0 | 0 | 0 |  | 0 | 0 | 1 | 1 | 1 |
| 6230211327 | 2.84 | 1.1 |  | 1.22 | 1.74 | 0 | 1 | 1 |  | 1 | 1 | 1 | 1 | 1 |
| 6230211328 | 2.57 | 0.76 |  | 1.56 | 0.89 | 0 | 0 | 1 |  | 0 | 0 | 1 | 1 | 1 |
| 6230211330 | 4.91 | 1.02 |  | 3.02 | 2.31 | 2 | 1 | 0 |  | 0 | 0 | 1 | 1 | 1 |
| 6230211331 | 2.39 | 0.89 |  | 1.19 | 1.06 | 1 | 0 | 0 |  | 0 | 0 | 1 | 1 | 1 |
| 6230211333 | 3.35 | 0.68 |  | 2.04 | 2.15 | 1 | 1 | 1 |  | 0 | 0 | 2 | 2 | 1 |
| 6230211334 | 4.81 | 0.64 |  | 2.21 | 6.17 | 2 | 1 | 0 |  | 0 | 0 | 1 | 1 | 1 |
| 6230211337 | 3.47 | 0.79 |  | 2.22 | 1.56 | 0 | 1 | 2 |  | 0 | 1 | 2 | 2 | 3 |
| 6230211338 | 3.26 | 0.73 |  | 2.07 | 1.46 | 2 | 1 | 0 |  | 0 | 1 | 1 | 1 | 1 |
| 6230211339 | 2.41 | 1.23 |  | 0.8 | 0.89 | 1 | 1 | 1 |  | 1 | 0 | 1 | 1 | 2 |
| 6230211341 | 2.3 | 0.95 |  | 0.85 | 0.73 | 0 | 1 | 1 |  | 0 | 1 | 1 | 1 | 1 |
| 6230211342 | 2.11 | 0.67 |  | 1.11 | 0.69 | 2 | 1 | 0 |  | 0 | 1 | 1 | 1 | 1 |
| 6230211345 | 2.27 | 0.75 |  | 1.22 | 0.58 | 0 | 1 | 0 |  | 0 | 0 | 1 | 1 | 1 |
| 6230211346 | 2.09 | 0.9 |  | 0.94 | 0.51 | 0 | 1 | 0 |  | 0 | 1 | 1 | 1 | 1 |
| 6230211348 | 2.79 | 0.71 |  | 1.64 | 1.32 | 1 | 1 | 1 |  | 0 | 1 | 1 | 1 | 1 |
| 6230211350 | 2.64 | 1.11 |  | 1.13 | 0.71 | 1 | 0 | 0 |  | 0 | 0 | 1 | 1 | 1 |
| 6230212101 | 3.02 | 1.1 |  | 1.36 | 1.04 | 2 | 1 | 0 |  | 0 | 0 | 1 | 1 | 1 |
| 6230212104 | 4.49 | 1.59 |  | 2.28 | 1.3 | 1 | 0 | 0 |  | 0 | 0 | 2 | 2 | 2 |
| 6230212105 | 2.7 | 0.77 |  | 1.41 | 1.2 | 2 | 0 | 0 |  | 0 | 0 | 1 | 1 | 2 |
| 6230212106 | 4.33 | 1.53 |  | 2.36 | 1.15 | 0 | 1 | 1 |  | 1 | 0 | 2 | 2 | 1 |
| 6230212108 | 3.47 | 1.07 |  | 1.83 | 1.29 | 1 | 1 | 0 |  | 0 | 0 | 2 | 2 | 1 |
| 6230212109 | 3.59 | 0.83 |  | 1.44 | 2.71 | 0 | 1 | 1 |  | 0 | 1 | 2 | 2 | 2 |
| 6230212110 | 3.21 | 0.78 |  | 2.11 | 1.12 | 0 | 1 | 1 |  | 0 | 0 | 1 | 1 | 2 |
| 6230212111 | 3.49 | 0.99 |  | 1.94 | 1.29 | 1 | 0 | 0 |  | 0 | 0 | 2 | 1 | 2 |
| 6230212112 | 2.3 | 0.71 |  | 1.14 | 1.13 | 2 | 0 | 0 |  | 0 | 0 | 1 | 1 | 3 |
| 6230212113 | 5.03 | 1.45 |  | 3.11 | 1.17 | 0 | 0 | 0 |  | 0 | 0 | 1 | 1 | 1 |
| 6230212115 | 3.69 | 0.79 |  | 2.5 | 1.22 | 2 | 1 | 1 |  | 0 | 1 | 1 | 1 | 1 |
| 6230212116 | 3.05 | 0.83 |  | 1.84 | 1.26 | 0 | 0 | 0 |  | 0 | 0 | 2 | 1 | 2 |
| 6230212118 | 2.45 | 0.77 |  | 1.34 | 0.95 | 1 | 0 | 0 |  | 0 | 0 | 1 | 1 | 1 |
| 6230212119 | 2.17 | 0.58 |  | 0.9 | 1.36 | 1 | 0 | 0 |  | 0 | 0 | 2 | 1 | 3 |
| 6230212120 | 2.94 | 0.89 |  | 1.56 | 1.16 | 0 | 0 | 0 |  | 0 | 0 | 1 | 1 | 1 |
| 6230212123 | 5.25 | 1.07 |  | 4.2 | 0.94 | 1 | 1 | 0 |  | 0 | 1 | 1 | 1 | 1 |
| 6230212124 | 2.64 | 0.73 |  | 1.44 | 1.12 | 2 | 1 | 0 |  | 0 | 1 | 1 | 1 | 1 |
| 6230212126 | 3.24 | 1.11 |  | 1.4 | 1.5 | 2 | 0 | 0 |  | 0 | 0 | 1 | 1 | 1 |
| 6230212128 | 3.74 | 0.77 |  | 2.15 | 1.93 | 0 | 1 | 1 |  | 0 | 0 | 2 | 2 | 3 |
| 6230212130 | 4.65 | 0.9 |  | 2.07 | 3.17 | 1 | 1 | 2 |  | 0 | 0 | 2 | 2 | 2 |
| 6230212131 | 3.22 | 0.93 |  | 1.8 | 1.15 | 1 | 1 | 1 |  | 0 | 1 | 1 | 1 | 1 |
| 6230212135 | 2.46 | 0.85 |  | 1.31 | 0.95 | 1 | 0 | 0 |  | 0 | 0 | 2 | 1 | 3 |
| 6230212136 | 4.39 | 1.64 |  | 2.22 | 1.08 | 1 | 1 | 1 |  | 0 | 0 | 1 | 1 | 1 |
| 6230212137 | 3.67 | 1.3 |  | 1.9 | 0.95 | 0 | 0 | 2 |  | 0 | 0 | 1 | 1 | 1 |
| 6230212138 | 3.48 | 1.13 |  | 1.91 | 1.16 | 0 | 0 | 0 |  | 0 | 0 | 1 | 1 | 1 |
| 6230212139 | 3.02 | 0.74 |  | 1.83 | 1.36 | 1 | 0 | 2 |  | 0 | 0 | 2 | 2 | 1 |
| 6230212140 | 2.77 | 0.95 |  | 1.25 | 0.97 | 0 | 1 | 0 |  | 0 | 0 | 1 | 1 | 1 |
| 6230212144 | 2.71 | 0.95 |  | 1.26 | 1.16 | 1 | 0 | 0 |  | 0 | 0 | 1 | 1 | 1 |
| 6230212145 | 2.56 | 0.95 |  | 1.19 | 0.56 | 0 | 0 | 0 |  | 0 | 0 | 1 | 1 | 1 |
| 6230212150 | 4.38 | 1.23 |  | 2.63 | 1.09 | 2 | 0 | 0 |  | 0 | 0 | 2 | 2 | 1 |
| 6230212151 | 4.78 | 1.01 |  | 3.3 | 1.31 | 1 | 0 | 1 |  | 0 | 0 | 1 | 1 | 2 |
| 6230212152 | 2.02 | 0.44 |  | 1.17 | 1.26 | 1 | 0 | 0 |  | 0 | 0 | 1 | 1 | 1 |
| 6230212201 | 4.48 | 0.87 |  | 1.79 | 5.49 | 0 | 0 | 0 |  | 0 | 0 | 1 | 1 | 1 |
| 6230212202 | 3.22 | 0.81 |  | 1.95 | 1.51 | 0 | 0 | 0 |  | 0 | 0 | 2 | 2 | 3 |
| 6230212203 | 3.42 | 1.39 |  | 1.43 | 1.04 | 2 | 0 | 0 |  | 0 | 0 | 1 | 1 | 1 |
| 6230212205 | 2.87 | 1.05 |  | 1.52 | 0.8 | 1 | 1 | 0 |  | 0 | 1 | 1 | 1 | 1 |
| 6230212206 | 3.76 | 0.77 |  | 1.6 | 5.71 | 0 | 1 | 0 |  | 0 | 0 | 2 | 1 | 1 |
| 6230212207 | 3.77 | 1.2 |  | 2.28 | 1.18 | 0 | 1 | 0 |  | 0 | 1 | 2 | 2 | 2 |
| 6230212208 | 2.99 | 0.8 |  | 1.53 | 2.16 | 1 | 0 | 0 |  | 0 | 0 | 1 | 1 | 1 |
| 6230212209 | 3.5 | 0.74 |  | 2.02 | 2.03 | 0 | 1 | 0 |  | 0 | 1 | 1 | 1 | 1 |
| 6230212210 | 2.76 | 0.92 |  | 1.32 | 0.65 | 0 | 0 | 1 |  | 0 | 0 | 1 | 1 | 1 |
| 6230212211 | 3.09 | 1.25 |  | 1.51 | 0.97 | 0 | 0 | 1 |  | 0 | 0 | 1 | 1 | 1 |
| 6230212213 | 3.2 | 0.8 |  | 1.64 | 1.45 | 2 | 1 | 0 |  | 0 | 1 | 3 | 2 | 3 |
| 6230212215 | 4.62 | 0.99 |  | 2.97 | 2.7 | 0 | 1 | 0 |  | 0 | 0 | 1 | 1 | 1 |
| 6230212216 | 3.56 | 1.11 |  | 1.97 | 1.03 | 0 | 0 | 0 |  | 0 | 0 | 1 | 1 | 1 |
| 6230212217 | 3 | 0.98 |  | 1.66 | 1.2 | 0 | 0 | 0 |  | 0 | 0 | 1 | 1 | 1 |
| 6230212218 | 2.97 | 0.89 |  | 1.82 | 0.6 | 0 | 1 | 1 |  | 0 | 0 | 1 | 1 | 1 |
| 6230212219 | 2.66 | 0.94 |  | 1.41 | 0.94 | 0 | 0 | 0 |  | 0 | 0 | 1 | 1 | 1 |
| 6230212220 | 3.17 | 1.27 |  | 1.58 | 0.59 | 1 | 1 | 1 |  | 0 | 1 | 1 | 1 | 1 |
| 6230212221 | 3 | 0.69 |  | 1.75 | 1.75 | 0 | 1 | 1 |  | 0 | 1 | 2 | 2 | 2 |
| 6230212222 | 3.6 | 1.13 |  | 1.94 | 1.73 | 0 | 1 | 1 |  | 0 | 0 | 1 | 1 | 1 |
| 6230212226 | 2.99 | 0.75 |  | 1.68 | 1.81 | 1 | 0 | 0 |  | 0 | 0 | 1 | 1 | 2 |
| 6230212228 | 4.1 | 0.99 |  | 2.55 | 1.94 | 0 | 1 | 0 |  | 0 | 0 | 1 | 1 | 1 |
| 6230212230 | 3.19 | 1.17 |  | 1.72 | 0.58 | 0 | 0 | 0 |  | 0 | 0 | 1 | 1 | 1 |
| 6230212233 | 3.68 | 0.82 |  | 2.62 | 1.24 | 1 | 0 | 0 |  | 0 | 0 | 2 | 1 | 2 |
| 6230212234 | 5.44 | 1.48 |  | 3.37 | 2.83 | 0 | 0 | 0 |  | 0 | 0 | 1 | 1 | 1 |
| 6230212235 | 1.79 | 0.74 |  | 0.76 | 0.89 | 0 | 0 | 0 |  | 0 | 0 | 1 | 1 | 2 |
| 6230212237 | 3.21 | 1.25 |  | 1.65 | 0.65 | 0 | 0 | 0 |  | 0 | 0 | 1 | 1 | 1 |
| 6230212238 | 3.87 | 1.13 |  | 2.22 | 1.19 | 0 | 0 | 0 |  | 0 | 0 | 1 | 1 | 1 |
| 6230212239 | 3.78 | 0.87 |  | 2.22 | 2.15 | 0 | 1 | 1 |  | 0 | 0 | 2 | 2 | 2 |
| 6230212240 | 2.93 | 1.29 |  | 1.37 | 0.65 | 0 | 0 | 0 |  | 0 | 0 | 1 | 1 | 1 |
| 6230212242 | 2.36 | 0.67 |  | 1.19 | 1.49 | 0 | 1 | 0 |  | 0 | 1 | 1 | 1 | 1 |
| 6230212244 | 3.54 | 1.13 |  | 1.96 | 1.23 | 0 | 1 | 1 |  | 0 | 1 | 2 | 1 | 2 |
| 6230212246 | 3.07 | 0.74 |  | 1.94 | 0.97 | 1 | 1 | 1 |  | 0 | 1 | 1 | 1 | 1 |
| 6230212247 | 3.25 | 0.77 |  | 2.12 | 0.93 | 1 | 0 | 0 |  | 0 | 0 | 1 | 1 | 1 |
| 6230212248 | 2.22 | 0.88 |  | 0.95 | 0.65 | 0 | 0 | 0 |  | 0 | 0 | 1 | 1 | 1 |
| 6230212249 | 4.15 | 1.16 |  | 2.27 | 1.54 | 2 | 0 | 0 |  | 0 | 0 | 1 | 1 | 2 |
| 6230212250 | 5.99 | 1.13 |  | 4.2 | 2.32 | 0 | 1 | 0 |  | 0 | 0 | 1 | 1 | 1 |
| 6230212251 | 3.32 | 1.46 |  | 1.43 | 1.02 | 0 | 0 | 2 |  | 0 | 0 | 1 | 1 | 1 |
| 6230212253 | 2.9 | 0.96 |  | 1.75 | 0.67 | 0 | 0 | 2 |  | 0 | 0 | 1 | 1 | 1 |
| 6230212301 | 3.39 | 0.87 |  | 1.93 | 1.27 | 0 | 0 | 0 |  | 0 | 0 | 1 | 1 | 2 |
| 6230212302 | 4.05 | 0.99 |  | 2.64 | 1.08 | 2 | 1 | 0 |  | 0 | 1 | 1 | 1 | 1 |
| 6230212308 | 3.67 | 0.9 |  | 1.72 | 2.3 | 2 | 0 | 0 |  | 0 | 0 | 1 | 1 | 2 |
| 6230212310 | 4.17 | 0.83 |  | 2.97 | 1.8 | 0 | 1 | 0 |  | 0 | 1 | 1 | 1 | 1 |
| 6230212311 | 3.51 | 1 |  | 2.13 | 0.96 | 1 | 1 | 1 |  | 0 | 0 | 2 | 2 | 3 |
| 6230212312 | 4.57 | 1.28 |  | 2.69 | 1.49 | 0 | 0 | 0 |  | 0 | 0 | 1 | 1 | 1 |
| 6230212315 | 2.73 | 0.77 |  | 1.13 | 2.08 | 1 | 0 | 1 |  | 0 | 0 | 1 | 1 | 1 |
| 6230212317 | 2.53 | 1.06 |  | 1.2 | 0.65 | 0 | 0 | 0 |  | 0 | 0 | 1 | 1 | 2 |
| 6230212318 | 4.76 | 1.16 |  | 3.28 | 1.24 | 2 | 1 | 0 |  | 0 | 1 | 1 | 1 | 2 |
| 6230212319 | 5.17 | 0.82 |  | 2.36 | 4.49 | 2 | 0 | 0 |  | 0 | 0 | 2 | 2 | 1 |
| 6230212320 | 3.78 | 1.1 |  | 2.19 | 1.38 | 0 | 0 | 0 |  | 0 | 0 | 1 | 1 | 3 |
| 6230212321 | 3.63 | 1.06 |  | 2.1 | 1.1 | 1 | 0 | 0 |  | 0 | 0 | 1 | 1 | 2 |
| 6230212323 | 4.11 | 0.98 |  | 2.56 | 1.26 | 0 | 0 | 1 |  | 0 | 0 | 2 | 2 | 3 |
| 6230212324 | 2.63 | 1.01 |  | 1.09 | 1.36 | 0 | 0 | 1 |  | 0 | 0 | 1 | 1 | 2 |
| 6230212325 | 2.81 | 1.04 |  | 1.46 | 0.64 | 0 | 0 | 1 |  | 0 | 0 | 2 | 2 | 1 |
| 6230212326 | 2.85 | 0.91 |  | 1.5 | 1.46 | 0 | 0 | 2 |  | 0 | 0 | 1 | 1 | 1 |
| 6230212327 | 2.92 | 1.35 |  | 1.16 | 0.57 | 0 | 1 | 0 |  | 0 | 0 | 1 | 1 | 1 |
| 6230212328 | 2.36 | 0.63 |  | 1.35 | 1.37 | 0 | 0 | 0 |  | 0 | 0 | 1 | 1 | 1 |
| 6230212329 | 2.96 | 1.08 |  | 1.66 | 0.74 | 0 | 0 | 0 |  | 0 | 0 | 1 | 1 | 1 |
| 6230212330 | 3.92 | 0.95 |  | 2.21 | 2.06 | 0 | 0 | 1 |  | 0 | 0 | 1 | 1 | 1 |
| 6230212331 | 4.01 | 1.22 |  | 2.38 | 1.24 | 0 | 0 | 1 |  | 0 | 0 | 1 | 1 | 1 |
| 6230212333 | 2.79 | 1.22 |  | 1.2 | 0.51 | 0 | 0 | 0 |  | 0 | 0 | 1 | 1 | 1 |
| 6230212335 | 2.74 | 0.98 |  | 1.46 | 0.83 | 0 | 0 | 1 |  | 0 | 0 | 1 | 1 | 1 |
| 6230212337 | 3.65 | 0.91 |  | 2.32 | 1.32 | 0 | 0 | 1 |  | 0 | 0 | 2 | 2 | 2 |
| 6230212338 | 2.85 | 1.23 |  | 1.16 | 0.68 | 1 | 0 | 0 |  | 0 | 0 | 1 | 1 | 2 |
| 6230212339 | 4.32 | 1.15 |  | 2.43 | 2.32 | 1 | 0 | 0 |  | 0 | 0 | 2 | 2 | 3 |
| 6230212341 | 4.04 | 0.96 |  | 2.43 | 1.99 | 0 | 0 | 1 |  | 0 | 0 | 1 | 1 | 3 |
| 6230212342 | 3.95 | 1.09 |  | 2.33 | 1.18 | 0 | 1 | 1 |  | 0 | 1 | 1 | 1 | 2 |
| 6230212343 | 3.63 | 1.09 |  | 2.24 | 0.99 | 0 | 0 | 1 |  | 0 | 0 | 1 | 1 | 1 |
| 6230212344 | 4.61 | 1.33 |  | 2.69 | 1.3 | 0 | 0 | 2 |  | 0 | 0 | 1 | 1 | 1 |
| 6230212345 | 4.05 | 0.79 |  | 1.73 | 5.6 | 0 | 0 | 0 |  | 0 | 0 | 2 | 2 | 3 |
| 6230212346 | 4.46 | 1.31 |  | 2.53 | 1.42 | 1 | 0 | 0 |  | 0 | 0 | 1 | 1 | 1 |
| 6230212347 | 3.87 | 0.78 |  | 2.62 | 1.35 | 0 | 1 | 1 |  | 0 | 0 | 1 | 1 | 1 |
| 6230212351 | 3 | 0.88 |  | 1.51 | 1.21 | 0 | 1 | 0 |  | 0 | 1 | 2 | 2 | 2 |
| 6230213101 | 2.55 | 0.9 |  | 1.21 | 1 | 2 | 1 | 0 |  | 0 | 0 | 1 | 1 | 1 |
| 6230213102 | 2.58 | 0.91 |  | 1.41 | 0.77 | 2 | 1 | 0 |  | 0 | 0 | 1 | 1 | 1 |
| 6230213103 | 2.47 | 1.03 |  | 1.05 | 0.75 | 0 | 0 | 0 |  | 0 | 0 | 1 | 1 | 1 |
| 6230213105 | 2.8 | 0.8 |  | 1.55 | 1.52 | 0 | 0 | 0 |  | 0 | 0 | 1 | 1 | 1 |
| 6230213106 | 3.33 | 0.84 |  | 2.04 | 1 | 2 | 0 | 0 |  | 0 | 0 | 1 | 1 | 1 |
| 6230213108 | 3.11 | 0.99 |  | 1.52 | 1.13 | 2 | 0 | 0 |  | 0 | 0 | 1 | 1 | 1 |
| 6230213109 | 2 | 0.56 |  | 0.98 | 0.96 | 2 | 0 | 0 |  | 0 | 0 | 1 | 1 | 1 |
| 6230213111 | 3.12 | 1.28 |  | 1.48 | 0.96 | 1 | 1 | 1 |  | 0 | 1 | 1 | 1 | 1 |
| 6230213113 | 5.43 | 0.76 |  | 1.8 | 6.35 | 2 | 0 | 0 |  | 0 | 0 | 2 | 2 | 2 |
| 6230213114 | 3.61 | 0.95 |  | 2.3 | 1.13 | 0 | 0 | 0 |  | 0 | 0 | 1 | 1 | 1 |
| 6230213115 | 3.49 | 0.88 |  | 1.95 | 1.7 | 2 | 0 | 0 |  | 0 | 0 | 2 | 2 | 1 |
| 6230213117 | 2 | 0.87 |  | 0.74 | 0.74 | 2 | 0 | 0 |  | 0 | 0 | 3 | 3 | 1 |
| 6230213118 | 2.62 | 0.74 |  | 1.48 | 0.77 | 1 | 0 | 0 |  | 0 | 0 | 3 | 3 | 2 |
| 6230213120 | 2.77 | 0.63 |  | 1.32 | 3.35 | 0 | 1 | 1 |  | 0 | 0 | 1 | 1 | 1 |
| 6230213122 | 2.02 | 0.84 |  | 0.94 | 0.43 | 0 | 0 | 0 |  | 0 | 0 | 1 | 1 | 2 |
| 6230213125 | 2.13 | 0.59 |  | 1.29 | 0.59 | 1 | 0 | 0 |  | 0 | 0 | 2 | 2 | 2 |
| 6230213126 | 3.07 | 0.81 |  | 1.92 | 0.98 | 0 | 1 | 1 |  | 0 | 0 | 1 | 1 | 1 |
| 6230213127 | 1.56 | 0.49 |  | 0.86 | 0.55 | 0 | 0 | 0 |  | 0 | 0 | 1 | 1 | 1 |
| 6230213128 | 2.28 | 0.94 |  | 1.05 | 0.46 | 1 | 0 | 0 |  | 0 | 0 | 2 | 1 | 1 |
| 6230213129 | 2.53 | 0.75 |  | 1.76 | 0.54 | 1 | 0 | 0 |  | 0 | 0 | 1 | 1 | 2 |
| 6230213131 | 2.49 | 0.83 |  | 1.44 | 0.64 | 1 | 1 | 1 |  | 0 | 0 | 1 | 1 | 1 |
| 6230213132 | 2.66 | 0.71 |  | 1.62 | 0.87 | 1 | 1 | 2 |  | 0 | 0 | 2 | 2 | 2 |
| 6230213133 | 2.73 | 1.11 |  | 1.06 | 1.14 | 1 | 0 | 0 |  | 0 | 0 | 1 | 1 | 2 |
| 6230213134 | 2.51 | 0.79 |  | 1.41 | 0.68 | 0 | 1 | 0 |  | 0 | 0 | 1 | 1 | 1 |
| 6230213135 | 2.1 | 0.8 |  | 0.95 | 0.84 | 0 | 0 | 0 |  | 0 | 0 | 1 | 1 | 1 |
| 6230213136 | 2.21 | 0.47 |  | 1.32 | 1.11 | 0 | 0 | 0 |  | 0 | 0 | 1 | 1 | 2 |
| 6230213137 | 2.62 | 0.97 |  | 1.33 | 0.75 | 0 | 0 | 0 |  | 0 | 0 | 3 | 2 | 2 |
| 6230213139 | 2.29 | 0.76 |  | 1.27 | 0.6 | 0 | 0 | 2 |  | 0 | 0 | 1 | 1 | 1 |
| 6230213140 | 2.78 | 0.81 |  | 1.19 | 2.1 | 1 | 1 | 0 |  | 0 | 0 | 1 | 1 | 1 |
| 6230213141 | 3.01 | 0.87 |  | 1.93 | 0.81 | 2 | 1 | 0 |  | 0 | 1 | 1 | 1 | 1 |
| 6230213144 | 2.8 | 0.74 |  | 1.65 | 1.2 | 0 | 0 | 0 |  | 0 | 0 | 1 | 1 | 1 |
| 6230213145 | 1.7 | 0.74 |  | 0.63 | 0.48 | 0 | 0 | 1 |  | 0 | 0 | 1 | 1 | 1 |
| 6230213146 | 2.58 | 0.92 |  | 1.21 | 1.19 | 0 | 0 | 0 |  | 0 | 0 | 1 | 1 | 1 |
| 6230213147 | 2.81 | 0.97 |  | 1.48 | 1.18 | 0 | 0 | 0 |  | 0 | 0 | 1 | 1 | 1 |
| 6230213148 | 2.82 | 0.9 |  | 1.43 | 1.09 | 2 | 1 | 0 |  | 0 | 0 | 1 | 1 | 1 |
| 6230213149 | 2.76 | 0.86 |  | 1.62 | 0.76 | 0 | 0 | 0 |  | 0 | 0 | 1 | 1 | 1 |
| 6230213150 | 2.34 | 0.59 |  | 1.25 | 1.19 | 0 | 0 | 0 |  | 0 | 0 | 1 | 1 | 1 |
| 6230213202 | 3.51 | 1.46 |  | 1.72 | 0.44 | 0 | 0 | 0 |  | 0 | 0 | 1 | 1 | 1 |
| 6230213204 | 2.77 | 0.97 |  | 1.4 | 1.37 | 0 | 0 | 0 |  | 0 | 0 | 1 | 1 | 1 |
| 6230213206 | 2.29 | 0.73 |  | 1.16 | 0.81 | 1 | 1 | 0 |  | 0 | 1 | 1 | 1 | 1 |
| 6230213207 | 2.24 | 0.69 |  | 1.4 | 0.51 | 0 | 1 | 0 |  | 0 | 0 | 1 | 1 | 2 |
| 6230213208 | 2.44 | 0.49 |  | 1.45 | 1.46 | 0 | 1 | 2 |  | 1 | 0 | 2 | 2 | 3 |
| 6230213209 | 2.05 | 0.53 |  | 1.18 | 0.97 | 1 | 0 | 0 |  | 0 | 0 | 2 | 1 | 1 |
| 6230213210 | 3.21 | 0.71 |  | 2.04 | 0.78 | 2 | 0 | 0 |  | 0 | 0 | 2 | 1 | 1 |
| 6230213212 | 2.74 | 0.91 |  | 1.52 | 0.62 | 0 | 0 | 0 |  | 0 | 0 | 1 | 1 | 1 |
| 6230213213 | 3.24 | 0.8 |  | 2.18 | 0.86 | 0 | 1 | 0 |  | 0 | 1 | 1 | 1 | 1 |
| 6230213216 | 2.7 | 0.77 |  | 1.58 | 0.9 | 1 | 0 | 0 |  | 0 | 0 | 1 | 1 | 2 |
| 6230213217 | 2.95 | 0.83 |  | 1.78 | 1.07 | 0 | 1 | 1 |  | 0 | 1 | 1 | 1 | 1 |
| 6230213218 | 1.62 | 0.58 |  | 0.71 | 0.65 | 1 | 0 | 0 |  | 0 | 0 | 1 | 1 | 1 |
| 6230213219 | 3 | 0.98 |  | 1.65 | 0.83 | 1 | 0 | 0 |  | 0 | 0 | 1 | 1 | 1 |
| 6230213220 | 2.6 | 1.02 |  | 1.14 | 1.13 | 0 | 1 | 1 |  | 0 | 0 | 1 | 1 | 1 |
| 6230213221 | 2.51 | 0.92 |  | 1.07 | 0.82 | 1 | 1 | 0 |  | 0 | 1 | 1 | 1 | 1 |
| 6230213223 | 3.3 | 0.95 |  | 1.89 | 0.92 | 1 | 1 | 1 |  | 0 | 1 | 1 | 1 | 1 |
| 6230213224 | 4.15 | 0.95 |  | 2.7 | 1.65 | 1 | 1 | 1 |  | 0 | 0 | 2 | 2 | 2 |
| 6230213225 | 3.09 | 0.92 |  | 1.88 | 0.9 | 1 | 0 | 1 |  | 0 | 0 | 1 | 1 | 1 |
| 6230213226 | 1.69 | 0.65 |  | 1.04 | 0.41 | 0 | 1 | 2 |  | 0 | 0 | 1 | 1 | 1 |
| 6230213227 | 2.42 | 0.87 |  | 1.14 | 0.81 | 1 | 0 | 0 |  | 0 | 0 | 1 | 1 | 1 |
| 6230213228 | 2.5 | 0.79 |  | 1.33 | 1.05 | 1 | 0 | 0 |  | 0 | 0 | 1 | 1 | 1 |
| 6230213230 | 3.26 | 1.08 |  | 1.68 | 0.97 | 2 | 1 | 0 |  | 0 | 1 | 1 | 1 | 1 |
| 6230213232 | 2.71 | 0.89 |  | 1.42 | 0.88 | 1 | 1 | 1 |  | 0 | 1 | 1 | 1 | 1 |
| 6230213235 | 1.59 | 0.65 |  | 0.72 | 0.31 | 0 | 0 | 1 |  | 0 | 0 | 2 | 1 | 2 |
| 6230213237 | 1.76 | 0.82 |  | 0.73 | 0.69 | 0 | 0 | 0 |  | 0 | 0 | 2 | 2 | 2 |
| 6230213239 | 2.29 | 0.82 |  | 1.1 | 0.81 | 1 | 1 | 1 |  | 0 | 1 | 1 | 1 | 1 |
| 6230213242 | 2.67 | 0.89 |  | 1.42 | 0.77 | 1 | 1 | 2 |  | 0 | 0 | 2 | 2 | 2 |
| 6230213244 | 1.52 | 0.54 |  | 0.79 | 0.47 | 0 | 1 | 1 |  | 0 | 0 | 1 | 1 | 1 |
| 6230213247 | 2.11 | 0.72 |  | 1.08 | 0.74 | 2 | 1 | 0 |  | 0 | 1 | 1 | 1 | 1 |
| 6230213248 | 2.95 | 1.3 |  | 1.25 | 0.88 | 0 | 1 | 1 |  | 0 | 1 | 1 | 1 | 1 |
| 6230213249 | 2.13 | 0.59 |  | 0.98 | 1.44 | 1 | 0 | 0 |  | 0 | 0 | 1 | 1 | 1 |
| 6230213251 | 3.11 | 0.89 |  | 1.83 | 1.27 | 1 | 1 | 0 |  | 1 | 1 | 2 | 2 | 2 |
| 6230213252 | 2.47 | 0.81 |  | 1.29 | 0.87 | 1 | 0 | 0 |  | 0 | 0 | 1 | 1 | 2 |
| 6230213253 | 3.03 | 1.08 |  | 1.61 | 0.9 | 1 | 0 | 0 |  | 0 | 0 | 2 | 1 | 2 |
| 6230213301 | 2.49 | 0.83 |  | 1.38 | 0.65 | 1 | 0 | 0 |  | 0 | 0 | 1 | 1 | 1 |
| 6230213302 | 2.77 | 0.76 |  | 1.57 | 1.33 | 1 | 1 | 2 |  | 0 | 0 | 1 | 1 | 1 |
| 6230213305 | 2.69 | 0.81 |  | 1.54 | 0.8 | 1 | 1 | 0 |  | 0 | 1 | 1 | 1 | 1 |
| 6230213306 | 3.34 | 1.31 |  | 1.4 | 1.03 | 2 | 1 | 0 |  | 0 | 0 | 1 | 1 | 1 |
| 6230213309 | 2.45 | 0.75 |  | 1.08 | 2.04 | 0 | 0 | 0 |  | 0 | 0 | 1 | 1 | 1 |
| 6230213310 | 2.44 | 0.73 |  | 1.23 | 1.25 | 1 | 0 | 0 |  | 0 | 0 | 1 | 1 | 1 |
| 6230213312 | 3.05 | 0.88 |  | 1.53 | 1.94 | 1 | 1 | 1 |  | 0 | 1 | 1 | 1 | 1 |
| 6230213313 | 2.97 | 1.03 |  | 1.57 | 0.77 | 2 | 1 | 1 |  | 0 | 1 | 1 | 1 | 1 |
| 6230213322 | 3.71 | 1.17 |  | 1.98 | 1.19 | 1 | 0 | 0 |  | 0 | 0 | 1 | 1 | 1 |
| 6230213323 | 2.43 | 0.76 |  | 1.34 | 0.88 | 0 | 0 | 0 |  | 0 | 0 | 2 | 1 | 1 |
| 6230213326 | 3.61 | 0.9 |  | 2.02 | 2.11 | 2 | 0 | 0 |  | 0 | 0 | 1 | 1 | 1 |
| 6230213329 | 2.57 | 0.82 |  | 1.25 | 0.97 | 1 | 0 | 0 |  | 0 | 0 | 1 | 1 | 1 |
| 6230213331 | 3.13 | 0.83 |  | 1.91 | 0.57 | 1 | 0 | 0 |  | 0 | 0 | 2 | 2 | 2 |
| 6230213332 | 4.07 | 1.39 |  | 2.43 | 0.64 | 0 | 0 | 0 |  | 0 | 0 | 1 | 1 | 1 |
| 6230213334 | 1.65 | 0.53 |  | 0.87 | 0.55 | 1 | 0 | 0 |  | 0 | 0 | 2 | 2 | 1 |
| 6230213336 | 3.35 | 0.84 |  | 2.26 | 0.61 | 0 | 1 | 0 |  | 0 | 1 | 1 | 1 | 1 |
| 6230213338 | 3.15 | 1.14 |  | 1.71 | 0.63 | 0 | 1 | 0 |  | 0 | 0 | 1 | 1 | 1 |
| 6230213340 | 2.52 | 0.92 |  | 1.41 | 0.7 | 1 | 0 | 0 |  | 0 | 0 | 1 | 1 | 1 |
| 6230213342 | 2.07 | 0.49 |  | 1.19 | 1.15 | 1 | 0 | 0 |  | 0 | 0 | 1 | 1 | 1 |
| 6230213343 | 2.6 | 0.92 |  | 1.49 | 0.53 | 1 | 0 | 0 |  | 0 | 0 | 1 | 1 | 1 |
| 6230213344 | 2.41 | 0.59 |  | 1.34 | 1.59 | 2 | 1 | 0 |  | 0 | 0 | 1 | 1 | 1 |
| 6230213347 | 4.1 | 1.49 |  | 2.1 | 0.74 | 1 | 1 | 0 |  | 0 | 0 | 1 | 1 | 1 |
| 6230213350 | 3.13 | 1 |  | 1.75 | 0.86 | 1 | 0 | 0 |  | 0 | 0 | 3 | 3 | 1 |
| 6230213351 | 3.18 | 0.89 |  | 1.86 | 0.71 | 2 | 1 | 0 |  | 0 | 0 | 1 | 1 | 1 |
| 6230214101 | 2.99 | 1.03 |  | 1.72 | 0.75 | 1 | 0 | 0 |  | 0 | 0 | 1 | 1 | 1 |
| 6230214102 | 2.59 | 0.75 |  | 1.48 | 0.68 | 1 | 1 | 2 |  | 0 | 1 | 1 | 1 | 1 |
| 6230214103 | 2.17 | 1.02 |  | 0.87 | 0.48 | 0 | 1 | 0 |  | 0 | 1 | 1 | 1 | 1 |
| 6230214105 | 3.32 | 0.95 |  | 1.85 | 1.36 | 1 | 0 | 0 |  | 0 | 0 | 2 | 1 | 2 |
| 6230214109 | 2.83 | 1.1 |  | 1.41 | 0.65 | 0 | 0 | 0 |  | 0 | 0 | 2 | 1 | 1 |
| 6230214111 | 3.06 | 0.83 |  | 1.84 | 0.85 | 2 | 0 | 0 |  | 0 | 0 | 1 | 1 | 1 |
| 6230214112 | 2.75 | 0.81 |  | 1.87 | 0.55 | 0 | 0 | 0 |  | 0 | 0 | 2 | 2 | 2 |
| 6230214113 | 2.32 | 0.74 |  | 1.06 | 1.47 | 0 | 0 | 0 |  | 0 | 0 | 1 | 1 | 1 |
| 6230214115 | 3.8 | 0.97 |  | 2.34 | 1.3 | 2 | 0 | 0 |  | 0 | 0 | 1 | 1 | 1 |
| 6230214118 | 3.27 | 1.23 |  | 1.52 | 0.85 | 2 | 1 | 0 |  | 0 | 0 | 1 | 1 | 1 |
| 6230214120 | 3.45 | 1.05 |  | 2.01 | 1.18 | 0 | 0 | 0 |  | 0 | 0 | 1 | 1 | 1 |
| 6230214122 | 3.49 | 0.86 |  | 1.87 | 1.99 | 0 | 0 | 0 |  | 0 | 0 | 1 | 1 | 1 |
| 6230214124 | 1.95 | 0.46 |  | 1.13 | 0.74 | 1 | 0 | 0 |  | 0 | 0 | 2 | 2 | 2 |
| 6230214125 | 2.93 | 0.77 |  | 1.93 | 0.9 | 2 | 1 | 0 |  | 0 | 0 | 3 | 2 | 3 |
| 6230214126 | 3.66 | 1.18 |  | 2.06 | 1.08 | 2 | 1 | 0 |  | 0 | 0 | 2 | 2 | 2 |
| 6230214127 | 2.39 | 0.66 |  | 1.3 | 1.2 | 1 | 0 | 0 |  | 0 | 0 | 1 | 1 | 1 |
| 6230214128 | 1.73 | 0.66 |  | 0.7 | 0.67 | 0 | 0 | 0 |  | 0 | 0 | 1 | 1 | 1 |
| 6230214129 | 1.98 | 0.61 |  | 1.01 | 0.92 | 0 | 0 | 0 |  | 0 | 0 | 1 | 1 | 2 |
| 6230214131 | 3.71 | 1.36 |  | 1.79 | 1.15 | 2 | 1 | 0 |  | 0 | 1 | 1 | 1 | 1 |
| 6230214135 | 3.78 | 1.2 |  | 2.29 | 0.84 | 1 | 0 | 0 |  | 0 | 0 | 1 | 1 | 1 |
| 6230214137 | 2.99 | 0.94 |  | 1.75 | 0.84 | 0 | 0 | 0 |  | 0 | 0 | 1 | 1 | 1 |
| 6230214138 | 2.32 | 0.74 |  | 1.28 | 0.67 | 2 | 0 | 0 |  | 0 | 0 | 1 | 1 | 1 |
| 6230214139 | 2.61 | 0.83 |  | 1.49 | 0.82 | 1 | 0 | 0 |  | 0 | 0 | 1 | 1 | 1 |
| 6230214140 | 2.71 | 0.73 |  | 1.59 | 1.39 | 0 | 1 | 1 |  | 0 | 0 | 2 | 2 | 3 |
| 6230214141 | 3.48 | 0.91 |  | 2.24 | 0.87 | 1 | 0 | 0 |  | 0 | 0 | 2 | 1 | 2 |
| 6230214142 | 3.35 | 1.06 |  | 1.79 | 0.81 | 2 | 1 | 0 |  | 0 | 1 | 1 | 1 | 1 |
| 6230214143 | 4.35 | 1.13 |  | 2.75 | 0.9 | 0 | 1 | 1 |  | 0 | 1 | 1 | 1 | 1 |
| 6230214144 | 3.24 | 1.01 |  | 1.79 | 0.93 | 2 | 1 | 0 |  | 0 | 0 | 1 | 1 | 1 |
| 6230214147 | 2.07 | 0.64 |  | 1.07 | 0.77 | 0 | 0 | 0 |  | 0 | 0 | 1 | 1 | 1 |
| 6230214148 | 3.78 | 1.17 |  | 2.26 | 1 | 1 | 0 | 0 |  | 0 | 0 | 1 | 1 | 1 |
| 6230214149 | 3.05 | 1.04 |  | 1.5 | 1.02 | 0 | 0 | 0 |  | 0 | 0 | 2 | 2 | 2 |
| 6230214151 | 3.18 | 0.99 |  | 2.05 | 0.49 | 0 | 0 | 0 |  | 0 | 0 | 1 | 1 | 1 |
| 6230214152 | 2.21 | 0.72 |  | 1.21 | 1.07 | 0 | 0 | 0 |  | 0 | 0 | 1 | 1 | 1 |
| 6230214153 | 2.56 | 0.89 |  | 1.17 | 1.31 | 0 | 0 | 0 |  | 0 | 0 | 1 | 1 | 1 |
| 6230214201 | 2.48 | 0.56 |  | 1.58 | 1.06 | 0 | 0 | 0 |  | 0 | 0 | 2 | 2 | 2 |
| 6230214203 | 3.24 | 1.08 |  | 1.69 | 0.84 | 0 | 1 | 0 |  | 0 | 0 | 1 | 1 | 1 |
| 6230214204 | 2.63 | 0.81 |  | 1.41 | 0.84 | 0 | 1 | 2 |  | 0 | 0 | 2 | 2 | 1 |
| 6230214205 | 3.18 | 1.04 |  | 1.76 | 1.26 | 0 | 0 | 0 |  | 0 | 0 | 2 | 2 | 2 |
| 6230214206 | 3.67 | 0.82 |  | 1.77 | 4.02 | 0 | 0 | 0 |  | 0 | 0 | 1 | 1 | 1 |
| 6230214207 | 2.56 | 0.89 |  | 1.46 | 0.51 | 0 | 1 | 1 |  | 0 | 1 | 1 | 1 | 1 |
| 6230214208 | 3.1 | 0.8 |  | 1.53 | 2.2 | 0 | 1 | 0 |  | 0 | 1 | 2 | 2 | 1 |
| 6230214209 | 2.24 | 0.87 |  | 0.9 | 1.13 | 2 | 0 | 0 |  | 0 | 0 | 1 | 1 | 1 |
| 6230214211 | 4.38 | 1.39 |  | 2.46 | 1.09 | 0 | 1 | 2 |  | 0 | 1 | 1 | 1 | 1 |
| 6230214212 | 3.45 | 1 |  | 1.96 | 1.02 | 1 | 0 | 0 |  | 0 | 0 | 1 | 1 | 1 |
| 6230214213 | 3.03 | 0.77 |  | 1.58 | 1.82 | 2 | 0 | 0 |  | 0 | 0 | 2 | 1 | 2 |
| 6230214214 | 2.21 | 0.67 |  | 1.25 | 0.78 | 2 | 0 | 0 |  | 0 | 0 | 3 | 2 | 3 |
| 6230214215 | 5.14 | 1.72 |  | 2.83 | 1.26 | 0 | 1 | 0 |  | 0 | 0 | 1 | 1 | 1 |
| 6230214217 | 2.71 | 1.05 |  | 1.26 | 0.91 | 0 | 1 | 1 |  | 0 | 1 | 1 | 1 | 1 |
| 6230214219 | 3.72 | 1.11 |  | 2.21 | 0.92 | 0 | 0 | 0 |  | 0 | 0 | 1 | 1 | 1 |
| 6230214220 | 3.78 | 1.08 |  | 2.07 | 1.34 | 2 | 0 | 0 |  | 0 | 0 | 1 | 1 | 2 |
| 6230214222 | 1.68 | 0.64 |  | 0.72 | 1.04 | 0 | 0 | 0 |  | 0 | 0 | 1 | 1 | 1 |
| 6230214224 | 2.27 | 1.12 |  | 0.94 | 0.41 | 0 | 0 | 0 |  | 0 | 0 | 1 | 1 | 1 |
| 6230214225 | 2.91 | 0.88 |  | 1.35 | 1.9 | 0 | 1 | 0 |  | 0 | 1 | 2 | 1 | 2 |
| 6230214226 | 2.8 | 0.93 |  | 1.38 | 0.85 | 0 | 1 | 1 |  | 0 | 1 | 1 | 1 | 1 |
| 6230214227 | 2.96 | 0.82 |  | 1.8 | 1.34 | 0 | 0 | 0 |  | 0 | 0 | 2 | 2 | 1 |
| 6230214228 | 3.73 | 1.05 |  | 2.13 | 1.12 | 2 | 0 | 0 |  | 0 | 0 | 1 | 1 | 1 |
| 6230214229 | 2.89 | 1.05 |  | 1.4 | 0.72 | 0 | 0 | 1 |  | 0 | 0 | 1 | 1 | 1 |
| 6230214230 | 3.42 | 1.03 |  | 1.74 | 2.14 | 1 | 0 | 0 |  | 0 | 0 | 2 | 2 | 2 |
| 6230214231 | 3.72 | 1.09 |  | 2.14 | 1.73 | 0 | 0 | 0 |  | 0 | 0 | 1 | 1 | 1 |
| 6230214232 | 4.25 | 0.96 |  | 2.46 | 1.73 | 2 | 0 | 0 |  | 0 | 0 | 2 | 1 | 1 |
| 6230214233 | 3.07 | 0.82 |  | 1.87 | 1.3 | 0 | 1 | 0 |  | 0 | 1 | 1 | 1 | 1 |
| 6230214236 | 6.65 | 1.41 |  | 4.01 | 3.47 | 0 | 1 | 1 |  | 0 | 1 | 1 | 1 | 1 |
| 6230214237 | 3.52 | 0.8 |  | 2.24 | 1.2 | 1 | 1 | 0 |  | 0 | 1 | 1 | 1 | 1 |
| 6230214238 | 4.89 | 1.45 |  | 2.69 | 1.97 | 1 | 0 | 0 |  | 0 | 0 | 1 | 1 | 1 |
| 6230214239 | 2.29 | 0.83 |  | 0.93 | 0.99 | 0 | 0 | 0 |  | 0 | 0 | 1 | 1 | 1 |
| 6230214242 | 3.49 | 0.83 |  | 2.41 | 0.79 | 2 | 1 | 1 |  | 0 | 0 | 1 | 1 | 1 |
| 6230214243 | 2.84 | 1.25 |  | 1.19 | 0.76 | 0 | 0 | 1 |  | 0 | 0 | 1 | 1 | 1 |
| 6230214244 | 3.62 | 0.95 |  | 2.32 | 1.15 | 0 | 0 | 0 |  | 0 | 0 | 2 | 1 | 2 |
| 6230214245 | 2.25 | 0.76 |  | 0.79 | 2.01 | 0 | 0 | 1 |  | 0 | 0 | 3 | 2 | 1 |
| 6230214246 | 2.5 | 1.14 |  | 0.91 | 0.92 | 2 | 0 | 0 |  | 0 | 0 | 1 | 1 | 1 |
| 6230214247 | 3.51 | 0.88 |  | 2.19 | 1.31 | 0 | 0 | 1 |  | 0 | 0 | 2 | 2 | 1 |
| 6230214248 | 3.09 | 0.88 |  | 2.01 | 0.77 | 2 | 1 | 0 |  | 0 | 1 | 1 | 1 | 1 |
| 6230214249 | 3.54 | 1.28 |  | 1.73 | 1.34 | 1 | 0 | 0 |  | 0 | 0 | 1 | 1 | 1 |
| 6230214250 | 2.79 | 0.91 |  | 1.54 | 0.8 | 1 | 1 | 0 |  | 0 | 1 | 1 | 1 | 1 |
| 6230214251 | 2.87 | 0.89 |  | 1.55 | 0.99 | 0 | 0 | 0 |  | 0 | 0 | 1 | 1 | 2 |
| 6230214301 | 4.79 | 0.97 |  | 2.73 | 2.64 | 0 | 1 | 1 |  | 1 | 1 | 1 | 1 | 1 |
| 6230214303 | 3.41 | 0.63 |  | 1.79 | 3.37 | 0 | 1 | 1 |  | 0 | 0 | 3 | 2 | 3 |
| 6230214304 | 2.88 | 0.96 |  | 1.59 | 0.67 | 0 | 0 | 0 |  | 0 | 0 | 1 | 1 | 1 |
| 6230214307 | 4.4 | 1.04 |  | 2.74 | 2.9 | 0 | 1 | 0 |  | 0 | 1 | 1 | 1 | 1 |
| 6230214309 | 1.95 | 0.81 |  | 0.82 | 0.7 | 0 | 0 | 1 |  | 0 | 0 | 1 | 1 | 1 |
| 6230214310 | 3.17 | 1.11 |  | 1.71 | 1.08 | 0 | 0 | 0 |  | 0 | 0 | 1 | 1 | 1 |
| 6230214311 | 4.32 | 0.91 |  | 2.77 | 1.68 | 1 | 0 | 0 |  | 0 | 0 | 1 | 1 | 1 |
| 6230214312 | 2.95 | 0.94 |  | 1.55 | 1.37 | 0 | 0 | 1 |  | 0 | 0 | 1 | 1 | 1 |
| 6230214315 | 3.18 | 1.09 |  | 1.57 | 2.04 | 0 | 0 | 1 |  | 0 | 0 | 1 | 1 | 1 |
| 6230214317 | 2.57 | 0.9 |  | 1.36 | 0.83 | 1 | 0 | 0 |  | 0 | 0 | 1 | 1 | 1 |
| 6230214318 | 4.03 | 1.05 |  | 2.55 | 1.81 | 0 | 1 | 1 |  | 0 | 0 | 1 | 1 | 2 |
| 6230214320 | 2.62 | 0.94 |  | 0.91 | 1.91 | 0 | 0 | 1 |  | 0 | 0 | 2 | 1 | 1 |
| 6230214321 | 2.67 | 0.82 |  | 1.46 | 1.06 | 0 | 0 | 0 |  | 0 | 0 | 1 | 1 | 1 |
| 6230214322 | 2.27 | 0.72 |  | 1.18 | 0.88 | 2 | 0 | 0 |  | 0 | 0 | 1 | 1 | 1 |
| 6230214323 | 4.06 | 0.86 |  | 2.65 | 1.25 | 0 | 0 | 0 |  | 0 | 0 | 1 | 1 | 1 |
| 6230214324 | 3.56 | 1.23 |  | 1.9 | 1.58 | 0 | 1 | 0 |  | 0 | 0 | 1 | 1 | 1 |
| 6230214325 | 2.49 | 0.71 |  | 1.19 | 1.72 | 0 | 0 | 0 |  | 0 | 0 | 1 | 1 | 1 |
| 6230214326 | 2.88 | 0.79 |  | 1.51 | 1.86 | 0 | 0 | 1 |  | 0 | 0 | 2 | 2 | 1 |
| 6230214327 | 2.42 | 0.78 |  | 1.38 | 0.64 | 1 | 0 | 0 |  | 0 | 0 | 1 | 1 | 1 |
| 6230214329 | 4.25 | 1.35 |  | 2.47 | 1.04 | 1 | 0 | 0 |  | 0 | 0 | 1 | 1 | 1 |
| 6230214330 | 3.01 | 1.04 |  | 1.68 | 1.41 | 1 | 1 | 1 |  | 0 | 1 | 2 | 2 | 2 |
| 6230214331 | 2.95 | 0.62 |  | 1.77 | 1.66 | 0 | 1 | 1 |  | 0 | 1 | 1 | 1 | 1 |
| 6230214332 | 4.14 | 0.88 |  | 2.66 | 2.85 | 0 | 1 | 1 |  | 1 | 0 | 1 | 1 | 1 |
| 6230214333 | 3.75 | 1.17 |  | 2.38 | 0.68 | 1 | 1 | 0 |  | 0 | 1 | 1 | 1 | 1 |
| 6230214334 | 3.31 | 1.1 |  | 1.8 | 1.41 | 0 | 1 | 0 |  | 0 | 1 | 1 | 1 | 1 |
| 6230214336 | 3.98 | 1.13 |  | 1.97 | 2.84 | 2 | 0 | 0 |  | 0 | 0 | 2 | 1 | 2 |
| 6230214338 | 4.57 | 1.48 |  | 2.63 | 1.39 | 1 | 1 | 1 |  | 0 | 0 | 1 | 1 | 1 |
| 6230214339 | 4.02 | 0.98 |  | 2.55 | 2.71 | 1 | 1 | 0 |  | 0 | 1 | 1 | 1 | 1 |
| 6230214342 | 2.23 | 0.68 |  | 1.16 | 1.09 | 0 | 0 | 0 |  | 0 | 0 | 1 | 1 | 1 |
| 6230214343 | 2.58 | 0.66 |  | 1.43 | 1.05 | 0 | 0 | 0 |  | 0 | 0 | 1 | 1 | 1 |
| 6230214344 | 3.96 | 1.27 |  | 2.26 | 0.89 | 0 | 0 | 0 |  | 0 | 0 | 2 | 2 | 1 |
| 6230214345 | 3.31 | 1.24 |  | 1.57 | 1.08 | 0 | 0 | 0 |  | 0 | 0 | 1 | 1 | 1 |
| 6230214346 | 3.58 | 1.16 |  | 1.71 | 1.53 | 1 | 1 | 0 |  | 0 | 1 | 1 | 1 | 2 |
| 6230214347 | 2.41 | 0.74 |  | 1.38 | 0.93 | 0 | 1 | 2 |  | 1 | 1 | 1 | 1 | 1 |
| 6230214348 | 2.32 | 0.88 |  | 0.91 | 1.62 | 1 | 0 | 0 |  | 0 | 0 | 1 | 1 | 1 |
| 6230214350 | 2.85 | 0.78 |  | 1.48 | 1.14 | 1 | 0 | 0 |  | 0 | 0 | 1 | 1 | 1 |
| 6230214351 | 2.44 | 0.87 |  | 1.26 | 0.56 | 0 | 0 | 1 |  | 0 | 0 | 1 | 1 | 1 |
| 6230214353 | 2.98 | 0.95 |  | 1.64 | 1.65 | 0 | 0 | 1 |  | 0 | 0 | 1 | 1 | 2 |
